# Targeting Anti-Apoptotic Bcl-2 Proteins with Triterpene-Heterocyclic Derivatives: A Combined Dual Docking and Molecular Dynamics Study

**DOI:** 10.3390/molecules30193919

**Published:** 2025-09-29

**Authors:** Marius Mioc, Silvia Gruin, Armand Gogulescu, Oana Bătrîna, Mihaela Jorgovan, Bogdan-Ionuț Mara, Codruța Șoica

**Affiliations:** 1 Faculty of Pharmacy, “Victor Babes” University of Medicine and Pharmacy, Eftimie Murgu Square, No. 2, 300041 Timisoara, Romania; marius.mioc@umft.ro (M.M.); oana.esanu@umft.ro (O.B.); mihaela.coban@umft.ro (M.J.); mara.bogdan@umft.ro (B.-I.M.); codrutasoica@umft.ro (C.Ș.); 2 Research Center for Experimental Pharmacology and Drug Design (X-Pharm Design), “Victor Babes” University of Medicine and Pharmacy, Eftimie Murgu Square, No. 2, 300041 Timișoara, Romania; 3 Faculty of Medicine, “Victor Babes” University of Medicine and Pharmacy, Eftimie Murgu Square, No. 2, 300041 Timisoara, Romania; silvia.gruin@gmail.com; 4 Coriolan Dragulescu Institute of Chemistry, Romanian Academy, Bv. M. Viteazu, No. 24, 300223 Timisoara, Romania

**Keywords:** Bcl2 family inhibitors, triterpene derivatives, molecular docking, Vina, Glide, molecular dynamics simulation

## Abstract

Anti-apoptotic Bcl-2 family proteins (Bcl-2, Bcl-xL, and Mcl-1), are often overexpressed in cancer, which aids tumor growth and treatment resistance. As a result, these proteins are excellent candidates for novel anticancer drugs. Within this study a virtual library of betuline derivatives was built and screened for possible Bcl-2, Bcl-XL, and Mcl-1 inhibitors. For every target, molecular docking simulations were performed using two different engines (AutoDock Vina and Glide). The ligands that most frequently appeared among the top candidates were shortlisted after comparing the top-20 hits from both docking scoring functions. To assess binding stability, five of these promising compounds were chosen and run through 100 ns molecular dynamics (MD) simulations in complex with every target protein. Key persistent intermolecular contacts were identified from MD contact frequency histograms, and stability was evaluated using root-mean-square deviation (RMSD) profiles of protein–ligand complexes following equilibration. Additionally, Prime MM-GBSA binding energies (ΔG_bind) for the 15 docked complexes were computed, and ligand efficiency was reported. Two substances, BOxNaf1 and BT3, stood out among the screened derivatives as the most stable binders to all three Bcl-2 family targets according to the dual docking and MD analysis approach. When the MM-GBSA and RMSF/rGyr data are considered alongside docking and MD stability, BOxNaf1 and BOxPhCl1 emerge as the most compelling dual/multi-target candidates, whereas BT3, though MD stable, shows weaker MM-GBSA energetics and is retained as a lower-priority backup chemotype.

## 1. Introduction

Over the past couple of decades, anticancer drug research has encountered numerous challenges yet achieved substantial progress in improving cancer treatment and management. Targeted therapies have been particularly important in improving patient outcomes. Even so, much work remains before these advances can significantly transform the overall landscape of cancer.

The Bcl2 protein family, which is intimately involved in apoptosis regulation, is one of the most studied targetable protein families in anticancer drug development research. Inhibiting anti-apoptotic proteins such as Bcl-2 or Bcl-XL is clearly beneficial in a variety of cancers. However, some of these selective inhibitors share a common drawback: resistance to therapy, as demonstrated by the case of the selective Bcl-2 inhibitor venetoclax [1]. The development of Bcl-2/Bcl-XL dual inhibitors has been a viable option that has stormed this research field, from Brunko et al., who claimed “the first dual, subnanomolar inhibitors of Bcl-xL and Bcl-2” [2] to the present day, where compounds like navitoclax, or obatoclax, are still being evaluated after more than a decade since their discovery [3,4]. More recent Bcl-2/Bcl-XL inhibitors like AZD4320 [5] have been biologically evaluated, indicating that the race to find these inhibitors is still on because each of them still has some drawbacks left to overcome. One major limitation shared by many of these agents is the inability to overcome Mcl-1 upregulation, which can compensate for Bcl-2/Bcl-XL inhibition and maintain cell survival. This MCL-1-mediated resistance mechanism has been documented in the case of venetoclax and observed in preclinical and clinical evaluations of dual inhibitors, highlighting the need for strategies that also target MCL-1 [6,7,8].

The common theme in this landscape is that all of these small molecule inhibitors are pure synthetic compounds. Some of the technological challenges associated with synthesizing these compounds include their large, complex structures with multiple rings, which necessitate multiple steps in synthesis and purification, potentially leading to unnecessary labor costs.

**Figure 1:**
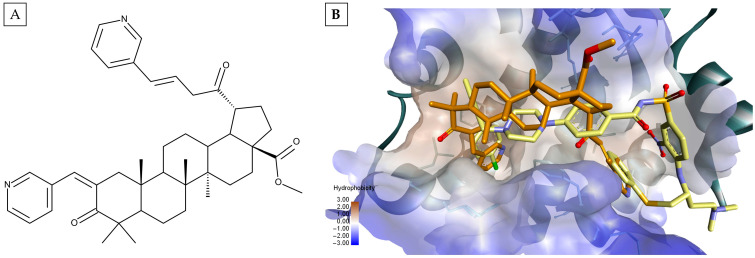
Chemical structure of the lupane-type pyrilydene derivative, methyl 3,20-dioxo-2,30-di-(3-pyridinylidene)-29-nor-lup-28-oate (**A**) and the docked pose of the same compound (orange) into the binding site of Bcl-XL, overlapped with the cocrystalized pose of the native ligand ABT-737 (yellow) (PDB ID: 2YXJ) (**B**).

The current study proposes a different approach by utilizing triterpenes as starting scaffolds for the design of triple Bcl-2/Bcl-XL/MCL-1 inhibitors. Pentacyclic triterpenes, such as betulinic acid (BA), Betulin (Bet), oleanolic acid (OA), and ursolic acid (UA), have been shown to induce cancer cell cytotoxicity by destabilizing the pro/antiapoptotic protein ratio of the Bcl-2 family [9,10], and they have been available in the scientific spotlight for quite some time. Previous docking studies [11,12] revealed that the triterpene scaffold alone binds to the large surface-accessible hydrophobic groove, roughly delimited by the α3, α4, and α5 helices found in the structure of Bcl-2 and Bcl-XL. However, in most cases, the analyzed triterpene derivatives lacked the necessary substitutions in order to reach the deeper hydrophobic pockets found in the Bcl-2/Bcl-XL binding sites. Despite all of that, we were particularly interested in one intriguing case. We investigated two pyrylidene triterpene derivatives featuring two pyridyl groups attached to the triterpenic core at approximately opposite ends. These substances displayed GI_50_ values on various cancer cell lines ranging from 6 μM to the nanomolar range [12]. Further research showed that these substances downregulated Bcl-XL and Bcl-2 antiapoptotic proteins while upregulating proapoptotic Bak and Bad genes. Molecular docking results, calculated using Autodock Vina’s scoring function, revealed that the Bcl-XL inhibitor ABT-737 was outperformed by the lupane-type pyrilydene derivative (Figure 1A). The docking analysis demonstrated that the two substituted ramifications with pyridyl rings at the end were appropriate for the structure to accommodate 2 out of the 4 deep hydrophobic binding site pockets, similarly to ABT-737 (Figure 1B). These observations served as a primary factor leading to the design of our triterpene-based antiapoptotic protein inhibitors.

The idea of designing triple Bcl-2/Bcl-XL/Mcl-1 inhibitors using triterpenes is innovative from a scientific standpoint because, as far as we are aware, it has never been attempted. Additionally, triterpenes like BA, Bet, OA, and UA already have a proapoptotic effect related to the Bcl-2 protein family regulation, which is a significant advantage. The extensive body of literature on pentacyclic triterpenes’ anticancer effects indicates that these substances are safe and have selective anticancer in vitro activity [13,14]. From a synthetic perspective, these compounds will be designed in such a way that allows for simple synthesis using workable reaction pathways and a minimum number of intermediate reactions. For the current study, Bet was chosen as the main scaffold used as a starting molecule for the design of our compounds. This choice was based on a few factors such as: Bet is a naturally occurring molecule, easily obtainable in high yields, it is also commercially available and has a significantly reduced cost compared to other triterpenes like BA, OA, or UA.

According to numerous previously reported studies, including our own, triazoles, oxadiazoles, and other related heterocyclic compounds boost the cytotoxic activity of pentacyclic triterpenes. Therefore, when building our compound library, we chose to incorporate these types of heterocycles into the structure of the designed compounds.

The current study aimed to design a series of compounds based on betulin’s scaffold, each bearing two heterocyclic groups at the 3- and 28-positions through variable carbon length spacers, and to evaluate them in silico as potential triple Bcl-2/Bcl-XL/Mcl-1 inhibitors. As part of our ligand design rationale, we created ligands with a triterpenic core that occupies the central hydrophobic BH3 groove (as previously mentioned) and two heterocycles that are connected by variable-length spacers to reach the P2 and P4 subpockets and serve as H-bond donors/acceptors and systems to stabilize hydrogen-bonding and π-interactions (π-π/cation-π) with groove residues (see Section 3.1). For this purpose, all compounds were docked into the three targets using two different software (Vina 1.1.2 and Glide), ranked, and the top candidates consistently appearing in the top 20 for all proteins were selected. The selected compounds were further subjected to molecular dynamics simulations, and those demonstrating favorable stability were considered suitable for synthesis and biological evaluation.

## 2. Results and Discussion

### 2.1. Molecular Docking and Candidate Selection

Molecular docking simulations for the Bet derivatives against the three antiapoptotic proteins (Bcl-2, Bcl-XL, and Mcl-1) were performed with both Autodock Vina and Glide. The full docking results, containing docking scores for each protein, ranked by both docking scores, are available in Tables S2–S7 (Supplementary Material). In order to highlight only the highest theoretically active candidates, a top-20 selection of unique compounds (only the best conformation/candidate molecule was kept) was made for each set of docking score pairs corresponding to the three protein targets. Following this selection, we counted the frequency of each compound across all six top-20 score lists. Results are depicted in Table 1, where cells containing compounds that appeared at least 4 times are highlighted in different colours. Bet and BA were not present in any of the 6 top-20 lists.

**Figure 2:**
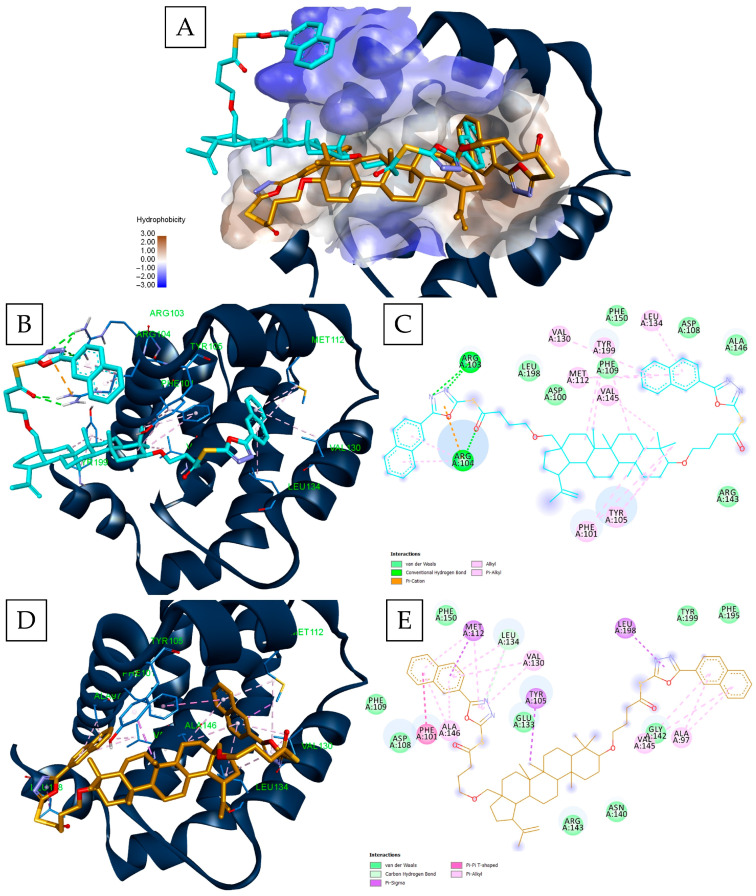
Docking poses of BOxNaf3 in the BH3 binding groove of Bcl-2 (PDB ID: 4LVT); Surface representation of the binding site with both Vina (orange) and Glide (cyan) poses overlaid (**A**); 3D (**B**) and 2D graphical representation (**C**) of the Glide docked pose and 3D (**D**) and 2D graphical representation (**E**) of the Vina docked pose.

Our results showed that BOxNaf3 is the only compound that appeared 5 times across the 6 top-20 lists. Four other compounds made the selection, namely: BOxPhCl1, BOxNaf1, BT1, and BT3. BOxNaf1 outperformed the native ligand (NL) of Mcl-1 (S63845) and Bcl-XL (ABT-737) when ranked according to Vina docking score. The same feat was accomplished by BT1 in the case of Bcl-XL (Table 1). Our docking results also show that Glide favors, overall, top scorers with longer side chains, whereas Vina scored short lateral chain-bearing compounds as top contenders. This discrepancy likely reflects the way the two scoring functions work. GlideScore specifically includes a hydrophobic enclosure term that increases the score for ligands that displace water molecules and fit tightly into hydrophobic pockets. This reward is added on top of van der Waals, hydrogen bonding, and rotatable bond penalties [15,16,17,18]. This is complemented by the structural nature of the BH-3 binding site. The four subpockets (P1-P4) within the BH-3 binding groove are highly hydrophobic [19,20]. Thus, ligands with more hydrophobic bulk can better occupy such sites. On the other hand, Vina’s scoring function is simpler. It includes a hydrophobic and a steric term, but lacks the enclosure hydrophobic packing component, and also penalizes ligand flexibility through an entropy term [21]. Thus, ligands with longer side chains get higher scores after being docked with Glide, whereas Vina’s scoring function does not include this score “boost”. This phenomenon is consistent with a previous case where betulinic acid fatty acid esters containing variable-length side chains were docked into the binding sites of Bcl-2, Bcl-XL, and NF-κB [22]. The docking results showed that Glide scoring was, again, more favorable towards longer-chained BA-esters as opposed to Vina. The difference in ranking is also consistent with the generated docking poses, as well. This can be illustrated by the case of BOxNaf3, which appeared in both top-20 lists of Bcl-2 (PDB-ID:4LVT), but the best docked pose by each software has a slightly different conformation in the binding site (Figure 2). Both poses have one naftyl-substituted oxadiazole occupying the P2 pocket; the Vina pose interacts with the C28-substituted sidechain (Figure 2D,E), whereas the Glide pose interacts with the P2 subpocket through the C3-substituted sidechain (Figure 2B,C). In addition, the conformation of both poses shows major differences towards the P4 region. The Vina pose (Figure 2D) extends its lateral chain along the groove, towards the P4 subpocket, although without establishing strong interactions in that region. On the contrary, the Glide pose (Figure 2B) extends away from P4, forming hydrogen bonds (HB) with residues such as Arg103 and Arg104. In the same Glide pose (Figure 2B,C), the C3-oxadiazole engages a H-bond/cation–π network with Arg103/Arg104, while the triterpenic core makes extensive hydrophobic/π-alkyl contacts with Phe101, Tyr105, Val145 and Phe109; the distal naphthyl ring stacks against Met112, Val130 and Leu134. In the Vina pose (Figure 2D,E), the C28 chain occupies P2 and interacts with Tyr105 through the triterpenic core, with additional hydrophobic contacts formed by the naphthyl-oxadiazole system with Ala97, Gly142, Val145 and Leu198. The multiple terminal aryl hydrophobic contacts on the C3 side (Phe101, Met112, Val130, Leu134, Ala146), strongly stabilize the ligand towards the P4 site. This example illustrates how different scoring functions may emphasize either hydrophobic pocket occupancy (Vina) or polar contacts (Glide), leading to divergent binding modes.

**Figure 3:**
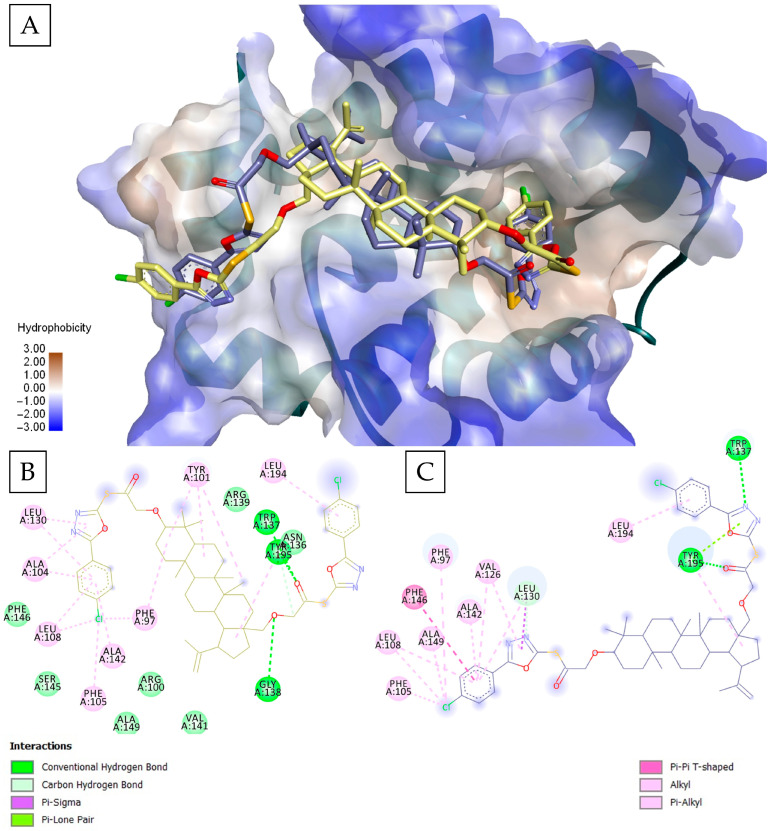
Best docking poses of BOxPhCl1 overlayed in Bcl-XL (PDB: 2YXJ), as predicted by Glide (yellow) and Vina (violet) (**A**). Both programs placed the chlorophenyl-oxadiazole group deep in the P2 pocket, while the betulin core aligned in a nearly identical orientation along the hydrophobic groove; 2D interaction diagram of BOxPhCl1 docked by Glide (**B**) and Vina (**C**) showing nearly identical interaction patterns.

On a different note, there were also cases where the results of the two docking approaches were in strong agreement. Compound BOxPhCl1 was ranked among the top compounds for Bcl-XL by both Vina and Glide, and the best poses predicted by the two docking engines were highly similar in orientation (Figure 3A). Both programs positioned the chlorophenyl-triazole moiety deep within the P2 pocket, while the triterpenic scaffold aligned in a nearly identical manner along the hydrophobic groove. In both poses, the P2-acomodated chlorophenyl-triazole makes π–π/π-alkyl contacts with aromatic/hydrophobic residues lining the pocket, most notably Phe105 (with contributions from Phe97, Leu108, Leu130 and Ala142) (Figure 3B,C). The polar edge of the P2/P4 boundary is contacted in the same region by both engines. Both poses form HBs with Tyr195 through the same carbonyl; both poses form HBs with Trp137, this time through different atoms and in both cases, the chlorophenyl (from the C28 end) is anchored by Leu194. The strong uniformity in both ranking, binding pose prediction, and formed contacts supports the robustness of BOxPhCl1 as a candidate and showcases the benefit of cross-validation across docking software.

These two very different cases show why our selection criteria required compounds to frequently show up on multiple target-generated ranking lists after being docked by multiple scoring functions. Candidates like BOxPhCl1, which show both cross-engine ranking and pose agreement, are very interesting. On the other hand, different cases like BOxNaf3 show that more validation is needed through MD simulations.

An explicit pre-MD ligand-strain screen was deemed unnecessary for interpreting the results presented here because, in this case, docking was only used for rank-based triage across engines, and pose plausibility was confirmed by a brief pre-production MD relaxation (low bound-ligand RMSD and maintenance of BH3-groove contacts) before production MD.

### 2.2. Single-Structure MM-GBSA on Docked Poses

We calculated Prime MM-GBSA binding energies on the 15 docked complexes (local 5 Å shell minimization; OPLS4/VSGB; five ligands evaluated in each target using Glide SP poses) in addition to docking-score ranking. Table 2 provides a summary of the per-complex ΔG_bind values. In accordance with the hydrophobic BH3 groove, vdW and lipophilic terms predominate, with modest Coulomb contributions except in the presence of a polar contact, and are partially offset by GB desolvation. The most favorable ΔG_bind values by target were: Bcl-xL (2YXJ)—BOxNaf3 (−102.3 kcal·mol^−1^), followed by BOxNaf1 (−100.1 kcal·mol^−1^); Bcl-2 (4LVT)—BOxNaf1 (−73.0 kcal·mol^−1^); Mcl-1 (5LOF)—BOxPhCl1 (−86.0 kcal·mol^−1^). For these leads, component analysis shows that there is a lot of vdW/lipophilic stabilization (for example, BOxNaf3 in Bcl-xL: vdW ≈ −87, Lipo ≈ −41), which is opposed by positive Solv_GB (for example, +25), and, if applicable, favorable Coulomb terms (for example, BOxNaf1 in Bcl-2: Coulomb ≈ −20). Generally, the ligand-strain energies were not too high (usually less than 15 kcal·mol^−1^; one higher-strain outlier in Mcl-1), which supported chemically reasonable poses. The MM-GBSA trends support the docking-derived prioritization (BOxNaf1/BOxNaf3/BOxPhCl1) and give an independent estimate of how well the shortlisted candidates bind to each other.

Since ΔG_bind changes with size, we also looked at ligand efficiency (LE = ΔG_bind/heavy-atom count) across targets (Table 2). Two chemotypes consistently exhibited high efficiency: BOxPhCl1 (with the most favorable LE overall, approximately −1.2 kcal·mol^−1^/HA on average) and BOxNaf1 (slightly behind, approximately∆ −1.18 kcal·mol^−1^/HA). These profiles indicate that both ligands convert atoms to binding energy unusually well in Bcl-xL/Mcl-1, with BOxNaf1 also being efficient in Bcl-2. On the other hand, bigger candidates, like BT3, have a reasonable ΔG_bind but a weaker LE, which suggests that their affinity is more size-based. Putting BOxPhCl1 and BOxNaf1 first strikes a balance between absolute ΔG_bind and size-normalized performance, which is helpful for synthesis and optimization.

### 2.3. Molecular Dynamics Simulation

To test protein-ligand complex stability, 100 ns MD simulation runs were recorded for 15 protein-ligand complexes (5 ligands × 3 protein targets). The results are discussed for each protein target.

**Figure 4:**
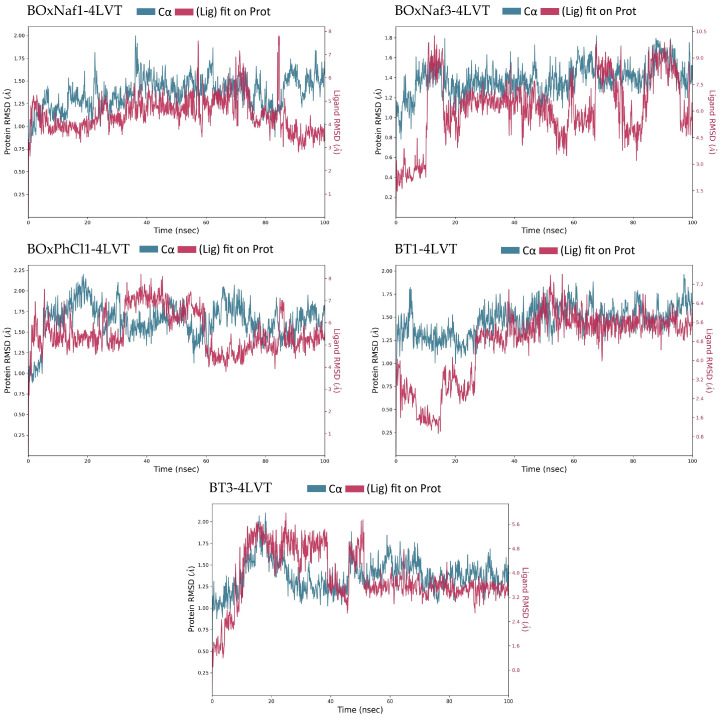
Protein–ligand RMSD trajectories of the five complexes (BoxNaf1–4LVT, BoxNaf3–4LVT, BoxPhCl1–4LVT, BT1–4LVT, and BT3–4LVT) over 100 ns MD simulations. The left *Y*-axis corresponds to protein Cα RMSD (blue), while the right *Y*-axis corresponds to ligand RMSD relative to the protein (red).

**Figure 5:**
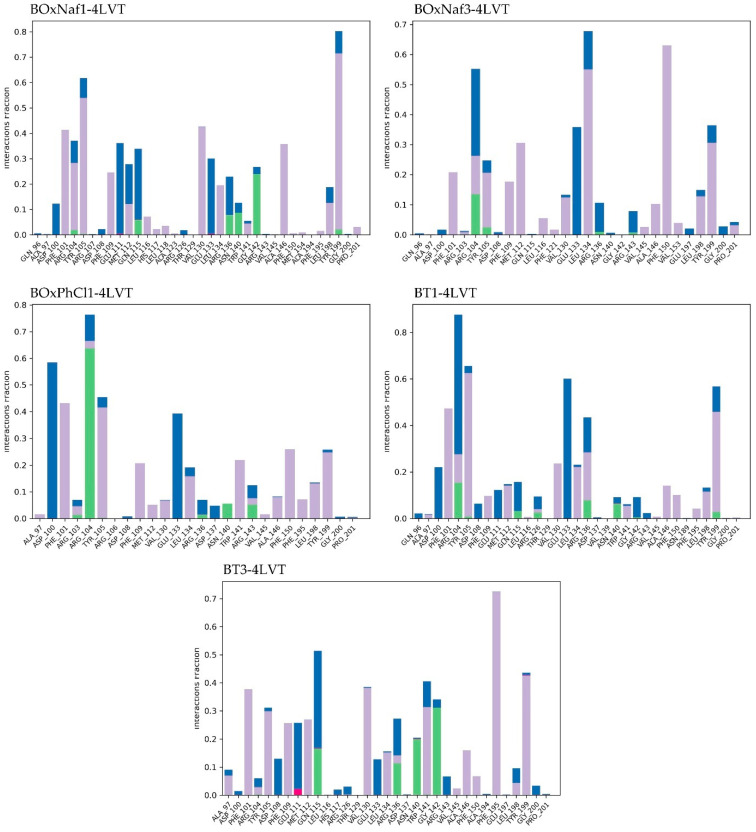
Protein–ligand interaction fractions during 100 ns MD simulations for five complexes (BoxNaf1–4LVT, BoxNaf3–4LVT, BoxPhCl1–4LVT, BT1–4LVT, and BT3–4LVT). Bars represent the fraction of simulation time during which specific residues maintained interactions with the ligand. Green: hydrogen bonds; purple: hydrophobic contacts; pink: ionic interactions; blue: water bridges.

In the case of Bcl-2 (PDB ID: 4LVT), RMSD trajectories were used to extract the Ligand RMSD and Protein RMSD (Cα) (Figure 4). After equilibration (~10 ns onward), the stable interval for each ligand was determined, and the fluctuations were reported as ranges. Each trajectory’s stability was qualitatively classified as varying degrees of fluctuation or stability. The contact histograms were used to quantify protein–ligand interactions (Figure 5). For each ligand, the average number of water bridges, hydrophobic contacts, and hydrogen bonds with persistence >0.1 was extracted. The relative stability and binding patterns of each ligand could be directly compared by analyzing these metrics. All these mentioned values are depicted in Table 3. Mechanistically, the ligands contact the polar rim close to Asn140–Gly142–Arg143, the triterpenic core packs along the hydrophobic channel (Phe101, Tyr105, Val145, Leu134), while the engagement with the BH3 groove happens through one heterocycle which is seated in P2.

BoxNaf1 was the best candidate among the five ligands, as it displayed the most stable binding mode, characterized by multiple persistent contacts and a relatively narrow ligand RMSD range. In particular, BOxNaf1 maintains H-bond/water-bridge interactions with Asn140 and Gly142 (each ~0.2–0.3, with intermittent Arg143 ~0.1–0.2) and ~9 hydrophobic contacts, consistent with sustained P2 anchoring. Despite slightly more variable protein RMSD, BT3 also displayed stable ligand RMSD, suggesting comparatively constant binding. BT3 provides consistent but less densely networked retention in P2 by relying on the canonical rim pair Asn140/Gly142 (0.1–0.2 each) in conjunction with approximately 8 hydrophobics. Similarly to BT1, which also fluctuated significantly, BoxNaf3 showed the highest ligand RMSD fluctuations (~6 Å range), indicating its unstable binding mode. Only a weaker H-bond to Asn140/Gly142 (~0.1–0.15) and fewer hydrophobics are seen for BOxNaf3, which is consistent with higher pose mobility. BoxPhCl1 exhibited moderate ligand fluctuation but a stable protein RMSD, occupying an intermediate conformational profile. With ~7 hydrophobics, BoxPhCl1 forms low-to-moderate occupancy contacts to Asn140 (~0.1–0.15) and Gly142 (~0.1); its intermediate stability is explained by the balance between hydrophobic packing and modest polar anchoring. Generally, BoxNaf1 and BT3 are the most stable binders, while BoxNaf3 and BT1 are the least stable, according to the RMSD trajectories (Figure 4) and contact histograms (Figure 5). Overall, the most stable binding in 4LVT and the lowest ligand RMSD are correlated with the Asn140–Gly142(–Arg143) rim network’s persistence in P2/P4 along with its dense hydrophobic enclosure.

**Figure 6:**
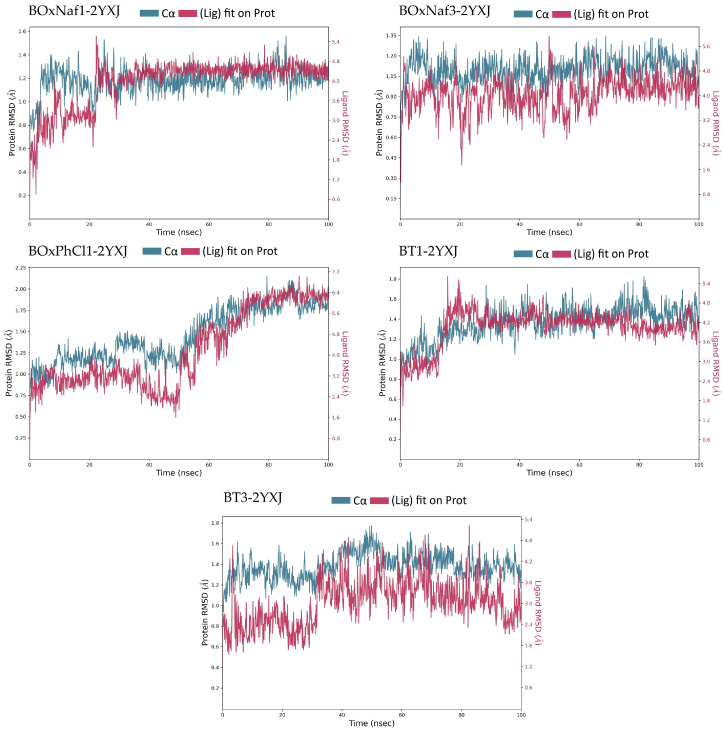
Protein–ligand RMSD trajectories of the five complexes (BoxNaf1–2YXJ, BoxNaf3–2YXJ, BoxPhCl1–2YXJ, BT1–2YXJ, and BT3–2YXJ) over 100 ns MD simulations. The left *Y*-axis corresponds to protein Cα RMSD (blue), while the right *Y*-axis corresponds to ligand RMSD relative to the protein (red).

**Figure 7:**
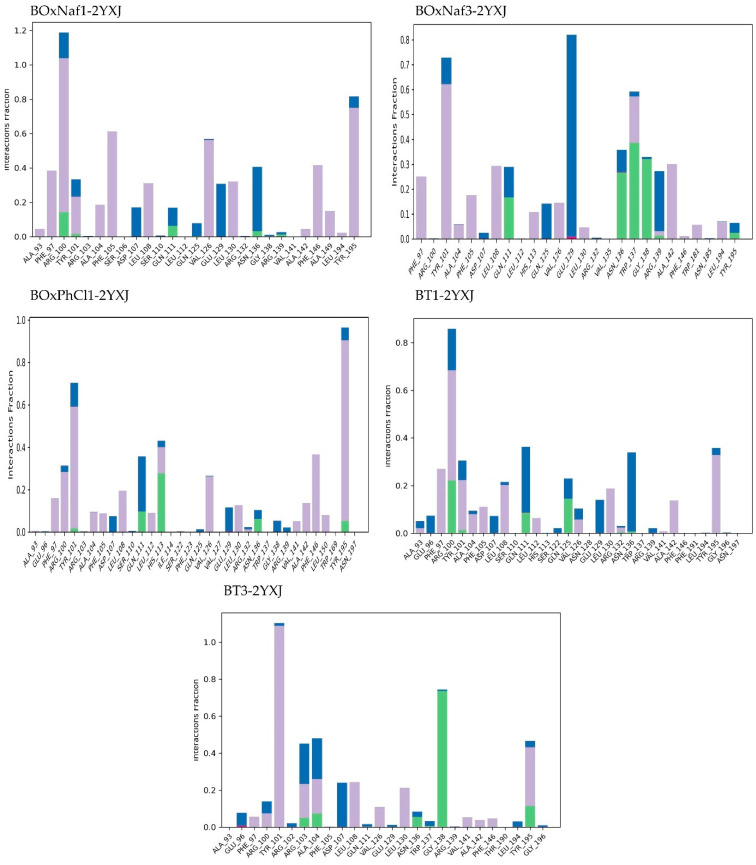
Protein–ligand interaction fractions during 100 ns MD simulations for five complexes (BoxNaf1–2YXJ, BoxNaf3–2YXJ, BoxPhCl1–2YXJ, BT1–2YXJ, and BT3–2YXJ). Bars represent the fraction of simulation time during which specific residues maintained interactions with the ligand. Green: hydrogen bonds; purple: hydrophobic contacts; pink: ionic interactions; blue: water bridges.

The same method was used for the characterization of the ligand-protein complexes of Bcl-XL (PDB ID: 2YXJ) with the 5 ligands. The RMSD values and formed interactions extracted from the RMSD trajectories (Figure 6) and protein-ligand contact histograms (Figure 7) are presented in Table 4. The protein RMSD stayed largely constant (<2 Å) across the five complexes, suggesting that Bcl-XL retained its structural integrity all along. Thus, ligand behaviour and interaction patterns played a major role in determining differences in stability (Figure 6 and Figure 7). BOxNaf1 and BT3 were the most stable ligands. As far as the binding mechanism goes, with the help of numerous hydrophobic contacts (~11 residues) and an intermittent Arg100 H-bond (~0.1 occupancy), BOxNaf1 exhibits a narrow protein RMSD (~1.0–1.3 Å) and a ligand RMSD that stabilizes after ~20 ns (~4–5 Å). Although BOxNaf3 maintains a similar hydrophobic footprint, it exhibits somewhat higher ligand mobility (~3.2–5.5 Å) and adds a multi-anchor polar network—Gln111, Asn136, Trp137, and Gly138 (each ~0.1–0.4) with ~6 water bridges. BOxPhCl1 engages His113 persistently (~0.3–0.4) with ~10 hydrophobics and ~3 water bridges; despite good early packing, the ligand shows a late upward RMSD drift (>80 ns), indicating a slow re-orientation. Despite interacting with fewer hydrophobic residues, BT3 was stabilized by a strong hydrogen bond with Gly138 that successfully anchored the ligand. With a moderate number of persistent contacts and ligand RMSD stabilizing later in the simulation, BT1 displayed intermediate stability. BT1 has about 8 hydrophobic interactions and moderate polar contacts (Arg100 ~0.2–0.3; Gln125 ~0.1–0.2). The ligand RMSD settles after about 20 ns (~3.4–5.0 Å). Phe105/Leu130/Leu194 and nearby hydrophobic interactions pack the triterpenic core along the BH3 groove, keeping the aryl-heterocycle buried in P2 across ligands. This enclosure is combined with a minimum of one rim polar contact (Arg/Gln/His) and sporadic water bridges to form stable complexes. BOxNaf1 and BT3 exhibit the best balance between stability and interaction persistence.

**Figure 8:**
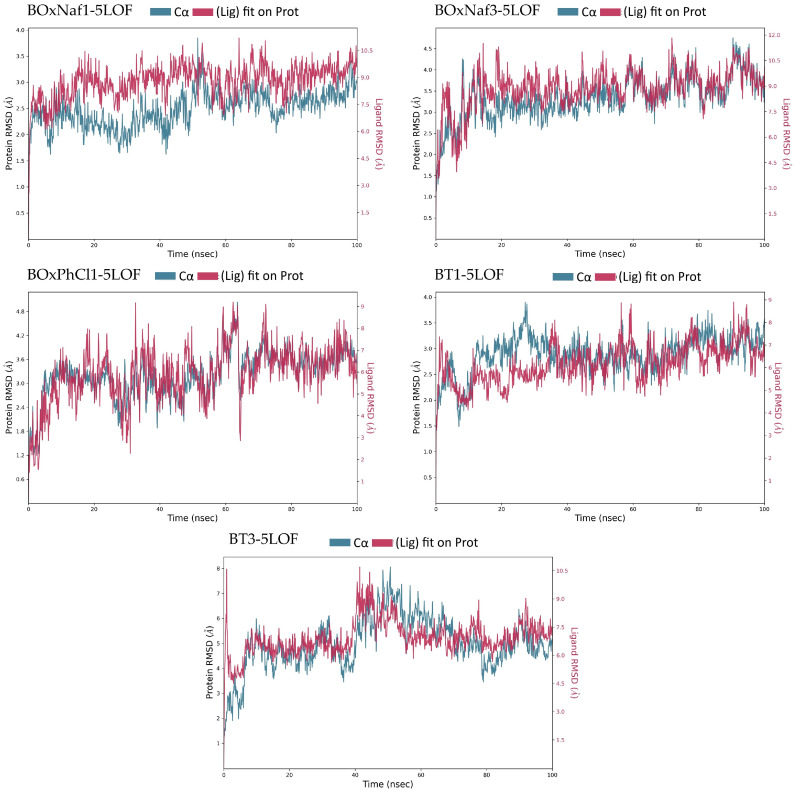
Protein–ligand RMSD trajectories of the five complexes (BoxNaf1–5LOF, BoxNaf3–5LOF, BoxPhCl1–5LOF, BT1–5LOF, and BT3–5LOF) over 100 ns MD simulations. The left *Y*-axis corresponds to protein Cα RMSD (blue), while the right *Y*-axis corresponds to ligand RMSD relative to the protein (red).

**Figure 9:**
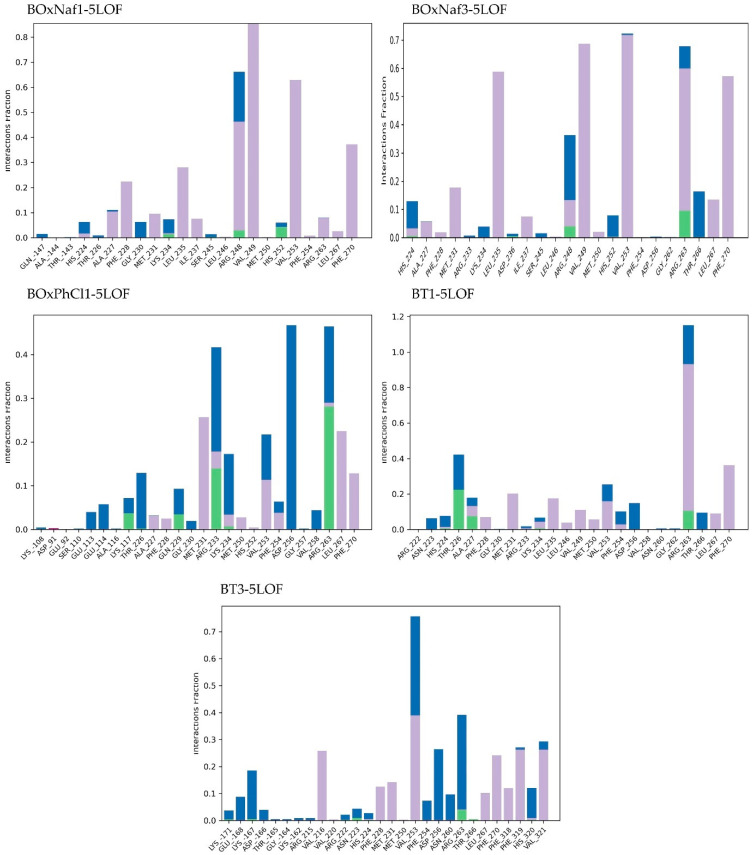
Protein–ligand interaction fractions during 100 ns MD simulations for five complexes BoxNaf1–5LOF, BoxNaf3–5LOF, BoxPhCl1–5LOF, BT1–5LOF, and BT3–5LOF). Bars represent the fraction of simulation time during which specific residues maintained interactions with the ligand. Green: hydrogen bonds; purple: hydrophobic contacts; pink: ionic interactions; blue: water bridges.

Lastly, in the case of Mcl-1 (PDB ID: 5LOF) the stability of all five ligand complexes was analyzed through the same method. Table 5 highlights the MD-derived stability parameters for the five ligand complexes bound to the target protein. With the exception of BT3, which caused noticeable oscillations up to ~7.5 Å, the protein stayed relatively stable (RMSD ~2–4 Å) across the five 5LOF complexes. Mechanistically, the ligands bind with one heterocycle buried in the BH3 P2 region while the triterpenic core spans the groove toward P4. Polar anchoring along the basic rim (particularly Arg263/Arg233/Thr226) and hydrophobic enclosure from pocket residues favor retention. Ligand stability differed more: BT1 was relatively more stable (~5.5–8.0 Å), whereas BOxNaf1 and BOxNaf3 displayed higher RMSD ranges (~7.5–10.5 Å), indicating mobility within the pocket. BOxNaf3 maintains a mixed network—Arg263 H-bond (0.1–0.2), ~8 hydrophobics and ~3 water bridges—supporting P2/P4 occupancy despite elevated RMSD. The most “mobile” were BOxPhCl1 and BT3, which fluctuated widely (up to 9.0–9.5 Å) (Figure 8). However, BOxPhCl1 exhibits dual arginine anchoring (Arg233 0.1–0.2; Arg263 0.2–0.3) along with about four hydrophobic interactions and about six water bridges, which is consistent with temporary reorientations as opposed to complete disengagement. Together with moderate water-bridge networks, BOxNaf3, BOxPhCl1, and BT1 established long-lasting H-bonds with important residues (such as Arg263, Thr226), which served to anchor them. While BT3 maximized hydrophobic interactions (~9 residues) but lacked stable H-bonds, which is correlated with its poor RMSD profile, BT1 combines Thr226 (0.2–0.3) and Arg263 (~0.1) contacts with ~6 hydrophobics and ~4 water bridges, yielding the most restrained ligand RMSD among the set. BOxNaf1 relied primarily on hydrophobic contacts (~7 residues) with minimal polar support (Figure 9). Because of their combined polar and hydrophobic stabilization, BT1, BOxNaf3, and BOxPhCl1 seem to be the more dependable binders overall, while BT3 was found to be the least stable ligand. In general, dense hydrophobic packing in P2/P4 and persistent engagement of Arg263/Arg233/Thr226 at the rim, enhanced by water bridges, correlate with better pose retention in Mcl-1, while purely hydrophobic binding (e.g., BT3) is linked to higher ligand mobility.

**Figure 10:**
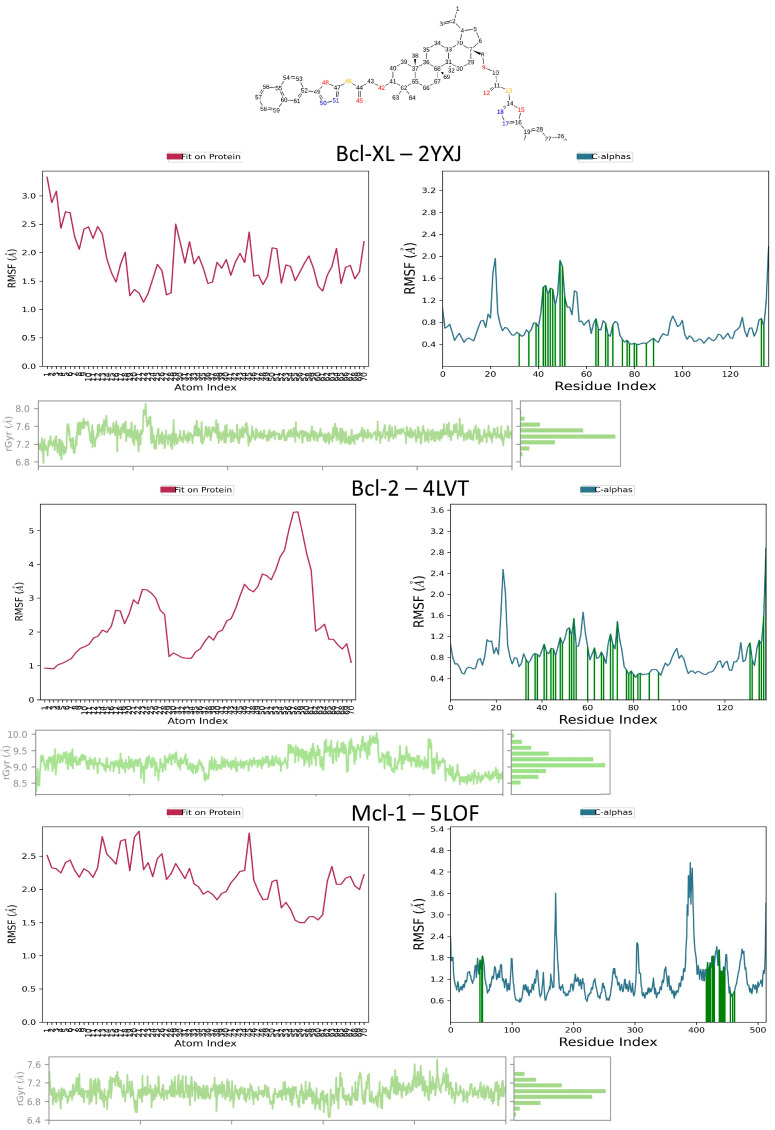
BOxNaf1 flexibility and compactness across Bcl-xL (2YXJ), Bcl-2 (4LVT), and Mcl-1 (5LOF). **Top**: 2D structure of BOxNaf1 with atom indices. For each target (rows): **left**, ligand heavy-atom RMSF (frames aligned on protein backbone); **right**, protein Cα RMSF per residue, (green bars mark contact residues); **bottom** of each row, ligand radius of gyration (rGyr) time series with distribution inset.

Regarding ligand RMSF and compactness Protein Cα RMSF stays low in the BH3 groove (flexibility confined to surface loops) across Bcl-xL (2YXJ), Bcl-2 (4LVT), and Mcl-1 (5LOF), suggesting a rigid binding channel (Figure 10). In accordance with a packed core plus flexible P2/P4 substituents, ligand RMSF is highest at terminal substituents and lowest on the triterpenic core. A compact bound ensemble is supported by ligand rGyr’s stability (no upward drift) in all three systems; only Mcl-1 exhibits somewhat wider fluctuations at the distal groups without persistent expansion.

For BoxNaf3 protein Cα RMSF profiles across Bcl-xL/2YXJ, Bcl-2/4LVT, and Mcl-1/5LOF reveal a stiff BH3 groove with flexibility limited to solvent-exposed loops; ligand-contacted bands align with low RMSF in the pocket. The ligand RMSF pattern is conserved: the distal naphthyl/heterocycle substituents that adjust to P2/P4 micro-motions exhibit greater fluctuations, whereas the betulin core is the least mobile. In all three systems, ligand rGyr is essentially constant (small, non-directional oscillations), suggesting compact binding as opposed to progressive extension; Mcl-1 exhibits the widest rGyr/RMSF variability, which is in line with its higher pocket plasticity, but without persistent expansion (Figure S1).

In the case of BoxPhCl1 the proteins’ low RMSF bands correspond to contact-marked residues in the pocket, suggesting stable packing around the ligand. Cα RMSF traces reveal a rigid BH3 groove with flexibility restricted to exposed loop segments. In accordance with a packed core and flexible P2/P4 termini, the ligand RMSF is lowest on the betulin core and increases on the chlorophenyl–triazole substituents. Compact binding is supported by the ligand radius of gyration (rGyr) remaining roughly constant in all three systems (small, non-directional oscillations); Mcl-1 exhibits the widest local RMSF/rGyr fluctuations, but without persistent expansion (Figure S2).

Consistent with persistent packing around BT1, the protein Cα RMSF traces across Bcl-xL/2YXJ, Bcl-2/4LVT, and Mcl-1/5LOF reveal a rigid BH3 groove with flexibility mostly limited to loop regions away from the pocket. All three proteins exhibit the same pattern, with ligand RMSF being highest toward the terminal heterocycles/aryl groups that adapt within P2/P4 and lowest on the betulin core. Each target’s ligand radius of gyration (rGyr) is roughly constant (small, non-directional oscillations), suggesting compact binding as opposed to progressive extension; Mcl-1 shows the widest local fluctuations but no persistent rGyr drift (Figure S3).

A rigid BH3 groove with mobility primarily in solvent-exposed loops is indicated by the protein Cα RMSF profiles with respect to BT3 compactness across Bcl-xL/2YXJ, Bcl-2/4LVT, and Mcl-1/5LOF; contact-band regions surrounding the pocket exhibit low RMSF, which is consistent with persistent packing. The ligand RMSF pattern is conserved: the terminal aryl/heterocycle substituents that adapt within P2/P4 exhibit greater fluctuations, while the betulin core is least mobile. There is no persistent rGyr drift, and ligand rGyr is roughly constant overall, indicating a compact bound ensemble, with the widest local fluctuations in Mcl-1, consistent with its higher pocket plasticity (Figure S4).

We compared the MD-derived stability metrics of all five compounds across the three targets in order to determine which triple inhibitors (ligands active against Bcl-2, Bcl-XL, and Mcl-1) were the most promising. Each protein–ligand complex’s post-equilibration RMSD behaviour and the durability of important contacts (hydrogen bonds, hydrophobic contacts, and water bridges) were used to assess stability. Interestingly, our findings show that ligands with several persistent interactions, particularly hydrophobic contacts and H-bonds, generally show fewer RMSD fluctuations, indicating more reliable binding [23]. This observation is in line with previously reported MD studies on BH3 groove inhibitors, which found that stable complexes are characterized by extensive hydrophobic contacts complemented by hydrogen bonds [23]. In our case, stability rankings were primarily determined by differences in ligand RMSD because protein Cα RMSD remained relatively restrained in the MD simulations (<~2 Å for Bcl-XL, ~2–4 Å for Mcl-1, and ~1–2 Å for Bcl-2 after equilibration).

Our results show that no single ligand was completely stable across Bcl-2, Bcl-XL, and Mcl-1 when all three targets were taken into consideration; however, two compounds, BoxNaf1 and BT3, stand out as being the most similar to the ideal profile. In two of the three proteins (Bcl-2 and Bcl-XL), both of these demonstrated strong stability, and in the third, they at least partially remained present. Despite its relatively dynamic complex with Mcl-1, BoxNaf1 in particular demonstrated a consistently narrow ligand RMSD and multiple contacts in 4LVT and 2YXJ, all the while engaging Mcl-1 via hydrophobic interactions. Similarly, BT3 occupied the pocket via hydrophobic interactions for a significant amount of the simulation, despite having poor Mcl-1 binding. The same ligand remained stably bound to Bcl-2 and Bcl-xL for the full 100 ns simulation. On the other hand, each of the other three ligands exhibited a critical weakness on either Bcl-2 or Bcl-XL, which would probably prevent them from exhibiting true pan-Bcl-2-family inhibition.

Our search for broad-spectrum Bcl-2 family inhibitors (including Bcl-2, Bcl-XL, and Mcl-1) is in line with current advances in anticancer medication development, particularly those that use natural or semi-synthetic triterpenoids. Triterpene-based compounds have been shown in some investigations to bind promiscuously to anti-apoptotic Bcl-2 proteins. The pentacyclic triterpenes ursolic acid and oleanolic acid were found to be promising multi-target inhibitors of Bcl-2, Bcl-XL, and Mcl-1 through a computational screening of Morinda lucida extracts [11]. Importantly, these compounds met the drug-likeness requirements and demonstrated greater docking affinities to the BH3 grooves than even well-known synthetic inhibitors. Our Bet derivative series shares a triterpenoid scaffold with ursolic acid and oleanolic acid, and their predicted broad activity supports the notion that a single triterpenoid can interact with several Bcl-2 family pockets.

Considering the aforementioned, BoxNaf1 and BOxPhCl1 are particularly noteworthy as multi-target inhibitors of the Bcl-2 family. BoxNaf1 combines strong MD stability in Bcl-2/Bcl-xL with favorable MM-GBSA across targets, while BOxPhCl1 shows reproducible poses across docking engines (pose overlay in Bcl-xL) and consistently favorable MM-GBSA in Bcl-xL and Mcl-1, with acceptable performance in Bcl-2. BT3, although MD-stable in Bcl-2/Bcl-xL, displayed weaker MM-GBSA energetics and is therefore retained as a lower-priority backup chemotype. In spite of the need for optimization on the third target, BOxNaf1 and BOxPhCl1 merit synthesis and biological testing as dual/multi-target leads based on their cross-target performance and interaction profiles.

## 3. Materials and Methods

### 3.1. Ligand Design and Compound Library Construction

All compounds were sketched using Biovia Draw, Version 19.1 (Dassault Systèmes Biovia Corp, San Diego, CA, USA) and exported as other required formats for the following steps. Guided by our ligand design rationale, we used the scaffold of betulin (Bet) to accommodate the central hydrophobic groove of the BH3 domain, while attaching to heterocycles via variable-length spacers (2–10 C atoms), to occupy the P2 and P4 subpockets. The heterocycles consisting of 1,2,4-triazoles, 1,3,5-oxadiazoles or 1,2,3-benzotriazoles were selected to facilitate H-bond and π interaction formation with groove neighboring residues.

**Figure 11:**
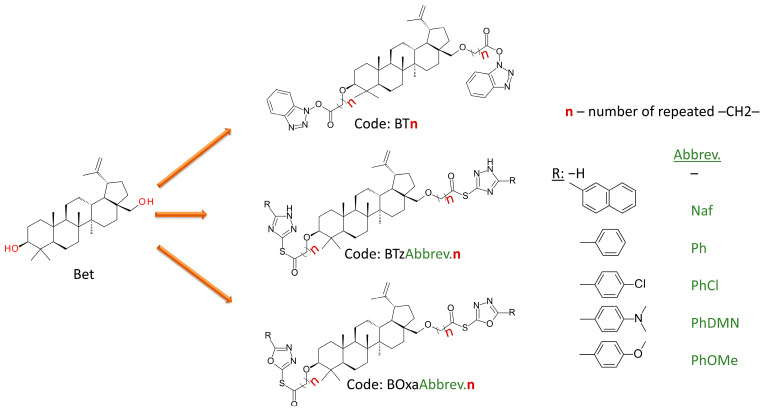
General chemical structure of the betulin-derived ligands. All compounds are based on the Bet scaffold, with heterocyclic moieties (substituted triazoles or oxadiazoles) linked at the C-3 and C-28 positions via a variable-length carbon spacer (*n* = 2–10). Compound codes begin with “B”, followed by a heterocycle identifier (T = benzotriazole, Tz = triazole, Oxa = oxadiazole), an abbreviation for the substituent on the heterocycle (as shown in the key), and a terminal number indicating the number of methylene units in the spacer.

Each compound is a derivative of Bet that contains the heterocyclic structures (linked at the OH groups from the 3rd and 28th through a variable-length spacer fragment (2–10 C atoms). The linker is bound to Bet via an ether bond and to the heterocycle through an ester bond. Each compound has a unique code. All code names start with “B” followed by letters denoting the heterocycle type, a specific abbreviation for a given radical substituted on the heterocycle (if the case), and a number that indicates how many methylene groups are found in the spacer molecule. The general chemical structure of the constructed ligands and information about their code identity are depicted in Figure 11. Smile strings for all constructed compounds are available in the Supplementary Files (Table S1).

### 3.2. Molecular Docking

#### 3.2.1. Docking with PyRx—Vina

The following docking protocol is a slightly modified variation in a previous reported method [24,25,26]. Protein targets 3D structures were obtained from the RCSB Protein Data Bank [27]. The inclusion criteria for protein 3D structures were: (i) resolution 1.5—2.5 Å; (ii) R-Free ≤ 0.25 and R-Work ≤ 0.20; (iii) structures with a complete binding site, without missing amino acid residues; (iv) the presence of a co-crystalized ligand that has at least pre-clinical relevance, meaning that it was previously reported as being in vivo effective or it was advanced to preclinical development. The chosen 3D structure files were 2YXJ for Bcl-XL (co-crystalized ligand: ABT-737), 4LVT for Bcl-2 (co-crystalized ligand: navitoclax), and 5LOF for Mcl-1 (co-crystalized ligand: S63845). The 3 protein targets were converted to PDBQT files, using PyRx’s v 0.8 (The Scripps Research Institute, La Jolla, CA, USA) automated feature “Make Macromolecule” [28].

The constructed Bet derivatives, previously saved as mol files, were converted into PDBQT 3D structure files using PyRx’s embedded Open Babel feature, by employing the uff force field (hydrogen addition/protonation and energy minimization with UFF; default settings). PyRx incorporates the AutoDock Vina docking engine, which allows users to adjust docking parameters while providing a graphical user interface for setting up, running, and visualizing docking experiments. We used this feature to perform molecular docking [28,29]. The grid box, which defines the search space, was adjusted to fit the site around the co-crystalized ligand. For every candidate pose, Vina conducts a local Broyden–Fletcher–Goldfarb–Shanno (BFGS) minimization and automatically performs flexible-ligand torsional sampling; no external conformer enumeration was employed in this step. The number of returned poses may be less than the nominal num_modes since Vina reports up to the maximum number of unique poses within the default energy window and RMSD clustering. Receptor grids were defined from the native ligands (Bcl-xL 2YXJ/ABT-737; Bcl-2 4LVT/Navitoclax; Mcl-1 5LOF/S63845), and self-docking reproduced the crystallographic poses; all-atom RMSD values did not exceed 2.0 Å, a widely accepted margin for the optimal reproduction of binding modes [30,31]. A short pre-production MD relaxation confirmed that the selected poses retained their binding geometry before entering production MD, supporting the realism of the starting conformations. Bet and BA were docked as well to serve as secondary positive controls. Ligand-protein binding interactions analysis and image generation were achieved using Discovery Studio Visualizer v20.1.0.19295 (Dassault Systèmes Biovia Corp, San Diego, CA, USA).

#### 3.2.2. Docking with Schrödinger—Glide

All the above-mentioned compounds were re-docked into the same targets using Schrödinger’s Glide module (Schrödinger Release 2022-4, Schrödinger, LLC, New York, NY, USA) [15,16,17]. For this purpose, a text file containing SMILE string descriptors for all constructed ligands was submitted to Maestro’s LigPrep for ligand preparation using the default features. The above-mentioned 3 protein targets (Bcl-XL—2YXJ, Bcl-2—4LVT, Mcl-1—5LOF) were prepared using Maestro’s Protein Preparation Wizard using default parameters, including multiple bond rectification, hydrogen atom (H) addition, non-polar H merging, and water molecule removal beyond a 5 Å radius from the co-crystalized ligand [32]. The wizard also performed a protein structure processing step to identify and correct errors, as well as a protein-ligand complex refinement using successive restrained minimization with the featured OPLS_2005 force field. Maestro’s receptor grid generation feature was used to generate the grid box for docking space delimitation, using the native ligand as the central point to define the active site, and then adjusting the grid box to better fit the active site. Receptor grids were centered on the co-crystallized ligands (Bcl-xL 2YXJ/ABT-737; Bcl-2 4LVT/ABT-263/Navitoclax; Mcl-1 5LOF/S63845) and self-docking reproduced the crystallographic poses (all-atom RMSD ≤ 2.0 Å). These native ligands were used as internal controls during screening. Image generation and interaction analysis were achieved using the same software mentioned above. Glide’s internal flexible-ligand sampling with post-docking minimization handled conformational variability, and LigPrep supplied minimized 3D protomer/tautomer states instead of external conformer enumeration. To reduce artifacts from high torsional flexibility, we used Glide’s penalty on non-planar amide rotations and verified pose stability by short pre-production MD (the bound-ligand heavy-atom RMSD remained low during the initial relaxation), before proceeding to production MD.

### 3.3. Candidate Selection Criteria: Scoring and Ranking

The AutoDock Vina results, recorded as binding energy values (ΔG, kcal/mol), were used to rank the compounds from most to least active. The same process was used for the Glide docking scores using the Glide r_i_docking_score. For each protein target (Bcl-2, Bcl-Xl, Mcl-1) the top 20 ranked unique compounds were selected for further refinement. In cases where multiple conformations of the same compound scored in the top 20, only the best-scoring conformation was selected. All six lists (3 protein targets × 2 scoring functions) were combined in one table, where the frequency of each compound was counted. A docked compound was considered a selection for further analysis if it appeared at least 4 times across the six top-20 lists, and 3 times in the top-20 list of each of the three protein targets, regardless of the used scoring function. The justification for using 4 as a frequency threshold instead of 3 (the minimum required for covering all three protein targets) was to ensure that each selected compound exhibited a strong theoretical inhibitory potential by appearing in both scoring functions-constructed top-20 lists of one protein target. Observing the same compound performing when scored by different docking software reduces method-specific bias and ensures that the compound might actually be a truly active inhibitor [30,33].

### 3.4. MM-GBSA on Docked Poses

Binding free energies (ΔG_bind) were estimated with Prime MM-GBSA (Schrödinger) on 15 protein–ligand complexes (five ligands per target: Bcl-xL/2YXJ, Bcl-2/4LVT, Mcl-1/5LOF). For each target, ligands were first docked with Glide SP on receptor grids centered on the native ligand; the top pose per ligand (with Glide post-docking minimization enabled) was used as input. Prime calculations employed OPLS4 with the VSGB implicit solvent model. Local minimization was applied to the ligand and protein residues within 5 Å of the ligand; the remainder of the protein was kept fixed. For each complex we report the total ΔG_bind (kcal·mol^−1^) and the standard components (vdW, Coulomb, Lipo, Solv_GB), together with ligand strain and receptor strain energies. Ligand efficiency (LE) was computed as ΔG_bind/N_heavy_atoms to aid size-normalized comparisons.

### 3.5. Molecular Dynamics Simulation

Molecular dynamics (MD) simulations on the binding sites of all three protein targets were performed using the Desmond package from Schrödinger (Schrödinger Release 2022-4, Schrödinger, LLC, New York, NY, USA), with compounds chosen after the docking step. The refined structures were solved for simulations in TIP3P water molecule periodic orthorhombic boxes with Desmond’s System Builder module. A 9 Å cutoff distance was set between the box’s edges and the receptors’ closest atoms. The systems were neutralized through the addition of chloride and sodium ions. The default relaxation and equilibration protocol implemented in Desmond was then used to relax the solvated systems. Each system underwent 100ns MD production runs. The simulations were run with no restraints in the NPT ensemble, with constant temperature (300 K) and pressure (1 atm). Non-bonded interactions were considered up to a cutoff distance of 9 Å. The coordinate recording interval was set to 4.8 picoseconds (ps). The trajectory analyses were carried out using Maestro’s Simulation Integration Diagram tool, within Schrödinger (Schrödinger Release 2022-4, Schrödinger, LLC, New York, NY, USA). The RMSD and RMSF of the receptor backbone atoms in relation to the reference structure were measured, analyzed, and represented for each system.

## 4. Conclusions

In conclusion, two betuline derivatives, BOxNaf1 and BOxPhCl1, were discovered by our combined virtual screening and simulation study, as the most compelling multi-target candidates against Bcl-2, Bcl-xL, and Mcl-1. Both ligands ranked among the top poses across docking engines and maintained compact, packed binding ensembles in the BH3 groove (low protein-pocket RMSF, stable ligand rGyr). BOxPhCl1 also displayed repeatable cross-engine poses. Their potential is further supported by single-structure Prime MM-GBSA, with favorable ΔG_bind components driven by vdW/lipophilic enclosure and complementary polar contacts. BT3 is kept as a lower-priority backup chemotype because it showed MD stability in Bcl-2/Bcl-xL but weaker MM-GBSA energetics. As dual/multi-target BH3-groove inhibitors, BOxNaf1 and BOxPhCl1 are the preferred leads for synthesis and experimental validation overall; further research should involve chemical synthesis and in vitro/in vivo evaluation to confirm efficacy and safety in regard to the Bcl-2 family.

## Figures and Tables

**Table 1:** Top-20 candidate selection for each protein target, according to Vina and Glide docking scores. Compounds that appeared at least 4 times across all six lists and across all three protein targets are highlighted in different colours. The native ligands, ABT-737 for Bcl-XL, Navitoclax for Bcl-2, and S63845 for Mcl-1 (PDB 5LOF) (red text), were redocked in their parent protein targets and ranked alongside the Bet derivatives.

Rank	GLIDE—Bcl-XL (kcal/mol)		VINA—Bcl-XL (kcal/mol)		GLIDE—Bcl-2 (kcal/mol)		VINA—Bcl-2 (kcal/mol)		GLIDE—MCL1 (kcal/mol)		VINA—Mcl-1 (kcal/mol)	
**1**	ABT-737	−9.45	BT2	−11.4	Navitoclax	−12.49	BTzNaf1	−11.7	S63845	−11.40	BOxNaf1	−9.8
**2**	BOxPhCl1	−8.79	BT1	−11.3	BOxPhCl5	−8.22	Navitoclax	−11.4	BTzPhCl4	−7.88	BTzNaf1	−9.1
**3**	BTzPhCl7	−8.72	BOxNaf1	−10.9	BOxPhDMN3	−7.97	BOxNaf3	−10.9	BTzPhDMN1	−7.56	BT4	−8.8
**4**	BT5	−8.70	ABT-737	−10.8	BOxPhCl3	−7.91	BT2	−10.7	BTzNaf8	−7.35	BTzNaf4	−8.8
**5**	BOxPhCl8	−8.56	BOxNaf2	−10.7	BOxPh6	−7.89	BOxNaf1	−10.6	BTzNaf7	−7.35	S63845	−8.8
**6**	BOxPhCl4	−8.48	BOxNaf3	−10.7	BOx9	−7.79	BT1	−10.5	BTzNaf9	−7.15	BOxPhCl1	−8.8
**7**	BTz9	−8.48	BOxPhCl1	−10.7	BTzPhCl5	−7.76	BtzPh1	−10.3	BOxPhCl7	−7.13	BtzPhCl3	−8.7
**8**	BOxNaf5	−8.46	BtzPhCl1	−10.4	BTzPhCl8	−7.63	BtzPh2	−10.3	BOxPhCl3	−7.12	BtzPhOMe1	−8.6
**9**	BOxPhOMe1	−8.17	BOxPh1	−10.4	BTz5	−7.60	BOxNaf4	−10.3	BTzNaf2	−7.05	BOxNaf3	−8.6
**10**	BTzNaf7	−7.98	BT3	−10.3	BOxPh3	−7.57	BTzNaf3	−10.2	BOxNaf6	−7.03	BOxPh2	−8.6
**11**	BOxNaf1	−7.94	BOxPhCl4	−10.3	BTz7	−7.56	BOxNaf2	−10.2	BTzPhCl5	−7.02	BtzPh2	−8.5
**12**	BOxPhDMN7	−7.92	BtzPh2	−10.2	BOxPh2	−7.51	BOxPh1	−10.2	BTzNaf5	−7.02	BT2	−8.4
**13**	BT1	−7.89	BtzPhDMN1	−10.2	BOxNaf3	−7.32	BOxPh2	−10.2	BOxPhOMe9	−7.00	BTzNaf2	−8.4
**14**	BTzPhCl3	−7.87	BT5	−10.1	BOxPhCl7	−7.31	BOxPhCl1	−10.2	BOxPh4	−6.96	BtzPhOMe4	−8.4
**15**	BOxNaf4	−7.81	BtzPhOMe1	−10.1	BT6	−7.30	BT3	−10.1	BOxPh5	−6.94	BT3	−8.3
**16**	BOxPh6	−7.78	BOxPhCl2	−10.1	BT5	−7.23	BT4	−10	BTzPhCl3	−6.90	BTzNaf6	−8.3
**17**	BTzPhOMe7	−7.78	BOxNaf4	−10	BTz4	−6.96	BTzNaf2	−10	BOxNaf7	−6.79	BtzPh1	−8.3
**18**	BTz7	−7.77	BOxNaf5	−9.9	BTz3	−6.95	BtzPhCl1	−10	BOxPhCl6	−6.76	BtzPhCl4	−8.3
**19**	BT3	−7.73	BT8	−9.8	BTzPh9	−6.93	BOxPhCl2	−9.8	BTzPhOMe4	−6.76	BOxNaf2	−8.3
**20**	BOxNaf3	−7.71	BtzPhCl2	−9.8	BOxPhOMe2	−6.89	BOxPhOMe1	−9.8	BTzNaf3	−6.74	BT1	−8.2

**Table 2:** Prime MM-GBSA ΔG_bind (kcal·mol^−1^) and components (vdW, Coulomb, Lipo, Solv_GB), plus ligand/receptor strain, for 15 docked complexes (five ligands per target).

Target	Ligand	G_Bind (kcal/mol)	vdW	Coulomb	Lipo	Solv_GB	Ligand Strain	Receptor Strain	Ligand Efficiency
**Bcl-2 (4LVT)**	BOxNaf1	−73.02	−60.21	−19.98	−29.93	39.12	9.61	14.66	−1.04
**Bcl-2 (4LVT)**	BOxPhCl1	−64.58	−49.68	−39.74	−28.82	49.66	8.77	25.50	−1.01
**Bcl-2 (4LVT)**	BOxNaf3	−62.48	−50.12	−45.20	−20.18	52.52	14.59	17.31	−0.84
**Bcl-2 (4LVT)**	BT3	−52.28	−54.52	−0.88	−19.01	27.81	8.84	23.23	−0.84
**Bcl-2 (4LVT)**	BT1	−40.52	−46.05	−1.89	−16.80	30.93	14.03	19.06	−0.69
**Bcl-xL (2YXJ)**	BOxNaf3	−102.26	−87.38	13.60	−41.26	25.50	6.19	30.22	−1.38
**Bcl-xL (2YXJ)**	BOxNaf1	−100.10	−83.97	1.75	−40.52	17.35	15.35	24.24	−1.43
**Bcl-xL (2YXJ)**	BOxPhCl1	−85.45	−67.68	4.76	−30.86	5.27	5.10	15.88	−1.33
**Bcl-xL (2YXJ)**	BT1	−71.00	−68.80	6.23	−24.51	11.65	4.82	15.22	−1.22
**Bcl-xL (2YXJ)**	BT3	−66.73	−61.75	8.47	−20.98	−0.08	13.01	8.17	−1.07
**Mcl-1 (5LOF)**	BOxPhCl1	−85.94	−69.04	−2.14	−40.39	10.26	9.84	2.41	−1.34
**Mcl-1 (5LOF)**	BOxNaf1	−74.58	−73.31	−20.37	−31.13	27.60	11.30	10.58	−1.06
**Mcl-1 (5LOF)**	BOxNaf3	−72.75	−47.65	−53.88	−23.40	48.51	26.30	13.73	−0.98
**Mcl-1 (5LOF)**	BT3	−70.58	−64.25	−4.39	−29.89	9.26	4.47	11.47	−1.13
**Mcl-1 (5LOF)**	BT1	−67.39	−58.37	−18.76	−28.41	32.45	8.61	22.15	−1.16

**Table 3:** Comparative summary of MD-derived stability parameters for the five ligands bound to protein 4LVT. Protein and ligand RMSD values are reported as stable ranges (Å) measured after equilibration (~10 ns). Interaction profiles are based on hydrogen bonds, hydrophobic contacts, and water bridges with persistence > 10% of the simulation. Qualitative stability notes are included for each trajectory. Data correspond to the RMSD trajectories (Figure 4) and ligand contact histograms (Figure 5).

Ligand	Protein RMSD (Å)	Ligand RMSD (Å)	H-Bonds (≥0.1 frac)	Hydrophobic Contacts (≥0.1 frac)	Water Bridges (≥0.1 frac)
**BoxNaf1**	0.8–2.0 (stable, minor drift)	3.0–5.5 (stable)	Asn140 (0.2–0.3), Gly142 (0.2–0.3), Arg143 (0.1–0.2)	~9 residues	~5 residues
**BoxNaf3**	1.2–1.8 (stable, slight drift)	4.5–10.5 (fluctuates)	Asn140 (0.1–0.15), Gly142 (0.1–0.15)	~8 residues	~4 residues
**BoxPhCl1**	1.0–1.7 (stable)	3.5–8.0 (fluctuates)	Asn140 (0.1–0.15), Gly142 (~0.1)	~7 residues	~3 residues
**BT1**	1.0–1.8 (stable, post 20 ns drift)	4.0–6.5 (post 20 ns drift, fluctuates)	Asn140 (0.2–0.3), Gly142 (0.1–0.2), Arg143 (0.1–0.2)	~10 residues	~6 residues
**BT3**	1.0–2.0 (stable after equilibration)	2.5–5.5 (mild fluctuation)	Asn140 (0.1–0.2), Gly142 (0.1–0.2)	~8 residues	~4 residues

**Table 4:** Comparative summary of MD-derived stability parameters for the five ligands bound to protein 2YXJ. Protein and ligand RMSD values are reported as stable ranges (Å) measured after equilibration (~10 ns). Interaction profiles are based on hydrogen bonds, hydrophobic contacts, and water bridges with persistence >10% of the simulation. Qualitative stability notes are included for each trajectory. Data correspond to the RMSD trajectories (Figure 6) and ligand contact histograms (Figure 7).

Ligand	Protein RMSD (Å)	Ligand RMSD (Å)	H-Bonds (≥0.1 frac)	Hydrophobic Contacts (≥0.1 frac)	Water Bridges (≥0.1 frac)
**BOxNaf1**	~1.0–1.3 (stable)	~3.0–5 (stable after 20 ns: 4.2–5)	Arg100 (0.1–0.1)	~11 residues	~4 residues
**BOxNaf3**	~0.9–1.3 (stable)	~3.2–5.5 (fluctuates)	Gln111 (0.1–0.2), Asn136 (0.2–0.3), Trp137 (0.3–0.4), Gly138 (0.3–0.4)	~8 residues	~6 residues
**BOxPhCl1**	~1.0–2.1 (late upward drift)	~2–6.5 (highly fluctuates)	His113 (0.3–0.4)	~10 residues	~3 residues
**BT1**	~1.0–1.8 (stable)	~3.4–5.0 (fluctuates; stabilizes after 20 ns)	Arg100 (0.2–0.3), Gln125 (0.1–0.2)	~8 residues	~4 residues
**BT3**	~1.1–1.6 (stable)	~2.0–4.0 (fluctuates)	Gly138 (0.7–0.8)	~6 residues	~3 residues

**Table 5:** Comparative summary of MD-derived stability parameters for the five ligands bound to protein 5LOF. Protein and ligand RMSD values are reported as stable ranges (Å) measured after equilibration (~10 ns). Interaction profiles are based on hydrogen bonds, hydrophobic contacts, and water bridges with persistence >10% of the simulation. Qualitative stability notes are included for each trajectory. Data correspond to the RMSD trajectories (Figure 8) and ligand contact histograms (Figure 9).

Ligand	Protein RMSD (Å)	Ligand RMSD (Å)	H-Bonds (≥0.1 frac)	Hydrophobic Contacts (≥0.1 frac)	Water Bridges (≥0.1 frac)
**BOxNaf1**	~1.5–3.5 (stable)	~7.5–10.5 (fluctuates)	-	~7 residues	~1 residue
**BOxNaf3**	~2.5–4.5 (stable)	~7.5–10.5 (fluctuates)	Arg263 (0.1–0.2)	~8 residues	~3 residues
**BOxPhCl1**	~2.0–4.0 (minor fluct.)	~3.0–9.0 (highly fluctuates)	Arg233 (0.1–0.2), Arg263 (0.2–0.3)	~4 residues	~6 residues
**BT1**	~2.5–3.5 (stable)	~5.5–8.0 (moderately fluctuates)	Thr226 (0.2–0.3), Arg263 (~0.1)	~6 residues	~4 residues
**BT3**	~2.0–7.5 (highly fluctuates)	~6.0–9.5 (highly fluctuates)	-	~9 residues	~4 residues

## Data Availability

The original contributions presented in the study are included in the article; further inquiries can be directed to the corresponding author.

## Supplementary Materials

The following supporting information can be downloaded at: https://www.mdpi.com/article/10.3390/molecules30193919/s1, Table S1. SMILE strings of the constructed Bet derivatives; Table S2. Vina docking scores of docked ligands against Bcl-XL (2YXJ); Table S3. Vina docking scores of docked ligands against Bcl-2 (4LVT); Table S4. Vina docking scores of docked ligands against Mcl-1 (5LOF); Table S5. Glide docking scores of docked ligands against Bcl-XL (2YXJ); Table S6. Glide docking scores of docked ligands against Bcl-2 (4LVT); Table S7. Glide docking scores of docked ligands against Mcl-1 (5LOF); Figure S1. BOxNaf3 flexibility and compactness across Bcl-xL (2YXJ), Bcl-2 (4LVT), and Mcl-1 (5LOF). Top: 2D structure of BOxNaf1 with atom indices. For each target (rows): left, ligand heavy-atom RMSF (frames aligned on protein backbone); right, protein Cα RMSF per residue, (green bars mark contact residues); bottom of each row, ligand radius of gyration (rGyr) time series with distribution inset; Figure S2. BOxPhCl1 flexibility and compactness across Bcl-xL (2YXJ), Bcl-2 (4LVT), and Mcl-1 (5LOF). Top: 2D structure of BOxNaf1 with atom indices. For each target (rows): left, ligand heavy-atom RMSF (frames aligned on protein backbone); right, protein Cα RMSF per residue, (green bars mark contact residues); bottom of each row, ligand radius of gyration (rGyr) time series with distribution inset; Figure S3. BT1 flexibility and compactness across Bcl-xL (2YXJ), Bcl-2 (4LVT), and Mcl-1 (5LOF). Top: 2D structure of BOxNaf1 with atom indices. For each target (rows): left, ligand heavy-atom RMSF (frames aligned on protein backbone); right, protein Cα RMSF per residue, (green bars mark contact residues); bottom of each row, ligand radius of gyration (rGyr) time series with distribution inset; Figure S4. BT3 flexibility and compactness across Bcl-xL (2YXJ), Bcl-2 (4LVT), and Mcl-1 (5LOF). Top: 2D structure of BOxNaf1 with atom indices. For each target (rows): left, ligand heavy-atom RMSF (frames aligned on protein backbone); right, protein Cα RMSF per residue, (green bars mark contact residues); bottom of each row, ligand radius of gyration (rGyr) time series with distribution inset.
