# Supplementary material for: Targeting Anti-Apoptotic Bcl-2 Proteins with Triterpene-Heterocyclic Derivatives: A Combined Dual Docking and Molecular Dynamics Study
Source: Molecules. 2025 Sep 29;30(19):3919. doi: 10.3390/molecules30193919 (PMC12526515; doi:10.3390/molecules30193919)
Supplement: Supplementary file 1 [file molecules-30-03919-s001.zip › molecules-3860171-supplementary.pdf]

SUPPLEMENTARY MATERIAL

Targeting Anti-Apoptotic Bcl-2 Proteins with Triterpene-Heterocyclic Derivatives: A Combined Dual Docking and Molecular Dynamics Study

Marius Mioc [1,2,†], Silvia Gruin [3,†], Armand Gogulescu [3,*], Oana Bătrîna [1,2], Mihaela Jorgovan [1,2], Bogdan-Ionuț Mara [1,2,4] and Codruța Șoica [1,2]

1 Faculty of Pharmacy, "Victor Babes" University of Medicine and Pharmacy, Eftimie Murgu Square, No. 2, 300041 Timisoara, Romania; marius.mioc@umft.ro (M.M.); oana.esanu@umft.ro (O.B.); mihaela.coban@umft.ro (M.J.); mara.bogdan@umft.ro (B.M.); codrutasoica@umft.ro (C.Ș.)

2 Research Center for Experimental Pharmacology and Drug Design (X-Pharm Design), "Victor Babes" University of Medicine and Pharmacy, Eftimie Murgu Square, No. 2, 300041 Timișoara, Romania

3 Faculty of Medicine, "Victor Babes" University of Medicine and Pharmacy, Eftimie Murgu Square, No. 2, 300041 Timisoara, Romania; silvia.gruin@gmail.com

4 Coriolan Dragulescu Institute of Chemistry, Romanian Academy, Bv. M. Viteazu, No. 24, 300223 Timisoara, Romania

* Correspondence: gogulescu.armand@umft.ro

† These authors contributed equally to this work.

Abstract

Anti-apoptotic Bcl-2 family proteins (Bcl-2, Bcl-xL, and Mcl-1), are often overexpressed in cancer, which aids tumor growth and treatment resistance. As a result, these proteins are excellent candidates for novel anticancer drugs. Within this study a virtual library of betuline derivatives was built and screened as possible Bcl-2, Bcl-XL, and Mcl-1 inhibitors. For every target, molecular docking simulations were performed using two different engines (AutoDock Vina and Glide). The ligands that most frequently appeared among the top candidates were shortlisted after comparing the top-20 hits from both docking scoring functions. To assess binding stability, five of these promising compounds were chosen and run through 100 ns molecular dynamics (MD) simulations in complex with every target protein. Key persistent intermolecular contacts were identified from MD contact frequency histograms, and stability was evaluated using root-mean-square deviation (RMSD) profiles of protein–ligand complexes following equilibration. Two substances, BOxNaf1 and BT3, stood out among the screened derivatives as the most stable binders to all three Bcl-2 family targets. These compounds were found to be highly stable, broad-spectrum binders of Bcl-2, Bcl-XL, and Mcl-1 overall using this dual docking and MD analysis approach, indicating their potential for further development toward anticancer therapy.

Contents

Table S1. SMILE strings of the constructed Bet derivatives
Table S2. Vina docking scores of docked ligands against Bcl-XL (2YXJ)
Table S3. Vina docking scores of docked ligands against Bcl-2 (4LVT)
Table S4. Vina docking scores of docked ligands against Mcl-1 (5LOF)
Table S5. Glide docking scores of docked ligands against Bcl-XL (2YXJ)
Table S6. Glide docking scores of docked ligands against Bcl-2 (4LVT)

Table S7. Glide docking scores of docked ligands against Mcl-1 (5LOF)

Figure S1. BOxNaf3 flexibility and compactness across Bcl-xL (2YXJ), Bcl-2 (4LVT), and Mcl-1 (5LOF). Top: 2D structure of BOxNaf1 with atom indices. For each target (rows): left, ligand heavy-atom RMSF (frames aligned on protein backbone); right, protein Cα RMSF per residue, (green bars mark contact residues); bottom of each row, ligand radius of gyration (rGyr) time series with distribution inset.

Figure S2. BOxPhCl1 flexibility and compactness across Bcl-xL (2YXJ), Bcl-2 (4LVT), and Mcl-1 (5LOF). Top: 2D structure of BOxNaf1 with atom indices. For each target (rows): left, ligand heavy-atom RMSF (frames aligned on protein backbone); right, protein Cα RMSF per residue, (green bars mark contact residues); bottom of each row, ligand radius of gyration (rGyr) time series with distribution inset.

Figure S3. BT1 flexibility and compactness across Bcl-xL (2YXJ), Bcl-2 (4LVT), and Mcl-1 (5LOF). Top: 2D structure of BOxNaf1 with atom indices. For each target (rows): left, ligand heavy-atom RMSF (frames aligned on protein backbone); right, protein Cα RMSF per residue, (green bars mark contact residues); bottom of each row, ligand radius of gyration (rGyr) time series with distribution inset.

Figure S4. BT3 flexibility and compactness across Bcl-xL (2YXJ), Bcl-2 (4LVT), and Mcl-1 (5LOF). Top: 2D structure of BOxNaf1 with atom indices. For each target (rows): left, ligand heavy-atom RMSF (frames aligned on protein backbone); right, protein Cα RMSF per residue, (green bars mark contact residues); bottom of each row, ligand radius of gyration (rGyr) time series with distribution inset.

Table S1. SMILE strings of the constructed Bet derivatives

Ligand ID	SMILES
B	CC(=C)[C@@H]1CC[C@]2([C@H]1[C@H]3CC[C@@H]4[C@]5(CC[C@@H](C([C@@H]5CC[C@]4([C@@]3(CC2)C)C)(C)C)O)C)CO
BA	CC(=C)[C@@H]1CC[C@]2([C@H]1[C@H]3CC[C@@H]4[C@]5(CC[C@@H](C([C@@H]5CC[C@]4([C@@]3(CC2)C)C)(C)C)O)C)C(=O)O
BOx1	CC(=C)[C@@H]1CC[C@]2(COCC(=O)Sc3ocnn3)CC[C@]4(C)[C@H](CC[C@@H]5[C@@]6(C)CC[C@H](OCC(=O)Sc7ocnn7)C(C)(C)[C@@H]6CC[C@@]45C)[C@@H]12
BOx2	CC(=C)[C@@H]1CC[C@]2(COCCC(=O)Sc3ocnn3)CC[C@]4(C)[C@H](CC[C@@H]5[C@@]6(C)CC[C@H](OCCC(=O)Sc7ocnn7)C(C)(C)[C@@H]6CC[C@@]45C)[C@@H]12
BOx3	CC(=C)[C@@H]1CC[C@]2(COCCCC(=O)Sc3ocnn3)CC[C@]4(C)[C@H](CC[C@@H]5[C@@]6(C)CC[C@H](OCCCC(=O)Sc7ocnn7)C(C)(C)[C@@H]6CC[C@@]45C)[C@@H]12
BOx4	CC(=C)[C@@H]1CC[C@]2(COCCCCC(=O)Sc3ocnn3)CC[C@]4(C)[C@H](CC[C@@H]5[C@@]6(C)CC[C@H](OCCCCC(=O)Sc7ocnn7)C(C)(C)[C@@H]6CC[C@@]45C)[C@@H]12
BOx5	CC(=C)[C@@H]1CC[C@]2(COCCCCCC(=O)Sc3ocnn3)CC[C@]4(C)[C@H](CC[C@@H]5[C@@]6(C)CC[C@H](OCCCCCC(=O)Sc7ocnn7)C(C)(C)[C@@H]6CC[C@@]45C)[C@@H]12
BOx6	CC(=C)[C@@H]1CC[C@]2(COCCCCCCC(=O)Sc3ocnn3)CC[C@]4(C)[C@H](CC[C@@H]5[C@@]6(C)CC[C@H](OCCCCCCC(=O)Sc7ocnn7)C(C)(C)[C@@H]6CC[C@@]45C)[C@@H]12
BOx7	CC(=C)[C@@H]1CC[C@]2(COCCCCCCCC(=O)Sc3ocnn3)CC[C@]4(C)[C@H](CC[C@@H]5[C@@]6(C)CC[C@H](OCCCCCCCC(=O)Sc7ocnn7)C(C)(C)[C@@H]6CC[C@@]45C)[C@@H]12
BOx8	CC(=C)[C@@H]1CC[C@]2(COCCCCCCCCC(=O)Sc3ocnn3)CC[C@]4(C)[C@H](CC[C@@H]5[C@@]6(C)CC[C@H](OCCCCCCCCC(=O)Sc7ocnn7)C(C)(C)[C@@H]6CC[C@@]45C)[C@@H]12
BOx9	CC(=C)[C@@H]1CC[C@]2(COCCCCCCCCCC(=O)Sc3ocnn3)CC[C@]4(C)[C@H](CC[C@@H]5[C@@]6(C)CC[C@H](OCCCCCCCCCCC(=O)Sc7ocnn7)C(C)(C)[C@@H]6CC[C@@]45C)[C@@H]12

BOxNaf1	CC(=C)[C@@H]1CC[C@]2(COCC(=O)Sc3oc(nn3)c4ccc5ccccc5c4)CC[C@]6(C)[C@H](CC[C@@H]7[C@@]8(C)CC[C@H](OCC(=O)Sc9oc(nn9)c%10ccc%11ccccc%11c%10)C(C)(C)[C@@H]8CC[C@@]67C)[C@@H]12
BOxNaf2	CC(=C)[C@@H]1CC[C@]2(COCCC(=O)Sc3oc(nn3)c4ccc5ccccc5c4)CC[C@]6(C)[C@H](CC[C@@H]7[C@@]8(C)CC[C@H](OCCC(=O)Sc9oc(nn9)c%10ccc%11ccccc%11c%10)C(C)(C)[C@@H]8CC[C@@]67C)[C@@H]12
BOxNaf3	CC(=C)[C@@H]1CC[C@]2(COCCCC(=O)Sc3oc(nn3)c4ccc5ccccc5c4)CC[C@]6(C)[C@H](CC[C@@H]7[C@@]8(C)CC[C@H](OCCCC(=O)Sc9oc(nn9)c%10ccc%11ccccc%11c%10)C(C)(C)[C@@H]8CC[C@@]67C)[C@@H]12
BOxNaf4	CC(=C)[C@@H]1CC[C@]2(COCCCCC(=O)Sc3oc(nn3)c4ccc5ccccc5c4)CC[C@]6(C)[C@H](CC[C@@H]7[C@@]8(C)CC[C@H](OCCCCC(=O)Sc9oc(nn9)c%10ccc%11ccccc%11c%10)C(C)(C)[C@@H]8CC[C@@]67C)[C@@H]12
BOxNaf5	CC(=C)[C@@H]1CC[C@]2(COCCCCCC(=O)Sc3oc(nn3)c4ccc5ccccc5c4)CC[C@]6(C)[C@H](CC[C@@H]7[C@@]8(C)CC[C@H](OCCCCCC(=O)Sc9oc(nn9)c%10ccc%11ccccc%11c%10)C(C)(C)[C@@H]8CC[C@@]67C)[C@@H]12
BOxNaf6	CC(=C)[C@@H]1CC[C@]2(COCCCCCCC(=O)Sc3oc(nn3)c4ccc5ccccc5c4)CC[C@]6(C)[C@H](CC[C@@H]7[C@@]8(C)CC[C@H](OCCCCCCC(=O)Sc9oc(nn9)c%10ccc%11ccccc%11c%10)C(C)(C)[C@@H]8CC[C@@]67C)[C@@H]12
BOxNaf7	CC(=C)[C@@H]1CC[C@]2(COCCCCCCCC(=O)Sc3oc(nn3)c4ccc5ccccc5c4)CC[C@]6(C)[C@H](CC[C@@H]7[C@@]8(C)CC[C@H](OCCCCCCCC(=O)Sc9oc(nn9)c%10ccc%11ccccc%11c%10)C(C)(C)[C@@H]8CC[C@@]67C)[C@@H]12
BOxNaf8	CC(=C)[C@@H]1CC[C@]2(COCCCCCCCCC(=O)Sc3oc(nn3)c4ccc5ccccc5c4)CC[C@]6(C)[C@H](CC[C@@H]7[C@@]8(C)CC[C@H](OCCCCCCCCC(=O)Sc9oc(nn9)c%10ccc%11ccccc%11c%10)C(C)(C)[C@@H]8CC[C@@]67C)[C@@H]12
BOxNaf9	CC(=C)[C@@H]1CC[C@]2(COCCCCCCCCCC(=O)Sc3oc(nn3)c4ccc5ccccc5c4)CC[C@]6(C)[C@H](CC[C@@H]7[C@@]8(C)CC[C@H](OCCCCCCCCCC(=O)Sc9oc(nn9)c%10ccc%11ccccc%11c%10)C(C)(C)[C@@H]8CC[C@@]67C)[C@@H]12
BOxPh1	CC(=C)[C@@H]1CC[C@]2(COCC(=O)Sc3oc(nn3)c4ccccc4)CC[C@]5(C)[C@H](CC[C@@H]6[C@@]7(C)CC[C@H](OCC(=O)Sc8oc(nn8)c9ccccc9)C(C)(C)[C@@H]7CC[C@@]56C)[C@@H]12
BOxPh2	CC(=C)[C@@H]1CC[C@]2(COCCC(=O)Sc3oc(nn3)c4ccccc4)CC[C@]5(C)[C@H](CC[C@@H]6[C@@]7(C)CC[C@H](OCCC(=O)Sc8oc(nn8)c9ccccc9)C(C)(C)[C@@H]7CC[C@@]56C)[C@@H]12
BOxPh3	CC(=C)[C@@H]1CC[C@]2(COCCCC(=O)Sc3oc(nn3)c4ccccc4)CC[C@]5(C)[C@H](CC[C@@H]6[C@@]7(C)CC[C@H](OCCCC(=O)Sc8oc(nn8)c9ccccc9)C(C)(C)[C@@H]7CC[C@@]56C)[C@@H]12
BOxPh4	CC(=C)[C@@H]1CC[C@]2(COCCCCC(=O)Sc3oc(nn3)c4ccccc4)CC[C@]5(C)[C@H](CC[C@@H]6[C@@]7(C)CC[C@H](OCCCCC(=O)Sc8oc(nn8)c9ccccc9)C(C)(C)[C@@H]7CC[C@@]56C)[C@@H]12
BOxPh5	CC(=C)[C@@H]1CC[C@]2(COCCCCCC(=O)Sc3oc(nn3)c4ccccc4)CC[C@]5(C)[C@H](CC[C@@H]6[C@@]7(C)CC[C@H](OCCCCCC(=O)Sc8oc(nn8)c9ccccc9)C(C)(C)[C@@H]7CC[C@@]56C)[C@@H]12
BOxPh6	CC(=C)[C@@H]1CC[C@]2(COCCCCCCC(=O)Sc3oc(nn3)c4ccccc4)CC[C@]5(C)[C@H](CC[C@@H]6[C@@]7(C)CC[C@H](OCCCCCCC(=O)Sc8oc(nn8)c9ccccc9)C(C)(C)[C@@H]7CC[C@@]56C)[C@@H]12
BOxPh7	CC(=C)[C@@H]1CC[C@]2(COCCCCCCCC(=O)Sc3oc(nn3)c4ccccc4)CC[C@]5(C)[C@H](CC[C@@H]6[C@@]7(C)CC[C@H](OCCCCCCCC(=O)Sc8oc(nn8)c9ccccc9)C(C)(C)[C@@H]7CC[C@@]56C)[C@@H]12
BOxPh8	CC(=C)[C@@H]1CC[C@]2(COCCCCCCCCC(=O)Sc3oc(nn3)c4ccccc4)CC[C@]5(C)[C@H](CC[C@@H]6[C@@]7(C)CC[C@H](OCCCCCCCCC(=O)Sc8oc(nn8)c9ccccc9)C(C)(C)[C@@H]7CC[C@@]56C)[C@@H]12
BOxPh9	CC(=C)[C@@H]1CC[C@]2(COCCCCCCCCCC(=O)Sc3oc(nn3)c4ccccc4)CC[C@]5(C)[C@H](CC[C@@H]6[C@@]7(C)CC[C@H](OCCCCCCCCCC(=O)Sc8oc(nn8)c9ccccc9)C(C)(C)[C@@H]7CC[C@@]56C)[C@@H]12
BOxPhCl1	CC(=C)[C@@H]1CC[C@]2(COCC(=O)Sc3oc(nn3)c4ccc(Cl)cc4)CC[C@]5(C)[C@H](CC[C@@H]6[C@@]7(C)CC[C@H](OCC(=O)Sc8oc(nn8)c9ccc(Cl)cc9)C(C)(C)[C@@H]7CC[C@@]56C)[C@@H]12
BOxPhCl2	CC(=C)[C@@H]1CC[C@]2(COCCC(=O)Sc3oc(nn3)c4ccc(Cl)cc4)CC[C@]5(C)[C@H](CC[C@@H]6[C@@]7(C)CC[C@H](OCCC(=O)Sc8oc(nn8)c9ccc(Cl)cc9)C(C)(C)[C@@H]7CC[C@@]56C)[C@@H]12
BOxPhCl3	CC(=C)[C@@H]1CC[C@]2(COCCCC(=O)Sc3oc(nn3)c4ccc(Cl)cc4)CC[C@]5(C)[C@H](CC[C@@H]6[C@@]7(C)CC[C@H](OCCCC(=O)Sc8oc(nn8)c9ccc(Cl)cc9)C(C)(C)[C@@H]7CC[C@@]56C)[C@@H]12
BOxPhCl4	CC(=C)[C@@H]1CC[C@]2(COCCCCC(=O)Sc3oc(nn3)c4ccc(Cl)cc4)CC[C@]5(C)[C@H](CC[C@@H]6[C@@]7(C)CC[C@H](OCCCCC(=O)Sc8oc(nn8)c9ccc(Cl)cc9)C(C)(C)[C@@H]7CC[C@@]56C)[C@@H]12
BOxPhCl5	CC(=C)[C@@H]1CC[C@]2(COCCCCCC(=O)Sc3oc(nn3)c4ccc(Cl)cc4)CC[C@]5(C)[C@H](CC[C@@H]6[C@@]7(C)CC[C@H](OCCCCCC(=O)Sc8oc(nn8)c9ccc(Cl)cc9)C(C)(C)[C@@H]7CC[C@@]56C)[C@@H]12
BOxPhCl6	CC(=C)[C@@H]1CC[C@]2(COCCCCCCC(=O)Sc3oc(nn3)c4ccc(Cl)cc4)CC[C@]5(C)[C@H](CC[C@@H]6[C@@]7(C)CC[C@H](OCCCCCCC(=O)Sc8oc(nn8)c9ccc(Cl)cc9)C(C)(C)[C@@H]7CC[C@@]56C)[C@@H]12
BOxPhCl7	CC(=C)[C@@H]1CC[C@]2(COCCCCCCCC(=O)Sc3oc(nn3)c4ccc(Cl)cc4)CC[C@]5(C)[C@H](CC[C@@H]6[C@@]7(C)CC[C@H](OCCCCCCCC(=O)Sc8oc(nn8)c9ccc(Cl)cc9)C(C)(C)[C@@H]7CC[C@@]56C)[C@@H]12
BOxPhCl8	CC(=C)[C@@H]1CC[C@]2(COCCCCCCCCC(=O)Sc3oc(nn3)c4ccc(Cl)cc4)CC[C@]5(C)[C@H](CC[C@@H]6[C@@]7(C)CC[C@H](OCCCCCCCCC(=O)Sc8oc(nn8)c9ccc(Cl)cc9)C(C)(C)[C@@H]7CC[C@@]56C)[C@@H]12
BOxPhCl9	CC(=C)[C@@H]1CC[C@]2(COCCCCCCCCCC(=O)Sc3oc(nn3)c4ccc(Cl)cc4)CC[C@]5(C)[C@H](CC[C@@H]6[C@@]7(C)CC[C@H](OCCCCCCCCCC(=O)Sc8oc(nn8)c9ccc(Cl)cc9)C(C)(C)[C@@H]7CC[C@@]56C)[C@@H]12
BOxPhDMN1	CN(C)c1ccc(cc1)c2oc(SC(=O)COC[C@]34CC[C@H]([C@@H]3[C@H]5CC[C@@H]6[C@@]7(C)CC[C@H](OCC(=O)Sc8oc(nn8)c9ccc(cc9)N(C)C)C(C)(C)[C@@H]7CC[C@@]6(C)[C@]5(C)CC4)C(=C)C)nn2
BOxPhDMN2	CN(C)c1ccc(cc1)c2oc(SC(=O)CCOC[C@]34CC[C@H]([C@@H]3[C@H]5CC[C@@H]6[C@@]7(C)CC[C@H](OCCC(=O)Sc8oc(nn8)c9ccc(cc9)N(C)C)C(C)(C)[C@@H]7CC[C@@]6(C)[C@]5(C)CC4)C(=C)C)nn2
BOxPhDMN3	CN(C)c1ccc(cc1)c2oc(SC(=O)CCCOC[C@]34CC[C@H]([C@@H]3[C@H]5CC[C@@H]6[C@@]7(C)CC[C@H](OCCCC(=O)Sc8oc(nn8)c9ccc(cc9)N(C)C)C(C)(C)[C@@H]7CC[C@@]6(C)[C@]5(C)CC4)C(=C)C)nn2
BOxPhDMN4	CN(C)c1ccc(cc1)c2oc(SC(=O)CCCCOC[C@]34CC[C@H]([C@@H]3[C@H]5CC[C@@H]6[C@@]7(C)CC[C@H](OCCCCC(=O)Sc8oc(nn8)c9ccc(cc9)N(C)C)C(C)(C)[C@@H]7CC[C@@]6(C)[C@]5(C)CC4)C(=C)C)nn2

BOxPh DMN5	CN(C)c1ccc(cc1)c2oc(SC(=O)CCCCCOC[C@]34CC[C@H]([C@@H]3[C@H]5CC[C@@H]6[C@@]7(C)CC[C@H](OCCCCCC(=O)Sc8oc(nn8)c9ccc(cc9)N(C)C)C(C)(C)[C@@H]7CC[C@@]6(C)[C@]5(C)CC4)C(=C)C)nn2
BOxPh DMN6	CN(C)c1ccc(cc1)c2oc(SC(=O)CCCCCCOC[C@]34CC[C@H]([C@@H]3[C@H]5CC[C@@H]6[C@@]7(C)CC[C@H](OCCCCCCC(=O)Sc8oc(nn8)c9ccc(cc9)N(C)C)C(C)(C)[C@@H]7CC[C@@]6(C)[C@]5(C)CC4)C(=C)C)nn2
BOxPh DMN7	CN(C)c1ccc(cc1)c2oc(SC(=O)CCCCCCCOC[C@]34CC[C@H]([C@@H]3[C@H]5CC[C@@H]6[C@@]7(C)CC[C@H](OCCCCCCCC(=O)Sc8oc(nn8)c9ccc(cc9)N(C)C)C(C)(C)[C@@H]7CC[C@@]6(C)[C@]5(C)CC4)C(=C)C)nn2
BOxPh DMN8	CN(C)c1ccc(cc1)c2oc(SC(=O)CCCCCCCCOC[C@]34CC[C@H]([C@@H]3[C@H]5CC[C@@H]6[C@@]7(C)CC[C@H](OCCCCCCCCC(=O)Sc8oc(nn8)c9ccc(cc9)N(C)C)C(C)(C)[C@@H]7CC[C@@]6(C)[C@]5(C)CC4)C(=C)C)nn2
BOxPh DMN9	CN(C)c1ccc(cc1)c2oc(SC(=O)CCCCCCCCCOC[C@]34CC[C@H]([C@@H]3[C@H]5CC[C@@H]6[C@@]7(C)CC[C@H](OCCCCCCCCCC(=O)Sc8oc(nn8)c9ccc(cc9)N(C)C)C(C)(C)[C@@H]7CC[C@@]6(C)[C@]5(C)CC4)C(=C)C)nn2
BOxPh OMe1	COc1ccc(cc1)c2oc(SC(=O)COC[C@]34CC[C@H]([C@@H]3[C@H]5CC[C@@H]6[C@@]7(C)CC[C@H](OCC(=O)Sc8oc(nn8)c9ccc(OC)cc9)C(C)(C)[C@@H]7CC[C@@]6(C)[C@]5(C)CC4)C(=C)C)nn2
BOxPh OMe2	COc1ccc(cc1)c2oc(SC(=O)CCOC[C@]34CC[C@H]([C@@H]3[C@H]5CC[C@@H]6[C@@]7(C)CC[C@H](OCCC(=O)Sc8oc(nn8)c9ccc(OC)cc9)C(C)(C)[C@@H]7CC[C@@]6(C)[C@]5(C)CC4)C(=C)C)nn2
BOxPh OMe3	COc1ccc(cc1)c2oc(SC(=O)CCCOC[C@]34CC[C@H]([C@@H]3[C@H]5CC[C@@H]6[C@@]7(C)CC[C@H](OCCCC(=O)Sc8oc(nn8)c9ccc(OC)cc9)C(C)(C)[C@@H]7CC[C@@]6(C)[C@]5(C)CC4)C(=C)C)nn2
BOxPh OMe4	COc1ccc(cc1)c2oc(SC(=O)CCCCOC[C@]34CC[C@H]([C@@H]3[C@H]5CC[C@@H]6[C@@]7(C)CC[C@H](OCCCCC(=O)Sc8oc(nn8)c9ccc(OC)cc9)C(C)(C)[C@@H]7CC[C@@]6(C)[C@]5(C)CC4)C(=C)C)nn2
BOxPh OMe5	COc1ccc(cc1)c2oc(SC(=O)CCCCCOC[C@]34CC[C@H]([C@@H]3[C@H]5CC[C@@H]6[C@@]7(C)CC[C@H](OCCCCCC(=O)Sc8oc(nn8)c9ccc(OC)cc9)C(C)(C)[C@@H]7CC[C@@]6(C)[C@]5(C)CC4)C(=C)C)nn2
BOxPh OMe6	COc1ccc(cc1)c2oc(SC(=O)CCCCCCOC[C@]34CC[C@H]([C@@H]3[C@H]5CC[C@@H]6[C@@]7(C)CC[C@H](OCCCCCCC(=O)Sc8oc(nn8)c9ccc(OC)cc9)C(C)(C)[C@@H]7CC[C@@]6(C)[C@]5(C)CC4)C(=C)C)nn2
BOxPh OMe7	COc1ccc(cc1)c2oc(SC(=O)CCCCCCCOC[C@]34CC[C@H]([C@@H]3[C@H]5CC[C@@H]6[C@@]7(C)CC[C@H](OCCCCCCCC(=O)Sc8oc(nn8)c9ccc(OC)cc9)C(C)(C)[C@@H]7CC[C@@]6(C)[C@]5(C)CC4)C(=C)C)nn2
BOxPh OMe8	COc1ccc(cc1)c2oc(SC(=O)CCCCCCCCOC[C@]34CC[C@H]([C@@H]3[C@H]5CC[C@@H]6[C@@]7(C)CC[C@H](OCCCCCCCCC(=O)Sc8oc(nn8)c9ccc(OC)cc9)C(C)(C)[C@@H]7CC[C@@]6(C)[C@]5(C)CC4)C(=C)C)nn2
BOxPh OMe9	COc1ccc(cc1)c2oc(SC(=O)CCCCCCCCCOC[C@]34CC[C@H]([C@@H]3[C@H]5CC[C@@H]6[C@@]7(C)CC[C@H](OCCCCCCCCCC(=O)Sc8oc(nn8)c9ccc(OC)cc9)C(C)(C)[C@@H]7CC[C@@]6(C)[C@]5(C)CC4)C(=C)C)nn2
BT1	CC(=C)[C@@H]1CC[C@]2(COCC(=O)On3nnc4ccccc34)CC[C@]5(C)[C@H](CC[C@@H]6[C@@]7(C)CC[C@H](OCC(=O)On8nnc9ccccc89)C(C)(C)[C@@H]7CC[C@@]56C)[C@@H]12
BT2	CC(=C)[C@@H]1CC[C@]2(COCCC(=O)On3nnc4ccccc34)CC[C@]5(C)[C@H](CC[C@@H]6[C@@]7(C)CC[C@H](OCCC(=O)On8nnc9ccccc89)C(C)(C)[C@@H]7CC[C@@]56C)[C@@H]12
BT3	CC(=C)[C@@H]1CC[C@]2(COCCCC(=O)On3nnc4ccccc34)CC[C@]5(C)[C@H](CC[C@@H]6[C@@]7(C)CC[C@H](OCCCC(=O)On8nnc9ccccc89)C(C)(C)[C@@H]7CC[C@@]56C)[C@@H]12
BT4	CC(=C)[C@@H]1CC[C@]2(COCCCCC(=O)On3nnc4ccccc34)CC[C@]5(C)[C@H](CC[C@@H]6[C@@]7(C)CC[C@H](OCCCCC(=O)On8nnc9ccccc89)C(C)(C)[C@@H]7CC[C@@]56C)[C@@H]12
BT5	CC(=C)[C@@H]1CC[C@]2(COCCCCCC(=O)On3nnc4ccccc34)CC[C@]5(C)[C@H](CC[C@@H]6[C@@]7(C)CC[C@H](OCCCCCC(=O)On8nnc9ccccc89)C(C)(C)[C@@H]7CC[C@@]56C)[C@@H]12
BT6	CC(=C)[C@@H]1CC[C@]2(COCCCCCCC(=O)On3nnc4ccccc34)CC[C@]5(C)[C@H](CC[C@@H]6[C@@]7(C)CC[C@H](OCCCCCCC(=O)On8nnc9ccccc89)C(C)(C)[C@@H]7CC[C@@]56C)[C@@H]12
BT7	CC(=C)[C@@H]1CC[C@]2(COCCCCCCCC(=O)On3nnc4ccccc34)CC[C@]5(C)[C@H](CC[C@@H]6[C@@]7(C)CC[C@H](OCCCCCCCC(=O)On8nnc9ccccc89)C(C)(C)[C@@H]7CC[C@@]56C)[C@@H]12
BT8	CC(=C)[C@@H]1CC[C@]2(COCCCCCCCCC(=O)On3nnc4ccccc34)CC[C@]5(C)[C@H](CC[C@@H]6[C@@]7(C)CC[C@H](OCCCCCCCCC(=O)On8nnc9ccccc89)C(C)(C)[C@@H]7CC[C@@]56C)[C@@H]12
BT9	CC(=C)[C@@H]1CC[C@]2(COCCCCCCCCCC(=O)On3nnc4ccccc34)CC[C@]5(C)[C@H](CC[C@@H]6[C@@]7(C)CC[C@H](OCCCCCCCCCC(=O)On8nnc9ccccc89)C(C)(C)[C@@H]7CC[C@@]56C)[C@@H]12
BTz1	CC(=C)[C@@H]1CC[C@]2(COCC(=O)Sc3nc[nH]n3)CC[C@]4(C)[C@H](CC[C@@H]5[C@@]6(C)CC[C@H](OCC(=O)Sc7nc[nH]n7)C(C)(C)[C@@H]6CC[C@@]45C)[C@@H]12
BTz2	CC(=C)[C@@H]1CC[C@]2(COCCC(=O)Sc3nc[nH]n3)CC[C@]4(C)[C@H](CC[C@@H]5[C@@]6(C)CC[C@H](OCCC(=O)Sc7nc[nH]n7)C(C)(C)[C@@H]6CC[C@@]45C)[C@@H]12
BTz3	CC(=C)[C@@H]1CC[C@]2(COCCCC(=O)Sc3nc[nH]n3)CC[C@]4(C)[C@H](CC[C@@H]5[C@@]6(C)CC[C@H](OCCCC(=O)Sc7nc[nH]n7)C(C)(C)[C@@H]6CC[C@@]45C)[C@@H]12
BTz4	CC(=C)[C@@H]1CC[C@]2(COCCCCC(=O)Sc3nc[nH]n3)CC[C@]4(C)[C@H](CC[C@@H]5[C@@]6(C)CC[C@H](OCCCCC(=O)Sc7nc[nH]n7)C(C)(C)[C@@H]6CC[C@@]45C)[C@@H]12
BTz5	CC(=C)[C@@H]1CC[C@]2(COCCCCCC(=O)Sc3nc[nH]n3)CC[C@]4(C)[C@H](CC[C@@H]5[C@@]6(C)CC[C@H](OCCCCCC(=O)Sc7nc[nH]n7)C(C)(C)[C@@H]6CC[C@@]45C)[C@@H]12
BTz6	CC(=C)[C@@H]1CC[C@]2(COCCCCCCC(=O)Sc3nc[nH]n3)CC[C@]4(C)[C@H](CC[C@@H]5[C@@]6(C)CC[C@H](OCCCCCCC(=O)Sc7nc[nH]n7)C(C)(C)[C@@H]6CC[C@@]45C)[C@@H]12
BTz7	CC(=C)[C@@H]1CC[C@]2(COCCCCCCCC(=O)Sc3nc[nH]n3)CC[C@]4(C)[C@H](CC[C@@H]5[C@@]6(C)CC[C@H](OCCCCCCCC(=O)Sc7nc[nH]n7)C(C)(C)[C@@H]6CC[C@@]45C)[C@@H]12
BTz8	CC(=C)[C@@H]1CC[C@]2(COCCCCCCCCC(=O)Sc3nc[nH]n3)CC[C@]4(C)[C@H](CC[C@@H]5[C@@]6(C)CC[C@H](OCCCCCCCCC(=O)Sc7nc[nH]n7)C(C)(C)[C@@H]6CC[C@@]45C)[C@@H]12

BTz9	CC(=C)[C@@H]1CC[C@]2(COCCCCCCCCCC(=O)Sc3nc[nH]n3)CC[C@]4(C)[C@H](CC[C@@H]5[C@@]6(C)CC[C@H](OCCCCCCCCCCC(=O)Sc7nc[nH]n7)C(C)(C)[C@@H]6CC[C@@]45C)[C@@H]12
BTzNaf1	CC(=C)[C@@H]1CC[C@]2(COCC(=O)Sc3n[nH]c(n3)c4ccc5ccccc5c4)CC[C@]6(C)[C@H](CC[C@@H]7[C@@]8(C)CC[C@H](OCC(=O)Sc9n[nH]c(n9)c%10ccc%11ccccc%11c%10)C(C)(C)[C@@H]8CC[C@@]67C)[C@@H]12
BTzNaf2	CC(=C)[C@@H]1CC[C@]2(COCCC(=O)Sc3n[nH]c(n3)c4ccc5ccccc5c4)CC[C@]6(C)[C@H](CC[C@@H]7[C@@]8(C)CC[C@H](OCCC(=O)Sc9n[nH]c(n9)c%10ccc%11ccccc%11c%10)C(C)(C)[C@@H]8CC[C@@]67C)[C@@H]12
BTzNaf3	CC(=C)[C@@H]1CC[C@]2(COCCCC(=O)Sc3n[nH]c(n3)c4ccc5ccccc5c4)CC[C@]6(C)[C@H](CC[C@@H]7[C@@]8(C)CC[C@H](OCCCC(=O)Sc9n[nH]c(n9)c%10ccc%11ccccc%11c%10)C(C)(C)[C@@H]8CC[C@@]67C)[C@@H]12
BTzNaf4	CC(=C)[C@@H]1CC[C@]2(COCCCCC(=O)Sc3n[nH]c(n3)c4ccc5ccccc5c4)CC[C@]6(C)[C@H](CC[C@@H]7[C@@]8(C)CC[C@H](OCCCCC(=O)Sc9n[nH]c(n9)c%10ccc%11ccccc%11c%10)C(C)(C)[C@@H]8CC[C@@]67C)[C@@H]12
BTzNaf5	CC(=C)[C@@H]1CC[C@]2(COCCCCCC(=O)Sc3n[nH]c(n3)c4ccc5ccccc5c4)CC[C@]6(C)[C@H](CC[C@@H]7[C@@]8(C)CC[C@H](OCCCCCC(=O)Sc9n[nH]c(n9)c%10ccc%11ccccc%11c%10)C(C)(C)[C@@H]8CC[C@@]67C)[C@@H]12
BTzNaf6	CC(=C)[C@@H]1CC[C@]2(COCCCCCCC(=O)Sc3n[nH]c(n3)c4ccc5ccccc5c4)CC[C@]6(C)[C@H](CC[C@@H]7[C@@]8(C)CC[C@H](OCCCCCCC(=O)Sc9n[nH]c(n9)c%10ccc%11ccccc%11c%10)C(C)(C)[C@@H]8CC[C@@]67C)[C@@H]12
BTzNaf7	CC(=C)[C@@H]1CC[C@]2(COCCCCCCCC(=O)Sc3n[nH]c(n3)c4ccc5ccccc5c4)CC[C@]6(C)[C@H](CC[C@@H]7[C@@]8(C)CC[C@H](OCCCCCCCC(=O)Sc9n[nH]c(n9)c%10ccc%11ccccc%11c%10)C(C)(C)[C@@H]8CC[C@@]67C)[C@@H]12
BTzNaf8	CC(=C)[C@@H]1CC[C@]2(COCCCCCCCCC(=O)Sc3n[nH]c(n3)c4ccc5ccccc5c4)CC[C@]6(C)[C@H](CC[C@@H]7[C@@]8(C)CC[C@H](OCCCCCCCCC(=O)Sc9n[nH]c(n9)c%10ccc%11ccccc%11c%10)C(C)(C)[C@@H]8CC[C@@]67C)[C@@H]12
BTzNaf9	CC(=C)[C@@H]1CC[C@]2(COCCCCCCCCCC(=O)Sc3n[nH]c(n3)c4ccc5ccccc5c4)CC[C@]6(C)[C@H](CC[C@@H]7[C@@]8(C)CC[C@H](OCCCCCCCCCC(=O)Sc9n[nH]c(n9)c%10ccc%11ccccc%11c%10)C(C)(C)[C@@H]8CC[C@@]67C)[C@@H]12
BTzPh1	CC(=C)[C@@H]1CC[C@]2(COCC(=O)Sc3n[nH]c(n3)c4ccccc4)CC[C@]5(C)[C@H](CC[C@@H]6[C@@]7(C)CC[C@H](OCC(=O)Sc8n[nH]c(n8)c9ccccc9)C(C)(C)[C@@H]7CC[C@@]56C)[C@@H]12
BTzPh2	CC(=C)[C@@H]1CC[C@]2(COCCC(=O)Sc3n[nH]c(n3)c4ccccc4)CC[C@]5(C)[C@H](CC[C@@H]6[C@@]7(C)CC[C@H](OCCC(=O)Sc8n[nH]c(n8)c9ccccc9)C(C)(C)[C@@H]7CC[C@@]56C)[C@@H]12
BTzPh3	CC(=C)[C@@H]1CC[C@]2(COCCCC(=O)Sc3n[nH]c(n3)c4ccccc4)CC[C@]5(C)[C@H](CC[C@@H]6[C@@]7(C)CC[C@H](OCCCC(=O)Sc8n[nH]c(n8)c9ccccc9)C(C)(C)[C@@H]7CC[C@@]56C)[C@@H]12
BTzPh4	CC(=C)[C@@H]1CC[C@]2(COCCCCC(=O)Sc3n[nH]c(n3)c4ccccc4)CC[C@]5(C)[C@H](CC[C@@H]6[C@@]7(C)CC[C@H](OCCCCC(=O)Sc8n[nH]c(n8)c9ccccc9)C(C)(C)[C@@H]7CC[C@@]56C)[C@@H]12
BTzPh5	CC(=C)[C@@H]1CC[C@]2(COCCCCCC(=O)Sc3n[nH]c(n3)c4ccccc4)CC[C@]5(C)[C@H](CC[C@@H]6[C@@]7(C)CC[C@H](OCCCCCC(=O)Sc8n[nH]c(n8)c9ccccc9)C(C)(C)[C@@H]7CC[C@@]56C)[C@@H]12
BTzPh6	CC(=C)[C@@H]1CC[C@]2(COCCCCCCC(=O)Sc3n[nH]c(n3)c4ccccc4)CC[C@]5(C)[C@H](CC[C@@H]6[C@@]7(C)CC[C@H](OCCCCCCC(=O)Sc8n[nH]c(n8)c9ccccc9)C(C)(C)[C@@H]7CC[C@@]56C)[C@@H]12
BTzPh7	CC(=C)[C@@H]1CC[C@]2(COCCCCCCCC(=O)Sc3n[nH]c(n3)c4ccccc4)CC[C@]5(C)[C@H](CC[C@@H]6[C@@]7(C)CC[C@H](OCCCCCCCC(=O)Sc8n[nH]c(n8)c9ccccc9)C(C)(C)[C@@H]7CC[C@@]56C)[C@@H]12
BTzPh8	CC(=C)[C@@H]1CC[C@]2(COCCCCCCCCC(=O)Sc3n[nH]c(n3)c4ccccc4)CC[C@]5(C)[C@H](CC[C@@H]6[C@@]7(C)CC[C@H](OCCCCCCCCC(=O)Sc8n[nH]c(n8)c9ccccc9)C(C)(C)[C@@H]7CC[C@@]56C)[C@@H]12
BTzPh9	CC(=C)[C@@H]1CC[C@]2(COCCCCCCCCCC(=O)Sc3n[nH]c(n3)c4ccccc4)CC[C@]5(C)[C@H](CC[C@@H]6[C@@]7(C)CC[C@H](OCCCCCCCCCC(=O)Sc8n[nH]c(n8)c9ccccc9)C(C)(C)[C@@H]7CC[C@@]56C)[C@@H]12
BTzPhCl1	CC(=C)[C@@H]1CC[C@]2(COCC(=O)Sc3n[nH]c(n3)c4ccc(Cl)cc4)CC[C@]5(C)[C@H](CC[C@@H]6[C@@]7(C)CC[C@H](OCC(=O)Sc8n[nH]c(n8)c9ccc(Cl)cc9)C(C)(C)[C@@H]7CC[C@@]56C)[C@@H]12
BTzPhCl2	CC(=C)[C@@H]1CC[C@]2(COCCC(=O)Sc3n[nH]c(n3)c4ccc(Cl)cc4)CC[C@]5(C)[C@H](CC[C@@H]6[C@@]7(C)CC[C@H](OCCC(=O)Sc8n[nH]c(n8)c9ccc(Cl)cc9)C(C)(C)[C@@H]7CC[C@@]56C)[C@@H]12
BTzPhCl3	CC(=C)[C@@H]1CC[C@]2(COCCCC(=O)Sc3n[nH]c(n3)c4ccc(Cl)cc4)CC[C@]5(C)[C@H](CC[C@@H]6[C@@]7(C)CC[C@H](OCCCC(=O)Sc8n[nH]c(n8)c9ccc(Cl)cc9)C(C)(C)[C@@H]7CC[C@@]56C)[C@@H]12
BTzPhCl4	CC(=C)[C@@H]1CC[C@]2(COCCCCC(=O)Sc3n[nH]c(n3)c4ccc(Cl)cc4)CC[C@]5(C)[C@H](CC[C@@H]6[C@@]7(C)CC[C@H](OCCCCC(=O)Sc8n[nH]c(n8)c9ccc(Cl)cc9)C(C)(C)[C@@H]7CC[C@@]56C)[C@@H]12
BTzPhCl5	CC(=C)[C@@H]1CC[C@]2(COCCCCCC(=O)Sc3n[nH]c(n3)c4ccc(Cl)cc4)CC[C@]5(C)[C@H](CC[C@@H]6[C@@]7(C)CC[C@H](OCCCCCC(=O)Sc8n[nH]c(n8)c9ccc(Cl)cc9)C(C)(C)[C@@H]7CC[C@@]56C)[C@@H]12
BTzPhCl6	CC(=C)[C@@H]1CC[C@]2(COCCCCCCC(=O)Sc3n[nH]c(n3)c4ccc(Cl)cc4)CC[C@]5(C)[C@H](CC[C@@H]6[C@@]7(C)CC[C@H](OCCCCCCC(=O)Sc8n[nH]c(n8)c9ccc(Cl)cc9)C(C)(C)[C@@H]7CC[C@@]56C)[C@@H]12
BTzPhCl7	CC(=C)[C@@H]1CC[C@]2(COCCCCCCCC(=O)Sc3n[nH]c(n3)c4ccc(Cl)cc4)CC[C@]5(C)[C@H](CC[C@@H]6[C@@]7(C)CC[C@H](OCCCCCCCC(=O)Sc8n[nH]c(n8)c9ccc(Cl)cc9)C(C)(C)[C@@H]7CC[C@@]56C)[C@@H]12
BTzPhCl8	CC(=C)[C@@H]1CC[C@]2(COCCCCCCCCC(=O)Sc3n[nH]c(n3)c4ccc(Cl)cc4)CC[C@]5(C)[C@H](CC[C@@H]6[C@@]7(C)CC[C@H](OCCCCCCCCC(=O)Sc8n[nH]c(n8)c9ccc(Cl)cc9)C(C)(C)[C@@H]7CC[C@@]56C)[C@@H]12
BTzPhCl9	CC(=C)[C@@H]1CC[C@]2(COCCCCCCCCCC(=O)Sc3n[nH]c(n3)c4ccc(Cl)cc4)CC[C@]5(C)[C@H](CC[C@@H]6[C@@]7(C)CC[C@H](OCCCCCCCCCC(=O)Sc8n[nH]c(n8)c9ccc(Cl)cc9)C(C)(C)[C@@H]7CC[C@@]56C)[C@@H]12
BTzPhDMN1	CN(C)c1ccc(cc1)c2nc(SC(=O)COC[C@]34CC[C@H]([C@@H]3[C@H]5CC[C@@H]6[C@@]7(C)CC[C@H](OCC(=O)Sc8n[nH]c(n8)c9ccc(cc9)N(C)C)C(C)(C)[C@@H]7CC[C@@]6(C)[C@]5(C)CC4)C(=C)C)n[nH]2
BTzPhDMN2	CN(C)c1ccc(cc1)c2nc(SC(=O)CCOC[C@]34CC[C@H]([C@@H]3[C@H]5CC[C@@H]6[C@@]7(C)CC[C@H](OCCC(=O)Sc8n[nH]c(n8)c9ccc(cc9)N(C)C)C(C)(C)[C@@H]7CC[C@@]6(C)[C@]5(C)CC4)C(=C)C)n[nH]2
BTzPhDMN3	CN(C)c1ccc(cc1)c2nc(SC(=O)CCCOC[C@]34CC[C@H]([C@@H]3[C@H]5CC[C@@H]6[C@@]7(C)CC[C@H](OCCCC(=O)Sc8n[nH]c(n8)c9ccc(cc9)N(C)C)C(C)(C)[C@@H]7CC[C@@]6(C)[C@]5(C)CC4)C(=C)C)n[nH]2

BTzPhDMN4	CN(C)c1ccc(cc1)c2nc(SC(=O)CCCCOC[C@]34CC[C@H]([C@@H]3[C@H]5CC[C@@H]6[C@@]7(C)CC[C@H](OCCCCC(=O)Sc8n[nH]c(n8)c9ccc(cc9)N(C)C)C(C)(C)[C@@H]7CC[C@@]6(C)[C@]5(C)CC4)C(=C)C)n[nH]2
BTzPhDMN5	CN(C)c1ccc(cc1)c2nc(SC(=O)CCCCCOC[C@]34CC[C@H]([C@@H]3[C@H]5CC[C@@H]6[C@@]7(C)CC[C@H](OCCCCCC(=O)Sc8n[nH]c(n8)c9ccc(cc9)N(C)C)C(C)(C)[C@@H]7CC[C@@]6(C)[C@]5(C)CC4)C(=C)C)n[nH]2
BTzPhDMN6	CN(C)c1ccc(cc1)c2nc(SC(=O)CCCCCCOC[C@]34CC[C@H]([C@@H]3[C@H]5CC[C@@H]6[C@@]7(C)CC[C@H](OCCCCCCC(=O)Sc8n[nH]c(n8)c9ccc(cc9)N(C)C)C(C)(C)[C@@H]7CC[C@@]6(C)[C@]5(C)CC4)C(=C)C)n[nH]2
BTzPhDMN7	CN(C)c1ccc(cc1)c2nc(SC(=O)CCCCCCCOC[C@]34CC[C@H]([C@@H]3[C@H]5CC[C@@H]6[C@@]7(C)CC[C@H](OCCCCCCCC(=O)Sc8n[nH]c(n8)c9ccc(cc9)N(C)C)C(C)(C)[C@@H]7CC[C@@]6(C)[C@]5(C)CC4)C(=C)C)n[nH]2
BTzPhDMN8	CN(C)c1ccc(cc1)c2nc(SC(=O)CCCCCCCCOC[C@]34CC[C@H]([C@@H]3[C@H]5CC[C@@H]6[C@@]7(C)CC[C@H](OCCCCCCCCC(=O)Sc8n[nH]c(n8)c9ccc(cc9)N(C)C)C(C)(C)[C@@H]7CC[C@@]6(C)[C@]5(C)CC4)C(=C)C)n[nH]2
BTzPhDMN9	CN(C)c1ccc(cc1)c2nc(SC(=O)CCCCCCCCCOC[C@]34CC[C@H]([C@@H]3[C@H]5CC[C@@H]6[C@@]7(C)CC[C@H](OCCCCCCCCCC(=O)Sc8n[nH]c(n8)c9ccc(cc9)N(C)C)C(C)(C)[C@@H]7CC[C@@]6(C)[C@]5(C)CC4)C(=C)C)n[nH]2
BTzPhOMe1	COc1ccc(cc1)c2nc(SC(=O)COC[C@]34CC[C@H]([C@@H]3[C@H]5CC[C@@H]6[C@@]7(C)CC[C@H](OCC(=O)Sc8n[nH]c(n8)c9ccc(OC)cc9)C(C)(C)[C@@H]7CC[C@@]6(C)[C@]5(C)CC4)C(=C)C)n[nH]2
BTzPhOMe2	COc1ccc(cc1)c2nc(SC(=O)CCOC[C@]34CC[C@H]([C@@H]3[C@H]5CC[C@@H]6[C@@]7(C)CC[C@H](OCCC(=O)Sc8n[nH]c(n8)c9ccc(OC)cc9)C(C)(C)[C@@H]7CC[C@@]6(C)[C@]5(C)CC4)C(=C)C)n[nH]2
BTzPhOMe3	COc1ccc(cc1)c2nc(SC(=O)CCCOC[C@]34CC[C@H]([C@@H]3[C@H]5CC[C@@H]6[C@@]7(C)CC[C@H](OCCCC(=O)Sc8n[nH]c(n8)c9ccc(OC)cc9)C(C)(C)[C@@H]7CC[C@@]6(C)[C@]5(C)CC4)C(=C)C)n[nH]2
BTzPhOMe4	COc1ccc(cc1)c2nc(SC(=O)CCCCOC[C@]34CC[C@H]([C@@H]3[C@H]5CC[C@@H]6[C@@]7(C)CC[C@H](OCCCCC(=O)Sc8n[nH]c(n8)c9ccc(OC)cc9)C(C)(C)[C@@H]7CC[C@@]6(C)[C@]5(C)CC4)C(=C)C)n[nH]2
BTzPhOMe5	COc1ccc(cc1)c2nc(SC(=O)CCCCCOC[C@]34CC[C@H]([C@@H]3[C@H]5CC[C@@H]6[C@@]7(C)CC[C@H](OCCCCCC(=O)Sc8n[nH]c(n8)c9ccc(OC)cc9)C(C)(C)[C@@H]7CC[C@@]6(C)[C@]5(C)CC4)C(=C)C)n[nH]2
BTzPhOMe6	COc1ccc(cc1)c2nc(SC(=O)CCCCCCOC[C@]34CC[C@H]([C@@H]3[C@H]5CC[C@@H]6[C@@]7(C)CC[C@H](OCCCCCCC(=O)Sc8n[nH]c(n8)c9ccc(OC)cc9)C(C)(C)[C@@H]7CC[C@@]6(C)[C@]5(C)CC4)C(=C)C)n[nH]2
BTzPhOMe7	COc1ccc(cc1)c2nc(SC(=O)CCCCCCCOC[C@]34CC[C@H]([C@@H]3[C@H]5CC[C@@H]6[C@@]7(C)CC[C@H](OCCCCCCCC(=O)Sc8n[nH]c(n8)c9ccc(OC)cc9)C(C)(C)[C@@H]7CC[C@@]6(C)[C@]5(C)CC4)C(=C)C)n[nH]2
BTzPhOMe8	COc1ccc(cc1)c2nc(SC(=O)CCCCCCCCOC[C@]34CC[C@H]([C@@H]3[C@H]5CC[C@@H]6[C@@]7(C)CC[C@H](OCCCCCCCCC(=O)Sc8n[nH]c(n8)c9ccc(OC)cc9)C(C)(C)[C@@H]7CC[C@@]6(C)[C@]5(C)CC4)C(=C)C)n[nH]2
BTzPhOMe9	COc1ccc(cc1)c2nc(SC(=O)CCCCCCCCCOC[C@]34CC[C@H]([C@@H]3[C@H]5CC[C@@H]6[C@@]7(C)CC[C@H](OCCCCCCCCCC(=O)Sc8n[nH]c(n8)c9ccc(OC)cc9)C(C)(C)[C@@H]7CC[C@@]6(C)[C@]5(C)CC4)C(=C)C)n[nH]2

Table S2. Vina docking scores of docked ligands against Bcl-XL (2YXJ)

Ligand	Binding Affinity	rmsd/ub	rmsd/lb
2yxjdock_BT2	-11,4	0	0
2yxjdock_BT1	-11,3	0	0
2yxjdock_BT2	-11,2	1,721	1,347
2yxjdock_BT1	-11,1	13,165	3,684
2yxjdock_BOxNaf1	-10,9	0	0
2yxjdock_2yxjlig	-10,8	0	0
2yxjdock_BT1	-10,7	5,974	3,095
2yxjdock_BOxNaf1	-10,7	7,249	3,745
2yxjdock_BOxNaf2	-10,7	0	0
2yxjdock_BOxNaf3	-10,7	0	0
2yxjdock_BOxPhCl1	-10,7	0	0
2yxjdock_BT1	-10,6	11,233	1,406
2yxjdock_BT2	-10,6	13,967	3,814
2yxjdock_BT2	-10,6	13,41	3,746
2yxjdock_BOxNaf1	-10,6	13,475	3,283
2yxjdock_BOxNaf2	-10,6	2,592	1,903
2yxjdock_BOxNaf2	-10,6	13,33	2,3
2yxjdock_BOxNaf2	-10,5	13,892	2,255
2yxjdock_BOxPhCl1	-10,5	5,011	3,83
2yxjdock_BT2	-10,4	11,488	1,614

2yxjdock_BtzPhCl1	-10,4	0	0
2yxjdock_BOxNaf1	-10,4	10,192	5,3
2yxjdock_BOxNaf1	-10,4	5,105	2,176
2yxjdock_BOxNaf3	-10,4	13,296	1,485
2yxjdock_BOxPh1	-10,4	0	0
2yxjdock_BT3	-10,3	0	0
2yxjdock_BOxPhCl4	-10,3	0	0
2yxjdock_BtzPh2	-10,2	0	0
2yxjdock_BtzPhDMN1	-10,2	0	0
2yxjdock_BOxPhCl1	-10,2	13,42	4,529
2yxjdock_BOxPhCl1	-10,2	2,087	1,736
2yxjdock_BT1	-10,1	6,345	3,688
2yxjdock_BT1	-10,1	3,266	1,933
2yxjdock_BT5	-10,1	0	0
2yxjdock_2yxjlig	-10,1	2,085	1,407
2yxjdock_BtzPh2	-10,1	2,601	1,759
2yxjdock_BtzPhOMe1	-10,1	0	0
2yxjdock_BtzPhOMe1	-10,1	2,985	2,129
2yxjdock_BOxNaf1	-10,1	5,337	2,359
2yxjdock_BOxPhCl2	-10,1	0	0
2yxjdock_BOxPhCl4	-10,1	13,072	1,96
2yxjdock_BOxPhCl4	-10,1	13,121	2,47
2yxjdock_BT3	-10	3,293	1,751
2yxjdock_BT5	-10	4,224	2,025
2yxjdock_2yxjlig	-10	1,95	1,295
2yxjdock_BtzPhCl1	-10	12,72	1,656
2yxjdock_BtzPhOMe1	-10	5,601	3,102
2yxjdock_BOxNaf1	-10	12,348	3,427
2yxjdock_BOxNaf3	-10	13,872	2,806
2yxjdock_BOxNaf4	-10	0	0
2yxjdock_BT1	-9,9	11,3	1,355
2yxjdock_BT1	-9,9	3,945	2,307
2yxjdock_BT1	-9,9	11,122	2,395
2yxjdock_BtzPh2	-9,9	13	2,601
2yxjdock_BtzPh2	-9,9	14,402	3,474
2yxjdock_BtzPhCl1	-9,9	14,168	4,014
2yxjdock_BOxNaf4	-9,9	3,057	1,776
2yxjdock_BOxNaf5	-9,9	0	0
2yxjdock_BOxPhCl4	-9,9	3,51	2,063
2yxjdock_BOxPhCl4	-9,9	3,35	2,143
2yxjdock_BT8	-9,8	0	0
2yxjdock_BtzPh2	-9,8	13,85	3,073
2yxjdock_BtzPhCl1	-9,8	12,905	1,31
2yxjdock_BtzPhCl2	-9,8	0	0
2yxjdock_BtzPhDMN1	-9,8	13,485	3,122
2yxjdock_BOxNaf2	-9,8	14,378	1,832

2yxjdock_BOxPhOMe2	-9,8	0	0
2yxjdock_BT4	-9,7	0	0
2yxjdock_BtzPh2	-9,7	3,651	2,04
2yxjdock_BtzPhOMe1	-9,7	13,609	2,925
2yxjdock_BOxNaf2	-9,7	13,535	2,525
2yxjdock_BOxNaf5	-9,7	2,995	1,802
2yxjdock_BOxPh1	-9,7	4,201	3,03
2yxjdock_BOxPhCl1	-9,7	11,42	5,326
2yxjdock_BOxPhCl1	-9,7	9,769	4,997
2yxjdock_BOxPhCl4	-9,7	13,648	2,34
2yxjdock_BT3	-9,6	11,208	1,931
2yxjdock_BT3	-9,6	3,566	1,907
2yxjdock_BT4	-9,6	11,366	1,651
2yxjdock_BT4	-9,6	3,506	2,572
2yxjdock_BT4	-9,6	11,783	1,718
2yxjdock_BtzPh1	-9,6	0	0
2yxjdock_BtzPhCl2	-9,6	4,344	2,633
2yxjdock_BtzPhCl2	-9,6	3,575	2,456
2yxjdock_BtzPhCl2	-9,6	5,131	3,071
2yxjdock_BOxPhCl1	-9,6	13,285	4,404
2yxjdock_BOxPhOMe3	-9,6	0	0
2yxjdock_BT2	-9,5	13,188	3,507
2yxjdock_BT7	-9,5	0	0
2yxjdock_2yxjlig	-9,5	2,768	1,977
2yxjdock_BOxNaf3	-9,5	13,306	3,865
2yxjdock_BOxPh2	-9,5	0	0
2yxjdock_BOxPhCl1	-9,5	12,892	4,526
2yxjdock_BOxPhCl2	-9,5	3,865	2,105
2yxjdock_BOxPhCl2	-9,5	4,886	2,972
2yxjdock_BOxPhOMe1	-9,5	0	0
2yxjdock_BT2	-9,4	2,23	1,659
2yxjdock_BT3	-9,4	10,872	1,779
2yxjdock_BT3	-9,4	6,191	4,037
2yxjdock_BT7	-9,4	12,23	1,58
2yxjdock_BT7	-9,4	6,114	2,798
2yxjdock_BT8	-9,4	2,571	1,461
2yxjdock_BtzPhCl2	-9,4	5,478	3,141
2yxjdock_BtzPhDMN1	-9,4	13,567	2,883
2yxjdock_BtzPhDMN2	-9,4	0	0
2yxjdock_BtzPhOMe1	-9,4	13,06	3,309
2yxjdock_BOxNaf1	-9,4	10,393	4,56
2yxjdock_BOxNaf1	-9,4	6,157	2,779
2yxjdock_BOxNaf3	-9,4	3,631	2,087
2yxjdock_BOxNaf3	-9,4	12,731	3,174
2yxjdock_BOxNaf5	-9,4	3,641	2,198
2yxjdock_BOxPh1	-9,4	4,174	2,292

2yxjdock_BOxPh2	-9,4	2,055	1,563
2yxjdock_BOxPhCl4	-9,4	4,915	2,655
2yxjdock_BOxPhOMe1	-9,4	5,503	3,808
2yxjdock_BOxPhOMe1	-9,4	4,449	2,522
2yxjdock_BOxPhOMe1	-9,4	1,824	1,339
2yxjdock_BT2	-9,3	3,78	1,84
2yxjdock_BT3	-9,3	10,673	1,385
2yxjdock_BT4	-9,3	2,055	1,55
2yxjdock_BT4	-9,3	13,251	3,14
2yxjdock_BT4	-9,3	13,298	3,334
2yxjdock_BT5	-9,3	3,533	1,725
2yxjdock_BT7	-9,3	12,872	1,653
2yxjdock_BT7	-9,3	13,65	2,828
2yxjdock_BT8	-9,3	4,273	2,191
2yxjdock_2yxjlig	-9,3	2,437	1,745
2yxjdock_BtzPh1	-9,3	12,093	3,179
2yxjdock_BtzPh1	-9,3	2,51	1,873
2yxjdock_BtzPh1	-9,3	12,421	2,775
2yxjdock_BtzPh2	-9,3	6,951	3,884
2yxjdock_BtzPhCl1	-9,3	10,51	6,883
2yxjdock_BtzPhOMe1	-9,3	13,717	3,338
2yxjdock_BOx5	-9,3	0	0
2yxjdock_BOxNaf4	-9,3	4,089	2,041
2yxjdock_BOxPh1	-9,3	2,162	1,527
2yxjdock_BOxPh1	-9,3	4,293	2,524
2yxjdock_BOxPh1	-9,3	2,384	1,927
2yxjdock_BOxPh2	-9,3	4,062	2,129
2yxjdock_BOxPhCl1	-9,3	10,19	5,68
2yxjdock_BOxPhCl4	-9,3	3,236	2,231
2yxjdock_BOxPhCl4	-9,3	5,016	2,863
2yxjdock_BT2	-9,2	12,348	2,76
2yxjdock_BT3	-9,2	11,184	1,894
2yxjdock_BT3	-9,2	5,935	3,124
2yxjdock_BT4	-9,2	6,151	3,1
2yxjdock_BT4	-9,2	13,065	2,823
2yxjdock_BT7	-9,2	12,164	2,012
2yxjdock_2yxjlig	-9,2	3,777	2,3
2yxjdock_2yxjlig	-9,2	13,375	4,904
2yxjdock_BtzPh1	-9,2	11,889	2,78
2yxjdock_BtzPh1	-9,2	4,965	2,624
2yxjdock_BtzPh1	-9,2	5,283	2,451
2yxjdock_BtzPhDMN1	-9,2	12,539	3,301
2yxjdock_BtzPhDMN2	-9,2	13,106	1,697
2yxjdock_BtzPhDMN2	-9,2	2,373	1,639
2yxjdock_BtzPhOMe1	-9,2	12,923	2,635
2yxjdock_BOxNaf2	-9,2	4,782	2,243

2yxjdock_BOxNaf3	-9,2	4,227	2,173
2yxjdock_BOxPhCl2	-9,2	4,15	2,382
2yxjdock_BT5	-9,1	12,856	2,688
2yxjdock_BT6	-9,1	0	0
2yxjdock_BT7	-9,1	6,469	3,094
2yxjdock_BT7	-9,1	13,733	2,961
2yxjdock_BT8	-9,1	4,312	1,962
2yxjdock_Btz1	-9,1	0	0
2yxjdock_BtzPh1	-9,1	12,055	2,971
2yxjdock_BtzPh1	-9,1	4,81	2,436
2yxjdock_BtzPh2	-9,1	13,336	2,961
2yxjdock_BtzPh2	-9,1	5,354	3,067
2yxjdock_BtzPhCl1	-9,1	12,72	3,005
2yxjdock_BtzPhDMN1	-9,1	12,427	2,794
2yxjdock_BtzPhOMe1	-9,1	6,627	4,026
2yxjdock_BtzPhOMe2	-9,1	0	0
2yxjdock_BOxNaf3	-9,1	4,236	2,185
2yxjdock_BOxNaf4	-9,1	13,268	1,585
2yxjdock_BT7	-9	12,449	2,212
2yxjdock_BtzPhDMN1	-9	13,35	2,978
2yxjdock_BtzPhDMN1	-9	12,804	1,324
2yxjdock_BtzPhOMe1	-9	12,978	3,297
2yxjdock_BtzPhOMe2	-9	13,115	3,258
2yxjdock_BOxNaf2	-9	14,179	2,529
2yxjdock_BOxPh1	-9	7,729	4,528
2yxjdock_BOxPh2	-9	13,94	2,336
2yxjdock_BOxPh4	-9	0	0
2yxjdock_BOxPhCl2	-9	4,998	2,709
2yxjdock_BOxPhCl3	-9	0	0
2yxjdock_BOxPhCl3	-9	12,326	1,827
2yxjdock_BOxPhCl5	-9	0	0
2yxjdock_BOxPhDMN2	-9	0	0
2yxjdock_BOxPhDMN3	-9	0	0
2yxjdock_BT8	-8,9	14,168	2,98
2yxjdock_BT8	-8,9	4,128	2,041
2yxjdock_2yxjlig	-8,9	13,459	4,876
2yxjdock_Btz1	-8,9	5,802	3,241
2yxjdock_BtzPhCl1	-8,9	7,335	3,466
2yxjdock_BtzPhCl2	-8,9	12,35	3,535
2yxjdock_BtzPhDMN2	-8,9	13,58	3,292
2yxjdock_BtzPhOMe2	-8,9	5,364	3,377
2yxjdock_BOxNaf7	-8,9	0	0
2yxjdock_BOxPh3	-8,9	0	0
2yxjdock_BOxPh4	-8,9	13,171	1,735
2yxjdock_BOxPhCl3	-8,9	12,346	1,572
2yxjdock_BOxPhOMe2	-8,9	4,643	2,32

2yxjdock_BOxPhOMe3	-8,9	12,81	1,553
2yxjdock_BOxPhOMe3	-8,9	13,282	1,246
2yxjdock_BA	-8,8	0	0
2yxjdock_BT5	-8,8	13,001	3,528
2yxjdock_BT5	-8,8	2,05	1,549
2yxjdock_BT5	-8,8	12,375	1,873
2yxjdock_BT6	-8,8	11,977	1,598
2yxjdock_BT8	-8,8	3,238	1,81
2yxjdock_BT8	-8,8	13,537	1,789
2yxjdock_2yxjlig	-8,8	13,397	5,242
2yxjdock_BtzPhCl1	-8,8	7,77	4,245
2yxjdock_BtzPhDMN1	-8,8	13,067	3,152
2yxjdock_BtzPhDMN2	-8,8	3,953	1,877
2yxjdock_BtzPhOMe2	-8,8	12,775	3,178
2yxjdock_BOxNaf2	-8,8	6,04	3,029
2yxjdock_BOxNaf4	-8,8	13,675	1,938
2yxjdock_BOxPh1	-8,8	12,417	3,834
2yxjdock_BOxPh3	-8,8	13,032	2,026
2yxjdock_BOxPh4	-8,8	13,021	1,695
2yxjdock_BOxPh4	-8,8	11,585	4,123
2yxjdock_BOxPh5	-8,8	0	0
2yxjdock_BOxPh5	-8,8	13,42	1,562
2yxjdock_BOxPhCl2	-8,8	5,336	3,189
2yxjdock_BOxPhCl5	-8,8	13,361	4,213
2yxjdock_BOxPhCl6	-8,8	0	0
2yxjdock_BOxPhOMe4	-8,8	0	0
2yxjdock_BOxPhOMe8	-8,8	0	0
2yxjdock_BT6	-8,7	2,493	1,304
2yxjdock_BT6	-8,7	2,523	1,401
2yxjdock_BT8	-8,7	12,84	2,36
2yxjdock_BtzPhCl1	-8,7	8,367	3,933
2yxjdock_BOx1	-8,7	0	0
2yxjdock_BOxPh1	-8,7	11,871	6,023
2yxjdock_BOxPh2	-8,7	12,603	1,886
2yxjdock_BOxPh5	-8,7	4,08	1,832
2yxjdock_BOxPhCl5	-8,7	13,25	1,474
2yxjdock_BOxPhOMe4	-8,7	13,48	1,412
2yxjdock_BOxPhOMe5	-8,7	0	0
2yxjdock_BT5	-8,6	12,76	2,777
2yxjdock_Btz2	-8,6	0	0
2yxjdock_BtzPhCl2	-8,6	2,417	1,948
2yxjdock_BtzPhCl2	-8,6	5,607	2,919
2yxjdock_BOxNaf3	-8,6	8,343	3,855
2yxjdock_BOxPh2	-8,6	4,936	3,303
2yxjdock_BOxPh2	-8,6	13,193	2,046
2yxjdock_BOxPh2	-8,6	13,066	2,622

2yxjdock_BOxPhDMN3	-8,6	13,593	1,478
2yxjdock_BOxPhDMN3	-8,6	13,975	2,14
2yxjdock_BOxPhOMe6	-8,6	0	0
2yxjdock_BT6	-8,5	12,126	1,445
2yxjdock_BT6	-8,5	3,135	1,79
2yxjdock_BT6	-8,5	12,621	1,905
2yxjdock_BT9	-8,5	0	0
2yxjdock_Bet	-8,5	0	0
2yxjdock_Btz2	-8,5	4,384	3,232
2yxjdock_BtzPhOMe2	-8,5	13,301	2,478
2yxjdock_BOx2	-8,5	0	0
2yxjdock_BOx3	-8,5	0	0
2yxjdock_BOxNaf7	-8,5	13,986	2,723
2yxjdock_BOxPh2	-8,5	3,201	2,153
2yxjdock_BOxPhCl5	-8,5	2,556	1,862
2yxjdock_BOxPhCl5	-8,5	12,32	2,137
2yxjdock_BOxPhCl7	-8,5	0	0
2yxjdock_BOxPhCl8	-8,5	0	0
2yxjdock_BOxPhDMN1	-8,5	0	0
2yxjdock_BOxPhDMN2	-8,5	4,963	2,517
2yxjdock_BOxPhDMN3	-8,5	6,772	2,905
2yxjdock_BOxPhOMe2	-8,5	12,842	2,236
2yxjdock_BT5	-8,4	11,408	1,835
2yxjdock_BT6	-8,4	5,99	2,696
2yxjdock_BT9	-8,4	2,919	2,061
2yxjdock_BT9	-8,4	2,563	1,3
2yxjdock_Bet	-8,4	9,178	4,941
2yxjdock_BtzPhCl2	-8,4	4,237	2,924
2yxjdock_BtzPhDMN2	-8,4	5,458	3,133
2yxjdock_BtzPhDMN2	-8,4	7,617	3,976
2yxjdock_BOx2	-8,4	4,276	2,978
2yxjdock_BOx3	-8,4	4,404	3,515
2yxjdock_BOxNaf5	-8,4	3,639	1,689
2yxjdock_BOxNaf6	-8,4	0	0
2yxjdock_BOxNaf8	-8,4	0	0
2yxjdock_BOxPh3	-8,4	2,012	1,598
2yxjdock_BOxPh3	-8,4	5,852	2,62
2yxjdock_BOxPh3	-8,4	5,341	2,58
2yxjdock_BOxPh3	-8,4	5,643	2,435
2yxjdock_BOxPh4	-8,4	2,422	1,878
2yxjdock_BOxPh6	-8,4	0	0
2yxjdock_BOxPh6	-8,4	13,768	2,305
2yxjdock_BOxPh6	-8,4	14,913	1,949
2yxjdock_BOxPh7	-8,4	0	0
2yxjdock_BOxPh8	-8,4	0	0
2yxjdock_BOxPh9	-8,4	0	0

2yxjdock_BOxPhCl8	-8,4	13,492	2,303
2yxjdock_BOxPhOMe2	-8,4	13,678	2,839
2yxjdock_BA	-8,3	10,04	5,695
2yxjdock_BT6	-8,3	11,772	2,142
2yxjdock_Btz2	-8,3	1,8	1,404
2yxjdock_Btz2	-8,3	11,827	3,448
2yxjdock_BOx1	-8,3	10,448	5,557
2yxjdock_BOx6	-8,3	0	0
2yxjdock_BOx6	-8,3	2,048	1,641
2yxjdock_BOxNaf6	-8,3	12,26	2,708
2yxjdock_BOxNaf6	-8,3	12,695	2,439
2yxjdock_BOxNaf6	-8,3	12,778	2,009
2yxjdock_BOxPhCl2	-8,3	4,303	2,865
2yxjdock_BOxPhDMN2	-8,3	13,535	2,368
2yxjdock_BOxPhDMN3	-8,3	4,262	2,075
2yxjdock_BOxPhOMe2	-8,3	11,917	4,187
2yxjdock_BOxPhOMe2	-8,3	12,99	2,602
2yxjdock_BOxPhOMe7	-8,3	0	0
2yxjdock_BOxPhOMe8	-8,3	15,019	3,835
2yxjdock_BOxPhOMe8	-8,3	14,256	4,197
2yxjdock_BOxPhOMe9	-8,3	0	0
2yxjdock_BA	-8,2	7,635	2,765
2yxjdock_BT9	-8,2	12,254	1,709
2yxjdock_BT9	-8,2	3,956	1,74
2yxjdock_Btz1	-8,2	6,746	4,5
2yxjdock_Btz1	-8,2	10,563	3,253
2yxjdock_BtzPhDMN2	-8,2	13,41	1,367
2yxjdock_BtzPhOMe2	-8,2	13,602	2,67
2yxjdock_BOx3	-8,2	5,461	3,776
2yxjdock_BOxNaf6	-8,2	4,976	3,227
2yxjdock_BOxPh3	-8,2	12,335	1,798
2yxjdock_BOxPh5	-8,2	4,583	2,648
2yxjdock_BOxPh6	-8,2	14,398	1,988
2yxjdock_BOxPh9	-8,2	13,558	1,794
2yxjdock_BOxPhDMN1	-8,2	14,036	4,487
2yxjdock_BOxPhDMN1	-8,2	12,435	4,342
2yxjdock_BOxPhOMe3	-8,2	12,818	2,965
2yxjdock_BOxPhOMe4	-8,2	13,441	2,003
2yxjdock_BOxPhOMe8	-8,2	6,347	4,308
2yxjdock_Btz1	-8,1	3,706	2,329
2yxjdock_Btz2	-8,1	2,984	2,409
2yxjdock_Btz2	-8,1	12,71	2,693
2yxjdock_BtzPhDMN1	-8,1	12,897	2,005
2yxjdock_BOx2	-8,1	10,599	1,968
2yxjdock_BOx2	-8,1	4,499	3,599
2yxjdock_BOx4	-8,1	0	0

2yxjdock_BOx4	-8,1	10,673	1,901
2yxjdock_BOx5	-8,1	4,495	2,566
2yxjdock_BOx5	-8,1	4,156	2,246
2yxjdock_BOxNaf4	-8,1	13,374	2,334
2yxjdock_BOxPh3	-8,1	13,357	2,647
2yxjdock_BOxPh3	-8,1	3,974	1,962
2yxjdock_BOxPh4	-8,1	4,179	2,234
2yxjdock_BOxPh4	-8,1	13,321	2,214
2yxjdock_BOxPh7	-8,1	2,767	1,818
2yxjdock_BOxPh7	-8,1	13,15	1,534
2yxjdock_BOxPh9	-8,1	13,798	1,869
2yxjdock_BOxPhCl2	-8,1	3,225	1,983
2yxjdock_BOxPhCl2	-8,1	13,028	3,24
2yxjdock_BOxPhCl5	-8,1	12,379	3,154
2yxjdock_BOxPhDMN1	-8,1	14,445	3,425
2yxjdock_BOxPhDMN2	-8,1	13,7	3,071
2yxjdock_BOxPhDMN8	-8,1	0	0
2yxjdock_BOxPhOMe2	-8,1	14,392	2,676
2yxjdock_BOxPhOMe7	-8,1	3,375	2,574
2yxjdock_BOxPhOMe9	-8,1	4,542	2,782
2yxjdock_BA	-8	3,821	1,818
2yxjdock_BT9	-8	12,578	1,796
2yxjdock_Btz1	-8	6,725	4,36
2yxjdock_BtzPhOMe2	-8	12,979	2,163
2yxjdock_BOx1	-8	10,137	4,91
2yxjdock_BOx2	-8	11,144	3,182
2yxjdock_BOx3	-8	6,121	4,153
2yxjdock_BOx5	-8	3,639	1,804
2yxjdock_BOx6	-8	5,198	2,849
2yxjdock_BOx7	-8	0	0
2yxjdock_BOx8	-8	0	0
2yxjdock_BOxNaf4	-8	14,438	2,048
2yxjdock_BOxNaf4	-8	13,09	2,576
2yxjdock_BOxNaf8	-8	15,058	2,856
2yxjdock_BOxPh5	-8	3,949	2,008
2yxjdock_BOxPh6	-8	4,557	2,013
2yxjdock_BOxPhCl3	-8	12,75	2,282
2yxjdock_BOxPhCl3	-8	13,57	2,459
2yxjdock_BOxPhCl3	-8	12,693	2,395
2yxjdock_BOxPhCl7	-8	3,927	2,01
2yxjdock_BOxPhDMN6	-8	0	0
2yxjdock_BOxPhOMe1	-8	13,57	3,051
2yxjdock_BOxPhOMe3	-8	4,938	2,227
2yxjdock_BOxPhOMe8	-8	3,756	2,534
2yxjdock_BOxPhOMe9	-8	6,226	3,934
2yxjdock_BA	-7,9	8,676	3,516

2yxjdock_Bet	-7,9	10,285	5,188
2yxjdock_Btz2	-7,9	2,293	2,014
2yxjdock_BOx1	-7,9	5,019	2,641
2yxjdock_BOx3	-7,9	5,756	3,902
2yxjdock_BOx3	-7,9	3,141	2,879
2yxjdock_BOx5	-7,9	2,229	1,907
2yxjdock_BOxNaf4	-7,9	13,997	2,364
2yxjdock_BOxNaf7	-7,9	5,769	3,258
2yxjdock_BOxNaf9	-7,9	0	0
2yxjdock_BOxPh6	-7,9	15,213	2,025
2yxjdock_BOxPh8	-7,9	13,113	1,938
2yxjdock_BOxPhCl3	-7,9	13,064	1,828
2yxjdock_BOxPhCl8	-7,9	14,809	2,248
2yxjdock_BOxPhDMN2	-7,9	4,144	2,115
2yxjdock_BOxPhDMN2	-7,9	4,735	2,602
2yxjdock_BOxPhOMe1	-7,9	4,448	2,4
2yxjdock_BOxPhOMe3	-7,9	12,651	1,987
2yxjdock_BOxPhOMe4	-7,9	13,419	2,011
2yxjdock_BOxPhOMe8	-7,9	14,141	1,686
2yxjdock_BT9	-7,8	12,422	1,766
2yxjdock_Btz1	-7,8	11,289	4,431
2yxjdock_BtzPhDMN2	-7,8	13,515	1,915
2yxjdock_BOx1	-7,8	5,545	3,395
2yxjdock_BOx6	-7,8	12,526	3,119
2yxjdock_BOx6	-7,8	13,056	2,145
2yxjdock_BOx7	-7,8	11,749	3,036
2yxjdock_BOxNaf5	-7,8	4,522	2,579
2yxjdock_BOxPh4	-7,8	6,413	3,055
2yxjdock_BOxPh7	-7,8	3,431	2,027
2yxjdock_BOxPh8	-7,8	6,124	2,923
2yxjdock_BOxPh8	-7,8	13,637	2,131
2yxjdock_BOxPhCl3	-7,8	12,917	2,118
2yxjdock_BOxPhCl6	-7,8	13,913	3,195
2yxjdock_BOxPhCl8	-7,8	13,509	2,139
2yxjdock_BOxPhCl8	-7,8	12,824	3,13
2yxjdock_BOxPhCl8	-7,8	14,348	2,832
2yxjdock_BOxPhDMN1	-7,8	13,583	3,108
2yxjdock_BOxPhDMN2	-7,8	14,019	2,065
2yxjdock_BOxPhDMN2	-7,8	14,956	2,061
2yxjdock_BOxPhDMN3	-7,8	6,332	3,294
2yxjdock_BOxPhDMN4	-7,8	0	0
2yxjdock_BOxPhDMN5	-7,8	0	0
2yxjdock_BOxPhOMe1	-7,8	6,637	3,065
2yxjdock_BOxPhOMe4	-7,8	3,057	2,035
2yxjdock_BOxPhOMe6	-7,8	2,381	1,499
2yxjdock_BOxPhOMe7	-7,8	7,27	3,086

2yxjdock_Btz1	-7,7	12,199	3,692
2yxjdock_Btz2	-7,7	2,807	2,214
2yxjdock_Btz2	-7,7	2,349	1,887
2yxjdock_BOx1	-7,7	10,758	5,623
2yxjdock_BOx3	-7,7	5,906	3,997
2yxjdock_BOx4	-7,7	10,5	1,684
2yxjdock_BOx5	-7,7	5,099	2,646
2yxjdock_BOx5	-7,7	2,634	2,08
2yxjdock_BOx7	-7,7	2,203	1,498
2yxjdock_BOxNaf5	-7,7	4,872	2,792
2yxjdock_BOxNaf8	-7,7	2,411	1,6
2yxjdock_BOxNaf8	-7,7	5,927	2,803
2yxjdock_BOxPh5	-7,7	13,586	2,312
2yxjdock_BOxPh8	-7,7	2,79	1,849
2yxjdock_BOxPh8	-7,7	2,028	1,557
2yxjdock_BOxPhCl3	-7,7	2,847	1,87
2yxjdock_BOxPhCl5	-7,7	12,486	3,438
2yxjdock_BOxPhCl6	-7,7	13,664	2,827
2yxjdock_BOxPhCl7	-7,7	6,383	4,035
2yxjdock_BOxPhCl8	-7,7	13,583	4,374
2yxjdock_BOxPhCl9	-7,7	0	0
2yxjdock_BOxPhDMN1	-7,7	8,12	4,206
2yxjdock_BOxPhDMN1	-7,7	4,46	2,543
2yxjdock_BOxPhOMe1	-7,7	13,141	1,948
2yxjdock_BA	-7,6	2,58	1,876
2yxjdock_BA	-7,6	7,444	3,179
2yxjdock_BT9	-7,6	12,827	2,269
2yxjdock_BT9	-7,6	4,043	1,749
2yxjdock_BOx2	-7,6	4,149	2,716
2yxjdock_BOx4	-7,6	2,024	1,663
2yxjdock_BOx4	-7,6	10,932	1,993
2yxjdock_BOx6	-7,6	3,718	2,621
2yxjdock_BOx6	-7,6	5,595	3,158
2yxjdock_BOxNaf5	-7,6	12,724	2,454
2yxjdock_BOxNaf5	-7,6	11,043	4,133
2yxjdock_BOxNaf6	-7,6	9,437	3
2yxjdock_BOxNaf7	-7,6	2,931	2,161
2yxjdock_BOxPh4	-7,6	13,062	3,764
2yxjdock_BOxPh5	-7,6	10,414	4,205
2yxjdock_BOxPh6	-7,6	13,921	2,325
2yxjdock_BOxPh6	-7,6	4,68	2,394
2yxjdock_BOxPh7	-7,6	2,925	1,494
2yxjdock_BOxPh8	-7,6	6,167	2,78
2yxjdock_BOxPhCl7	-7,6	13,765	2,013
2yxjdock_BOxPhCl8	-7,6	2,47	1,676
2yxjdock_BOxPhCl8	-7,6	5,46	2,21

2yxjdock_BOxPhDMN2	-7,6	14,552	2,263
2yxjdock_BOxPhDMN3	-7,6	6,424	3,196
2yxjdock_BOxPhDMN4	-7,6	3,195	2,065
2yxjdock_BOxPhDMN5	-7,6	5,894	2,402
2yxjdock_BOxPhDMN6	-7,6	4,2	2,157
2yxjdock_BOxPhDMN7	-7,6	0	0
2yxjdock_BOxPhOMe1	-7,6	13,021	2,726
2yxjdock_BOxPhOMe2	-7,6	13,098	4,585
2yxjdock_BOxPhOMe2	-7,6	12,746	2,91
2yxjdock_BOxPhOMe3	-7,6	4,087	1,998
2yxjdock_BOxPhOMe4	-7,6	12,855	2,614
2yxjdock_BOxPhOMe4	-7,6	13,163	3,134
2yxjdock_BOxPhOMe5	-7,6	8,7	4,199
2yxjdock_BOxPhOMe5	-7,6	5,9	3,878
2yxjdock_BOxPhOMe6	-7,6	13,376	2,51
2yxjdock_BA	-7,5	2,475	1,673
2yxjdock_Bet	-7,5	8,992	3,428
2yxjdock_Bet	-7,5	3,846	1,935
2yxjdock_Btz1	-7,5	11,259	4,129
2yxjdock_BtzPhOMe2	-7,5	13,09	3,4
2yxjdock_BOx1	-7,5	10,266	4,775
2yxjdock_BOx4	-7,5	11,403	2,321
2yxjdock_BOx6	-7,5	12,464	2,994
2yxjdock_BOxPhCl7	-7,5	6,2	3,665
2yxjdock_BOxPhDMN1	-7,5	6,198	3,095
2yxjdock_BOxPhDMN3	-7,5	4,04	2,243
2yxjdock_BOxPhDMN3	-7,5	5,381	2,731
2yxjdock_BOxPhDMN5	-7,5	10,77	3,882
2yxjdock_BOxPhOMe3	-7,5	3,895	2,04
2yxjdock_BOxPhOMe3	-7,5	2,512	1,762
2yxjdock_BOxPhOMe4	-7,5	13,893	3,232
2yxjdock_BOxPhOMe6	-7,5	13,914	2,022
2yxjdock_BA	-7,4	8,286	2,114
2yxjdock_Bet	-7,4	1,772	1,273
2yxjdock_BtzPhOMe2	-7,4	6,092	3,029
2yxjdock_BOx1	-7,4	11,316	5,503
2yxjdock_BOx1	-7,4	7,05	4,817
2yxjdock_BOx2	-7,4	11,273	3,435
2yxjdock_BOx2	-7,4	10,752	2,104
2yxjdock_BOx3	-7,4	6,39	4,633
2yxjdock_BOx6	-7,4	12,525	2,646
2yxjdock_BOx7	-7,4	12,101	3,395
2yxjdock_BOx8	-7,4	2,555	1,798
2yxjdock_BOxNaf9	-7,4	13,428	2,546
2yxjdock_BOxPh5	-7,4	2,956	1,839
2yxjdock_BOxPh8	-7,4	13,794	2,806

2yxjdock_BOxPhCl5	-7,4	13,358	2,458
2yxjdock_BOxPhDMN4	-7,4	3,893	2,501
2yxjdock_BOxPhDMN7	-7,4	4,163	2,11
2yxjdock_BOxPhOMe4	-7,4	10,456	4,271
2yxjdock_BOxPhOMe7	-7,4	13,766	2,618
2yxjdock_BOx3	-7,3	11,869	4,35
2yxjdock_BOx4	-7,3	11,122	2,114
2yxjdock_BOxNaf6	-7,3	12,574	2,071
2yxjdock_BOxNaf6	-7,3	7,423	2,834
2yxjdock_BOxNaf8	-7,3	15,793	2,339
2yxjdock_BOxPh5	-7,3	3,315	2,061
2yxjdock_BOxPh6	-7,3	5,151	2,634
2yxjdock_BOxPh7	-7,3	2,129	1,635
2yxjdock_BOxPh8	-7,3	13,762	1,863
2yxjdock_BOxPhCl6	-7,3	13,668	4,333
2yxjdock_BOxPhCl9	-7,3	13,768	3,996
2yxjdock_BOxPhDMN1	-7,3	2,025	1,491
2yxjdock_BOxPhOMe6	-7,3	13,952	1,885
2yxjdock_BOxPhOMe6	-7,3	14,107	2,042
2yxjdock_BOxPhOMe7	-7,3	14,184	2,966
2yxjdock_BOx4	-7,2	11,249	3,082
2yxjdock_BOx5	-7,2	11,726	5,6
2yxjdock_BOx9	-7,2	0	0
2yxjdock_BOxPh7	-7,2	5,444	2,927
2yxjdock_BOxPh7	-7,2	13,449	1,939
2yxjdock_BOxPhCl6	-7,2	5,421	2,693
2yxjdock_BOxPhCl7	-7,2	6	3,245
2yxjdock_BOxPhCl9	-7,2	13,619	2,723
2yxjdock_BOxPhCl9	-7,2	14,135	2,478
2yxjdock_BOxPhDMN4	-7,2	14,064	2,734
2yxjdock_BOxPhDMN9	-7,2	0	0
2yxjdock_BOxPhOMe9	-7,2	6,593	3,529
2yxjdock_Bet	-7,1	1,878	1,466
2yxjdock_Bet	-7,1	7,312	4,127
2yxjdock_BOx4	-7,1	12,175	3,427
2yxjdock_BOx8	-7,1	12,071	2,49
2yxjdock_BOxNaf6	-7,1	5,002	2,644
2yxjdock_BOxNaf8	-7,1	15,534	2,465
2yxjdock_BOxPhCl7	-7,1	6,973	4,239
2yxjdock_BOxPhCl7	-7,1	2,528	1,984
2yxjdock_BOxPhOMe5	-7,1	2,062	1,37
2yxjdock_BOxPhOMe9	-7,1	13,759	3,765
2yxjdock_BOxPhOMe9	-7,1	14,793	3,922
2yxjdock_BOxPhOMe9	-7,1	2,676	2,074
2yxjdock_BOxPhOMe9	-7,1	6,508	4,012
2yxjdock_Bet	-7	6,045	3,014

2yxjdock_BOx5	-7	3,862	1,915
2yxjdock_BOx7	-7	12,698	2,422
2yxjdock_BOx7	-7	2,222	1,833
2yxjdock_BOx8	-7	3,566	2,326
2yxjdock_BOx8	-7	11,59	2,155
2yxjdock_BOxNaf9	-7	6,952	4,571
2yxjdock_BOxPh9	-7	12,153	2,809
2yxjdock_BOxPhCl7	-7	3,909	2,157
2yxjdock_BOxPhCl9	-7	5,522	2,935
2yxjdock_BOxPhDMN5	-7	6,507	2,931
2yxjdock_BOxPhOMe6	-7	3,209	1,734
2yxjdock_BOxPhOMe6	-7	4,256	2,147
2yxjdock_BOxPhOMe8	-7	14,43	2,354
2yxjdock_BOx2	-6,9	11,778	3,919
2yxjdock_BOx7	-6,9	11,855	2,094
2yxjdock_BOx7	-6,9	12,29	3,105
2yxjdock_BOx8	-6,9	12,539	3,247
2yxjdock_BOx8	-6,9	11,647	2,862
2yxjdock_BOxNaf8	-6,9	3,632	2,103
2yxjdock_BOxPhCl5	-6,9	3,143	2,465
2yxjdock_BOxPhCl9	-6,9	5,171	2,619
2yxjdock_BOxPhCl9	-6,9	13,052	4,099
2yxjdock_BOxPhDMN4	-6,9	13,222	1,839
2yxjdock_BOxPhDMN8	-6,9	7,446	3,122
2yxjdock_BOxPhOMe5	-6,9	3,093	2,142
2yxjdock_BOxPhOMe6	-6,9	14,125	4,372
2yxjdock_BOxPhOMe8	-6,9	7,302	3,759
2yxjdock_BOxPhOMe8	-6,9	14,755	4,081
2yxjdock_BOx7	-6,8	11,939	2,267
2yxjdock_BOx8	-6,8	11,623	3,06
2yxjdock_BOx9	-6,8	3,133	1,945
2yxjdock_BOx9	-6,8	2,315	1,481
2yxjdock_BOx9	-6,8	5,099	2,811
2yxjdock_BOxNaf9	-6,8	13,765	3,399
2yxjdock_BOxPh7	-6,8	2,189	1,73
2yxjdock_BOxPh9	-6,8	13,162	2,353
2yxjdock_BOxPhCl9	-6,8	4,269	2,041
2yxjdock_BOxPhDMN4	-6,8	13,249	2,692
2yxjdock_BOxPhDMN5	-6,8	15,05	2,372
2yxjdock_BOxPhDMN5	-6,8	14,832	2,573
2yxjdock_BOxPhOMe5	-6,8	5,028	2,433
2yxjdock_BOx8	-6,7	7,874	3,547
2yxjdock_BOx9	-6,7	2,074	1,282
2yxjdock_BOx9	-6,7	12,749	3,534
2yxjdock_BOx9	-6,7	2,758	1,887
2yxjdock_BOxNaf7	-6,7	5,145	2,531

2yxjdock_BOxNaf9	-6,7	5,572	3,28
2yxjdock_BOxPhCl9	-6,7	12,965	3,674
2yxjdock_BOxPhDMN6	-6,7	3,69	1,985
2yxjdock_BOxPhDMN8	-6,7	1,999	1,541
2yxjdock_BOxPhDMN9	-6,7	4,336	2,27
2yxjdock_BOxPhOMe5	-6,7	3,545	2,081
2yxjdock_BOxPhCl6	-6,6	13,548	3,805
2yxjdock_BOxPhDMN4	-6,6	5,364	2,5
2yxjdock_BOxPhDMN4	-6,6	13,053	2,511
2yxjdock_BOxPhDMN8	-6,6	2,959	1,572
2yxjdock_BOxPhOMe5	-6,6	6,466	3,931
2yxjdock_BOxPh9	-6,5	12,206	2,726
2yxjdock_BOxPhDMN5	-6,5	14,598	1,871
2yxjdock_BOxPhDMN5	-6,5	5,261	2,896
2yxjdock_BOxPhOMe7	-6,5	13,684	2,948
2yxjdock_BOx9	-6,4	13,257	2,646
2yxjdock_BOxPhCl6	-6,4	5,845	2,754
2yxjdock_BOxPhDMN4	-6,4	14,079	3,102
2yxjdock_BOxPhDMN9	-6,4	13,531	2,301
2yxjdock_BOxPhOMe5	-6,4	6,525	3,824
2yxjdock_BOx9	-6,3	13,186	3,837
2yxjdock_BOxPhCl6	-6,3	13,999	3,296
2yxjdock_BOxPhDMN8	-6,3	13,223	3,228
2yxjdock_BOxPhDMN9	-6,3	12,939	1,953
2yxjdock_BOxPhDMN7	-6,2	14,932	3,534
2yxjdock_BOxNaf7	-6,1	4,146	1,76
2yxjdock_BOxNaf8	-6,1	10,777	5,5
2yxjdock_BOxPhDMN5	-6,1	2,683	1,694
2yxjdock_BOxPhDMN7	-6,1	8,276	3,357
2yxjdock_BOxPhDMN8	-6,1	13,084	3,141
2yxjdock_BOxPhDMN8	-6,1	4,941	2,844
2yxjdock_BOxNaf7	-6	14,381	3,198
2yxjdock_BOxNaf7	-6	14,16	3,098
2yxjdock_BOxPh9	-6	12,715	2,805
2yxjdock_BOxPhCl6	-6	14,8	2,658
2yxjdock_BOxNaf8	-5,9	15,982	2,485
2yxjdock_BOxPhDMN7	-5,9	2,302	1,766
2yxjdock_BOxPhDMN7	-5,9	14,416	4,26
2yxjdock_BOxPhDMN7	-5,8	3,765	2,273
2yxjdock_BOxPhDMN9	-5,8	3,864	1,837
2yxjdock_BOxPhOMe9	-5,8	6,938	3,494
2yxjdock_BOxPh9	-5,7	13,383	2,282
2yxjdock_BOxPhDMN7	-5,7	12,52	5,061
2yxjdock_BOxPhDMN8	-5,7	7,096	3,408
2yxjdock_BOxPhDMN8	-5,7	7,187	3,027
2yxjdock_BOxPhDMN9	-5,7	14,042	3,445

2yxjdock_BOxPhOMe7	-5,7	13,439	4,578
2yxjdock_BOxNaf9	-5,5	6,174	3,384
2yxjdock_BOxPhDMN6	-5,5	9,394	4,557
2yxjdock_BOxPhOMe7	-5,5	9,647	3,52
2yxjdock_BOxPhDMN6	-5,4	2,974	2,27
2yxjdock_BOxPhOMe7	-5,4	14,05	3,927
2yxjdock_BOxPhDMN7	-5,3	14,351	4,064
2yxjdock_BOxPhDMN6	-5,2	5,11	2,768
2yxjdock_BOxPhDMN6	-5,2	2,16	1,902
2yxjdock_BOxPhDMN9	-5,2	13,796	2,349
2yxjdock_BOxPhDMN6	-5,1	6,258	3,176
2yxjdock_BOxPhDMN6	-5,1	6,267	2,769
2yxjdock_BOxPhDMN9	-4,5	7,173	2,836
2yxjdock_BOxPhDMN9	-4,4	14,81	2,698

Table S3. Vina docking scores of docked ligands against Bcl-2 (4LVT)

Ligand	Binding Affinity	rmsd/ub	rmsd/lb
4lvtAdock_BTzNaf1	-11,7	0	0
4lvtAdock_4LVTlig	-11,4	0	0
4lvtAdock_BOxNaf3	-10,9	0	0
4lvtAdock_BT2	-10,7	0	0
4lvtAdock_BT2	-10,7	2,071	1,53
4lvtAdock_BOxNaf1	-10,6	0	0
4lvtAdock_BT1	-10,5	0	0
4lvtAdock_4LVTlig	-10,5	3,316	1,878
4lvtAdock_BTzNaf1	-10,3	4,009	2,49
4lvtAdock_BtzPh1	-10,3	0	0
4lvtAdock_BtzPh2	-10,3	0	0
4lvtAdock_BOxNaf4	-10,3	0	0
4lvtAdock_BTzNaf3	-10,2	0	0
4lvtAdock_BOxNaf2	-10,2	0	0
4lvtAdock_BOxNaf2	-10,2	2,201	1,455
4lvtAdock_BOxPh1	-10,2	0	0
4lvtAdock_BOxPh1	-10,2	3,888	2,376
4lvtAdock_BOxPh2	-10,2	0	0
4lvtAdock_BOxPhCl1	-10,2	0	0
4lvtAdock_BT1	-10,1	11,573	3,447
4lvtAdock_BT1	-10,1	2,87	2,052
4lvtAdock_BT3	-10,1	0	0
4lvtAdock_BTzNaf3	-10,1	13,695	2,868
4lvtAdock_BTzNaf3	-10,1	13,333	2,613
4lvtAdock_BtzPh1	-10,1	3,952	2,407
4lvtAdock_BT4	-10	0	0
4lvtAdock_BTzNaf2	-10	0	0
4lvtAdock_BtzPhCl1	-10	0	0
4lvtAdock_BOxNaf2	-10	5,63	2,449
4lvtAdock_BT2	-9,9	11,993	1,617

4lvtAdock_BTzNaf1	-9,9	12,68	2,001
4lvtAdock_BOxNaf1	-9,9	13,689	3,263
4lvtAdock_BOxNaf3	-9,9	4,422	1,882
4lvtAdock_BOxPh2	-9,9	4,797	2,473
4lvtAdock_4LVTlig	-9,9	14,742	6,87
4lvtAdock_BT2	-9,8	11,61	1,978
4lvtAdock_BT3	-9,8	1,998	1,352
4lvtAdock_BtzPh2	-9,8	2,716	1,993
4lvtAdock_4LVTlig	-9,8	3,264	1,873
4lvtAdock_BOxNaf1	-9,8	12,524	1,706
4lvtAdock_BOxPh1	-9,8	4,157	2,622
4lvtAdock_BOxPhCl2	-9,8	0	0
4lvtAdock_BOxPhOMe1	-9,8	0	0
4lvtAdock_BT1	-9,7	11,167	1,481
4lvtAdock_BT1	-9,7	11,447	1,69
4lvtAdock_BT2	-9,7	12,149	2,299
4lvtAdock_BTzNaf3	-9,7	13,599	3,113
4lvtAdock_BtzPh1	-9,7	2,861	1,846
4lvtAdock_BtzPhCl1	-9,7	1,652	1,348
4lvtAdock_BOxNaf4	-9,7	5,042	2,208
4lvtAdock_BT2	-9,6	12,245	3,253
4lvtAdock_BT3	-9,6	4,077	2,22
4lvtAdock_BtzPhCl2	-9,6	0	0
4lvtAdock_BOxNaf3	-9,6	14,394	3,203
4lvtAdock_BOxNaf4	-9,6	2,157	1,772
4lvtAdock_BOxPhCl2	-9,6	4,013	2,571
4lvtAdock_BtzPhCl1	-9,5	2,293	1,825
4lvtAdock_BtzPhCl2	-9,5	4,714	2,573
4lvtAdock_BtzPhOMe1	-9,5	0	0
4lvtAdock_BOxNaf1	-9,5	2,127	1,599
4lvtAdock_BOxNaf2	-9,5	3,455	1,502
4lvtAdock_BOxNaf4	-9,5	14,255	1,812
4lvtAdock_BOxNaf4	-9,5	14,205	2,412
4lvtAdock_BOxNaf5	-9,5	0	0
4lvtAdock_BOxPh4	-9,5	0	0
4lvtAdock_BOxPh4	-9,5	3,496	1,785
4lvtAdock_BOxPhCl2	-9,5	2,927	2,563
4lvtAdock_BT2	-9,4	5,149	2,747
4lvtAdock_BT2	-9,4	12,332	3,378
4lvtAdock_BT3	-9,4	5,45	2,17
4lvtAdock_BT3	-9,4	2,757	1,887
4lvtAdock_BT4	-9,4	2,33	1,916
4lvtAdock_BTzNaf2	-9,4	4	1,975
4lvtAdock_BTzNaf3	-9,4	5,411	3,093
4lvtAdock_BTzNaf4	-9,4	0	0
4lvtAdock_BTzNaf5	-9,4	0	0

4lvtAdock_BTzNaf5	-9,4	5,075	2,704
4lvtAdock_BtzPh3	-9,4	0	0
4lvtAdock_BtzPh3	-9,4	12,178	2,334
4lvtAdock_BtzPh5	-9,4	0	0
4lvtAdock_BtzPh5	-9,4	13,853	2,68
4lvtAdock_BtzPhDMN1	-9,4	0	0
4lvtAdock_BOxPh3	-9,4	0	0
4lvtAdock_BOxPhCl1	-9,4	4,041	2,495
4lvtAdock_BOxPhCl1	-9,4	3,978	2,477
4lvtAdock_BT1	-9,3	12,281	3,612
4lvtAdock_BT1	-9,3	5,252	3,808
4lvtAdock_BT2	-9,3	11,973	1,472
4lvtAdock_BT3	-9,3	12,23	2,497
4lvtAdock_BT4	-9,3	12,604	1,921
4lvtAdock_BTzNaf1	-9,3	13,304	2,788
4lvtAdock_BTzNaf3	-9,3	14,111	3,469
4lvtAdock_BtzPh1	-9,3	4,052	2,234
4lvtAdock_BtzPh3	-9,3	12,296	2,053
4lvtAdock_BtzPh6	-9,3	0	0
4lvtAdock_4LVTlig	-9,3	3,296	2,145
4lvtAdock_BOxNaf1	-9,3	14,514	3,969
4lvtAdock_BOxNaf4	-9,3	2,12	1,671
4lvtAdock_BOxNaf5	-9,3	14,424	1,907
4lvtAdock_BOxNaf6	-9,3	0	0
4lvtAdock_BOxPh2	-9,3	13,165	2,932
4lvtAdock_BOxPhCl1	-9,3	4,075	2,567
4lvtAdock_BOxPhCl3	-9,3	0	0
4lvtAdock_BT1	-9,2	5,848	3,55
4lvtAdock_BT5	-9,2	0	0
4lvtAdock_BT5	-9,2	11,594	1,246
4lvtAdock_BTzNaf1	-9,2	5,645	3,8
4lvtAdock_BtzPh2	-9,2	3,595	2,215
4lvtAdock_BtzPh2	-9,2	12,949	2,724
4lvtAdock_BtzPh4	-9,2	0	0
4lvtAdock_BtzPh5	-9,2	13,59	3,027
4lvtAdock_BOxNaf3	-9,2	13,541	1,688
4lvtAdock_BOxNaf3	-9,2	5,793	3,325
4lvtAdock_BOxPh6	-9,2	0	0
4lvtAdock_BOxPhCl1	-9,2	4,122	2,374
4lvtAdock_BOxPhCl2	-9,2	5,323	3,032
4lvtAdock_BT1	-9,1	4,276	2,772
4lvtAdock_BT3	-9,1	13,022	2,626
4lvtAdock_BT4	-9,1	11,836	1,922
4lvtAdock_BTzNaf1	-9,1	12,341	1,499
4lvtAdock_BTzNaf1	-9,1	4,257	2,47
4lvtAdock_BTzNaf5	-9,1	4,983	3,043

4lvtAdock_BtzPh1	-9,1	12,4	2,692
4lvtAdock_BOxNaf1	-9,1	13,346	2,318
4lvtAdock_BOxNaf2	-9,1	12,756	1,674
4lvtAdock_BOxPh2	-9,1	12,704	2,195
4lvtAdock_BOxPh3	-9,1	4,254	2,688
4lvtAdock_BOxPh3	-9,1	12,903	1,657
4lvtAdock_BOxPh3	-9,1	3,886	2,368
4lvtAdock_BOxPh4	-9,1	13,447	2,146
4lvtAdock_BOxPhCl2	-9,1	12,857	3,12
4lvtAdock_BT3	-9	11,916	2,554
4lvtAdock_BT5	-9	3,075	1,557
4lvtAdock_BTzNaf1	-9	13,443	2,687
4lvtAdock_BTzNaf3	-9	3,382	2,522
4lvtAdock_BtzPh1	-9	4,917	3,181
4lvtAdock_BtzPh2	-9	12,604	1,275
4lvtAdock_BtzPh3	-9	12,543	1,909
4lvtAdock_BtzPh5	-9	3,174	1,337
4lvtAdock_BtzPhCl1	-9	5,443	3,061
4lvtAdock_BtzPhCl2	-9	12,924	2,421
4lvtAdock_BtzPhCl3	-9	0	0
4lvtAdock_BtzPhOMe1	-9	4,403	1,983
4lvtAdock_BOxNaf1	-9	6,192	4,102
4lvtAdock_BOxNaf4	-9	5,812	2,906
4lvtAdock_BOxPh2	-9	3,417	2,102
4lvtAdock_BOxPh3	-9	3,076	1,846
4lvtAdock_BOxPh6	-9	2,548	1,962
4lvtAdock_BOxPhCl2	-9	13,072	3,563
4lvtAdock_BOxPhCl4	-9	0	0
4lvtAdock_BOxPhDMN2	-9	0	0
4lvtAdock_BOxPhOMe1	-9	4,092	2,046
4lvtAdock_BOxPhOMe1	-9	4,227	2,262
4lvtAdock_BOxPhOMe2	-9	0	0
4lvtAdock_BT5	-8,9	12,111	2,165
4lvtAdock_BT5	-8,9	4,949	2,257
4lvtAdock_BT7	-8,9	0	0
4lvtAdock_BT8	-8,9	0	0
4lvtAdock_BT9	-8,9	0	0
4lvtAdock_BTzNaf1	-8,9	6,952	4,109
4lvtAdock_BTzNaf4	-8,9	3,373	1,93
4lvtAdock_BTzNaf6	-8,9	0	0
4lvtAdock_BtzPh1	-8,9	3,727	2,081
4lvtAdock_BtzPh1	-8,9	3,742	2,374
4lvtAdock_BtzPh3	-8,9	12,738	2,569
4lvtAdock_BtzPh4	-8,9	12,966	2,501
4lvtAdock_BtzPh5	-8,9	12,878	2,07
4lvtAdock_BtzPhCl3	-8,9	3,516	2,838

4lvtAdock_BOxNaf3	-8,9	4,927	2,153
4lvtAdock_BOxNaf5	-8,9	3,81	2,08
4lvtAdock_BOxPh3	-8,9	12,822	2,176
4lvtAdock_BOxPh5	-8,9	0	0
4lvtAdock_BOxPh5	-8,9	3,444	2,381
4lvtAdock_BOxPhCl3	-8,9	12,69	2,466
4lvtAdock_BOxPhCl3	-8,9	4,237	2,53
4lvtAdock_BOxPhCl5	-8,9	0	0
4lvtAdock_BOxPhCl5	-8,9	3,145	2,102
4lvtAdock_BOxPhCl6	-8,9	0	0
4lvtAdock_BOxPhDMN1	-8,9	0	0
4lvtAdock_BOxPhDMN2	-8,9	6,066	3,508
4lvtAdock_BOxPhOMe5	-8,9	0	0
4lvtAdock_BT3	-8,8	13,386	3,073
4lvtAdock_BT4	-8,8	11,885	1,625
4lvtAdock_BT6	-8,8	0	0
4lvtAdock_BTzNaf2	-8,8	6,542	3,853
4lvtAdock_BTzNaf2	-8,8	12,842	6,822
4lvtAdock_BTzNaf2	-8,8	5,315	3,179
4lvtAdock_BtzPh1	-8,8	12,414	2,944
4lvtAdock_BtzPh3	-8,8	12,885	2,041
4lvtAdock_BtzPh4	-8,8	3,747	2,369
4lvtAdock_BtzPhCl3	-8,8	4,35	2,654
4lvtAdock_BtzPhCl3	-8,8	13,106	3,112
4lvtAdock_BtzPhCl5	-8,8	0	0
4lvtAdock_BtzPhDMN1	-8,8	4,601	2,499
4lvtAdock_4LVTlig	-8,8	2,342	1,939
4lvtAdock_4LVTlig	-8,8	3,7	2,692
4lvtAdock_BOxNaf2	-8,8	14,288	3,346
4lvtAdock_BOxNaf6	-8,8	13,901	2,222
4lvtAdock_BOxNaf6	-8,8	14,619	2,218
4lvtAdock_BOxPh1	-8,8	12,293	2,545
4lvtAdock_BOxPh3	-8,8	4,747	2,796
4lvtAdock_4LVTlig	-8,8	4,321	2,18
4lvtAdock_4LVTlig	-8,8	15,364	6,558
4lvtAdock_BOxPhCl1	-8,8	6,169	3,69
4lvtAdock_BOxPhCl1	-8,8	5,65	3,345
4lvtAdock_BOxPhCl1	-8,8	11,986	3,038
4lvtAdock_BOxPhCl1	-8,8	2,203	1,787
4lvtAdock_BOxPhCl3	-8,8	13,092	2,435
4lvtAdock_BOxPhCl6	-8,8	4,914	3,092
4lvtAdock_BOxPhCl6	-8,8	3,481	2,315
4lvtAdock_BOxPhOMe1	-8,8	13,337	3,531
4lvtAdock_BOxPhOMe3	-8,8	0	0
4lvtAdock_BOxPhOMe5	-8,8	2,189	1,772
4lvtAdock_BT5	-8,7	11,695	1,685

4lvtAdock_BT6	-8,7	4,87	2,286
4lvtAdock_BT8	-8,7	2,209	1,534
4lvtAdock_BT9	-8,7	2,336	1,547
4lvtAdock_BTz3	-8,7	0	0
4lvtAdock_BTzNaf3	-8,7	3,809	1,786
4lvtAdock_BTzNaf5	-8,7	2,852	2,119
4lvtAdock_BTzNaf8	-8,7	0	0
4lvtAdock_BtzPh4	-8,7	4,172	2,115
4lvtAdock_BtzPhCl2	-8,7	4,767	2,627
4lvtAdock_BtzPhDMN3	-8,7	0	0
4lvtAdock_BtzPhDMN4	-8,7	0	0
4lvtAdock_BtzPhOMe1	-8,7	4,228	2,149
4lvtAdock_BOxNaf4	-8,7	2,992	2,002
4lvtAdock_BOxPh1	-8,7	2,37	1,48
4lvtAdock_BOxPh2	-8,7	12,283	1,979
4lvtAdock_BOxPh2	-8,7	5,79	2,906
4lvtAdock_BOxPh2	-8,7	5,261	2,921
4lvtAdock_BOxPh4	-8,7	2,23	1,919
4lvtAdock_BOxPh4	-8,7	2,177	1,723
4lvtAdock_BOxPh5	-8,7	12,487	3,657
4lvtAdock_BOxPh6	-8,7	4,431	2,31
4lvtAdock_BOxPh6	-8,7	3,636	1,645
4lvtAdock_4LVTlig	-8,7	4,399	2,477
4lvtAdock_BOxPhCl2	-8,7	12,893	1,644
4lvtAdock_BOxPhCl5	-8,7	13,546	2,452
4lvtAdock_BOxPhCl6	-8,7	2,843	2,097
4lvtAdock_BOxPhCl6	-8,7	2,116	1,633
4lvtAdock_BOxPhDMN2	-8,7	4,715	2,709
4lvtAdock_BOxPhOMe2	-8,7	4,149	2,549
4lvtAdock_BOxPhOMe4	-8,7	0	0
4lvtAdock_BT5	-8,6	2,265	1,563
4lvtAdock_BT7	-8,6	4,001	1,904
4lvtAdock_BT9	-8,6	12,991	2,305
4lvtAdock_BTzNaf4	-8,6	13,746	1,86
4lvtAdock_BtzPh3	-8,6	12,721	2,624
4lvtAdock_BtzPh4	-8,6	13,276	3,086
4lvtAdock_BtzPh6	-8,6	13,749	3,213
4lvtAdock_BtzPh7	-8,6	0	0
4lvtAdock_BtzPhCl1	-8,6	5,627	3,501
4lvtAdock_BtzPhCl2	-8,6	12,174	2,036
4lvtAdock_BtzPhCl3	-8,6	13,328	3,428
4lvtAdock_BtzPhCl4	-8,6	0	0
4lvtAdock_BtzPhCl4	-8,6	2,004	1,526
4lvtAdock_BtzPhDMN3	-8,6	4,691	2,51
4lvtAdock_BtzPhOMe5	-8,6	0	0
4lvtAdock_4LVTlig	-8,6	5,147	2,653

4lvtAdock_BOxNaf1	-8,6	12,935	2,179
4lvtAdock_BOxNaf5	-8,6	3,608	2,218
4lvtAdock_BOxNaf9	-8,6	0	0
4lvtAdock_BOxPh4	-8,6	3,636	2,137
4lvtAdock_4LVTlig	-8,6	14,831	6,734
4lvtAdock_BOxPhCl2	-8,6	12,752	1,886
4lvtAdock_BOxPhCl5	-8,6	4,679	2,71
4lvtAdock_BOxPhDMN1	-8,6	2,382	1,591
4lvtAdock_BOxPhOMe1	-8,6	12,331	1,957
4lvtAdock_BTzNaf3	-8,5	3,962	3,349
4lvtAdock_BTzNaf4	-8,5	3,975	1,972
4lvtAdock_BTzNaf4	-8,5	6,752	3,983
4lvtAdock_BTzNaf6	-8,5	3,192	1,996
4lvtAdock_BTzNaf6	-8,5	3,836	2,431
4lvtAdock_BtzPh3	-8,5	13,613	2,914
4lvtAdock_BtzPh4	-8,5	4,143	2,493
4lvtAdock_BtzPh4	-8,5	12,849	3,242
4lvtAdock_BtzPh4	-8,5	13,074	2,552
4lvtAdock_BtzPh5	-8,5	14,248	3,956
4lvtAdock_BtzPhCl3	-8,5	5,984	3,515
4lvtAdock_BtzPhCl5	-8,5	14,057	1,84
4lvtAdock_BOx4	-8,5	0	0
4lvtAdock_BOxNaf1	-8,5	2,089	1,876
4lvtAdock_BOxNaf2	-8,5	5,701	2,876
4lvtAdock_BOxNaf3	-8,5	6,611	3,06
4lvtAdock_BOxNaf5	-8,5	2,756	1,973
4lvtAdock_BOxNaf6	-8,5	3,533	1,808
4lvtAdock_BOxNaf6	-8,5	14,785	2,098
4lvtAdock_BOxPh2	-8,5	4,323	2,082
4lvtAdock_BOxPh4	-8,5	13,34	2,175
4lvtAdock_BOxPh4	-8,5	4,609	2,931
4lvtAdock_BOxPh9	-8,5	0	0
4lvtAdock_BOxPhCl2	-8,5	12,526	3,73
4lvtAdock_BOxPhDMN1	-8,5	2,753	1,856
4lvtAdock_BOxPhOMe1	-8,5	13,463	3,27
4lvtAdock_BOxPhOMe2	-8,5	14,999	4,337
4lvtAdock_BOxPhOMe6	-8,5	0	0
4lvtAdock_BOxPhOMe6	-8,5	14,861	2,402
4lvtAdock_BT5	-8,4	4,315	2,056
4lvtAdock_BT5	-8,4	2,792	1,343
4lvtAdock_BT6	-8,4	11,942	1,58
4lvtAdock_BTzNaf2	-8,4	4,031	2,004
4lvtAdock_BTzNaf7	-8,4	0	0
4lvtAdock_BTzNaf7	-8,4	14,265	2,28
4lvtAdock_BTzNaf8	-8,4	14,477	3,057
4lvtAdock_Btz7	-8,4	0	0

4lvtAdock_BtzPhCl2	-8,4	3,972	2,155
4lvtAdock_BtzPhCl6	-8,4	0	0
4lvtAdock_BtzPhDMN1	-8,4	13,694	2,179
4lvtAdock_BtzPhDMN1	-8,4	5,448	3,352
4lvtAdock_BtzPhDMN1	-8,4	4,99	2,757
4lvtAdock_BtzPhOMe2	-8,4	0	0
4lvtAdock_BtzPhOMe4	-8,4	0	0
4lvtAdock_BtzPhOMe5	-8,4	2,843	1,976
4lvtAdock_BOx1	-8,4	0	0
4lvtAdock_BOx2	-8,4	0	0
4lvtAdock_BOxNaf4	-8,4	5,652	2,502
4lvtAdock_BOxNaf5	-8,4	2,19	1,643
4lvtAdock_BOxNaf8	-8,4	0	0
4lvtAdock_BOxPh5	-8,4	3,437	2,154
4lvtAdock_BOxPhCl3	-8,4	13,859	3,004
4lvtAdock_BOxPhCl3	-8,4	4,268	2,739
4lvtAdock_BOxPhCl4	-8,4	13,605	2,128
4lvtAdock_BOxPhCl5	-8,4	2,802	2,232
4lvtAdock_BOxPhCl6	-8,4	14,286	3,734
4lvtAdock_BOxPhCl6	-8,4	15,532	3,509
4lvtAdock_BOxPhCl7	-8,4	0	0
4lvtAdock_BOxPhCl7	-8,4	2,127	1,624
4lvtAdock_BOxPhDMN3	-8,4	0	0
4lvtAdock_BOxPhDMN4	-8,4	0	0
4lvtAdock_BOxPhDMN4	-8,4	14,488	2,495
4lvtAdock_BOxPhOMe1	-8,4	5,033	3,058
4lvtAdock_BOxPhOMe2	-8,4	3,927	2,453
4lvtAdock_BOxPhOMe5	-8,4	3,163	2,527
4lvtAdock_BT8	-8,3	12,583	1,797
4lvtAdock_BTz3	-8,3	11,262	2,226
4lvtAdock_BTz5	-8,3	0	0
4lvtAdock_BTzNaf4	-8,3	12,434	7,189
4lvtAdock_BTzNaf4	-8,3	4,231	2,221
4lvtAdock_BTzNaf6	-8,3	15,441	2,177
4lvtAdock_BTzNaf7	-8,3	13,757	2,297
4lvtAdock_Btz2	-8,3	0	0
4lvtAdock_BtzPh3	-8,3	12,49	1,577
4lvtAdock_BtzPh4	-8,3	3,714	2,034
4lvtAdock_BtzPhCl1	-8,3	5,409	3,139
4lvtAdock_BtzPhCl3	-8,3	2,217	1,76
4lvtAdock_BtzPhCl4	-8,3	6,313	3,612
4lvtAdock_BtzPhOMe1	-8,3	12,091	3,233
4lvtAdock_BtzPhOMe1	-8,3	4,177	2,551
4lvtAdock_BOx4	-8,3	4,982	3,412
4lvtAdock_BOx5	-8,3	0	0
4lvtAdock_BOxNaf2	-8,3	13,775	2,599

4lvtAdock_BOxNaf3	-8,3	8,898	4,266
4lvtAdock_BOxNaf6	-8,3	4,826	2,106
4lvtAdock_BOxNaf7	-8,3	0	0
4lvtAdock_BOxPh1	-8,3	11,741	2,157
4lvtAdock_BOxPh1	-8,3	3,745	2,266
4lvtAdock_BOxPh3	-8,3	5,192	2,819
4lvtAdock_BOxPh3	-8,3	4,907	2,614
4lvtAdock_BOxPh5	-8,3	2,401	1,669
4lvtAdock_BOxPh5	-8,3	6,067	2,929
4lvtAdock_BOxPh6	-8,3	3,089	1,507
4lvtAdock_BOxPh9	-8,3	3,212	1,804
4lvtAdock_BOxPhCl4	-8,3	13,489	3,657
4lvtAdock_BOxPhCl6	-8,3	14,204	2,976
4lvtAdock_BOxPhCl7	-8,3	14,108	3,146
4lvtAdock_BOxPhDMN2	-8,3	13,981	3,186
4lvtAdock_BOxPhDMN3	-8,3	13,852	2,544
4lvtAdock_BOxPhDMN3	-8,3	5,504	3,796
4lvtAdock_BOxPhOMe2	-8,3	6,912	3,4
4lvtAdock_BOxPhOMe4	-8,3	5,322	3,055
4lvtAdock_BT4	-8,2	12,61	1,965
4lvtAdock_BT4	-8,2	12,179	1,869
4lvtAdock_BT6	-8,2	11,769	2,177
4lvtAdock_BT7	-8,2	14,279	3,949
4lvtAdock_BTz6	-8,2	0	0
4lvtAdock_BTzNaf4	-8,2	14,246	1,945
4lvtAdock_BTzNaf4	-8,2	10,896	8,374
4lvtAdock_BTzNaf8	-8,2	14,51	2,752
4lvtAdock_Btz1	-8,2	0	0
4lvtAdock_BtzPh2	-8,2	12,965	2,732
4lvtAdock_BtzPh2	-8,2	2,036	1,616
4lvtAdock_BtzPh2	-8,2	3,174	2,018
4lvtAdock_BtzPh5	-8,2	14,613	3,568
4lvtAdock_BtzPh5	-8,2	14,159	3,653
4lvtAdock_BtzPh6	-8,2	4,574	2,086
4lvtAdock_BtzPhCl6	-8,2	2,958	2,174
4lvtAdock_BtzPhDMN1	-8,2	13,812	2,67
4lvtAdock_BtzPhDMN2	-8,2	0	0
4lvtAdock_BtzPhDMN2	-8,2	3,639	2,04
4lvtAdock_BtzPhDMN4	-8,2	3,793	2,091
4lvtAdock_BtzPhDMN4	-8,2	3,766	2,432
4lvtAdock_BtzPhDMN4	-8,2	13,662	2,499
4lvtAdock_BtzPhOMe4	-8,2	13,261	1,901
4lvtAdock_BtzPhOMe4	-8,2	4,527	1,846
4lvtAdock_BtzPhOMe4	-8,2	13,592	2,173
4lvtAdock_BOx2	-8,2	2,28	1,861
4lvtAdock_BOx4	-8,2	2,03	1,499

4lvtAdock_BOxNaf2	-8,2	4,419	2,405
4lvtAdock_BOxNaf6	-8,2	3,788	2,598
4lvtAdock_BOxPh1	-8,2	3,974	2,236
4lvtAdock_BOxPh4	-8,2	13,631	2,328
4lvtAdock_BOxPh5	-8,2	3,183	1,914
4lvtAdock_BOxPh6	-8,2	2,112	1,469
4lvtAdock_BOxPh6	-8,2	3,136	1,732
4lvtAdock_BOxPhCl3	-8,2	14,2	3,384
4lvtAdock_BOxPhCl4	-8,2	13,249	2,892
4lvtAdock_BOxPhCl5	-8,2	14,086	2,665
4lvtAdock_BOxPhCl7	-8,2	14,385	2,173
4lvtAdock_BOxPhDMN3	-8,2	2,99	2,51
4lvtAdock_BOxPhDMN4	-8,2	9,226	3,802
4lvtAdock_BOxPhDMN4	-8,2	3,192	2,271
4lvtAdock_BOxPhDMN8	-8,2	0	0
4lvtAdock_BOxPhOMe2	-8,2	6,997	3,508
4lvtAdock_BOxPhOMe5	-8,2	6,144	3,502
4lvtAdock_BOxPhOMe5	-8,2	4,682	2,971
4lvtAdock_BOxPhOMe6	-8,2	15,246	2,326
4lvtAdock_BT6	-8,1	12,422	2,719
4lvtAdock_BT7	-8,1	4,265	2,019
4lvtAdock_BT9	-8,1	2,154	1,543
4lvtAdock_BT9	-8,1	13,754	2,527
4lvtAdock_BTz5	-8,1	12,359	2,678
4lvtAdock_BTz6	-8,1	12,499	3,954
4lvtAdock_BTzNaf2	-8,1	7,016	4,514
4lvtAdock_BTzNaf2	-8,1	7,194	4,55
4lvtAdock_BTzNaf2	-8,1	11,234	7,41
4lvtAdock_BTzNaf6	-8,1	13,731	2,802
4lvtAdock_BTzNaf6	-8,1	14,068	2,326
4lvtAdock_BTzNaf8	-8,1	3,276	2,03
4lvtAdock_BtzPh5	-8,1	14,784	3,854
4lvtAdock_BtzPh6	-8,1	13,734	3,086
4lvtAdock_BtzPh6	-8,1	13,891	3,468
4lvtAdock_BtzPh7	-8,1	13,426	1,509
4lvtAdock_BtzPhCl3	-8,1	4,7	2,836
4lvtAdock_BtzPhCl5	-8,1	13,228	2,143
4lvtAdock_BtzPhCl5	-8,1	2,077	1,697
4lvtAdock_BtzPhDMN4	-8,1	13,346	2,268
4lvtAdock_BtzPhDMN5	-8,1	0	0
4lvtAdock_BtzPhDMN9	-8,1	0	0
4lvtAdock_BtzPhOMe3	-8,1	0	0
4lvtAdock_BtzPhOMe4	-8,1	3,871	2,358
4lvtAdock_BtzPhOMe4	-8,1	4,058	1,848
4lvtAdock_BtzPhOMe4	-8,1	3,435	1,679
4lvtAdock_BtzPhOMe6	-8,1	0	0

4lvtAdock_BOx1	-8,1	10,721	3,623
4lvtAdock_BOx6	-8,1	0	0
4lvtAdock_BOxNaf3	-8,1	13,133	3,001
4lvtAdock_BOxPh5	-8,1	12,854	2,833
4lvtAdock_BOxPh6	-8,1	1,821	1,375
4lvtAdock_BOxPh9	-8,1	2,937	2,133
4lvtAdock_BOxPhCl3	-8,1	6,118	3,234
4lvtAdock_BOxPhCl3	-8,1	13,207	2,431
4lvtAdock_BOxPhCl5	-8,1	14,523	3,46
4lvtAdock_BOxPhCl6	-8,1	4,189	2,157
4lvtAdock_BOxPhDMN3	-8,1	13,733	2,86
4lvtAdock_BOxPhDMN3	-8,1	4,39	2,114
4lvtAdock_BOxPhDMN3	-8,1	6,812	3,581
4lvtAdock_BOxPhDMN4	-8,1	14,516	3,051
4lvtAdock_BOxPhDMN4	-8,1	15,706	4,193
4lvtAdock_BOxPhDMN6	-8,1	0	0
4lvtAdock_BOxPhDMN6	-8,1	2,356	1,678
4lvtAdock_BOxPhOMe3	-8,1	13,948	2,103
4lvtAdock_BOxPhOMe6	-8,1	15,203	2,75
4lvtAdock_BT6	-8	6,169	2,882
4lvtAdock_BT9	-8	13,4	2,466
4lvtAdock_BTz3	-8	11,509	3,559
4lvtAdock_BTz4	-8	0	0
4lvtAdock_BTz5	-8	12,774	2,522
4lvtAdock_BTz6	-8	11,706	1,995
4lvtAdock_BTzNaf6	-8	3,357	1,796
4lvtAdock_BTzNaf9	-8	0	0
4lvtAdock_Btz1	-8	2,545	2,046
4lvtAdock_BtzPh9	-8	0	0
4lvtAdock_BtzPhCl2	-8	4,061	2,389
4lvtAdock_BtzPhCl5	-8	4,544	2,191
4lvtAdock_BtzPhCl9	-8	0	0
4lvtAdock_BtzPhOMe8	-8	0	0
4lvtAdock_BOx3	-8	0	0
4lvtAdock_BOx4	-8	5,441	2,53
4lvtAdock_BOx4	-8	13,252	3,316
4lvtAdock_BOxPhCl4	-8	4,372	2,75
4lvtAdock_BOxPhCl4	-8	2,559	2,148
4lvtAdock_BOxPhCl5	-8	4,724	2,546
4lvtAdock_BOxPhCl7	-8	14,933	3,739
4lvtAdock_BOxPhDMN3	-8	6,382	2,799
4lvtAdock_BOxPhDMN4	-8	8,849	3,52
4lvtAdock_BOxPhDMN4	-8	15,551	3,874
4lvtAdock_BOxPhDMN5	-8	0	0
4lvtAdock_BOxPhDMN6	-8	3,535	1,785
4lvtAdock_BOxPhDMN6	-8	5,28	2,315

4lvtAdock_BOxPhOMe2	-8	7,115	3,979
4lvtAdock_BOxPhOMe2	-8	2,669	2,15
4lvtAdock_BOxPhOMe5	-8	4,534	2,627
4lvtAdock_BOxPhOMe6	-8	15,491	2,952
4lvtAdock_BOxPhOMe6	-8	15,502	2,244
4lvtAdock_BT6	-7,9	5,959	2,73
4lvtAdock_BT8	-7,9	13,271	2,194
4lvtAdock_BT8	-7,9	12,292	1,919
4lvtAdock_BTz5	-7,9	11,81	2,462
4lvtAdock_BTz5	-7,9	12,045	2,321
4lvtAdock_BTz8	-7,9	0	0
4lvtAdock_Btz7	-7,9	12,039	1,874
4lvtAdock_Btz7	-7,9	5,293	2,303
4lvtAdock_Btz7	-7,9	12,976	2,656
4lvtAdock_BtzPh6	-7,9	3,78	2,421
4lvtAdock_BtzPh7	-7,9	13,245	1,948
4lvtAdock_BtzPh7	-7,9	6,364	2,803
4lvtAdock_BtzPh7	-7,9	13,037	2,171
4lvtAdock_BtzPhCl2	-7,9	5,952	3,309
4lvtAdock_BtzPhCl2	-7,9	13,348	2,377
4lvtAdock_BtzPhCl3	-7,9	6,134	3,254
4lvtAdock_BtzPhCl4	-7,9	4,654	2,44
4lvtAdock_BtzPhCl5	-7,9	14,498	2,342
4lvtAdock_BtzPhCl6	-7,9	4,122	1,878
4lvtAdock_BtzPhCl6	-7,9	14,804	1,864
4lvtAdock_BtzPhDMN1	-7,9	13,551	2,102
4lvtAdock_BtzPhDMN2	-7,9	14,851	3,77
4lvtAdock_BtzPhDMN2	-7,9	5,017	2,58
4lvtAdock_BtzPhDMN3	-7,9	4,349	2,296
4lvtAdock_BtzPhDMN4	-7,9	14,009	2,956
4lvtAdock_BtzPhDMN4	-7,9	6,679	3,032
4lvtAdock_BtzPhDMN5	-7,9	3,534	2,094
4lvtAdock_BtzPhOMe1	-7,9	5,258	3
4lvtAdock_BtzPhOMe3	-7,9	12,94	2,022
4lvtAdock_BOx7	-7,9	0	0
4lvtAdock_BOxNaf8	-7,9	3,486	1,921
4lvtAdock_BOxNaf8	-7,9	15,45	3,718
4lvtAdock_BOxNaf8	-7,9	15,741	4,313
4lvtAdock_BOxPh5	-7,9	13,496	3,124
4lvtAdock_BOxPh8	-7,9	0	0
4lvtAdock_BOxPh9	-7,9	2,341	1,765
4lvtAdock_BOxPhCl4	-7,9	13,532	3,506
4lvtAdock_BOxPhCl5	-7,9	14,146	2,967
4lvtAdock_BOxPhCl7	-7,9	3,582	2,135
4lvtAdock_BOxPhCl7	-7,9	3,301	2,07
4lvtAdock_BOxPhDMN1	-7,9	2,499	1,932

4lvtAdock_BOxPhDMN1	-7,9	4,221	3,03
4lvtAdock_BOxPhDMN1	-7,9	4,153	2,656
4lvtAdock_BOxPhDMN4	-7,9	6,796	3,883
4lvtAdock_BOxPhDMN6	-7,9	2,21	1,691
4lvtAdock_BOxPhDMN7	-7,9	0	0
4lvtAdock_BOxPhOMe2	-7,9	6,933	3,959
4lvtAdock_BOxPhOMe4	-7,9	13,67	3,228
4lvtAdock_BOxPhOMe5	-7,9	4,987	2,981
4lvtAdock_BOxPhOMe5	-7,9	16,6	3,62
4lvtAdock_BT4	-7,8	14,076	6,67
4lvtAdock_BT9	-7,8	13,455	2,89
4lvtAdock_BTz6	-7,8	12,42	3,468
4lvtAdock_BTz8	-7,8	12,797	2,307
4lvtAdock_BTzNaf7	-7,8	14,665	2,42
4lvtAdock_BTzNaf8	-7,8	2,468	1,502
4lvtAdock_BtzPh6	-7,8	13,303	3,555
4lvtAdock_BtzPh8	-7,8	0	0
4lvtAdock_BtzPh8	-7,8	4,292	2,062
4lvtAdock_BtzPh9	-7,8	4,952	2,562
4lvtAdock_BtzPhCl1	-7,8	13,435	8,587
4lvtAdock_BtzPhCl1	-7,8	2,675	1,482
4lvtAdock_BtzPhCl4	-7,8	4,766	2,543
4lvtAdock_BtzPhCl6	-7,8	14,999	2,152
4lvtAdock_BtzPhDMN8	-7,8	0	0
4lvtAdock_BOx4	-7,8	4,993	3,429
4lvtAdock_BOx4	-7,8	5,166	2,622
4lvtAdock_BOx5	-7,8	11,576	3,732
4lvtAdock_BOx5	-7,8	12,394	3,461
4lvtAdock_BOx6	-7,8	2,066	1,667
4lvtAdock_BOx6	-7,8	12,309	2,527
4lvtAdock_BOxNaf9	-7,8	4,987	2,133
4lvtAdock_BOxNaf9	-7,8	4,956	2,523
4lvtAdock_BOxNaf9	-7,8	15,067	2,593
4lvtAdock_BOxPh6	-7,8	14,16	2,921
4lvtAdock_BOxPh8	-7,8	3,13	1,452
4lvtAdock_BOxPhCl4	-7,8	5,486	2,972
4lvtAdock_BOxPhCl4	-7,8	13,514	3,189
4lvtAdock_BOxPhDMN1	-7,8	6,661	3,611
4lvtAdock_BOxPhDMN2	-7,8	5,839	2,875
4lvtAdock_BOxPhDMN3	-7,8	7,188	3,844
4lvtAdock_BOxPhDMN6	-7,8	5,216	2,73
4lvtAdock_BOxPhDMN8	-7,8	3,866	1,854
4lvtAdock_BOxPhOMe3	-7,8	4,583	2,048
4lvtAdock_BOxPhOMe3	-7,8	6,226	2,821
4lvtAdock_BOxPhOMe5	-7,8	6,173	2,887
4lvtAdock_BOxPhOMe6	-7,8	15,161	2,577

4lvtAdock_BOxPhOMe6	-7,8	15,422	2,738
4lvtAdock_BOxPhOMe6	-7,8	4,63	1,885
4lvtAdock_BA	-7,7	0	0
4lvtAdock_BA	-7,7	9,316	5,423
4lvtAdock_BT9	-7,7	15,217	3,394
4lvtAdock_BTz4	-7,7	11,778	2,49
4lvtAdock_BTz4	-7,7	11,425	2,133
4lvtAdock_BTz6	-7,7	2,164	1,572
4lvtAdock_BTzNaf5	-7,7	3,307	2,485
4lvtAdock_BTzNaf6	-7,7	3,003	1,78
4lvtAdock_BTzNaf8	-7,7	14,808	3,095
4lvtAdock_BTzNaf8	-7,7	14,377	2,92
4lvtAdock_Btz1	-7,7	10,6	2,802
4lvtAdock_Btz2	-7,7	2,608	1,662
4lvtAdock_BtzPh2	-7,7	13,395	2,451
4lvtAdock_BtzPh6	-7,7	6,319	2,97
4lvtAdock_BtzPh7	-7,7	13,24	2,088
4lvtAdock_BtzPh7	-7,7	2,907	1,579
4lvtAdock_BtzPhCl1	-7,7	4,501	2,649
4lvtAdock_BtzPhDMN3	-7,7	4,878	2,318
4lvtAdock_BtzPhDMN3	-7,7	14,22	1,754
4lvtAdock_BtzPhDMN5	-7,7	3,607	2,054
4lvtAdock_BtzPhDMN5	-7,7	14,887	4,669
4lvtAdock_BtzPhDMN6	-7,7	0	0
4lvtAdock_BtzPhOMe3	-7,7	2,392	1,647
4lvtAdock_BtzPhOMe6	-7,7	5,077	2,164
4lvtAdock_BtzPhOMe9	-7,7	0	0
4lvtAdock_BOx1	-7,7	11,562	2,783
4lvtAdock_BOx3	-7,7	11,179	2,75
4lvtAdock_BOx4	-7,7	15,209	4,465
4lvtAdock_BOx7	-7,7	11,95	2,016
4lvtAdock_BOx7	-7,7	3,868	2,109
4lvtAdock_BOx7	-7,7	4,196	2,401
4lvtAdock_BOx9	-7,7	0	0
4lvtAdock_BOxNaf9	-7,7	14,078	2,395
4lvtAdock_BOxPh1	-7,7	9,889	5,964
4lvtAdock_BOxPh7	-7,7	0	0
4lvtAdock_BOxPh8	-7,7	13,843	1,989
4lvtAdock_BOxPh9	-7,7	0	0
4lvtAdock_BOxPhCl7	-7,7	3,399	2,485
4lvtAdock_BOxPhCl9	-7,7	0	0
4lvtAdock_BOxPhDMN2	-7,7	6,049	3,017
4lvtAdock_BOxPhDMN6	-7,7	3,592	2,354
4lvtAdock_BOxPhOMe1	-7,7	2,686	1,631
4lvtAdock_BOxPhOMe3	-7,7	4,33	2,391
4lvtAdock_BOxPhOMe8	-7,7	0	0

4lvtAdock_BT6	-7,6	12,28	2,364
4lvtAdock_BT7	-7,6	4,703	2,065
4lvtAdock_BTz3	-7,6	5,582	3,005
4lvtAdock_BTz5	-7,6	12,406	2,83
4lvtAdock_BTzNaf6	-7,6	5,97	4,088
4lvtAdock_BTzNaf7	-7,6	14,428	1,558
4lvtAdock_BTzNaf9	-7,6	12,739	6,563
4lvtAdock_Btz2	-7,6	11,098	1,899
4lvtAdock_Btz7	-7,6	2,125	1,413
4lvtAdock_BtzPh8	-7,6	3,075	1,559
4lvtAdock_BtzPh9	-7,6	4,108	2,314
4lvtAdock_BtzPhCl5	-7,6	13,893	1,942
4lvtAdock_BtzPhCl6	-7,6	13,866	2,167
4lvtAdock_BtzPhCl6	-7,6	14,953	1,749
4lvtAdock_BtzPhCl7	-7,6	0	0
4lvtAdock_BtzPhDMN1	-7,6	12,971	2,715
4lvtAdock_BtzPhDMN2	-7,6	4,939	2,62
4lvtAdock_BtzPhDMN3	-7,6	13,643	5,892
4lvtAdock_BtzPhDMN5	-7,6	3,316	1,59
4lvtAdock_BtzPhOMe1	-7,6	6,216	3,042
4lvtAdock_BtzPhOMe4	-7,6	5,039	2,349
4lvtAdock_BtzPhOMe6	-7,6	14,821	2,161
4lvtAdock_BtzPhOMe6	-7,6	14,593	1,901
4lvtAdock_BtzPhOMe8	-7,6	3,75	1,641
4lvtAdock_BtzPhOMe9	-7,6	4,975	3,401
4lvtAdock_BOx1	-7,6	1,939	1,679
4lvtAdock_BOx1	-7,6	11,242	3,323
4lvtAdock_BOx2	-7,6	2,809	2,158
4lvtAdock_BOx9	-7,6	2,596	2,036
4lvtAdock_BOxNaf7	-7,6	4,084	1,745
4lvtAdock_BOxNaf8	-7,6	3,592	1,993
4lvtAdock_BOxNaf9	-7,6	15,084	2,657
4lvtAdock_BOxPhCl7	-7,6	14,402	2,661
4lvtAdock_BOxPhDMN5	-7,6	14,208	1,789
4lvtAdock_BOxPhDMN6	-7,6	15,612	2,215
4lvtAdock_BOxPhDMN8	-7,6	15,017	2,444
4lvtAdock_BOxPhOMe1	-7,6	13,105	2,281
4lvtAdock_BT7	-7,5	13,952	4,153
4lvtAdock_BT9	-7,5	8,369	3,889
4lvtAdock_BTz3	-7,5	11,843	2,706
4lvtAdock_BTz6	-7,5	12,273	2,154
4lvtAdock_BTz8	-7,5	12,473	2,492
4lvtAdock_BTzNaf7	-7,5	15,092	1,538
4lvtAdock_Bet	-7,5	0	0
4lvtAdock_Bet	-7,5	10,272	5,081
4lvtAdock_BtzPh6	-7,5	6	2,983

4lvtAdock_BtzPh8	-7,5	4,802	2,275
4lvtAdock_BtzPhCl5	-7,5	4,68	2,586
4lvtAdock_BtzPhCl6	-7,5	14,811	1,877
4lvtAdock_BtzPhCl6	-7,5	4,189	2,076
4lvtAdock_BtzPhCl7	-7,5	3,684	2,196
4lvtAdock_BtzPhCl9	-7,5	7,249	2,438
4lvtAdock_BtzPhDMN1	-7,5	2,847	1,995
4lvtAdock_BtzPhDMN9	-7,5	2,383	1,739
4lvtAdock_BtzPhOMe2	-7,5	2,356	1,758
4lvtAdock_BtzPhOMe4	-7,5	4,668	2,227
4lvtAdock_BtzPhOMe8	-7,5	5,37	2,167
4lvtAdock_BtzPhOMe9	-7,5	4,753	2,258
4lvtAdock_BOx1	-7,5	5,444	3,008
4lvtAdock_BOx3	-7,5	11,218	1,79
4lvtAdock_BOx4	-7,5	5,497	2,743
4lvtAdock_BOx5	-7,5	1,969	1,608
4lvtAdock_BOx5	-7,5	3,836	2,846
4lvtAdock_BOx6	-7,5	12,192	2,434
4lvtAdock_BOx6	-7,5	4,081	1,848
4lvtAdock_BOx9	-7,5	2,157	1,579
4lvtAdock_BOxNaf6	-7,5	15,218	2,238
4lvtAdock_BOxNaf6	-7,5	14,955	2,198
4lvtAdock_BOxPh8	-7,5	2,701	1,668
4lvtAdock_BOxPh8	-7,5	3,939	2,453
4lvtAdock_BOxPh9	-7,5	4,119	2,792
4lvtAdock_BOxPhOMe4	-7,5	14,324	3,387
4lvtAdock_BOxPhOMe4	-7,5	13,555	3,175
4lvtAdock_BOxPhOMe4	-7,5	12,755	2,38
4lvtAdock_BOxPhOMe4	-7,5	2,522	1,784
4lvtAdock_BOxPhOMe7	-7,5	0	0
4lvtAdock_BOxPhOMe7	-7,5	5,433	3,002
4lvtAdock_BOxPhOMe7	-7,5	14,027	2,592
4lvtAdock_BOxPhOMe8	-7,5	5,036	2,378
4lvtAdock_BA	-7,4	9,088	5,587
4lvtAdock_BA	-7,4	9,889	5,96
4lvtAdock_BT6	-7,4	12,928	3,155
4lvtAdock_BT7	-7,4	2,737	1,888
4lvtAdock_BTz4	-7,4	4,907	2,974
4lvtAdock_BTz8	-7,4	3,076	2,16
4lvtAdock_BTzNaf8	-7,4	3,895	2,12
4lvtAdock_BTzNaf9	-7,4	11,411	5,292
4lvtAdock_Btz2	-7,4	7,156	4,72
4lvtAdock_Btz2	-7,4	4,269	3,381
4lvtAdock_Btz7	-7,4	2,234	1,614
4lvtAdock_BtzPh8	-7,4	3,501	1,788
4lvtAdock_BtzPh8	-7,4	5,143	1,841

4lvtAdock_BtzPhCl9	-7,4	4,16	2,08
4lvtAdock_BtzPhDMN3	-7,4	12,837	7,049
4lvtAdock_BtzPhDMN4	-7,4	5,482	2,612
4lvtAdock_BtzPhDMN4	-7,4	5,023	2,274
4lvtAdock_BtzPhDMN5	-7,4	3,567	2,461
4lvtAdock_BtzPhDMN6	-7,4	2,19	1,645
4lvtAdock_BtzPhOMe6	-7,4	2,234	1,74
4lvtAdock_BtzPhOMe7	-7,4	0	0
4lvtAdock_BtzPhOMe7	-7,4	3,316	1,708
4lvtAdock_BOx2	-7,4	5,095	3,684
4lvtAdock_BOx6	-7,4	2,595	2,161
4lvtAdock_BOx7	-7,4	11,681	1,973
4lvtAdock_BOx7	-7,4	4,035	2,275
4lvtAdock_BOx9	-7,4	7,395	3,169
4lvtAdock_BOx9	-7,4	5,504	2,879
4lvtAdock_BOxNaf7	-7,4	6,022	2,38
4lvtAdock_BOxNaf7	-7,4	14,975	2,326
4lvtAdock_BOxNaf7	-7,4	14,44	2,523
4lvtAdock_BOxNaf9	-7,4	14,412	2,551
4lvtAdock_BOxPh8	-7,4	14,065	2,089
4lvtAdock_BOxPh8	-7,4	13,291	2,374
4lvtAdock_BOxPhDMN1	-7,4	4,668	2,57
4lvtAdock_BOxPhDMN5	-7,4	14,077	2,202
4lvtAdock_BOxPhDMN7	-7,4	4,244	2,169
4lvtAdock_BOxPhDMN7	-7,4	2,037	1,46
4lvtAdock_BOxPhDMN8	-7,4	14,985	3,438
4lvtAdock_BOxPhDMN8	-7,4	14,551	2,726
4lvtAdock_BOxPhDMN9	-7,4	0	0
4lvtAdock_BOxPhOMe4	-7,4	12,933	3,642
4lvtAdock_BOxPhOMe8	-7,4	4,152	2,617
4lvtAdock_BA	-7,3	9,312	4,504
4lvtAdock_BTz3	-7,3	11,772	1,733
4lvtAdock_BTz5	-7,3	11,161	2,411
4lvtAdock_BTz8	-7,3	12,464	1,906
4lvtAdock_BTz8	-7,3	4,176	2,232
4lvtAdock_BTzNaf9	-7,3	10,73	4,951
4lvtAdock_Btz1	-7,3	6,391	3,575
4lvtAdock_Btz2	-7,3	7,049	4,47
4lvtAdock_Btz2	-7,3	6,78	4,282
4lvtAdock_Btz9	-7,3	0	0
4lvtAdock_BtzPh7	-7,3	6,191	2,715
4lvtAdock_BtzPh8	-7,3	6,954	2,849
4lvtAdock_BtzPhCl5	-7,3	14,027	2,68
4lvtAdock_BtzPhCl7	-7,3	13,801	3,037
4lvtAdock_BtzPhCl7	-7,3	13,906	2,943
4lvtAdock_BtzPhDMN3	-7,3	14,217	2,16

4lvtAdock_BtzPhDMN6	-7,3	2,119	1,538
4lvtAdock_BtzPhDMN7	-7,3	0	0
4lvtAdock_BtzPhDMN7	-7,3	14,093	2,455
4lvtAdock_BtzPhDMN9	-7,3	2,93	2,168
4lvtAdock_BtzPhOMe2	-7,3	8,993	5,636
4lvtAdock_BtzPhOMe3	-7,3	11,121	6,376
4lvtAdock_BtzPhOMe6	-7,3	4,755	2,5
4lvtAdock_BtzPhOMe8	-7,3	5,885	2,165
4lvtAdock_BtzPhOMe9	-7,3	15,735	3,12
4lvtAdock_BOx1	-7,3	11,103	3,32
4lvtAdock_BOx5	-7,3	12,415	3,263
4lvtAdock_BOx7	-7,3	12,113	2,012
4lvtAdock_BOx8	-7,3	0	0
4lvtAdock_BOx9	-7,3	12,686	2,675
4lvtAdock_BOxPh7	-7,3	5,138	2,434
4lvtAdock_BOxPh8	-7,3	5,193	2,589
4lvtAdock_BOxPhDMN1	-7,3	7,169	3,85
4lvtAdock_BOxPhDMN2	-7,3	2,707	2,166
4lvtAdock_BOxPhDMN2	-7,3	4,553	2,45
4lvtAdock_BOxPhDMN2	-7,3	4,055	2,251
4lvtAdock_BOxPhDMN6	-7,3	5,289	2,882
4lvtAdock_BOxPhDMN7	-7,3	5,044	2,808
4lvtAdock_BOxPhOMe8	-7,3	3,557	2,155
4lvtAdock_BOxPhOMe8	-7,3	14,088	3,211
4lvtAdock_BA	-7,2	8,017	1,647
4lvtAdock_BA	-7,2	7,996	3,949
4lvtAdock_BTz3	-7,2	3,1	2,279
4lvtAdock_BTz5	-7,2	3,251	2,304
4lvtAdock_BTz6	-7,2	11,814	1,42
4lvtAdock_BTz8	-7,2	4,039	2,173
4lvtAdock_BTzNaf9	-7,2	12,831	6,758
4lvtAdock_Bet	-7,2	11,6	6,541
4lvtAdock_Btz1	-7,2	6,267	3,915
4lvtAdock_Btz1	-7,2	2,577	2,141
4lvtAdock_Btz7	-7,2	11,779	2,264
4lvtAdock_BtzPh7	-7,2	12,793	2,439
4lvtAdock_BtzPhCl7	-7,2	5,642	2,993
4lvtAdock_BtzPhCl7	-7,2	4,954	2,715
4lvtAdock_BtzPhCl8	-7,2	0	0
4lvtAdock_BtzPhCl9	-7,2	7,413	3,378
4lvtAdock_BtzPhDMN6	-7,2	14,949	2,082
4lvtAdock_BtzPhDMN6	-7,2	14,774	2,029
4lvtAdock_BtzPhOMe2	-7,2	8,039	3,885
4lvtAdock_BtzPhOMe3	-7,2	12,799	2,947
4lvtAdock_BtzPhOMe5	-7,2	13,543	3,57
4lvtAdock_BtzPhOMe6	-7,2	14,737	2,271

4lvtAdock_BtzPhOMe6	-7,2	3,735	2,4
4lvtAdock_BtzPhOMe6	-7,2	5,423	2,855
4lvtAdock_BtzPhOMe7	-7,2	13,787	2,462
4lvtAdock_BOx1	-7,2	13,379	4,051
4lvtAdock_BOx3	-7,2	2,18	1,745
4lvtAdock_BOx6	-7,2	2,101	1,711
4lvtAdock_BOx6	-7,2	5,021	2,247
4lvtAdock_BOx6	-7,2	3,849	1,935
4lvtAdock_BOx7	-7,2	5,162	2,904
4lvtAdock_BOx8	-7,2	4,574	2,27
4lvtAdock_BOx9	-7,2	7,107	3,419
4lvtAdock_BOxNaf8	-7,2	2,04	1,38
4lvtAdock_BOxPh7	-7,2	2,424	1,799
4lvtAdock_BOxPh9	-7,2	15,41	3,089
4lvtAdock_BOxPh9	-7,2	15,506	3,258
4lvtAdock_BOxPh9	-7,2	4,108	2,621
4lvtAdock_BOxPhCl8	-7,2	0	0
4lvtAdock_BOxPhCl9	-7,2	4,626	2,301
4lvtAdock_BOxPhCl9	-7,2	6,33	3,232
4lvtAdock_BOxPhCl9	-7,2	5,719	2,963
4lvtAdock_BOxPhDMN5	-7,2	13,544	2,609
4lvtAdock_BOxPhOMe3	-7,2	13,179	2,308
4lvtAdock_BOxPhOMe3	-7,2	5,816	2,892
4lvtAdock_BOxPhOMe8	-7,2	4,334	2,213
4lvtAdock_BOxPhOMe8	-7,2	6,723	3,143
4lvtAdock_BT8	-7,1	4,251	2,113
4lvtAdock_BTz3	-7,1	6,295	3,8
4lvtAdock_BTz3	-7,1	3,607	2,783
4lvtAdock_BTz4	-7,1	4,93	2,89
4lvtAdock_BTz4	-7,1	6,24	4,495
4lvtAdock_BTz5	-7,1	14,675	5,495
4lvtAdock_BTz8	-7,1	12,084	2,549
4lvtAdock_BTz8	-7,1	2,312	1,639
4lvtAdock_BTzNaf5	-7,1	16,313	4,384
4lvtAdock_BTzNaf7	-7,1	14,69	2,093
4lvtAdock_Bet	-7,1	9,807	4,24
4lvtAdock_Bet	-7,1	7,105	3,84
4lvtAdock_Btz2	-7,1	5,681	3,584
4lvtAdock_Btz7	-7,1	12,277	2,237
4lvtAdock_Btz7	-7,1	2,376	1,782
4lvtAdock_BtzPh9	-7,1	6,241	2,717
4lvtAdock_BtzPhCl8	-7,1	12,462	2,54
4lvtAdock_BtzPhDMN2	-7,1	6,275	3,986
4lvtAdock_BtzPhDMN5	-7,1	3,753	1,835
4lvtAdock_BtzPhDMN8	-7,1	14,051	2,54
4lvtAdock_BtzPhDMN8	-7,1	14,347	2,85

4lvtAdock_BtzPhDMN8	-7,1	14,221	2,972
4lvtAdock_BtzPhOMe1	-7,1	6,938	3,813
4lvtAdock_BtzPhOMe2	-7,1	4,563	2,21
4lvtAdock_BtzPhOMe3	-7,1	5,491	2,65
4lvtAdock_BtzPhOMe8	-7,1	2,123	1,552
4lvtAdock_BtzPhOMe9	-7,1	14,605	1,967
4lvtAdock_BOx2	-7,1	6,505	3,763
4lvtAdock_BOx2	-7,1	2,467	1,916
4lvtAdock_BOx5	-7,1	11,749	3,401
4lvtAdock_BOxNaf7	-7,1	14,416	2,481
4lvtAdock_BOxNaf9	-7,1	15,134	2,357
4lvtAdock_BOxNaf9	-7,1	6,182	2,464
4lvtAdock_BOxPh8	-7,1	5,101	2,42
4lvtAdock_BOxPh9	-7,1	13,711	3,015
4lvtAdock_BOxPh9	-7,1	13,951	2,687
4lvtAdock_BOxPh9	-7,1	15,514	2,932
4lvtAdock_BOxPhCl8	-7,1	4,019	1,977
4lvtAdock_BOxPhCl8	-7,1	7,21	3,059
4lvtAdock_BOxPhCl9	-7,1	16,042	3,884
4lvtAdock_BOxPhCl9	-7,1	5,612	2,946
4lvtAdock_BOxPhDMN5	-7,1	14,121	2,351
4lvtAdock_BOxPhDMN7	-7,1	3,735	1,829
4lvtAdock_BOxPhDMN8	-7,1	15,008	2,454
4lvtAdock_BOxPhOMe3	-7,1	3,986	2,306
4lvtAdock_BOxPhOMe3	-7,1	2,255	1,673
4lvtAdock_BOxPhOMe4	-7,1	11,915	3,29
4lvtAdock_BOxPhOMe8	-7,1	13,878	2,838
4lvtAdock_BOxPhOMe9	-7,1	0	0
4lvtAdock_BT7	-7	6,399	3,535
4lvtAdock_BT7	-7	2,892	2,255
4lvtAdock_BT8	-7	14,58	6,968
4lvtAdock_BT8	-7	12,801	1,958
4lvtAdock_BTz4	-7	11,534	2,339
4lvtAdock_BTz4	-7	12,321	2,977
4lvtAdock_BTz6	-7	11,513	1,994
4lvtAdock_BTzNaf7	-7	4,037	1,691
4lvtAdock_BTzNaf9	-7	11,453	5,582
4lvtAdock_Bet	-7	3,884	1,769
4lvtAdock_Btz1	-7	6,08	3,492
4lvtAdock_Btz2	-7	6,591	3,955
4lvtAdock_Btz9	-7	5,094	2,655
4lvtAdock_BtzPh9	-7	3,237	1,652
4lvtAdock_BtzPhCl7	-7	5,723	3,262
4lvtAdock_BtzPhCl8	-7	13,024	2,732
4lvtAdock_BtzPhCl8	-7	14,231	3,945
4lvtAdock_BtzPhCl8	-7	12,3	2,338

4lvtAdock_BtzPhCl9	-7	8,058	3,594
4lvtAdock_BtzPhCl9	-7	5,677	2,651
4lvtAdock_BtzPhCl9	-7	15,134	4,268
4lvtAdock_BtzPhDMN2	-7	4,919	2,52
4lvtAdock_BtzPhDMN3	-7	4,361	2,279
4lvtAdock_BtzPhDMN5	-7	15,264	2,23
4lvtAdock_BtzPhDMN5	-7	6,83	2,908
4lvtAdock_BtzPhDMN6	-7	14,659	2,538
4lvtAdock_BtzPhDMN7	-7	3,759	2,078
4lvtAdock_BtzPhOMe2	-7	8,299	5,27
4lvtAdock_BtzPhOMe3	-7	12,467	2,354
4lvtAdock_BtzPhOMe7	-7	4,612	2,602
4lvtAdock_BtzPhOMe7	-7	4,006	2,054
4lvtAdock_BtzPhOMe8	-7	5,523	2,341
4lvtAdock_BtzPhOMe9	-7	4,424	3,08
4lvtAdock_BOx1	-7	6,083	3,355
4lvtAdock_BOx2	-7	2,153	1,831
4lvtAdock_BOx2	-7	3,4	2,548
4lvtAdock_BOxNaf5	-7	4,861	2,446
4lvtAdock_BOxNaf5	-7	14,526	2,054
4lvtAdock_BOxNaf7	-7	3,344	1,908
4lvtAdock_BOxNaf7	-7	15,406	2,186
4lvtAdock_BOxNaf7	-7	14,667	1,825
4lvtAdock_BOxPh7	-7	4,197	2,19
4lvtAdock_BOxPhCl8	-7	13,135	3,307
4lvtAdock_BOxPhCl8	-7	13,641	2,526
4lvtAdock_BOxPhCl9	-7	4,172	2,774
4lvtAdock_BOxPhDMN9	-7	3,852	1,788
4lvtAdock_BOxPhOMe7	-7	3,43	2,015
4lvtAdock_BOxPhOMe8	-7	8,057	3,775
4lvtAdock_BA	-6,9	8,187	1,568
4lvtAdock_BA	-6,9	9,934	5,817
4lvtAdock_BT8	-6,9	4,711	2,452
4lvtAdock_BTzNaf8	-6,9	2,744	1,761
4lvtAdock_Bet	-6,9	1,616	1,18
4lvtAdock_Btz9	-6,9	4,546	2,44
4lvtAdock_BtzPh8	-6,9	6,789	2,964
4lvtAdock_BtzPh9	-6,9	3,691	1,876
4lvtAdock_BtzPh9	-6,9	11,381	6,568
4lvtAdock_BtzPhCl4	-6,9	4,527	2,102
4lvtAdock_BtzPhDMN2	-6,9	4,652	2,428
4lvtAdock_BtzPhDMN6	-6,9	14,368	3,069
4lvtAdock_BtzPhDMN8	-6,9	2,723	1,984
4lvtAdock_BtzPhOMe1	-6,9	12,539	7,165
4lvtAdock_BtzPhOMe2	-6,9	5,893	2,615
4lvtAdock_BtzPhOMe8	-6,9	13,638	2,95

4lvtAdock_BOx3	-6,9	10,664	1,858
4lvtAdock_BOx5	-6,9	3,677	2,477
4lvtAdock_BOx8	-6,9	12,27	2,101
4lvtAdock_BOx8	-6,9	11,996	2,6
4lvtAdock_BOx9	-6,9	11,948	2,828
4lvtAdock_BOx9	-6,9	12,582	2,756
4lvtAdock_BOxNaf5	-6,9	13,946	3,158
4lvtAdock_BOxNaf8	-6,9	3,937	2,192
4lvtAdock_BOxNaf8	-6,9	15,043	2,283
4lvtAdock_BOxPh7	-6,9	13,964	2,687
4lvtAdock_BOxPhCl9	-6,9	5,539	2,988
4lvtAdock_BOxPhCl9	-6,9	15,076	3,028
4lvtAdock_BOxPhDMN5	-6,9	8,246	3,536
4lvtAdock_BOxPhDMN5	-6,9	14,962	2,774
4lvtAdock_BOxPhDMN7	-6,9	2,537	1,966
4lvtAdock_BOxPhDMN7	-6,9	3,909	1,985
4lvtAdock_BOxPhDMN7	-6,9	7,324	3,513
4lvtAdock_BOxPhDMN8	-6,9	15,251	2,869
4lvtAdock_BOxPhOMe9	-6,9	3,348	1,884
4lvtAdock_BTz4	-6,8	5,773	3,638
4lvtAdock_BTzNaf7	-6,8	11,832	7,706
4lvtAdock_Btz9	-6,8	11,549	2,318
4lvtAdock_BtzPh8	-6,8	5,305	2,416
4lvtAdock_BtzPh9	-6,8	3,523	2,269
4lvtAdock_BtzPh9	-6,8	3,188	1,903
4lvtAdock_BtzPhCl4	-6,8	4,183	3,226
4lvtAdock_BtzPhCl4	-6,8	4,605	2,591
4lvtAdock_BtzPhCl7	-6,8	5,1	2,677
4lvtAdock_BtzPhDMN6	-6,8	14,491	1,756
4lvtAdock_BtzPhDMN6	-6,8	14,866	1,539
4lvtAdock_BtzPhDMN7	-6,8	12,48	6,764
4lvtAdock_BtzPhOMe5	-6,8	13,967	3,015
4lvtAdock_BtzPhOMe5	-6,8	14,016	2,204
4lvtAdock_BtzPhOMe8	-6,8	13,94	2,993
4lvtAdock_BtzPhOMe9	-6,8	15,176	3,059
4lvtAdock_BOx2	-6,8	5,312	3,213
4lvtAdock_BOxNaf8	-6,8	15,599	1,852
4lvtAdock_BOxPh7	-6,8	5,94	2,691
4lvtAdock_BOxPh9	-6,8	13,449	2,611
4lvtAdock_BOxPhCl8	-6,8	12,447	2,956
4lvtAdock_BOxPhDMN7	-6,8	2,254	1,648
4lvtAdock_BOxPhDMN8	-6,8	15,312	2,422
4lvtAdock_BOxPhOMe9	-6,8	5,702	3,198
4lvtAdock_BTzNaf5	-6,7	17,332	5,713
4lvtAdock_BTzNaf9	-6,7	2,755	1,937
4lvtAdock_Bet	-6,7	2,034	1,562

4lvtAdock_Btz1	-6,7	2,999	2,392
4lvtAdock_Btz9	-6,7	3,217	2,183
4lvtAdock_Btz9	-6,7	4,911	2,545
4lvtAdock_BtzPhCl7	-6,7	5,603	2,866
4lvtAdock_BtzPhDMN7	-6,7	3,756	2,029
4lvtAdock_BtzPhOMe2	-6,7	5,603	3,034
4lvtAdock_BtzPhOMe7	-6,7	4,082	2,209
4lvtAdock_BtzPhOMe7	-6,7	4,479	2,414
4lvtAdock_BtzPhOMe9	-6,7	4,234	2,095
4lvtAdock_BOx5	-6,7	2,752	2,045
4lvtAdock_BOx7	-6,7	4,343	2,622
4lvtAdock_BOxPh7	-6,7	14,718	3,632
4lvtAdock_BOxPh7	-6,7	13,695	2,418
4lvtAdock_BOxPhCl8	-6,7	6,985	3,46
4lvtAdock_BOxPhDMN5	-6,7	8,822	3,095
4lvtAdock_BOxPhDMN8	-6,7	14,621	2,69
4lvtAdock_BOxPhOMe7	-6,7	14,648	3,121
4lvtAdock_BOxPhOMe7	-6,7	4,803	2,291
4lvtAdock_BOxPhOMe9	-6,7	5,929	3,131
4lvtAdock_BTzNaf5	-6,6	3,994	2,889
4lvtAdock_Btz1	-6,6	6,527	4,106
4lvtAdock_Btz9	-6,6	5,32	4,17
4lvtAdock_BtzPhCl8	-6,6	2,69	1,904
4lvtAdock_BtzPhDMN2	-6,6	4,586	2,608
4lvtAdock_BtzPhOMe2	-6,6	16,044	7,652
4lvtAdock_BOx8	-6,6	12,099	2,366
4lvtAdock_BOxPh9	-6,6	13,779	2,878
4lvtAdock_BOxPhCl8	-6,6	13,938	2,862
4lvtAdock_BOxPhCl8	-6,6	14,351	2,644
4lvtAdock_BOxPhDMN5	-6,6	15,913	2,754
4lvtAdock_BOxPhOMe7	-6,6	5,361	2,99
4lvtAdock_BOxPhOMe7	-6,6	5,14	2,781
4lvtAdock_BTz6	-6,5	12,63	3,448
4lvtAdock_Bet	-6,5	15,111	10,904
4lvtAdock_Btz9	-6,5	5,292	2,736
4lvtAdock_BtzPhCl9	-6,5	7,376	3,078
4lvtAdock_BtzPhDMN7	-6,5	5,922	3,073
4lvtAdock_BtzPhDMN8	-6,5	6,081	2,622
4lvtAdock_BtzPhOMe5	-6,5	13,975	2,816
4lvtAdock_BtzPhOMe5	-6,5	14,633	8,237
4lvtAdock_BtzPhOMe5	-6,5	14,568	2,604
4lvtAdock_BtzPhOMe8	-6,5	3,953	2,081
4lvtAdock_BOx3	-6,5	11,288	2,055
4lvtAdock_BOx8	-6,5	13,568	3,184
4lvtAdock_BOxPhOMe7	-6,5	14,221	2,635
4lvtAdock_BTzNaf9	-6,4	11,595	4,2

4lvtAdock_BtzPhCl8	-6,4	12,822	2,585
4lvtAdock_BtzPhDMN7	-6,4	6,332	3,164
4lvtAdock_BtzPhDMN8	-6,4	2,008	1,437
4lvtAdock_BtzPhDMN9	-6,4	7,965	4,477
4lvtAdock_BtzPhOMe3	-6,4	12,957	1,716
4lvtAdock_BtzPhOMe7	-6,4	13,869	2,728
4lvtAdock_BOxPh7	-6,4	12,67	2,144
4lvtAdock_BOxPh9	-6,4	14,221	2,415
4lvtAdock_BOxPh9	-6,4	15,546	3,476
4lvtAdock_BOxPhDMN9	-6,4	5,636	2,596
4lvtAdock_BtzPhCl8	-6,3	13,682	3,137
4lvtAdock_BtzPhCl8	-6,3	12,789	3,153
4lvtAdock_BtzPhDMN8	-6,3	14,668	2,389
4lvtAdock_BtzPhOMe7	-6,3	14,411	2,431
4lvtAdock_BtzPhOMe9	-6,3	14,401	2,475
4lvtAdock_BOx3	-6,3	11,984	2,661
4lvtAdock_BOxPhDMN9	-6,3	5,78	3,321
4lvtAdock_BTzNaf9	-6,2	12,236	6,597
4lvtAdock_Btz9	-6,2	5,176	3,979
4lvtAdock_BtzPhDMN8	-6,2	7,003	3,063
4lvtAdock_BtzPhOMe5	-6,2	15,091	8,542
4lvtAdock_BOx3	-6,2	2,043	1,784
4lvtAdock_BOxPh9	-6,2	12,824	2,396
4lvtAdock_BOxPhDMN9	-6,2	5,876	2,675
4lvtAdock_BOxPhOMe9	-6,2	2,811	1,899
4lvtAdock_BtzPhCl9	-6,1	2,311	2,022
4lvtAdock_BtzPhDMN7	-6,1	12,211	7,282
4lvtAdock_BtzPhDMN7	-6,1	7,007	3,381
4lvtAdock_BtzPhDMN9	-6,1	3,836	2,822
4lvtAdock_BtzPhDMN9	-6,1	7,355	4,101
4lvtAdock_BOxPh9	-6,1	14,107	2,656
4lvtAdock_BOxPhOMe9	-6,1	8,517	3,861
4lvtAdock_BtzPhCl4	-6	12,175	7,67
4lvtAdock_BtzPhOMe3	-6	5,497	2,694
4lvtAdock_BOx3	-6	12,774	7,474
4lvtAdock_BOx8	-6	12,416	6,271
4lvtAdock_BOx8	-6	11,928	2,571
4lvtAdock_BtzPhDMN9	-5,9	7,148	4,144
4lvtAdock_BOxPhDMN9	-5,9	16,51	2,565
4lvtAdock_BOxPhDMN9	-5,9	5,233	2,243
4lvtAdock_BOxPhOMe9	-5,9	7,536	3,539
4lvtAdock_BtzPhDMN9	-5,8	2,235	1,626
4lvtAdock_BtzPhDMN9	-5,8	7,788	4,262
4lvtAdock_BOxPhOMe9	-5,8	6,693	3,212
4lvtAdock_BOxPhOMe9	-5,8	7,345	3,576
4lvtAdock_BOx8	-5,6	12,265	2,602

4lvtAdock_BOxPhDMN9	-5,5	6,436	3,63
4lvtAdock_BOxPhDMN9	-5,2	6,402	2,773

Table S4. Vina docking scores of docked ligands against Mcl-1 (5LOF)

Ligand	Binding Affinity	rmsd/ub	rmsd/lb
5LOFdock_BOxNaf1	-9,8	0	0
5LOFdock_BTzNaf1	-9,1	0	0
5LOFdock_BTzNaf1	-9	11,159	2,641
5LOFdock_BOxNaf1	-8,8	4,726	2,855
5LOFdock_BOxNaf1	-8,8	4,604	1,883
5LOFdock_BT4	-8,8	0	0
5LOFdock_BTzNaf1	-8,8	15,296	6,99
5LOFdock_BTzNaf4	-8,8	0	0
5LOFdock_5LOFlig	-8,8	0	0
5LOFdock_BOxPhCl1	-8,8	0	0
5LOFdock_BOxNaf1	-8,7	7,304	5,006
5LOFdock_BtzPhCl3	-8,7	0	0
5LOFdock_5LOFlig	-8,7	3,396	2,329
5LOFdock_BOxNaf1	-8,6	8,969	5,259
5LOFdock_BtzPhOMe1	-8,6	0	0
5LOFdock_BOxNaf3	-8,6	0	0
5LOFdock_BOxPh2	-8,6	0	0
5LOFdock_BTzNaf1	-8,5	2,066	1,217
5LOFdock_BtzPh2	-8,5	0	0
5LOFdock_BOxPhCl1	-8,5	10,547	4,655
5LOFdock_BT2	-8,4	0	0
5LOFdock_BTzNaf2	-8,4	0	0
5LOFdock_BTzNaf2	-8,4	12,168	2,344
5LOFdock_BtzPhCl3	-8,4	6,856	4,666
5LOFdock_BtzPhOMe4	-8,4	0	0
5LOFdock_BOxPhCl1	-8,4	10,311	4,436
5LOFdock_BOxNaf1	-8,3	5,127	3,294
5LOFdock_BOxNaf1	-8,3	14,089	6,797
5LOFdock_BT3	-8,3	0	0
5LOFdock_BT3	-8,3	4,517	2,181
5LOFdock_BTzNaf1	-8,3	3,523	2,036
5LOFdock_BTzNaf6	-8,3	0	0
5LOFdock_BtzPh1	-8,3	0	0
5LOFdock_BtzPhCl3	-8,3	7,203	4,792
5LOFdock_BtzPhCl4	-8,3	0	0
5LOFdock_BOxNaf2	-8,3	0	0
5LOFdock_BT1	-8,2	0	0
5LOFdock_BT2	-8,2	11,401	3,746
5LOFdock_BT2	-8,2	11,435	2,48
5LOFdock_BTzNaf1	-8,2	9,773	1,841
5LOFdock_BTzNaf2	-8,2	12,405	2,409

5LOFdock_BtzPhCl1	-8,2	0	0
5LOFdock_BtzPhCl3	-8,2	12,505	5,251
5LOFdock_BtzPhCl4	-8,2	2,956	1,997
5LOFdock_BOxPhCl1	-8,2	4,034	3,066
5LOFdock_BT1	-8,1	2,655	1,521
5LOFdock_BT2	-8,1	11,096	2,348
5LOFdock_BT2	-8,1	11,394	2,739
5LOFdock_BT3	-8,1	3,763	2,132
5LOFdock_BTzNaf1	-8,1	12,454	5,328
5LOFdock_BTzNaf2	-8,1	12,353	3,306
5LOFdock_BTzNaf3	-8,1	0	0
5LOFdock_BtzPh1	-8,1	9,469	2,876
5LOFdock_BtzPh1	-8,1	12,394	5,101
5LOFdock_BtzPhCl2	-8,1	0	0
5LOFdock_BtzPhOMe4	-8,1	3,961	2,735
5LOFdock_5LOFlig	-8,1	2,966	1,904
5LOFdock_BOxPh1	-8,1	0	0
5LOFdock_BOxNaf1	-8	9,252	5,761
5LOFdock_BT1	-8	7,318	4,155
5LOFdock_BT3	-8	4,034	1,664
5LOFdock_BTzNaf1	-8	6,707	3,527
5LOFdock_BTzNaf1	-8	5,444	3,02
5LOFdock_BtzPh1	-8	9,889	2,873
5LOFdock_BtzPh1	-8	2,255	1,639
5LOFdock_BtzPh1	-8	11,352	3,161
5LOFdock_BtzPhCl1	-8	13,485	7,423
5LOFdock_BtzPhCl2	-8	12,564	3,114
5LOFdock_5LOFlig	-8	8,469	3,616
5LOFdock_BOx1	-8	0	0
5LOFdock_BOxNaf4	-8	0	0
5LOFdock_BOxPh3	-8	0	0
5LOFdock_BOxPhCl2	-8	0	0
5LOFdock_BOxNaf1	-7,9	12,11	4,071
5LOFdock_BT1	-7,9	6,238	3,53
5LOFdock_BT1	-7,9	5,351	3,071
5LOFdock_BT1	-7,9	5,965	3,687
5LOFdock_BT1	-7,9	6,822	4,836
5LOFdock_BTzNaf7	-7,9	0	0
5LOFdock_Bet	-7,9	0	0
5LOFdock_BtzPh1	-7,9	8,855	3,087
5LOFdock_BtzPhCl2	-7,9	12,491	4,29
5LOFdock_BtzPhCl3	-7,9	14,122	5,355
5LOFdock_BtzPhCl3	-7,9	10,588	6,196
5LOFdock_BtzPhDMN1	-7,9	0	0
5LOFdock_BtzPhOMe1	-7,9	13,453	5,298
5LOFdock_BtzPhOMe4	-7,9	5,384	4,017

5LOFdock_5LOFlig	-7,9	9,019	4,251
5LOFdock_5LOFlig	-7,9	8,811	3,96
5LOFdock_5LOFlig	-7,9	8,979	3,442
5LOFdock_5LOFlig	-7,9	8,996	4,03
5LOFdock_5LOFlig	-7,9	9,035	4,216
5LOFdock_BA	-7,9	0	0
5LOFdock_BOxNaf3	-7,9	3,488	2,19
5LOFdock_BOxNaf3	-7,9	3,345	2,388
5LOFdock_BOxPh2	-7,9	10,483	4,961
5LOFdock_BOxPhCl1	-7,9	9,515	3,238
5LOFdock_BOxPhCl1	-7,9	3,429	2,436
5LOFdock_BOxPhCl2	-7,9	8,359	5,447
5LOFdock_BOxPhCl2	-7,9	6,721	4,001
5LOFdock_BOxPhOMe4	-7,9	0	0
5LOFdock_Bet	-7,9	0	0
5LOFdock_BA	-7,8	0	0
5LOFdock_BT2	-7,8	12,402	3,486
5LOFdock_BT2	-7,8	11,243	2,31
5LOFdock_BT3	-7,8	13,622	5,931
5LOFdock_BT4	-7,8	4,657	2,491
5LOFdock_BTzNaf2	-7,8	11,853	3,331
5LOFdock_BTzNaf7	-7,8	1,662	1,169
5LOFdock_BtzPh7	-7,8	0	0
5LOFdock_BtzPhCl1	-7,8	12,81	5,388
5LOFdock_BtzPhCl3	-7,8	3,763	2,773
5LOFdock_BtzPhCl4	-7,8	2,053	1,626
5LOFdock_BtzPhDMN1	-7,8	2,012	1,459
5LOFdock_BtzPhOMe1	-7,8	13,275	5,11
5LOFdock_BtzPhOMe4	-7,8	2,054	1,647
5LOFdock_BOxNaf3	-7,8	7,483	3,565
5LOFdock_BOxPh2	-7,8	10,694	2,937
5LOFdock_BOxPh2	-7,8	9,232	5,897
5LOFdock_BOxPh2	-7,8	7,33	4,16
5LOFdock_BOxPhOMe1	-7,8	0	0
5LOFdock_BA	-7,7	4,47	2,603
5LOFdock_BOxNaf2	-7,7	0	0
5LOFdock_BT4	-7,7	3,143	1,746
5LOFdock_BTzNaf2	-7,7	13,413	4,287
5LOFdock_BTzNaf4	-7,7	12,307	4,148
5LOFdock_BTzNaf4	-7,7	3,549	2,356
5LOFdock_BTzNaf5	-7,7	0	0
5LOFdock_Bet	-7,7	4,589	2,873
5LOFdock_BtzPh5	-7,7	0	0
5LOFdock_BtzPhCl1	-7,7	2,903	2,475
5LOFdock_BtzPhCl2	-7,7	12,371	2,523
5LOFdock_BtzPhCl3	-7,7	2,307	1,784

5LOFdock_BtzPhDMN1	-7,7	3,234	2,4
5LOFdock_BtzPhDMN1	-7,7	13,85	4,133
5LOFdock_BtzPhDMN1	-7,7	5,729	3,503
5LOFdock_BtzPhDMN1	-7,7	3,233	1,959
5LOFdock_BA	-7,7	4,455	2,591
5LOFdock_BOxNaf3	-7,7	2,217	1,281
5LOFdock_BOxNaf3	-7,7	9,966	3,189
5LOFdock_BOxNaf4	-7,7	9,637	3,331
5LOFdock_BOxPh1	-7,7	10,965	5,535
5LOFdock_BOxPh2	-7,7	9,763	6,478
5LOFdock_BOxPhCl1	-7,7	4,076	3,077
5LOFdock_BOxPhCl1	-7,7	6,475	3,212
5LOFdock_BOxPhCl1	-7,7	2,004	1,684
5LOFdock_BOxPhCl2	-7,7	7,477	4,797
5LOFdock_BOxPhOMe2	-7,7	0	0
5LOFdock_BOxPhOMe4	-7,7	11,674	3,324
5LOFdock_BOxPhOMe4	-7,7	6,427	3,653
5LOFdock_Bet	-7,7	4,614	2,876
5LOFdock_BT3	-7,6	2,099	1,356
5LOFdock_BT4	-7,6	11,429	1,68
5LOFdock_BT4	-7,6	13,015	8,07
5LOFdock_BT5	-7,6	0	0
5LOFdock_BTzNaf4	-7,6	12,682	3,332
5LOFdock_BTzNaf6	-7,6	12,833	2,383
5LOFdock_BTzNaf6	-7,6	2,572	1,705
5LOFdock_BtzPhCl3	-7,6	14,525	4,809
5LOFdock_BtzPhDMN1	-7,6	12,571	2,499
5LOFdock_BtzPhOMe4	-7,6	14,262	3,855
5LOFdock_BtzPhOMe4	-7,6	6,736	4,35
5LOFdock_BOxNaf1	-7,6	0	0
5LOFdock_BOxNaf2	-7,6	12,926	5,098
5LOFdock_BOxNaf2	-7,6	6,422	3,773
5LOFdock_BOxPh1	-7,6	8,973	5,001
5LOFdock_BOxPh2	-7,6	9,978	6,196
5LOFdock_BOxPhCl2	-7,6	9,878	6,555
5LOFdock_BOxPhDMN1	-7,6	0	0
5LOFdock_BOxPhOMe4	-7,6	7,132	4,401
5LOFdock_BT1	-7,5	6,724	4,36
5LOFdock_BT2	-7,5	11,416	2,781
5LOFdock_BT3	-7,5	1,807	1,435
5LOFdock_BT4	-7,5	6,311	3,562
5LOFdock_BT5	-7,5	11,404	1,664
5LOFdock_BT5	-7,5	11,149	6,306
5LOFdock_BTzNaf2	-7,5	8,784	4,754
5LOFdock_BTzNaf2	-7,5	13,666	2,523
5LOFdock_BTzNaf2	-7,5	12,207	3,282

5LOFdock_BTzNaf9	-7,5	0	0
5LOFdock_BtzPh2	-7,5	5,128	3,028
5LOFdock_BtzPh3	-7,5	0	0
5LOFdock_BtzPh5	-7,5	14,357	5,961
5LOFdock_BtzPhDMN2	-7,5	0	0
5LOFdock_BtzPhOMe2	-7,5	0	0
5LOFdock_BtzPhOMe4	-7,5	13,834	4,736
5LOFdock_BtzPhOMe4	-7,5	13,994	4,251
5LOFdock_BtzPhOMe4	-7,5	14,781	4,567
5LOFdock_BOx5	-7,5	0	0
5LOFdock_BOxNaf1	-7,5	8,528	2,797
5LOFdock_BOxNaf3	-7,5	8,923	2,771
5LOFdock_BOxPh2	-7,5	6,497	3,714
5LOFdock_BOxPh3	-7,5	6,868	3,304
5LOFdock_BOxPhCl2	-7,5	12,424	4,638
5LOFdock_BT1	-7,4	6,767	3,975
5LOFdock_BT2	-7,4	10,911	6,531
5LOFdock_BTz3	-7,4	0	0
5LOFdock_BTzNaf3	-7,4	4,611	2,411
5LOFdock_BTzNaf6	-7,4	13,807	3,566
5LOFdock_BtzPh1	-7,4	5,142	2,687
5LOFdock_BtzPh2	-7,4	7,454	4,449
5LOFdock_BtzPh2	-7,4	3,861	2,188
5LOFdock_BtzPhCl4	-7,4	14,176	4,253
5LOFdock_BA	-7,4	4,222	2,646
5LOFdock_BOxNaf1	-7,4	8,832	3,319
5LOFdock_BOxPh2	-7,4	3,495	1,763
5LOFdock_BOxPhCl2	-7,4	8,904	5,401
5LOFdock_BOxPhCl4	-7,4	0	0
5LOFdock_BOxPhOMe4	-7,4	11,311	5,408
5LOFdock_BA	-7,3	4,196	2,683
5LOFdock_BT5	-7,3	11,371	3,321
5LOFdock_BT6	-7,3	0	0
5LOFdock_BTz3	-7,3	5,673	3,727
5LOFdock_BTzNaf5	-7,3	12,584	2,374
5LOFdock_BTzNaf7	-7,3	12,133	2,717
5LOFdock_BtzPh1	-7,3	13,003	5,604
5LOFdock_BtzPhCl1	-7,3	10,847	3,056
5LOFdock_BtzPhOMe2	-7,3	4,67	2,776
5LOFdock_BtzPhOMe3	-7,3	0	0
5LOFdock_BOxNaf2	-7,3	6,239	3,011
5LOFdock_BOxNaf3	-7,3	8,549	5,076
5LOFdock_BOxNaf4	-7,3	10,281	3,703
5LOFdock_BOxNaf8	-7,3	0	0
5LOFdock_BOxPh1	-7,3	9,98	4,953
5LOFdock_BOxPh1	-7,3	11,698	4,986

5LOFdock_BOxPh3	-7,3	6,056	3,236
5LOFdock_BOxPh4	-7,3	0	0
5LOFdock_BOxPhCl2	-7,3	10,463	4,812
5LOFdock_BOxPhCl2	-7,3	10,294	6,545
5LOFdock_BOxPhCl3	-7,3	0	0
5LOFdock_BOxPhDMN1	-7,3	7,847	3,338
5LOFdock_BOxPhDMN1	-7,3	11,002	4,491
5LOFdock_BOxPhOMe2	-7,3	8,994	4,797
5LOFdock_BOxPhOMe2	-7,3	9,801	5,913
5LOFdock_BOxPhOMe4	-7,3	8,658	4,623
5LOFdock_BOxPhOMe4	-7,3	5,297	3,484
5LOFdock_BT3	-7,2	5,616	3,211
5LOFdock_BT6	-7,2	13,814	5,11
5LOFdock_BTz3	-7,2	2,465	2,087
5LOFdock_BTzNaf4	-7,2	7,508	4,744
5LOFdock_BTzNaf7	-7,2	2,746	1,843
5LOFdock_Btz1	-7,2	0	0
5LOFdock_Btz1	-7,2	5,954	4,167
5LOFdock_BtzPh4	-7,2	0	0
5LOFdock_BtzPh7	-7,2	2,789	1,91
5LOFdock_BtzPh7	-7,2	1,803	1,41
5LOFdock_BtzPhCl1	-7,2	10,288	2,446
5LOFdock_BtzPhCl5	-7,2	0	0
5LOFdock_BtzPhDMN4	-7,2	0	0
5LOFdock_BtzPhOMe1	-7,2	4,485	2,037
5LOFdock_BtzPhOMe2	-7,2	3,038	2,034
5LOFdock_BOx1	-7,2	3,071	2,593
5LOFdock_BOx3	-7,2	0	0
5LOFdock_BOx5	-7,2	9,007	4,536
5LOFdock_BOxNaf1	-7,2	9,801	3,311
5LOFdock_BOxNaf2	-7,2	6,983	3,869
5LOFdock_BOxNaf4	-7,2	7,959	3,815
5LOFdock_BOxPh1	-7,2	8,413	3,591
5LOFdock_BOxPh3	-7,2	10,471	3,044
5LOFdock_BOxPh3	-7,2	6,189	3,092
5LOFdock_BOxPh3	-7,2	2,922	2,041
5LOFdock_BOxPh4	-7,2	9,946	4,318
5LOFdock_BOxPh4	-7,2	6,067	2,905
5LOFdock_BOxPhDMN1	-7,2	4,444	2,235
5LOFdock_BOxPhDMN3	-7,2	0	0
5LOFdock_BOxPhOMe1	-7,2	2,251	1,723
5LOFdock_BOxPhOMe2	-7,2	4,543	2,588
5LOFdock_BOxPhOMe3	-7,2	0	0
5LOFdock_BOxPhOMe4	-7,2	6,91	4,004
5LOFdock_BOxNaf2	-7,1	13,947	6,073
5LOFdock_BT4	-7,1	15,919	7,7

5LOFdock_BTzNaf4	-7,1	13,293	4,007
5LOFdock_BTzNaf6	-7,1	2,731	1,732
5LOFdock_BTzNaf8	-7,1	0	0
5LOFdock_BtzPh4	-7,1	4,604	2,357
5LOFdock_BtzPh5	-7,1	13,165	5,449
5LOFdock_BtzPh5	-7,1	13,344	5,091
5LOFdock_BtzPh5	-7,1	11,887	4,807
5LOFdock_BtzPh7	-7,1	15,718	6,291
5LOFdock_BtzPhCl2	-7,1	15,206	7,486
5LOFdock_BtzPhCl4	-7,1	13,002	3,741
5LOFdock_BtzPhCl4	-7,1	2,853	2,099
5LOFdock_BtzPhCl4	-7,1	3,875	3,131
5LOFdock_BtzPhCl4	-7,1	12,816	4,786
5LOFdock_BtzPhCl4	-7,1	13,71	4,394
5LOFdock_BtzPhCl5	-7,1	14,321	9,487
5LOFdock_BtzPhDMN2	-7,1	7,493	3,279
5LOFdock_BtzPhDMN3	-7,1	0	0
5LOFdock_BtzPhOMe1	-7,1	13,112	5,94
5LOFdock_BtzPhOMe1	-7,1	2,802	2,26
5LOFdock_BOx3	-7,1	8,959	4,906
5LOFdock_BOx5	-7,1	8,722	4,728
5LOFdock_BOxNaf1	-7,1	9,372	3,112
5LOFdock_BOxNaf2	-7,1	7,84	3,648
5LOFdock_BOxNaf2	-7,1	8,823	4,165
5LOFdock_BOxNaf3	-7,1	9,582	3,297
5LOFdock_BOxNaf7	-7,1	0	0
5LOFdock_BOxPh1	-7,1	10,342	4,711
5LOFdock_BOxPh1	-7,1	8,274	3,99
5LOFdock_BOxPh1	-7,1	8,292	3,183
5LOFdock_BOxPh3	-7,1	10,688	3,244
5LOFdock_BOxPh4	-7,1	10,5	3,438
5LOFdock_BOxPhCl4	-7,1	10,318	5,843
5LOFdock_BOxPhCl4	-7,1	10,462	3,963
5LOFdock_BOxPhCl4	-7,1	11,275	3,24
5LOFdock_BOxPhDMN2	-7,1	0	0
5LOFdock_BOxPhDMN3	-7,1	4,433	2,237
5LOFdock_BOxPhOMe1	-7,1	9,472	5,247
5LOFdock_BOxPhOMe2	-7,1	7,5	4,092
5LOFdock_BOxPhOMe4	-7,1	9,42	4,54
5LOFdock_BOxNaf2	-7	11,266	2,502
5LOFdock_BT3	-7	4,054	2,329
5LOFdock_BT5	-7	11,565	2,753
5LOFdock_BT7	-7	0	0
5LOFdock_BT7	-7	3,847	1,757
5LOFdock_BT8	-7	0	0
5LOFdock_BTzNaf3	-7	7,431	4,808

5LOFdock_BTzNaf3	-7	6,701	4,488
5LOFdock_BTzNaf4	-7	11,551	3,183
5LOFdock_BTzNaf4	-7	12,741	3,925
5LOFdock_BTzNaf5	-7	3,031	1,994
5LOFdock_BTzNaf7	-7	14,044	6,072
5LOFdock_BTzNaf8	-7	2,307	1,7
5LOFdock_Btz1	-7	5,972	4,27
5LOFdock_BtzPh2	-7	7,08	4,109
5LOFdock_BtzPh3	-7	14,054	6,54
5LOFdock_BtzPh3	-7	2,035	1,608
5LOFdock_BtzPh4	-7	4,458	2,071
5LOFdock_BtzPh5	-7	13,221	4,75
5LOFdock_BtzPhCl5	-7	2,046	1,631
5LOFdock_BtzPhCl5	-7	12,489	2,15
5LOFdock_BtzPhCl5	-7	12,32	2,436
5LOFdock_BA	-7	8,172	1,645
5LOFdock_BOx1	-7	2,16	1,859
5LOFdock_BOx2	-7	0	0
5LOFdock_BOx3	-7	10,113	2,807
5LOFdock_BOx5	-7	8,548	4,288
5LOFdock_BOxNaf1	-7	8,942	3,461
5LOFdock_BOxNaf1	-7	9,648	4,417
5LOFdock_BOxNaf1	-7	9,527	3,594
5LOFdock_BOxNaf1	-7	6,904	3,156
5LOFdock_BOxNaf2	-7	12,183	4,964
5LOFdock_BOxNaf2	-7	8,034	4,248
5LOFdock_BOxNaf4	-7	7,993	4,172
5LOFdock_BOxNaf4	-7	8,738	4,75
5LOFdock_BOxPh3	-7	7,919	3,763
5LOFdock_BOxPh3	-7	5,866	2,982
5LOFdock_BOxPh4	-7	3,473	2,19
5LOFdock_BOxPhDMN1	-7	7,803	3,857
5LOFdock_BOxPhDMN1	-7	6,785	4,277
5LOFdock_BOxPhDMN1	-7	2,018	1,496
5LOFdock_BOxPhDMN1	-7	9,687	4,278
5LOFdock_BOxPhDMN1	-7	9,929	4,005
5LOFdock_BOxPhDMN2	-7	5,131	3,224
5LOFdock_BOxPhDMN2	-7	11,638	4,315
5LOFdock_BOxPhDMN2	-7	12,488	2,761
5LOFdock_BOxPhOMe1	-7	10,988	4,327
5LOFdock_BOxPhOMe1	-7	9,03	5,577
5LOFdock_BOxPhOMe1	-7	2,528	1,888
5LOFdock_BOxPhOMe2	-7	8,068	4,324
5LOFdock_Bet	-7	2,538	1,787
5LOFdock_BA	-6,9	8,206	1,711
5LOFdock_BOxNaf2	-6,9	14,308	6,195

5LOFdock_BT6	-6,9	2,357	1,282
5LOFdock_BT8	-6,9	11,299	6,008
5LOFdock_BTz3	-6,9	2,39	1,942
5LOFdock_BTz3	-6,9	5,911	3,99
5LOFdock_BTzNaf3	-6,9	12,678	5,851
5LOFdock_BTzNaf6	-6,9	13,507	3,926
5LOFdock_BTzNaf7	-6,9	11,884	2,578
5LOFdock_BTzNaf9	-6,9	15,806	4,182
5LOFdock_Bet	-6,9	2,36	1,599
5LOFdock_BtzPh3	-6,9	13,96	6,218
5LOFdock_BtzPh3	-6,9	14,459	6,646
5LOFdock_BtzPh5	-6,9	13,697	5,212
5LOFdock_BtzPh5	-6,9	12,079	4,614
5LOFdock_BtzPh5	-6,9	9,302	4,883
5LOFdock_BtzPhCl1	-6,9	14,114	6,928
5LOFdock_BtzPhCl2	-6,9	12,56	3,051
5LOFdock_BtzPhCl2	-6,9	14,388	7,447
5LOFdock_BtzPhDMN2	-6,9	5,122	2,996
5LOFdock_BtzPhDMN5	-6,9	0	0
5LOFdock_BA	-6,9	3,29	2,322
5LOFdock_BOx3	-6,9	9,682	2,249
5LOFdock_BOx3	-6,9	9,52	1,461
5LOFdock_BOx3	-6,9	10,269	3,51
5LOFdock_BOx5	-6,9	9,436	4,684
5LOFdock_BOx5	-6,9	2,233	2,06
5LOFdock_BOxNaf4	-6,9	3,484	1,917
5LOFdock_BOxNaf8	-6,9	10,93	3,246
5LOFdock_BOxPhCl4	-6,9	8,02	4,819
5LOFdock_BOxPhOMe1	-6,9	8,924	4,607
5LOFdock_BOxPhOMe2	-6,9	9,527	5,021
5LOFdock_BOxPhOMe3	-6,9	2,469	1,768
5LOFdock_BOxNaf2	-6,8	14,301	6,172
5LOFdock_BT4	-6,8	13,605	5,875
5LOFdock_BT5	-6,8	7,229	3,865
5LOFdock_BT6	-6,8	13,311	5,015
5LOFdock_BT8	-6,8	11,023	5,691
5LOFdock_BTz6	-6,8	0	0
5LOFdock_BTzNaf7	-6,8	12,349	2,152
5LOFdock_BTzNaf8	-6,8	2,398	1,533
5LOFdock_BTzNaf9	-6,8	16,25	5,137
5LOFdock_Btz7	-6,8	0	0
5LOFdock_BtzPh2	-6,8	7,053	4,318
5LOFdock_BtzPh2	-6,8	6,862	3,671
5LOFdock_BtzPh3	-6,8	6,28	3,707
5LOFdock_BtzPh4	-6,8	4,567	3,079
5LOFdock_BtzPh7	-6,8	10,055	5,958

5LOFdock_BtzPhCl1	-6,8	13,34	6,214
5LOFdock_BtzPhCl1	-6,8	10,587	2,676
5LOFdock_BtzPhCl2	-6,8	9,137	5,351
5LOFdock_BtzPhCl2	-6,8	10,708	6,455
5LOFdock_BtzPhDMN1	-6,8	2,225	1,705
5LOFdock_BtzPhDMN1	-6,8	15,819	7,759
5LOFdock_BtzPhDMN3	-6,8	14,463	6,385
5LOFdock_BtzPhDMN5	-6,8	2,454	1,982
5LOFdock_BtzPhOMe1	-6,8	3,174	2,461
5LOFdock_BOx2	-6,8	6,338	3,836
5LOFdock_BOx3	-6,8	2,854	2,015
5LOFdock_BOx3	-6,8	6,305	3,06
5LOFdock_BOxNaf8	-6,8	7,319	3,694
5LOFdock_BOxPhCl4	-6,8	10,439	5,629
5LOFdock_BOxPhDMN2	-6,8	2,356	1,646
5LOFdock_BOxPhOMe2	-6,8	9,016	4,691
5LOFdock_BOxPhOMe2	-6,8	11,867	5,735
5LOFdock_Bet	-6,8	5,383	3,367
5LOFdock_BOxNaf2	-6,7	14,208	6,847
5LOFdock_BOxNaf2	-6,7	13,268	6,482
5LOFdock_BT4	-6,7	15,913	7,453
5LOFdock_BT5	-6,7	11,761	6,497
5LOFdock_BTz3	-6,7	6,657	4,021
5LOFdock_BTzNaf3	-6,7	9,726	2,511
5LOFdock_BTzNaf4	-6,7	3,95	1,803
5LOFdock_BTzNaf5	-6,7	4,22	2,514
5LOFdock_BTzNaf6	-6,7	3,609	1,564
5LOFdock_BTzNaf7	-6,7	14,653	4,624
5LOFdock_BTzNaf8	-6,7	3,215	1,511
5LOFdock_Btz7	-6,7	6,636	4,013
5LOFdock_Btz7	-6,7	5,319	2,62
5LOFdock_Btz7	-6,7	4,772	2,787
5LOFdock_BtzPh2	-6,7	13,735	8,938
5LOFdock_BtzPh3	-6,7	14,386	6,621
5LOFdock_BtzPh3	-6,7	5,239	2,861
5LOFdock_BtzPh4	-6,7	6,152	3,722
5LOFdock_BtzPh8	-6,7	0	0
5LOFdock_BtzPhCl5	-6,7	13,424	2,9
5LOFdock_BtzPhDMN2	-6,7	9,963	5,398
5LOFdock_BtzPhDMN2	-6,7	9,09	4,784
5LOFdock_BtzPhOMe2	-6,7	12,431	3,645
5LOFdock_BOx2	-6,7	5,884	3,546
5LOFdock_BOx3	-6,7	9,96	2,114
5LOFdock_BOx5	-6,7	6,014	4,599
5LOFdock_BOx5	-6,7	9,594	4,328
5LOFdock_BOx6	-6,7	0	0

5LOFdock_BOx7	-6,7	0	0
5LOFdock_BOxNaf4	-6,7	6,941	4,197
5LOFdock_BOxNaf6	-6,7	0	0
5LOFdock_BOxNaf7	-6,7	3,359	1,987
5LOFdock_BOxNaf8	-6,7	2,764	2,101
5LOFdock_BOxNaf8	-6,7	2,961	2,516
5LOFdock_BOxPh4	-6,7	2,473	1,65
5LOFdock_BOxPhCl4	-6,7	11,18	3,094
5LOFdock_BOxPhCl4	-6,7	7,344	4,582
5LOFdock_BOxPhCl4	-6,7	11,998	5,809
5LOFdock_BOxPhOMe1	-6,7	9,36	4,709
5LOFdock_BOxPhOMe3	-6,7	10,392	4,522
5LOFdock_BT5	-6,6	2,358	1,651
5LOFdock_BTz4	-6,6	0	0
5LOFdock_BTz5	-6,6	0	0
5LOFdock_BTzNaf3	-6,6	13,247	6,385
5LOFdock_BTzNaf3	-6,6	9,311	2,907
5LOFdock_BTzNaf6	-6,6	12,821	2,345
5LOFdock_BTzNaf7	-6,6	12,369	2,487
5LOFdock_BTzNaf9	-6,6	13,55	6,019
5LOFdock_Bet	-6,6	6,649	3,118
5LOFdock_Btz1	-6,6	11,286	3,813
5LOFdock_Btz7	-6,6	5,468	3,484
5LOFdock_Btz7	-6,6	3,496	2,265
5LOFdock_BtzPh2	-6,6	10,511	4,076
5LOFdock_BtzPh3	-6,6	13,035	8,767
5LOFdock_BtzPh6	-6,6	0	0
5LOFdock_BtzPh7	-6,6	13,742	6,13
5LOFdock_BtzPh7	-6,6	13	3,413
5LOFdock_BtzPhCl7	-6,6	0	0
5LOFdock_BtzPhCl7	-6,6	2,17	1,822
5LOFdock_BtzPhDMN2	-6,6	13,534	8,183
5LOFdock_BtzPhDMN3	-6,6	12,923	3,127
5LOFdock_BtzPhDMN4	-6,6	11,341	6,249
5LOFdock_BtzPhDMN6	-6,6	0	0
5LOFdock_BtzPhOMe1	-6,6	9,88	5,687
5LOFdock_BtzPhOMe2	-6,6	5,627	3,612
5LOFdock_BtzPhOMe3	-6,6	8,567	4,341
5LOFdock_BOx1	-6,6	2,813	2,264
5LOFdock_BOx2	-6,6	9,366	5,013
5LOFdock_BOx2	-6,6	8,85	4,632
5LOFdock_BOx4	-6,6	0	0
5LOFdock_BOx4	-6,6	11,172	5,1
5LOFdock_BOx5	-6,6	8,32	3,269
5LOFdock_BOxNaf4	-6,6	7,831	3,708
5LOFdock_BOxNaf6	-6,6	7,919	3,103

5LOFdock_BOxNaf6	-6,6	9,363	3,064
5LOFdock_BOxNaf7	-6,6	10,879	3,789
5LOFdock_BOxNaf8	-6,6	10,617	3,395
5LOFdock_BOxPh4	-6,6	10,847	3,709
5LOFdock_BOxPh4	-6,6	10,136	5,235
5LOFdock_BOxPh4	-6,6	6,724	3,887
5LOFdock_BOxPhCl3	-6,6	10,164	4,18
5LOFdock_BOxPhCl6	-6,6	0	0
5LOFdock_BOxPhDMN2	-6,6	1,959	1,314
5LOFdock_BOxPhDMN3	-6,6	4,99	2,525
5LOFdock_BOxPhDMN5	-6,6	0	0
5LOFdock_BOxPhDMN5	-6,6	10,703	4,415
5LOFdock_BOxPhOMe1	-6,6	10,642	4,485
5LOFdock_Bet	-6,6	6,633	3,047
5LOFdock_BOxNaf2	-6,5	5,676	4,296
5LOFdock_BOxNaf2	-6,5	16,658	8,828
5LOFdock_BTz4	-6,5	10,067	2,292
5LOFdock_BTz5	-6,5	3,875	2,709
5LOFdock_BTzNaf5	-6,5	12,515	4,232
5LOFdock_Btz2	-6,5	0	0
5LOFdock_Btz2	-6,5	9,943	2,35
5LOFdock_BtzPh6	-6,5	6,72	4,013
5LOFdock_BtzPhCl5	-6,5	11,047	6,06
5LOFdock_BtzPhCl5	-6,5	12,327	2,826
5LOFdock_BtzPhDMN3	-6,5	3,288	2,54
5LOFdock_BtzPhDMN4	-6,5	11,695	2,615
5LOFdock_BtzPhDMN4	-6,5	6,427	4,248
5LOFdock_BtzPhOMe1	-6,5	12,696	5,923
5LOFdock_BtzPhOMe2	-6,5	14,659	6,645
5LOFdock_BtzPhOMe5	-6,5	0	0
5LOFdock_BOx1	-6,5	9,734	5,512
5LOFdock_BOx4	-6,5	9,909	4,208
5LOFdock_BOxNaf8	-6,5	9,881	3,54
5LOFdock_BOxNaf8	-6,5	5,458	3,283
5LOFdock_BOxPh7	-6,5	0	0
5LOFdock_BOxPhCl3	-6,5	10,589	4,269
5LOFdock_BOxPhCl3	-6,5	9,404	3,9
5LOFdock_BOxPhDMN2	-6,5	10,822	2,989
5LOFdock_BOxPhDMN2	-6,5	11,35	4,292
5LOFdock_BOxPhDMN3	-6,5	3,21	2,058
5LOFdock_BOxPhDMN5	-6,5	9,97	4,36
5LOFdock_BOxPhOMe8	-6,5	0	0
5LOFdock_Bet	-6,5	5,853	2,68
5LOFdock_BT5	-6,4	2,06	1,569
5LOFdock_BT7	-6,4	11,788	7,284
5LOFdock_BT7	-6,4	10,69	2,867

5LOFdock_BTz3	-6,4	3,537	2,25
5LOFdock_BTz3	-6,4	5,762	3,394
5LOFdock_BTz5	-6,4	3,991	2,05
5LOFdock_BTz5	-6,4	4,849	2,765
5LOFdock_BTzNaf5	-6,4	12,849	4,058
5LOFdock_BTzNaf6	-6,4	3,549	1,943
5LOFdock_Btz1	-6,4	11,146	3,777
5LOFdock_Btz1	-6,4	5,225	3,56
5LOFdock_Btz7	-6,4	3,35	2,049
5LOFdock_BtzPh4	-6,4	7,071	4,592
5LOFdock_BtzPh6	-6,4	5,741	3,563
5LOFdock_BtzPh6	-6,4	6,829	4,439
5LOFdock_BtzPh6	-6,4	7,332	3,414
5LOFdock_BtzPh6	-6,4	6,931	3,549
5LOFdock_BtzPh7	-6,4	2,206	1,618
5LOFdock_BtzPh7	-6,4	5,701	2,817
5LOFdock_BtzPh9	-6,4	0	0
5LOFdock_BtzPhCl7	-6,4	2,994	2,01
5LOFdock_BtzPhDMN3	-6,4	12,15	4,594
5LOFdock_BtzPhDMN5	-6,4	6,665	3,213
5LOFdock_BtzPhOMe2	-6,4	4,869	2,792
5LOFdock_BtzPhOMe6	-6,4	0	0
5LOFdock_BA	-6,4	3,956	2,128
5LOFdock_BOx1	-6,4	10,114	6,391
5LOFdock_BOx2	-6,4	8,302	3,484
5LOFdock_BOx2	-6,4	6,29	3,508
5LOFdock_BOx4	-6,4	9,457	3,524
5LOFdock_BOx4	-6,4	2,247	1,655
5LOFdock_BOx7	-6,4	4,644	2,531
5LOFdock_BOx7	-6,4	9,663	2,702
5LOFdock_BOxNaf5	-6,4	0	0
5LOFdock_BOxNaf5	-6,4	2,224	1,697
5LOFdock_BOxNaf5	-6,4	9,254	2,904
5LOFdock_BOxNaf5	-6,4	9,504	3,506
5LOFdock_BOxNaf7	-6,4	7,129	2,671
5LOFdock_BOxNaf7	-6,4	8,355	2,719
5LOFdock_BOxPhCl3	-6,4	10,242	5,802
5LOFdock_BOxPhCl3	-6,4	9,623	3,813
5LOFdock_BOxPhCl5	-6,4	0	0
5LOFdock_BOxPhCl6	-6,4	8,968	3,328
5LOFdock_BOxPhCl6	-6,4	6,729	3,573
5LOFdock_BOxPhDMN2	-6,4	10,186	3,366
5LOFdock_BOxPhDMN3	-6,4	9,747	4,182
5LOFdock_BOxPhDMN8	-6,4	0	0
5LOFdock_BOxPhOMe3	-6,4	11,314	4,601
5LOFdock_BOxPhOMe3	-6,4	9,753	4,948

5LOFdock_BOxPhOMe8	-6,4	6,704	3,02
5LOFdock_BA	-6,3	3,494	2,526
5LOFdock_BT6	-6,3	14,58	5,91
5LOFdock_BT6	-6,3	12,864	4,671
5LOFdock_BT8	-6,3	12,422	3,071
5LOFdock_BTz3	-6,3	6,309	4,237
5LOFdock_BTz4	-6,3	10,051	2,218
5LOFdock_BTz5	-6,3	5,712	3,644
5LOFdock_BTz5	-6,3	3,304	1,972
5LOFdock_BTz8	-6,3	0	0
5LOFdock_BTzNaf5	-6,3	4,764	2,955
5LOFdock_BTzNaf8	-6,3	9,687	3,83
5LOFdock_BTzNaf8	-6,3	12,703	1,75
5LOFdock_BTzNaf9	-6,3	6,847	3,428
5LOFdock_Btz1	-6,3	2,469	1,97
5LOFdock_Btz1	-6,3	5,474	3,752
5LOFdock_Btz1	-6,3	5,689	4,019
5LOFdock_Btz2	-6,3	3,194	2,385
5LOFdock_Btz2	-6,3	9,224	3,467
5LOFdock_Btz2	-6,3	5,689	3,61
5LOFdock_BtzPh6	-6,3	5,407	3,042
5LOFdock_BtzPhCl5	-6,3	17,557	9,333
5LOFdock_BtzPhCl7	-6,3	12,931	4,648
5LOFdock_BtzPhDMN4	-6,3	6,213	4,07
5LOFdock_BtzPhDMN5	-6,3	14,585	5,201
5LOFdock_BtzPhOMe2	-6,3	3,997	2,416
5LOFdock_BtzPhOMe5	-6,3	16,759	7,457
5LOFdock_BtzPhOMe5	-6,3	15,361	6,718
5LOFdock_BtzPhOMe5	-6,3	12,471	4,71
5LOFdock_BtzPhOMe6	-6,3	6,079	3,836
5LOFdock_BtzPhOMe9	-6,3	0	0
5LOFdock_BtzPhOMe9	-6,3	7,258	3,119
5LOFdock_BOx2	-6,3	5,857	3,498
5LOFdock_BOx2	-6,3	7,646	5,02
5LOFdock_BOx4	-6,3	5,729	3,961
5LOFdock_BOx4	-6,3	9,051	2,942
5LOFdock_BOx6	-6,3	6,747	3,528
5LOFdock_BOxNaf5	-6,3	10,297	2,499
5LOFdock_BOxNaf6	-6,3	7,094	3,291
5LOFdock_BOxNaf7	-6,3	10,409	4,238
5LOFdock_BOxNaf8	-6,3	8,476	3,147
5LOFdock_BOxPh7	-6,3	3,372	2,128
5LOFdock_BOxPh7	-6,3	3,051	1,928
5LOFdock_BOxPh8	-6,3	0	0
5LOFdock_BOxPhDMN5	-6,3	7,829	2,916
5LOFdock_BOxPhDMN5	-6,3	8,443	3,982

5LOFdock_BOxPhDMN7	-6,3	0	0
5LOFdock_BOxPhDMN8	-6,3	4,558	2,322
5LOFdock_BOxPhOMe5	-6,3	0	0
5LOFdock_BOxPhOMe8	-6,3	2,222	1,56
5LOFdock_BTz4	-6,2	3,462	2,238
5LOFdock_BTz4	-6,2	5,358	3,251
5LOFdock_BTz8	-6,2	2,898	1,99
5LOFdock_BTzNaf3	-6,2	11,075	2,657
5LOFdock_BTzNaf5	-6,2	13,282	3,98
5LOFdock_BTzNaf8	-6,2	8,719	4,051
5LOFdock_BTzNaf9	-6,2	5,288	3,169
5LOFdock_Bet	-6,2	6,628	3,515
5LOFdock_Btz7	-6,2	15,544	7,593
5LOFdock_Btz7	-6,2	11,541	2,32
5LOFdock_Btz9	-6,2	0	0
5LOFdock_BtzPh4	-6,2	14,059	6,374
5LOFdock_BtzPh4	-6,2	12,96	5,813
5LOFdock_BtzPh9	-6,2	10,743	6,402
5LOFdock_BtzPh9	-6,2	16,787	8,593
5LOFdock_BtzPhDMN2	-6,2	12,75	7,572
5LOFdock_BtzPhDMN3	-6,2	11,531	5,744
5LOFdock_BtzPhDMN3	-6,2	14,43	5,319
5LOFdock_BtzPhDMN5	-6,2	2,166	1,598
5LOFdock_BtzPhDMN6	-6,2	2,66	2,007
5LOFdock_BtzPhDMN8	-6,2	0	0
5LOFdock_BtzPhDMN9	-6,2	0	0
5LOFdock_BtzPhOMe8	-6,2	0	0
5LOFdock_BtzPhOMe9	-6,2	7,392	4,06
5LOFdock_BOx1	-6,2	10,519	5,132
5LOFdock_BOx1	-6,2	9,677	5,839
5LOFdock_BOx4	-6,2	10,157	3,87
5LOFdock_BOx4	-6,2	2,876	2,176
5LOFdock_BOx7	-6,2	2,549	1,659
5LOFdock_BOxNaf7	-6,2	10,04	3,761
5LOFdock_BOxPh5	-6,2	0	0
5LOFdock_BOxPh9	-6,2	0	0
5LOFdock_BOxPhCl6	-6,2	8,913	4,124
5LOFdock_BOxPhCl7	-6,2	0	0
5LOFdock_BOxPhDMN4	-6,2	0	0
5LOFdock_BOxPhDMN5	-6,2	9,326	3,287
5LOFdock_BOxPhDMN5	-6,2	8,302	2,393
5LOFdock_BOxPhDMN7	-6,2	6,513	3,832
5LOFdock_BOxPhDMN7	-6,2	4,287	2,719
5LOFdock_BOxPhDMN9	-6,2	0	0
5LOFdock_BOxPhOMe7	-6,2	0	0
5LOFdock_BT6	-6,1	12,274	4,487

5LOFdock_BT7	-6,1	13,706	8,8
5LOFdock_BT7	-6,1	10,854	3,105
5LOFdock_BT8	-6,1	16,239	7,418
5LOFdock_BTz5	-6,1	5,495	3,483
5LOFdock_BTz5	-6,1	3,656	1,85
5LOFdock_BTz5	-6,1	6,695	4,153
5LOFdock_BTz8	-6,1	14,503	6,248
5LOFdock_BTz8	-6,1	6,205	3,649
5LOFdock_Bet	-6,1	7,216	3,622
5LOFdock_BtzPh4	-6,1	12,949	5,602
5LOFdock_BtzPh6	-6,1	6,603	3,41
5LOFdock_BtzPh6	-6,1	7,426	4,823
5LOFdock_BtzPh8	-6,1	10,03	5,446
5LOFdock_BtzPh8	-6,1	3,252	1,708
5LOFdock_BtzPh8	-6,1	11,09	6,365
5LOFdock_BtzPh9	-6,1	2,674	2,152
5LOFdock_BtzPhCl7	-6,1	14,445	5,865
5LOFdock_BtzPhDMN2	-6,1	14,471	6,279
5LOFdock_BtzPhDMN3	-6,1	14,764	3,877
5LOFdock_BtzPhDMN6	-6,1	5,595	2,692
5LOFdock_BtzPhDMN8	-6,1	8,15	3,722
5LOFdock_BtzPhOMe2	-6,1	4,341	3,671
5LOFdock_BtzPhOMe3	-6,1	13,96	9,128
5LOFdock_BtzPhOMe3	-6,1	15,737	7,788
5LOFdock_BtzPhOMe6	-6,1	14,316	6,135
5LOFdock_BOx1	-6,1	9,806	2,816
5LOFdock_BOx6	-6,1	6,033	3,034
5LOFdock_BOx6	-6,1	8,185	4,07
5LOFdock_BOx6	-6,1	2,404	1,604
5LOFdock_BOx6	-6,1	6,979	3,256
5LOFdock_BOx7	-6,1	7,413	3,614
5LOFdock_BOx8	-6,1	0	0
5LOFdock_BOxNaf5	-6,1	6,908	3,297
5LOFdock_BOxNaf5	-6,1	10,41	2,688
5LOFdock_BOxNaf6	-6,1	8,337	3,409
5LOFdock_BOxNaf7	-6,1	10,244	4,14
5LOFdock_BOxPh5	-6,1	8,419	4,584
5LOFdock_BOxPh5	-6,1	2,456	1,711
5LOFdock_BOxPh7	-6,1	9,447	2,685
5LOFdock_BOxPh9	-6,1	10,497	3,218
5LOFdock_BOxPhCl6	-6,1	10,859	3,54
5LOFdock_BOxPhCl6	-6,1	10,737	4,408
5LOFdock_BOxPhCl7	-6,1	8,597	1,713
5LOFdock_BOxPhDMN3	-6,1	10,193	4,92
5LOFdock_BOxPhDMN5	-6,1	9,384	4,861
5LOFdock_BOxPhDMN7	-6,1	6,073	3,325

5LOFdock_BOxPhOMe3	-6,1	8,99	3,904
5LOFdock_BOxPhOMe3	-6,1	9,421	3,675
5LOFdock_BOxPhOMe7	-6,1	8,075	3,433
5LOFdock_BOxPhOMe8	-6,1	8,304	2,166
5LOFdock_Bet	-6,1	5,996	3,657
5LOFdock_BT8	-6	11,183	3,717
5LOFdock_BTz4	-6	5,463	3,557
5LOFdock_BTz6	-6	12,718	5,642
5LOFdock_BTz8	-6	3,633	2,433
5LOFdock_BTz8	-6	5,682	2,913
5LOFdock_BTzNaf5	-6	2,438	1,761
5LOFdock_BTzNaf8	-6	13,616	1,912
5LOFdock_Bet	-6	5,976	3,78
5LOFdock_BtzPh9	-6	15,738	5,976
5LOFdock_BtzPh9	-6	12,837	2,086
5LOFdock_BtzPh9	-6	12,038	2,587
5LOFdock_BtzPhCl7	-6	7,395	4,322
5LOFdock_BtzPhCl7	-6	4,247	2,334
5LOFdock_BtzPhCl7	-6	4,027	2,142
5LOFdock_BtzPhDMN3	-6	2,349	1,889
5LOFdock_BtzPhDMN9	-6	14,31	3,902
5LOFdock_BtzPhOMe3	-6	14,03	6,404
5LOFdock_BtzPhOMe3	-6	13,666	8,985
5LOFdock_BA	-6	7,014	3,222
5LOFdock_BOx6	-6	8,279	4,445
5LOFdock_BOx6	-6	6,88	3,425
5LOFdock_BOx6	-6	7,719	4,647
5LOFdock_BOx7	-6	3,325	1,906
5LOFdock_BOx8	-6	2,27	1,74
5LOFdock_BOx8	-6	10,066	3,424
5LOFdock_BOx8	-6	7,704	3,688
5LOFdock_BOxNaf5	-6	9,899	3,116
5LOFdock_BOxNaf6	-6	10,1	2,313
5LOFdock_BOxPh6	-6	0	0
5LOFdock_BOxPh6	-6	7,15	2,747
5LOFdock_BOxPh7	-6	10,202	4,013
5LOFdock_BOxPh7	-6	3,495	2,001
5LOFdock_BOxPh9	-6	8,768	3,073
5LOFdock_BOxPhCl3	-6	5,532	2,938
5LOFdock_BOxPhCl5	-6	9,099	3,857
5LOFdock_BOxPhCl6	-6	10,091	5,496
5LOFdock_BOxPhCl7	-6	7,278	3,195
5LOFdock_BOxPhCl7	-6	7,395	3,143
5LOFdock_BOxPhDMN3	-6	10,057	4,619
5LOFdock_BOxPhDMN3	-6	8,048	4,007
5LOFdock_BOxPhDMN3	-6	10,974	4,18

5LOFdock_BOxPhDMN5	-6	8,746	4,162
5LOFdock_BOxPhDMN8	-6	7,776	3,898
5LOFdock_BOxPhDMN8	-6	3,504	1,861
5LOFdock_BOxPhOMe5	-6	11,901	5,156
5LOFdock_BOxPhOMe6	-6	0	0
5LOFdock_BOxPhOMe8	-6	8,384	2,464
5LOFdock_BA	-5,9	7,898	1,712
5LOFdock_BT6	-5,9	4,119	2,457
5LOFdock_BT6	-5,9	14,112	5,179
5LOFdock_BT7	-5,9	11,732	6,757
5LOFdock_BT7	-5,9	10,248	2,56
5LOFdock_BT8	-5,9	16,698	7,339
5LOFdock_BTz6	-5,9	4,553	2,761
5LOFdock_BTz8	-5,9	6,05	3,372
5LOFdock_Btz2	-5,9	10,074	2,781
5LOFdock_BtzPh8	-5,9	3,302	1,793
5LOFdock_BtzPh9	-5,9	13,201	7,445
5LOFdock_BtzPh9	-5,9	9,802	5,783
5LOFdock_BtzPhCl6	-5,9	0	0
5LOFdock_BtzPhCl7	-5,9	12,829	4,18
5LOFdock_BtzPhDMN2	-5,9	7,117	3,031
5LOFdock_BtzPhDMN4	-5,9	2,433	1,856
5LOFdock_BtzPhDMN4	-5,9	5,902	3,524
5LOFdock_BtzPhDMN4	-5,9	11,565	6,505
5LOFdock_BtzPhDMN4	-5,9	12,857	5,692
5LOFdock_BtzPhDMN5	-5,9	5,946	3,047
5LOFdock_BtzPhDMN6	-5,9	3,668	2,44
5LOFdock_BtzPhDMN6	-5,9	8,822	4,47
5LOFdock_BtzPhOMe3	-5,9	7,788	4,143
5LOFdock_BtzPhOMe3	-5,9	5,647	2,92
5LOFdock_BtzPhOMe6	-5,9	12,452	4,22
5LOFdock_BA	-5,9	5,37	3,452
5LOFdock_BOx7	-5,9	10,012	4,987
5LOFdock_BOx7	-5,9	6,075	3,005
5LOFdock_BOx7	-5,9	7,157	3,2
5LOFdock_BOxNaf5	-5,9	3,035	2,31
5LOFdock_BOxPh7	-5,9	7,997	3,135
5LOFdock_BOxPh8	-5,9	10,038	3,438
5LOFdock_BOxPh9	-5,9	6,482	3,339
5LOFdock_BOxPh9	-5,9	7,448	2,708
5LOFdock_BOxPh9	-5,9	5,814	2,701
5LOFdock_BOxPh9	-5,9	10,413	3,35
5LOFdock_BOxPhCl3	-5,9	4,798	2,845
5LOFdock_BOxPhCl3	-5,9	4,514	2,979
5LOFdock_BOxPhCl5	-5,9	8,358	3,485
5LOFdock_BOxPhCl9	-5,9	0	0

5LOFdock_BOxPhDMN4	-5,9	2,299	1,631
5LOFdock_BOxPhDMN7	-5,9	10,695	3,142
5LOFdock_BOxPhDMN8	-5,9	7,914	3,91
5LOFdock_BOxPhDMN8	-5,9	5,264	2,776
5LOFdock_BOxPhOMe3	-5,9	9,585	3,808
5LOFdock_BOxPhOMe3	-5,9	9,697	5,497
5LOFdock_BOxPhOMe5	-5,9	8,602	3,02
5LOFdock_BOxPhOMe5	-5,9	8,157	3,601
5LOFdock_BOxPhOMe6	-5,9	11,037	2,895
5LOFdock_BOxPhOMe6	-5,9	3,877	1,57
5LOFdock_BOxPhOMe7	-5,9	9,854	3,131
5LOFdock_BOxPhOMe7	-5,9	8,39	2,782
5LOFdock_BOxPhOMe8	-5,9	8,768	3,288
5LOFdock_BOxPhOMe8	-5,9	8,946	2,823
5LOFdock_BOxPhOMe8	-5,9	7,905	2,494
5LOFdock_BA	-5,8	7,581	1,851
5LOFdock_BA	-5,8	5,282	3,425
5LOFdock_BT7	-5,8	11,652	7,073
5LOFdock_BT8	-5,8	16,791	7,288
5LOFdock_BTz4	-5,8	3,829	2,52
5LOFdock_BTz6	-5,8	3,921	2,499
5LOFdock_BTz6	-5,8	12,406	5,593
5LOFdock_BTzNaf8	-5,8	12,819	2,975
5LOFdock_Bet	-5,8	7,262	3,726
5LOFdock_Bet	-5,8	6,84	2,68
5LOFdock_BtzPhCl6	-5,8	12,672	7,629
5LOFdock_BtzPhCl6	-5,8	4,106	2,826
5LOFdock_BtzPhDMN7	-5,8	0	0
5LOFdock_BtzPhDMN7	-5,8	7,406	3,338
5LOFdock_BtzPhDMN9	-5,8	4,794	2,601
5LOFdock_BtzPhOMe3	-5,8	12,826	8,48
5LOFdock_BtzPhOMe5	-5,8	18,919	9,503
5LOFdock_BtzPhOMe7	-5,8	0	0
5LOFdock_BA	-5,8	7,593	1,901
5LOFdock_BOx8	-5,8	10,097	3,33
5LOFdock_BOxNaf7	-5,8	10,489	3,442
5LOFdock_BOxPh5	-5,8	10,349	4,906
5LOFdock_BOxPh7	-5,8	6,031	3,3
5LOFdock_BOxPh8	-5,8	9,159	2,572
5LOFdock_BOxPh9	-5,8	10,633	3,324
5LOFdock_BOxPhCl5	-5,8	6,969	3,352
5LOFdock_BOxPhCl6	-5,8	6,938	3,146
5LOFdock_BOxPhCl7	-5,8	4,491	2,985
5LOFdock_BOxPhCl7	-5,8	7,77	3,263
5LOFdock_BOxPhDMN4	-5,8	6,699	3,123
5LOFdock_BOxPhDMN4	-5,8	4,807	2,078

5LOFdock_BOxPhDMN6	-5,8	0	0
5LOFdock_BOxPhDMN7	-5,8	9,237	5,093
5LOFdock_BOxPhDMN7	-5,8	4,065	3,154
5LOFdock_BOxPhOMe5	-5,8	5,181	2,794
5LOFdock_BOxPhOMe7	-5,8	7,468	3,462
5LOFdock_BA	-5,7	4,831	2,537
5LOFdock_BTz4	-5,7	11,97	5,629
5LOFdock_BTz6	-5,7	4,604	2,579
5LOFdock_BTz6	-5,7	13,087	5,764
5LOFdock_BTzNaf9	-5,7	13,686	5,109
5LOFdock_Btz2	-5,7	10,696	3,511
5LOFdock_BtzPh8	-5,7	9,816	5,222
5LOFdock_BtzPhCl6	-5,7	14,897	6,602
5LOFdock_BtzPhCl8	-5,7	0	0
5LOFdock_BtzPhCl9	-5,7	0	0
5LOFdock_BtzPhDMN5	-5,7	11,777	2,633
5LOFdock_BtzPhDMN5	-5,7	15,151	5,244
5LOFdock_BtzPhDMN8	-5,7	9,61	1,812
5LOFdock_BtzPhDMN9	-5,7	6,424	3,316
5LOFdock_BtzPhDMN9	-5,7	9,31	3,391
5LOFdock_BtzPhOMe8	-5,7	13,4	3,231
5LOFdock_BOx8	-5,7	9,959	3,474
5LOFdock_BOxNaf6	-5,7	9,926	2,943
5LOFdock_BOxPh6	-5,7	8,767	4,401
5LOFdock_BOxPh6	-5,7	7,715	4,526
5LOFdock_BOxPh7	-5,7	10,126	2,907
5LOFdock_BOxPh8	-5,7	6,432	2,592
5LOFdock_BOxPh8	-5,7	8,682	3,238
5LOFdock_BOxPh8	-5,7	11,164	5,326
5LOFdock_BOxPh9	-5,7	10,876	3,013
5LOFdock_BOxPhCl6	-5,7	7,487	4,07
5LOFdock_BOxPhCl7	-5,7	9,933	3,784
5LOFdock_BOxPhOMe6	-5,7	9,41	2,533
5LOFdock_BOxPhOMe6	-5,7	11,344	2,918
5LOFdock_BOxPhOMe8	-5,7	8,394	2,335
5LOFdock_BT8	-5,6	12,385	7,236
5LOFdock_BT9	-5,6	0	0
5LOFdock_BT9	-5,6	10,336	2,477
5LOFdock_BTz6	-5,6	4,315	2,276
5LOFdock_BTz8	-5,6	14,529	5,861
5LOFdock_BTz8	-5,6	13,498	5,416
5LOFdock_Btz2	-5,6	6,734	4,773
5LOFdock_BtzPhCl9	-5,6	14,824	8,848
5LOFdock_BtzPhDMN7	-5,6	4,051	2,149
5LOFdock_BtzPhDMN8	-5,6	2,597	1,781
5LOFdock_BtzPhDMN9	-5,6	7,368	4,012

5LOFdock_BtzPhDMN9	-5,6	9,331	4,051
5LOFdock_BtzPhOMe5	-5,6	10,909	6,755
5LOFdock_BtzPhOMe5	-5,6	17,161	7,977
5LOFdock_BtzPhOMe6	-5,6	11,787	3,742
5LOFdock_BtzPhOMe9	-5,6	6,568	3,503
5LOFdock_BOx8	-5,6	4,002	2,984
5LOFdock_BOx8	-5,6	10,63	5,646
5LOFdock_BOxNaf6	-5,6	9,773	2,815
5LOFdock_BOxPh5	-5,6	5,701	3,642
5LOFdock_BOxPh6	-5,6	6,049	3,108
5LOFdock_BOxPh8	-5,6	8,833	2,915
5LOFdock_BOxPh8	-5,6	10,838	3,574
5LOFdock_BOxPh8	-5,6	8,695	3,325
5LOFdock_BOxPhCl5	-5,6	8,036	3,396
5LOFdock_BOxPhCl7	-5,6	4,573	2,612
5LOFdock_BOxPhCl7	-5,6	10,311	3,636
5LOFdock_BOxPhDMN4	-5,6	9,045	3,854
5LOFdock_BOxPhDMN4	-5,6	7,036	2,562
5LOFdock_BOxPhDMN6	-5,6	9,808	2,905
5LOFdock_BOxPhDMN6	-5,6	8,999	4,506
5LOFdock_BOxPhDMN7	-5,6	8,234	3,207
5LOFdock_BOxPhDMN8	-5,6	5,226	2,855
5LOFdock_BOxPhDMN8	-5,6	8,552	2,749
5LOFdock_BOxPhDMN8	-5,6	7,999	4,012
5LOFdock_BOxPhDMN9	-5,6	10,748	3,375
5LOFdock_BOxPhOMe5	-5,6	11,89	5,521
5LOFdock_BOxPhOMe6	-5,6	10,794	4,967
5LOFdock_BOxPhOMe6	-5,6	5,21	2,522
5LOFdock_BOxPhOMe6	-5,6	5,139	2,346
5LOFdock_BOxPhOMe6	-5,6	10,801	4,951
5LOFdock_Bet	-5,6	5,438	3,523
5LOFdock_Bet	-5,6	6,919	3,11
5LOFdock_BT9	-5,5	11,143	2,009
5LOFdock_BTz4	-5,5	3,17	2,233
5LOFdock_BTz6	-5,5	5,083	3,484
5LOFdock_BTzNaf9	-5,5	8,025	4,984
5LOFdock_Btz2	-5,5	11,382	7,283
5LOFdock_BtzPh8	-5,5	11,832	6,8
5LOFdock_BtzPh8	-5,5	12,031	6,77
5LOFdock_BtzPhDMN5	-5,5	4,665	2,39
5LOFdock_BtzPhDMN6	-5,5	2,878	2,038
5LOFdock_BtzPhDMN6	-5,5	8,103	4,085
5LOFdock_BtzPhDMN6	-5,5	11,351	5,963
5LOFdock_BtzPhDMN7	-5,5	4,787	2,366
5LOFdock_BtzPhDMN8	-5,5	11,884	5,337
5LOFdock_BtzPhDMN9	-5,5	14,467	3,627

5LOFdock_BtzPhOMe5	-5,5	19,262	9,828
5LOFdock_BtzPhOMe6	-5,5	15,013	6,064
5LOFdock_BOx8	-5,5	4,66	3,456
5LOFdock_BOx9	-5,5	0	0
5LOFdock_BOxNaf6	-5,5	12,095	4,239
5LOFdock_BOxNaf9	-5,5	0	0
5LOFdock_BOxPh6	-5,5	5,419	3,053
5LOFdock_BOxPhCl5	-5,5	10,49	4,491
5LOFdock_BOxPhDMN4	-5,5	6,785	2,859
5LOFdock_BOxPhDMN6	-5,5	7,171	3,748
5LOFdock_BOxPhDMN9	-5,5	2,078	1,586
5LOFdock_BOxPhOMe5	-5,5	5,836	2,982
5LOFdock_BOxPhOMe5	-5,5	9,147	4,148
5LOFdock_BOxPhOMe7	-5,5	6,464	3,029
5LOFdock_BOxPhOMe7	-5,5	3,991	2,432
5LOFdock_BOxPhOMe7	-5,5	3,926	2,285
5LOFdock_BTzNaf9	-5,4	8,156	5,347
5LOFdock_BtzPh8	-5,4	10,827	6,017
5LOFdock_BtzPhCl6	-5,4	13,115	7,938
5LOFdock_BtzPhCl6	-5,4	14,948	6,096
5LOFdock_BtzPhCl6	-5,4	5,957	3,32
5LOFdock_BtzPhDMN9	-5,4	15,063	3,078
5LOFdock_BtzPhOMe5	-5,4	10,902	6,428
5LOFdock_BtzPhOMe6	-5,4	8,519	4,44
5LOFdock_BtzPhOMe6	-5,4	13,287	4,419
5LOFdock_BtzPhOMe6	-5,4	3,987	3,129
5LOFdock_BtzPhOMe7	-5,4	17,542	7,56
5LOFdock_BtzPhOMe8	-5,4	9,998	4,155
5LOFdock_BtzPhOMe9	-5,4	12,48	4,002
5LOFdock_BOxPh6	-5,4	7,311	4,331
5LOFdock_BOxPh6	-5,4	9,067	2,977
5LOFdock_BOxPhCl5	-5,4	3,611	2,711
5LOFdock_BOxPhCl8	-5,4	0	0
5LOFdock_BOxPhCl9	-5,4	8,728	4,81
5LOFdock_BOxPhDMN4	-5,4	5,526	2,665
5LOFdock_BOxPhDMN4	-5,4	8,39	2,985
5LOFdock_BOxPhDMN6	-5,4	7,297	2,741
5LOFdock_BOxPhOMe5	-5,4	9,125	4,335
5LOFdock_BOxPhOMe9	-5,4	0	0
5LOFdock_BtzPhCl6	-5,3	6,213	3,679
5LOFdock_BtzPhCl6	-5,3	14,301	6,175
5LOFdock_BtzPhCl8	-5,3	3,044	2,069
5LOFdock_BtzPhCl9	-5,3	13,523	7,57
5LOFdock_BtzPhDMN6	-5,3	8,16	4,574
5LOFdock_BtzPhDMN7	-5,3	3,71	2,153
5LOFdock_BtzPhDMN8	-5,3	11,616	5,129

5LOFdock_BtzPhOMe7	-5,3	3,711	2,757
5LOFdock_BtzPhOMe8	-5,3	13,609	3,16
5LOFdock_BtzPhOMe8	-5,3	15,027	5,503
5LOFdock_BtzPhOMe9	-5,3	13,022	5,778
5LOFdock_BOx9	-5,3	6,335	3,961
5LOFdock_BOxPh5	-5,3	8,236	4,386
5LOFdock_BOxPh5	-5,3	11,331	5,284
5LOFdock_BOxPh6	-5,3	9,136	5,023
5LOFdock_BOxPhCl9	-5,3	8,926	4,532
5LOFdock_BOxPhDMN6	-5,3	9,501	3,349
5LOFdock_BOxPhDMN9	-5,3	10,693	3,362
5LOFdock_BOxPhDMN9	-5,3	7,763	2,507
5LOFdock_BT9	-5,2	13,876	5,517
5LOFdock_BtzPhCl9	-5,2	13,048	8,192
5LOFdock_BtzPhCl9	-5,2	6,912	4,205
5LOFdock_BtzPhDMN7	-5,2	14,331	5,011
5LOFdock_BtzPhDMN8	-5,2	7,741	3,534
5LOFdock_BtzPhOMe7	-5,2	11,546	3,561
5LOFdock_BtzPhOMe8	-5,2	5,294	2,103
5LOFdock_BtzPhOMe8	-5,2	11,025	5,927
5LOFdock_BtzPhOMe9	-5,2	12,553	3,993
5LOFdock_BtzPhOMe9	-5,2	12	6,736
5LOFdock_BtzPhOMe9	-5,2	8,201	4,325
5LOFdock_BOx9	-5,2	3,525	2,184
5LOFdock_BOxPh5	-5,2	8,688	4,56
5LOFdock_BOxPh5	-5,2	4,964	2,507
5LOFdock_BOxPhCl5	-5,2	7,985	3,405
5LOFdock_BOxPhCl9	-5,2	4,346	2,585
5LOFdock_BOxPhCl9	-5,2	6,845	3,418
5LOFdock_BOxPhDMN6	-5,2	1,896	1,588
5LOFdock_BOxPhDMN6	-5,2	10,67	2,531
5LOFdock_BOxPhDMN7	-5,2	8,639	3,326
5LOFdock_BOxPhDMN9	-5,2	9,987	3,511
5LOFdock_BOxPhOMe7	-5,2	8,276	4,736
5LOFdock_BOxPhOMe9	-5,2	9,443	4,358
5LOFdock_BT9	-5,1	2,218	1,47
5LOFdock_Btz9	-5,1	10,188	5,607
5LOFdock_BOx9	-5,1	9,77	3,882
5LOFdock_BOxPhCl5	-5,1	8,162	3,589
5LOFdock_BOxPhCl8	-5,1	8,524	1,995
5LOFdock_BOxPhCl8	-5,1	4,925	2,364
5LOFdock_BOxPhCl9	-5,1	10,984	4,589
5LOFdock_BOxPhCl9	-5,1	11,31	3,941
5LOFdock_BOxPhDMN6	-5,1	8,353	4,185
5LOFdock_BOxPhDMN9	-5,1	11,192	2,584
5LOFdock_BOxPhOMe9	-5,1	10,335	2,701

5LOFdock_BOxPhOMe9	-5,1	9,661	3,143
5LOFdock_BT9	-5	11,764	2,371
5LOFdock_BtzPhCl8	-5	13,037	5,319
5LOFdock_BtzPhCl9	-5	4,133	2,505
5LOFdock_BtzPhCl9	-5	17,06	7,622
5LOFdock_BtzPhCl9	-5	12,224	6,672
5LOFdock_BtzPhDMN7	-5	5,124	2,681
5LOFdock_BtzPhDMN7	-5	4,064	2,173
5LOFdock_BtzPhDMN8	-5	6,547	3,368
5LOFdock_BtzPhDMN8	-5	8,111	4,305
5LOFdock_BtzPhOMe7	-5	11,955	3,162
5LOFdock_BtzPhOMe7	-5	11,066	6,693
5LOFdock_BtzPhOMe8	-5	9,818	4,943
5LOFdock_BOx9	-5	9,626	5,891
5LOFdock_BOx9	-5	9,136	5,389
5LOFdock_BOx9	-5	10,685	4,025
5LOFdock_BOxPhOMe9	-5	8,896	3,773
5LOFdock_BOxPhOMe9	-5	5,809	3,291
5LOFdock_BT9	-4,9	5,904	2,345
5LOFdock_BT9	-4,9	14,347	6,037
5LOFdock_BT9	-4,9	14,014	5,881
5LOFdock_BtzPhCl8	-4,9	2,968	2,09
5LOFdock_BtzPhCl9	-4,9	16,246	5,683
5LOFdock_BtzPhDMN7	-4,9	2,738	1,781
5LOFdock_BtzPhOMe7	-4,9	12,785	3,602
5LOFdock_BOx9	-4,9	8,684	4,475
5LOFdock_BOxPhCl8	-4,9	8,554	2,437
5LOFdock_BOxPhCl9	-4,9	9,912	4,068
5LOFdock_BOxPhDMN9	-4,9	9,577	3,154
5LOFdock_BOxPhDMN9	-4,9	9,432	3,132
5LOFdock_Btz9	-4,8	14,889	6,449
5LOFdock_Btz9	-4,8	10,421	5,685
5LOFdock_Btz9	-4,8	2,322	1,473
5LOFdock_BtzPhOMe7	-4,8	16,77	6,673
5LOFdock_BtzPhOMe8	-4,8	10,589	5,681
5LOFdock_BOx9	-4,8	9,087	5,378
5LOFdock_BOxPhCl9	-4,8	9,32	4,629
5LOFdock_BOxPhOMe9	-4,8	9,644	4,741
5LOFdock_BOxPhOMe9	-4,8	10,441	5,83
5LOFdock_BtzPhCl8	-4,7	13,317	7,975
5LOFdock_BtzPhCl8	-4,7	4,236	2,909
5LOFdock_BOxNaf9	-4,7	9,185	4,899
5LOFdock_BOxNaf9	-4,7	10,331	4,338
5LOFdock_BOxPhCl8	-4,7	3,422	2,176
5LOFdock_BOxPhOMe9	-4,7	8,015	3,541
5LOFdock_BtzPhOMe7	-4,6	11,4	6,93

5LOFdock_BOxNaf9	-4,6	2,029	1,531
5LOFdock_BOxNaf9	-4,6	8,143	2,564
5LOFdock_BOxPhCl8	-4,6	8,175	1,943
5LOFdock_Btz9	-4,5	15,257	6,753
5LOFdock_Btz9	-4,5	10,633	6,015
5LOFdock_Btz9	-4,5	17,233	8,877
5LOFdock_Btz9	-4,5	16,803	8,424
5LOFdock_BtzPhCl8	-4,5	7,589	3,227
5LOFdock_BtzPhCl8	-4,5	3,312	2,462
5LOFdock_BOxNaf9	-4,5	11,535	5,112
5LOFdock_BOxPhCl8	-4,5	5,754	3,287
5LOFdock_BOxPhCl8	-4,5	8,985	3,09
5LOFdock_BOxPhCl8	-4,5	7,799	3,714
5LOFdock_BtzPhCl8	-4,4	7,434	3,337
5LOFdock_BOxNaf9	-4,4	8,365	4,273
5LOFdock_BOxNaf9	-4,4	8,255	3,919
5LOFdock_BOxNaf9	-4,4	7,48	4,043

Table S5. Glide docking scores of docked ligands against Bcl-XL (2YXJ)

title	i_i_glide_lignum	docking_status	r_glide_cpu_time	r_i_docking_score	s_i_glide_gridfile	r_i_glide_gscore	r_i_glide_lipo	r_i_glide_hbond	r_i_glide_metal	r_i_glide_rewards	r_i_glide_evdw	r_i_glide_ecoul	r_i_glide_erotb	r_i_glide_esite	r_i_glide_emodel	r_i_glide_energy	r_i_glide_einternal
2YXJ_ligand	1	Done	115,29	-9,45931	glide-grid_2YXJtest	-9,49851	-4,15831	0	0	-1,31313	-62,9486	-7,55541	0,253677	0	-115,769	-70,504	11,6942
BOxPhCl1	30	Done	56,41	-8,79298	glide-grid_2YXJtest	-8,79298	-5,0262	0	0	-0,36979	-66,4738	-1,21248	0,124234	-0,01566	-96,767	-67,6863	14,8967
BTzPhCl7	298	Done	68,12	-8,72716	glide-grid_2YXJtest	-10,1275	-4,69746	-0,16	0	-0,62941	-78,1886	-5,72144	0,248219	-0,12117	-124,744	-83,91	19,7227
BT5	5	Done	65,65	-8,70308	glide-grid_2YXJtest	-8,70308	-4,90028	0	0	-0,5611	-65,3933	-1,01221	0,266438	-0,08664	-95,5691	-66,4056	7,32417
BOxPhCl8	37	Done	81,12	-8,56715	glide-grid_2YXJtest	-8,56715	-4,38948	0	0	-0,5278	-74,4687	-1,07392	0,259952	-0,0253	-111,735	-75,5426	10,0523
BOxPhCl4	33	Done	82,62	-8,4818	glide-grid_2YXJtest	-8,4818	-3,6937	-0,24648	0	-0,49719	-69,7933	-4,6733	0,196117	-0,04989	-104,92	-74,4666	11,2603
BTz9	116	Done	63,27	-8,48062	glide-grid_2YXJtest	-8,93932	-3,08909	-0,5906	0	-1,27119	-62,8153	-8,03463	0,41828	-0,06075	-102,012	-70,85	13,2203
BOxNaf5	16	Done	64,21	-8,46811	glide-grid_2YXJtest	-8,46811	-4,27703	-0,19061	0	-0,23466	-67,0578	-3,96886	0,19158	-0,00917	-99,3548	-71,0267	13,9768
BOxPhOMe1	48	Done	48,99	-8,17891	glide-grid_2YXJtest	-8,17891	-4,68768	0	0	-0,15582	-66,8744	-0,81731	0,130906	0	-89,7585	-67,6917	23,5173

2YXJ_ligand	2	Done	98,13	-8,06308	glide-grid_2YXJtest	-9,69228	-4,58463	0	0	-1,13324	-66,7183	-5,93926	0,254936	-0,00254	-109,665	-72,6575	19,4448
BTzNaf7	170	Done	57,35	-7,98536	glide-grid_2YXJtest	-8,76546	-3,55596	-0,39349	0	-0,56754	-56,2792	-10,1922	0,225404	-0,13109	-98,9506	-66,4714	11,0324
BOxNaf1	12	Done	46,47	-7,94592	glide-grid_2YXJtest	-7,94592	-4,18837	-0,01696	0	-0,46141	-61,3012	-1,36932	0,102315	-0,11104	-91,9496	-62,6705	5,23956
BOxPhDMN7	45	Done	67,54	-7,92306	glide-grid_2YXJtest	-7,92306	-3,16989	0	0	-0,57497	-70,1395	-5,61827	0,233478	-0,06197	-106,045	-75,7578	10,0937
BT1	1	Done	30,69	-7,899	glide-grid_2YXJtest	-7,899	-4,6862	0	0	-0,66589	-54,2088	0,135332	0,143227	0	-76,8857	-54,0735	9,08663
BTzPhCl3	272	Done	80,47	-7,87914	glide-grid_2YXJtest	-8,77414	-3,26479	-0,32	0	-0,77081	-64,445	-8,99941	0,17724	-0,02363	-106,669	-73,4444	11,3394
BOxNaf4	15	Done	56,43	-7,8158	glide-grid_2YXJtest	-7,8158	-3,8368	0	0	-0,20557	-71,5733	-2,37053	0,172882	-0,01207	-103,425	-73,9438	9,07668
BOxPh6	26	Done	68,23	-7,78445	glide-grid_2YXJtest	-7,78445	-4,16011	0	0	-0,15939	-66,452	-2,42612	0,292205	-0,07063	-93,8241	-68,8781	12,4035
BTzPhOMe7	432	Done	77,76	-7,78042	glide-grid_2YXJtest	-8,79452	-3,6775	-0,32	0	-0,87957	-70,9814	-4,12264	0,256104	-0,00608	-113,389	-75,104	14,4605
BTz7	100	Done	76,53	-7,77154	glide-grid_2YXJtest	-8,23034	-2,95663	-0,55334	0	-0,89222	-63,6089	-6,91942	0,390204	0	-96,9029	-70,5283	15,9603
BT3	3	Done	53,79	-7,7351	glide-grid_2YXJtest	-7,7351	-4,11015	-0,03116	0	-0,61579	-60,154	-1,18318	0,213345	-0,00617	-86,1444	-61,3372	10,9114
BOxNaf3	14	Done	47,88	-7,71975	glide-grid_2YXJtest	-8,47455	-2,51501	-0,71012	0	-0,57971	-58,1018	-12,5935	0,164133	-0,03972	-103,131	-70,6953	6,04635
BOxPhOMe2	49	Done	73,29	-7,69511	glide-grid_2YXJtest	-7,69511	-4,07545	-0,1114	0	-0,10845	-61,0398	-3,33317	0,157917	-0,00577	-88,144	-64,373	13,7466
BTzNaf8	180	Done	74,18	-7,68009	glide-grid_2YX	-9,12829	-3,0509	-0,88875	0	-0,31632	-68,6765	-10,4134	0,238059	-0,1144	-115,648	-79,09	12,0811

					Jtest		6							8			
BOxPh2	22	Done	71,77	-7,67627	glide-grid_2YXJtest	-7,67627	-4,50323	0	0	-0,25892	-61,3474	-0,38239	0,210609	0	-85,1945	-61,7298	8,29469
BT6	6	Done	63,35	-7,66678	glide-grid_2YXJtest	-7,66678	-4,10854	0	0	-0,42423	-60,425	-2,08519	0,287971	-0,08795	-88,8796	-62,5102	7,79209
BTzPhDMN3	328	Done	64,02	-7,64827	glide-grid_2YXJtest	-7,64827	-3,74558	0	0	-0,2746	-68,3938	-2,40244	0,151965	0	-99,1197	-70,7962	8,385
BT7	7	Done	66,95	-7,5942	glide-grid_2YXJtest	-7,5942	-4,00014	-0,14223	0	-0,34726	-63,5974	-1,49678	0,306732	-0,00692	-90,3815	-65,0941	8,25296
BOxPhDMN1	39	Done	39,56	-7,59176	glide-grid_2YXJtest	-7,59176	-4,0895	0	0	-0,36559	-62,7218	-0,57857	0,111822	-0,02562	-83,3474	-63,3003	22,3479
BOxPhCl5	34	Done	83,01	-7,58278	glide-grid_2YXJtest	-7,58278	-4,13916	0	0	-0,09583	-69,4772	-0,59264	0,214968	0	-96,804	-70,0698	9,7129
BTzPhOMe6	425	Done	76,87	-7,57956	glide-grid_2YXJtest	-8,95976	-3,12932	-0,56898	0	-0,70933	-65,2519	-9,94973	0,240388	-0,03747	-104,9	-75,2016	21,6408
BTzNaf7	175	Done	55,84	-7,56367	glide-grid_2YXJtest	-10,0958	-5,09558	-0,16	0	-0,58232	-76,7813	-3,45852	0,224677	-0,12471	-123,514	-80,2398	8,59881
BOxPh3	23	Done	77,76	-7,56046	glide-grid_2YXJtest	-7,56046	-3,55899	-0,29916	0	-0,19943	-60,5176	-4,63065	0,235165	-0,01757	-89,6067	-65,1483	9,82053
BT8	8	Done	69,43	-7,55028	glide-grid_2YXJtest	-7,55028	-3,55981	-0,23808	0	-0,42978	-67,2094	-1,83482	0,323069	-0,00999	-95,9434	-69,0442	10,5815
BT2	2	Done	45,62	-7,54845	glide-grid_2YXJtest	-7,54845	-3,7532	-0,28357	0	-0,47679	-56,1289	-2,72789	0,180737	0	-78,6446	-58,8568	14,6598
BOxNaf8	19	Done	64,49	-7,54149	glide-grid_2YXJtest	-7,54149	-3,90745	0	0	-0,36025	-70,1581	-0,0169	0,23665	0	-98,9909	-70,175	9,56521
BTzPhOMe3	397	Done	64,11	-7,51682	glide-grid_2YXJtest	-8,53412	-2,79854	-0,608	0	-0,28529	-55,7804	-14,4821	0,18426	-0,06522	-101,446	-70,2624	10,6425

BOxPhOMe6	53	Done	80,26	-7,50355	glide-grid_2YXJtest	-7,50355	-3,64165	0	0	-0,42293	-67,1006	-2,1185	0,23879	-0,00496	-95,275	-69,2191	12,5543
BTz5	84	Done	84,25	-7,40512	glide-grid_2YXJtest	-7,87102	-2,80717	-0,45227	0	-1,1231	-54,4277	-7,05618	0,351423	-0,06009	-86,524	-61,4839	9,98947
BTzPhDMN8	364	Done	62,94	-7,37017	glide-grid_2YXJtest	-7,99067	-3,14815	-0,32	0	-0,23378	-56,3077	-9,6868	0,249802	-0,27014	-95,8682	-65,9945	8,88606
BTzPhCl5	286	Done	62,56	-7,36788	glide-grid_2YXJtest	-8,78278	-4,22794	-0,16343	0	-0,57673	-58,9899	-6,53661	0,216522	-0,10121	-97,7077	-65,5265	11,0688
BOxNaf6	17	Done	45,24	-7,32501	glide-grid_2YXJtest	-7,32501	-4,0462	0	0	-0,21097	-55,6736	-3,25153	0,208302	-0,00473	-84,9018	-58,9251	5,07176
BTzNaf3	138	Done	59,06	-7,2885	glide-grid_2YXJtest	-7,8827	-3,97124	-0,06214	0	-0,64376	-60,5913	-1,85418	0,154763	-0,05263	-93,5164	-62,4455	7,38287
BTz7	107	Done	82,44	-7,25348	glide-grid_2YXJtest	-9,79848	-3,60238	-0,608	0	-1,56624	-62,3734	-8,58105	0,390204	-0,00623	-104,559	-70,9545	18,6043
BT4	4	Done	67,1	-7,23285	glide-grid_2YXJtest	-7,23285	-3,45325	-0,08879	0	-0,63292	-59,8108	-2,04173	0,241723	-0,00281	-83,6717	-61,8525	9,22007
BOx6	8	Done	107,74	-7,21109	glide-grid_2YXJtest	-7,21109	-3,49884	0	0	-0,43512	-60,6267	-3,7642	0,369602	-0,05077	-85,8735	-64,3909	14,387
BTzPh9	256	Done	70,8	-7,20465	glide-grid_2YXJtest	-8,19545	-3,63476	-0,48443	0	-0,41818	-68,4839	-2,04043	0,333491	-0,2613	-96,4855	-70,5244	30,506
BOxPhDMN2	40	Done	58,21	-7,19642	glide-grid_2YXJtest	-7,19642	-3,50499	-0,06183	0	-0,25603	-59,8335	-3,43031	0,138334	-0,00568	-87,4641	-63,2638	9,34686
BOxPhDMN5	43	Done	69,83	-7,19575	glide-grid_2YXJtest	-7,19575	-3,19832	0	0	-0,22613	-65,4926	-4,6563	0,201769	0	-96,3137	-70,1489	5,96081
BOxPhOMe7	54	Done	66,83	-7,19191	glide-grid_2YXJtest	-7,19191	-3,55653	0	0	-0,26915	-68,14	-1,05545	0,253695	-0,0546	-89,8966	-69,1954	19,4588
BTzPhDMN	330	Done	47,35	-7,16567	glide-grid_2YX	-8,49877	-2,6490	-0,79135	0	-0,44832	-57,5781	-12,2502	0,163391	-0,0570	-99,6047	-69,8283	9,02081

3					Jtest		5							2			
BOxPh4	24	Done	60,04	-7,16228	glide-grid_2YXJtest	-7,16228	-3,78576	0	0	-0,23232	-65,4998	-0,71689	0,256709	-0,01839	-85,4637	-66,2167	18,245
BOxPhOMe3	50	Done	79,3	-7,16115	glide-grid_2YXJtest	-7,16115	-3,27861	-0,16	0	-0,14129	-61,9873	-4,34739	0,181871	-0,01165	-93,3468	-66,3347	3,93126
BOxPhOMe8	55	Done	81,9	-7,15803	glide-grid_2YXJtest	-7,15803	-3,09442	0	0	-0,23762	-67,1788	-3,70445	0,266935	-0,17831	-92,2308	-70,8832	21,7262
BOxPhCl7	36	Done	65,93	-7,13281	glide-grid_2YXJtest	-7,13281	-3,4671	0	0	0	-64,5631	-4,2101	0,246669	-0,0527	-93,3588	-68,7732	9,82842
BOxPhDMN4	42	Done	60,76	-7,12372	glide-grid_2YXJtest	-7,12372	-2,75395	-0,16	0	-0,35618	-62,2747	-5,13789	0,182995	-0,15217	-92,8246	-67,4126	9,75163
BTzNaf1	129	Done	38,98	-7,11186	glide-grid_2YXJtest	-9,45866	-3,76467	-0,26843	0	-1,54162	-63,0638	-5,56182	0,103626	-0,0001	-104,434	-68,6256	7,85845
BTzNaf3	139	Done	62,29	-7,11045	glide-grid_2YXJtest	-7,86525	-3,48181	0	0	-0,70976	-64,2669	-3,61094	0,154052	-0,07275	-98,7401	-67,8779	8,37303
BTzNaf2	133	Done	66,62	-7,10718	glide-grid_2YXJtest	-7,96508	-3,68567	-0,18382	0	-0,28587	-63,755	-4,65865	0,130594	-0,05376	-98,4406	-68,4137	12,7465
BOxNaf2	13	Done	53,71	-7,10481	glide-grid_2YXJtest	-7,10481	-2,48024	-0,43627	0	-0,14679	-58,7035	-7,91703	0,12855	-0,04734	-90,1788	-66,6205	11,8389
BTzPhDMN5	343	Done	59,46	-7,0664	glide-grid_2YXJtest	-7,678	-4,30324	0	0	-0,82782	-55,337	0,273208	0,204756	-0,02583	-82,1369	-55,0638	7,96116
BOxPhDMN6	44	Done	66,37	-7,01683	glide-grid_2YXJtest	-7,01683	-2,69922	-0,16	0	-0,28566	-65,8029	-4,30764	0,218523	-0,15419	-93,567	-70,1105	16,2537
BOxPh7	27	Done	60,9	-7,01001	glide-grid_2YXJtest	-7,01001	-3,57942	0	0	0	-62,9052	-3,57745	0,306757	-0,05547	-91,8035	-66,4827	9,28685
BTz8	108	Done	67,09	-6,99784	glide-grid_2YXJtest	-7,45654	-2,7637	-0,32032	0	-1,28924	-55,2448	-4,84294	0,405393	0	-85,1655	-60,0877	9,3482

BTzNaf1	125	Done	41,7	-6,97608	glide-grid_2YXJtest	-7,71968	-4,2758	0	0	-0,36567	-61,8569	-0,24209	0,1043	-0,05335	-89,9346	-62,099	6,20895
BTz6	94	Done	88,3	-6,96613	glide-grid_2YXJtest	-7,90833	-2,89074	-0,608	0	-0,85577	-51,1239	-7,58106	0,373771	-0,23424	-86,2862	-58,705	10,8628
BTzPhDMN1	313	Done	33,95	-6,94972	glide-grid_2YXJtest	-7,61662	-3,80715	-0,04805	0	-0,64275	-58,8983	-1,46393	0,114621	-0,06879	-88,1833	-60,3622	7,84905
BTz6	92	Done	90,5	-6,93761	glide-grid_2YXJtest	-7,39731	-2,69354	-0,32	0	-1,09121	-56,3639	-5,2639	0,372355	-0,05714	-87,2588	-61,6278	7,93566
BOxPhDMN3	41	Done	55,55	-6,92856	glide-grid_2YXJtest	-6,92856	-2,63584	-0,28918	0	-0,2663	-58,0953	-6,54828	0,161947	-0,01218	-89,2362	-64,6435	6,0293
BTzPhOMe2	387	Done	61,65	-6,92143	glide-grid_2YXJtest	-7,44293	-2,63444	-0,30348	0	-0,35273	-58,3492	-9,17161	0,161071	-0,02015	-92,4898	-67,5208	17,8032
BOxPhCl3	32	Done	74,57	-6,90079	glide-grid_2YXJtest	-6,90079	-2,65958	-0,30074	0	-0,1967	-57,1266	-7,07264	0,174922	-0,00146	-88,4314	-64,1993	4,29537
BOxPhOMe5	52	Done	68,36	-6,89026	glide-grid_2YXJtest	-6,89026	-2,64529	-0,16	0	-0,24181	-62,5551	-5,98893	0,222015	-0,03908	-90,1058	-68,544	17,4277
BOxPhDMN8	46	Done	73,23	-6,88669	glide-grid_2YXJtest	-6,88669	-2,81475	-0,16	0	-0,32841	-68,313	-1,93423	0,246826	-0,12456	-93,6797	-70,2472	14,3135
BOxPh8	28	Done	75,32	-6,83955	glide-grid_2YXJtest	-6,83955	-3,47863	0	0	-0,08626	-63,3492	-2,84467	0,319509	0	-88,1804	-66,1939	12,7938
BTzPhDMN3	327	Done	60,48	-6,80424	glide-grid_2YXJtest	-7,39844	-2,61873	-0,2795	0	-0,38741	-59,3676	-8,6291	0,164879	-0,01494	-97,3858	-67,9967	7,73634
BOxPhCl9	38	Done	61,37	-6,80348	glide-grid_2YXJtest	-6,80348	-3,1401	-0,01765	0	-0,12306	-61,5941	-3,69203	0,271767	-0,16092	-85,3357	-65,2862	19,2015
BOxPhCl2	31	Done	70,23	-6,79382	glide-grid_2YXJtest	-6,79382	-3,2373	-0,16	0	-0,20011	-57,6979	-3,00513	0,151079	-0,01183	-83,2252	-60,703	7,00903
BOxPh5	25	Done	80,99	-6,74295	glide-grid_2YX	-6,74295	-3,7450	0	0	-0,15848	-66,2953	-1,37006	0,275615	-0,0058	-85,0558	-64,9253	12,2031

					Jtest		2							1			
BTzPh<br>6	230	Done	67,2	-6,73967	glide-<br>grid_2YX<br>Jtest	-<br>7,30037	-<br>3,0166	-0,16	0	-0,65701	-<br>51,8284	-<br>7,22885	0,29612<br>4	-<br>0,0871<br>3	-90,041	-59,0572	4,54781
BTzPh<br>3	208	Done	82,08	-6,73037	glide-<br>grid_2YX<br>Jtest	-<br>7,48517	-<br>2,3841<br>3	-0,59494	0	-0,31928	-<br>53,0452	-<br>11,6042	0,23811<br>2	-<br>0,0320<br>4	-91,5196	-64,6494	6,61583
BTzPh<br>Cl2	270	Done	59,27	-6,72836	glide-<br>grid_2YX<br>Jtest	-<br>8,30596	-<br>4,0193<br>2	0	0	-1,10345	-<br>69,3797	0,92594<br>8	0,15258<br>5	-<br>0,0056<br>8	-99,4578	-68,4537	8,99669
BOxP<br>hCl6	35	Done	73,81	-6,7223	glide-<br>grid_2YX<br>Jtest	-6,7223	-<br>3,5365<br>5	0	0	-0,14743	-<br>57,8414	-2,5199	0,23174<br>2	0	-78,5013	-60,3613	12,9685
BOx5	7	Done	117,65	-6,7185	glide-<br>grid_2YX<br>Jtest	-6,7185	-<br>3,3871<br>7	0	0	-0,30948	-<br>56,6471	-<br>3,22804	0,34863<br>8	-<br>0,0539<br>3	-80,1571	-59,8752	9,67852
BTzPh<br>4	216	Done	62,07	-6,68046	glide-<br>grid_2YX<br>Jtest	-<br>7,46146	-<br>2,7325<br>6	-0,32	0	-0,33771	-<br>61,6287	-<br>8,10207	0,25966	-<br>0,0341<br>1	-94,9904	-69,7307	13,4626
BTzN<br>af3	141	Done	62,45	-6,67753	glide-<br>grid_2YX<br>Jtest	-<br>8,01063	-<br>2,5058<br>3	-0,51479	0	-0,47607	-<br>59,9954	-<br>10,8689	0,15334<br>3	-<br>0,0371<br>8	-100,853	-70,8644	4,95934
BTzPh<br>OMe8	444	Done	74,33	-6,67639	glide-<br>grid_2YX<br>Jtest	-<br>8,31229	-<br>4,1504<br>9	-0,16	0	-0,21126	-<br>64,4148	-<br>3,25433	0,26932<br>8	-<br>0,3509<br>8	-100,769	-67,6691	14,9686
BTzPh<br>DMN<br>5	346	Done	52,61	-6,67392	glide-<br>grid_2YX<br>Jtest	-<br>7,73782	-<br>2,8768<br>3	-0,33261	0	-0,21182	-61,098	-<br>9,36253	0,20324<br>1	-<br>0,0605<br>2	-98,5006	-70,4605	9,33828
BOxP<br>h1	21	Done	58,85	-6,65969	glide-<br>grid_2YX<br>Jtest	-<br>6,65969	-<br>3,1813<br>7	0	0	-0,08048	-<br>53,3033	-<br>5,26477	0,18260<br>5	-<br>0,1255<br>6	-79,7356	-58,5681	9,15986
BTzPh<br>Cl8	301	Done	64,09	-6,64333	glide-<br>grid_2YX<br>Jtest	-<br>7,04223	-<br>3,2628<br>7	-0,16	0	-0,51851	-<br>56,3729	-<br>3,21474	0,26307<br>9	-<br>0,0630<br>8	-86,3606	-59,5876	12,5901
BTzN<br>af2	132	Done	50,61	-6,64203	glide-<br>grid_2YX<br>Jtest	-<br>7,12973	-<br>4,0952<br>1	-0,0216	0	-0,56806	-<br>51,1811	1,30584	0,13129<br>1	-<br>0,2129<br>7	-73,7447	-49,8752	3,81225
BOx4	6	Done	115,67	-6,64135	glide-<br>grid_2YX<br>Jtest	-<br>6,64135	-<br>3,4052<br>6	0	0	-0,30802	-<br>55,1107	-<br>3,16992	0,32410<br>1	-<br>0,0211<br>5	-76,7435	-58,2806	10,1311

BTzPhCl3	273	Done	80,48	-6,62682	glide-grid_2YXJtest	-7,74842	-3,07868	-0,30755	0	-0,90943	-62,6853	-3,28143	0,17724	-0,00351	-94,2701	-65,9667	11,0195
BTzNaf4	146	Done	61,01	-6,61624	glide-grid_2YXJtest	-7,18294	-3,12198	-0,2312	0	-0,31397	-49,1897	-7,80955	0,175718	-0,06059	-86,5259	-56,9992	7,1618
BTzNaf4	151	Done	49,35	-6,61056	glide-grid_2YXJtest	-9,06896	-3,66081	-0,71124	0	-0,53732	-48,2047	-10,697	0,174997	-0,31981	-93,9783	-58,9016	8,0478
BTzNaf7	172	Done	60,86	-6,6049	glide-grid_2YXJtest	-8,0531	-3,03602	-0,0704	0	-0,886	-72,6866	-4,34025	0,224677	0	-113,056	-77,0269	9,32997
BTzPhCl9	307	Done	68,42	-6,59793	glide-grid_2YXJtest	-6,99683	-2,66546	-0,10735	0	-0,08769	-64,8854	-6,58338	0,274868	-0,17941	-99,2624	-71,4688	14,8546
BTzNaf3	140	Done	67,31	-6,5774	glide-grid_2YXJtest	-7,6011	-3,26883	0	0	-0,86171	-69,244	-1,02996	0,154052	-0,00792	-103,37	-70,2739	4,56152
BOx9	11	Done	81,93	-6,56654	glide-grid_2YXJtest	-6,56654	-3,14194	0	0	0	-58,1763	-6,00037	0,415668	-0,0314	-82,606	-64,1766	15,272
BTzPhCl5	285	Done	64,42	-6,56599	glide-grid_2YXJtest	-7,71659	-2,4351	-0,58329	0	-0,386	-55,5917	-11,3455	0,21732	-0,04811	-96,3612	-66,9372	8,41567
BTzPhDMN8	367	Done	79,74	-6,55761	glide-grid_2YXJtest	-7,60981	-2,67376	-0,16	0	-0,5461	-60,9824	-7,95475	0,248293	-0,2359	-97,5149	-68,9372	9,99757
BTzPhOMe3	402	Done	64,11	-6,54452	glide-grid_2YXJtest	-8,51732	-3,31372	-0,4948	0	-0,6744	-65,6899	-6,14296	0,183449	-0,0119	-102,065	-71,8329	13,1145
BTzPhOMe2	389	Done	60,22	-6,5294	glide-grid_2YXJtest	-7,5616	-4,07969	0	0	-0,38261	-59,8559	-1,68147	0,160269	-0,01456	-87,8016	-61,5374	10,6793
BOx7	9	Done	93,19	-6,52496	glide-grid_2YXJtest	-6,52496	-2,92138	0	0	-0,45217	-57,9652	-4,27095	0,387492	0	-81,2277	-62,2361	12,9213
BOxPhDMN9	47	Done	75,84	-6,51978	glide-grid_2YXJtest	-6,51978	-2,06346	-0,16	0	-0,12768	-59,5824	-8,26468	0,258739	-0,20856	-90,1542	-67,8471	12,8125
BTzPhCl5	283	Done	77,06	-6,51821	glide-grid_2YX	-6,91081	-3,2083	-0,16	0	-0,58363	-57,246	-1,86608	0,218122	-0,0347	-87,1975	-59,1121	5,7494

					Jtest		1							8			
BTzNaf4	152	Done	53	-6,50531	glide-grid_2YXJtest	-9,02871	-3,35144	-0,31975	0	-1,15382	-65,8888	-6,97163	0,174279	-0,0378	-102,707	-72,8604	19,8899
BTz7	105	Done	72,29	-6,50365	glide-grid_2YXJtest	-8,85855	-3,04503	-0,608	0	-1,20585	-58,4806	-9,73444	0,390204	-0,00568	-95,0163	-68,215	19,7892
BTzPhCl8	302	Done	62,17	-6,49672	glide-grid_2YXJtest	-7,39632	-2,599	-0,47866	0	-0,15389	-59,1363	-9,0327	0,262284	-0,11534	-92,8277	-68,169	17,1073
BOxPhOMe4	51	Done	55,85	-6,49666	glide-grid_2YXJtest	-6,49666	-2,42678	-0,16	0	-0,28137	-59,3458	-5,04881	0,203133	-0,10704	-87,633	-64,3946	8,18571
BTzNaf5	154	Done	61,37	-6,47926	glide-grid_2YXJtest	-7,09086	-2,9194	-0,19334	0	-0,40744	-57,8316	-4,11173	0,194439	-0,25679	-91,1852	-61,9433	8,70711
BTz3	68	Done	57,47	-6,43589	glide-grid_2YXJtest	-6,89219	-2,28203	-0,59672	0	-0,30345	-40,2637	-12,17	0,298207	-0,16951	-74,168	-52,4336	4,61053
BTzPhOMe5	413	Done	88,07	-6,42949	glide-grid_2YXJtest	-7,06829	-2,83344	-0,27425	0	-0,39552	-58,2086	-5,81284	0,225259	-0,00799	-91,4478	-64,0214	10,5989
BTzPh9	255	Done	72,39	-6,42281	glide-grid_2YXJtest	-7,20291	-3,16115	-0,39909	0	-0,26246	-59,4215	-4,4449	0,333491	-0,07589	-91,1629	-63,8664	10,1649
BTzPhDMN6	352	Done	50,14	-6,41728	glide-grid_2YXJtest	-7,46368	-3,05677	-0,32	0	-0,26908	-56,9863	-6,83388	0,220756	-0,16419	-89,4088	-63,8201	13,4342
BOxNaf9	20	Done	71,27	-6,39388	glide-grid_2YXJtest	-6,39388	-2,6253	-0,06664	0	0	-64,3757	-3,51526	0,248626	-0,20449	-87,2923	-67,891	15,3387
BTzNaf4	148	Done	55,25	-6,38443	glide-grid_2YXJtest	-7,36793	-2,40808	-0,43253	0	-0,15058	-53,6934	-10,3367	0,174997	-0,31657	-93,2152	-64,0301	8,06604
BTzPhOMe1	384	Done	39,4	-6,36908	glide-grid_2YXJtest	-8,20248	-3,37528	-0,12962	0	-0,56726	-53,0546	-9,76755	0,132423	-0,14488	-89,7736	-62,8222	12,8557
BTzPhCl9	309	Done	58,31	-6,36766	glide-grid_2YXJtest	-7,51336	-2,98096	-0,0195	0	-0,32262	-68,4541	-5,18375	0,274081	-0,2641	-104,995	-73,6378	12,2265

BTz6	93	Done	94,32	-6,36669	glide-grid_2YXJtest	-7,30889	-2,98996	-0,32	0	-1,04838	-56,5745	-3,04268	0,373771	-0,03919	-84,6252	-59,6172	11,0098
BTzNaf6	164	Done	62,01	-6,36407	glide-grid_2YXJtest	-7,81227	-2,58041	-0,71784	0	-0,34249	-55,3481	-10,1963	0,209716	-0,0844	-93,2263	-65,5444	7,87702
BTzPh3	209	Done	73,93	-6,36071	glide-grid_2YXJtest	-7,38441	-2,66343	-0,4994	0	-0,45845	-59,0168	-6,92822	0,238112	-0,01116	-90,5548	-65,9451	14,135
BTzPhDMN5	344	Done	54,1	-6,3544	glide-grid_2YXJtest	-7,1781	-2,29941	-0,26728	0	-0,74303	-53,5433	-8,58718	0,203997	-0,10713	-88,3097	-62,1305	7,66113
BTzPhCl2	268	Done	66,55	-6,35056	glide-grid_2YXJtest	-7,29936	-2,36157	-0,45379	0	-0,42092	-56,3971	-9,26447	0,153359	-0,00692	-91,4968	-65,6615	12,1484
BTzPh3	210	Done	74,54	-6,33668	glide-grid_2YXJtest	-7,66978	-2,38647	-0,66497	0	-0,46388	-57,0219	-10,0583	0,237111	-0,03173	-94,9185	-67,0802	6,15166
BTz9	121	Done	67,42	-6,32465	glide-grid_2YXJtest	-8,67945	-3,26264	-0,31711	0	-1,19495	-64,035	-6,79321	0,41828	-0,1023	-100,287	-70,8282	13,1197
BTzPhDMN3	329	Done	46,61	-6,32041	glide-grid_2YXJtest	-7,34411	-2,6306	-0,18925	0	-0,52152	-60,7318	-6,93086	0,164133	-0,09066	-93,0755	-67,6626	10,3316
BTzPhDMN7	358	Done	52,5	-6,29391	glide-grid_2YXJtest	-7,11621	-1,84296	-0,62629	0	-0,56098	-48,3009	-11,0873	0,235707	-0,24354	-87,5673	-59,3882	8,61511
BTzNaf6	163	Done	55,91	-6,29185	glide-grid_2YXJtest	-7,28265	-3,36554	-0,32	0	-0,48774	-62,7245	-0,96657	0,210443	-0,03862	-96,3743	-63,691	7,11221
BTzPh3	207	Done	93,74	-6,2892	glide-grid_2YXJtest	-6,8834	-2,49908	-0,45806	0	-0,33221	-54,2129	-7,4067	0,239117	-0,01151	-87,3079	-61,6196	6,51969
BTzNaf5	160	Done	64,45	-6,28491	glide-grid_2YXJtest	-8,88021	-4,11827	-0,32	0	-0,46022	-64,8628	-6,02342	0,192988	-0,02805	-106,836	-70,8863	6,49879
BTzPhDMN9	374	Done	69,39	-6,23933	glide-grid_2YXJtest	-7,29153	-2,56858	-0,16	0	-0,25888	-65,3436	-7,41665	0,260195	-0,18459	-102,312	-72,7602	10,0897
BTzPhOMe1	383	Done	40,23	-6,23706	glide-grid_2YX	-7,98586	-3,7895	-0,16	0	-0,59926	-59,4961	-3,26426	0,133203	-0,1058	-90,9664	-62,7604	11,258

					Jtest		2							4			
BTzPh9	254	Done	62,49	-6,22946	glide-grid_2YXJtest	-6,79016	-2,10833	-0,43728	0	-0,14185	-52,8192	-9,64121	0,334451	-0,35002	-85,1814	-62,4604	18,6811
BOx3	5	Done	98,05	-6,2247	glide-grid_2YXJtest	-6,2247	-2,4573	-0,16782	0	-0,23901	-49,868	-7,63102	0,295398	-0,01791	-74,6383	-57,499	9,36323
BTzNaf2	134	Done	44,48	-6,20865	glide-grid_2YXJtest	-7,28095	-2,52615	-0,42148	0	-0,30733	-53,7281	-7,84173	0,130594	-0,29393	-89,1098	-61,5698	9,49512
BOx8	10	Done	75,95	-6,2052	glide-grid_2YXJtest	-6,2052	-2,75453	0	0	0	-49,3572	-7,80853	0,402729	-0,21425	-77,1971	-57,1658	6,46213
BTzPhOMe8	441	Done	84,97	-6,20174	glide-grid_2YXJtest	-7,21584	-1,95423	-0,45809	0	-0,09042	-55,2333	-13,575	0,269328	-0,18453	-95,0232	-68,8083	14,6503
BTzPhCl3	271	Done	80,43	-6,20136	glide-grid_2YXJtest	-6,61936	-2,25511	-0,40771	0	-0,37941	-55,9016	-6,26987	0,17803	-0,01961	-88,1674	-62,1715	4,02893
BTzPh5	223	Done	82,28	-6,20122	glide-grid_2YXJtest	-6,81282	-2,81181	-0,34397	0	-0,3711	-53,0247	-4,72384	0,27956	-0,20569	-83,107	-57,7485	12,6263
BTzPhDMN6	350	Done	62,08	-6,19954	glide-grid_2YXJtest	-6,82004	-2,52667	-0,16	0	-0,71705	-52,8909	-4,62439	0,221516	-0,29963	-81,7702	-57,5153	11,0997
BTzPhDMN5	345	Done	48,9	-6,18771	glide-grid_2YXJtest	-7,23611	-2,23818	-0,50771	0	-0,13945	-56,8523	-11,0173	0,203997	-0,05957	-93,3063	-67,8695	8,2835
BTzPhOMe5	415	Done	63,58	-6,17615	glide-grid_2YXJtest	-7,19105	-3,06994	-0,0101	0	-0,44828	-64,1547	-4,49017	0,224435	-0,00591	-90,7602	-68,6448	19,483
BTzNaf6	161	Done	49,19	-6,16788	glide-grid_2YXJtest	-6,72858	-3,70075	0	0	-0,52772	-58,1998	1,45421	0,211172	-0,01943	-82,1576	-56,7456	5,68489
BTzNaf1	130	Done	40,72	-6,16222	glide-grid_2YXJtest	-8,75942	-4,07938	-0,16	0	-0,39954	-58,8937	-7,49415	0,1043	-0,15599	-100,174	-66,3879	9,57231
BOx2	4	Done	88,68	-6,15723	glide-grid_2YXJtest	-6,15723	-3,20701	-0,09097	0	-0,14741	-51,0024	-2,78816	0,261818	-0,00531	-69,6838	-53,7905	8,79486

BTzPhOMe4	405	Done	71,24	-6,15552	glide-grid_2YXJtest	-7,16242	-2,15278	-0,68146	0	-0,10893	-49,8464	-10,8706	0,205543	-0,30188	-86,7114	-60,717	11,0143
BTzNaf6	162	Done	51,68	-6,1506	glide-grid_2YXJtest	-6,9307	-2,9996	-0,39066	0	-0,04236	-60,589	-2,9492	0,210443	-0,2367	-88,2795	-63,5382	10,8912
BTzNaf3	144	Done	59,97	-6,14134	glide-grid_2YXJtest	-8,62914	-4,29463	0	0	-0,967	-67,4231	-0,91368	0,154052	-0,01335	-100,679	-68,3368	7,41726
BTzPhCl1	263	Done	53,08	-6,14125	glide-grid_2YXJtest	-7,03875	-3,50563	0	0	-0,46094	-57,9629	-1,70667	0,126457	-0,04449	-84,4909	-59,6696	8,44565
BTzNaf1	126	Done	46,55	-6,13308	glide-grid_2YXJtest	-7,10718	-3,39814	-0,01248	0	-0,63067	-60,7223	-0,8629	0,1043	-0,00463	-89,1441	-61,5852	2,9461
BTzPh2	203	Done	68,71	-6,12832	glide-grid_2YXJtest	-7,20062	-3,62815	0	0	-0,54492	-60,5241	-1,43259	0,213536	0	-77,998	-61,9567	28,556
BTzPh2	201	Done	72,09	-6,12544	glide-grid_2YXJtest	-6,61314	-2,5435	-0,21012	0	-0,41377	-54,6766	-5,89974	0,214534	-0,04149	-84,4776	-60,5764	9,67595
BTzPhDMN9	372	Done	80,12	-6,12398	glide-grid_2YXJtest	-6,94628	-2,20101	-0,36558	0	-0,98651	-54,1246	-4,92401	0,260944	-0,20929	-85,9177	-59,0486	10,0864
BTzNaf8	179	Done	72,29	-6,12373	glide-grid_2YXJtest	-7,11453	-2,49293	-0,27045	0	-0,15101	-63,3671	-7,30098	0,238783	-0,17542	-99,0834	-70,6681	10,6728
BTzNaf3	145	Done	54,07	-6,12209	glide-grid_2YXJtest	-8,80129	-4,39692	-0,16721	0	-0,4655	-57,1681	-6,22293	0,153343	-0,13316	-95,8378	-63,391	7,36858
BTzPh1	193	Done	59,85	-6,11779	glide-grid_2YXJtest	-6,78469	-3,40522	-0,02092	0	-0,46735	-55,2345	-1,60974	0,186474	-0,07449	-80,4809	-56,8442	7,14288
BTz4	78	Done	112,46	-6,11349	glide-grid_2YXJtest	-7,05459	-2,08068	-0,48648	0	-0,49581	-49,7736	-12,0278	0,328349	-0,02713	-84,3164	-61,8014	9,89728
BTzPhOMe2	390	Done	59,21	-6,09575	glide-grid_2YXJtest	-7,63735	-3,46978	-0,16	0	-0,30646	-58,0975	-5,60614	0,15947	-0,11477	-91,6191	-63,7037	7,85924
BTzPhDMN	314	Done	35,28	-6,09551	glide-grid_2YX	-6,83911	-2,3434	-0,32	0	-0,23116	-54,001	-7,56186	0,113909	-0,2241	-88,6427	-61,5628	5,07954

1					Jtest									3			
BTzPh<br>OMe2	391	Done	64,69	-6,09132	glide-<br>grid_2YX<br>Jtest	-<br>7,79202	-<br>2,4258<br>7	-0,864	0	-0,23584	-<br>51,2625	-<br>10,8691	0,16026<br>9	-0,2331	-90,3094	-62,1315	10,9836
BTzN<br>af9	187	Done	78,48	-6,08226	glide-<br>grid_2YX<br>Jtest	-<br>7,07306	-<br>2,6448<br>9	-0,456	0	-0,15753	-<br>53,8275	-<br>8,17487	0,25074<br>7	-<br>0,1477<br>8	-92,3073	-62,0024	5,98121
BTzPh<br>DMN<br>1	315	Done	36,19	-6,06322	glide-<br>grid_2YX<br>Jtest	-<br>7,03732	-<br>3,1453<br>1	0	0	-0,74244	-<br>59,0999	-<br>1,98551	0,11390<br>9	-<br>0,0106<br>6	-84,0037	-61,0854	11,212
BTz3	70	Done	80,28	-6,04973	glide-<br>grid_2YX<br>Jtest	-<br>6,99913	-<br>2,0168<br>3	-0,68978	0	-0,57798	-<br>45,3826	-<br>11,5322	0,29965<br>3	-<br>0,0152<br>2	-80,5902	-56,9149	4,5528
BTzPh<br>DMN<br>7	360	Done	59,36	-6,0363	glide-<br>grid_2YX<br>Jtest	-7,0885	-<br>1,8059<br>6	-0,42051	0	-0,49284	-<br>58,5807	-<br>9,70354	0,23495	-<br>0,2195<br>7	-94,1597	-68,2842	10,6392
BTz7	101	Done	77,78	-6,0343	glide-<br>grid_2YX<br>Jtest	-6,9776	-<br>2,8524<br>9	-0,29692	0	-0,68976	-<br>59,4703	-<br>3,61056	0,39159<br>9	-<br>0,0149<br>2	-90,3049	-63,0809	7,64538
BTz2	62	Done	66,54	-6,02791	glide-<br>grid_2YX<br>Jtest	-<br>6,60591	-<br>2,5422<br>5	-0,13011	0	-1,13702	-<br>51,5714	-<br>3,18195	0,26461<br>1	-<br>0,0052<br>8	-71,1328	-54,7533	10,6313
BTzPh<br>Cl8	304	Done	62,71	-6,01354	glide-<br>grid_2YX<br>Jtest	-<br>7,41384	-<br>2,5156<br>4	-0,25697	0	-0,84466	-<br>60,4926	-<br>6,24784	0,26149<br>2	-<br>0,0962<br>6	-91,7759	-66,7404	10,1174
BTzPh<br>1	194	Done	53,1	-6,00838	glide-<br>grid_2YX<br>Jtest	-<br>6,75198	-<br>2,4444<br>3	-0,32	0	-0,70153	-<br>48,8761	-<br>6,20413	0,18549	-<br>0,0970<br>9	-76,0923	-55,0803	9,14687
BTzPh<br>Cl4	279	Done	63,25	-6,00639	glide-<br>grid_2YX<br>Jtest	-<br>7,14419	-<br>2,7205<br>2	-0,28986	0	-0,70498	-<br>60,4238	-<br>4,04061	0,19845<br>8	0	-88,7776	-64,4645	12,0976
BTzPh<br>5	226	Done	68,32	-6,00058	glide-<br>grid_2YX<br>Jtest	-<br>7,06448	-<br>2,6737<br>1	-0,40061	0	-0,40136	-<br>62,5895	-<br>4,15181	0,27755<br>8	-<br>0,1141<br>2	-91,7292	-66,7413	8,58403
BTzPh<br>Cl4	280	Done	62,92	-5,99997	glide-<br>grid_2YX<br>Jtest	-<br>7,38607	-<br>3,2214<br>1	-0,26967	0	-0,42879	-<br>55,1813	-<br>5,08679	0,19766<br>3	-<br>0,1417<br>7	-85,1425	-60,2681	9,33838
BTzN<br>af7	176	Done	68,86	-5,99974	glide-<br>grid_2YX<br>Jtest	-<br>8,67234	-<br>3,9173<br>9	-0,32	0	-0,48595	-69,005	-<br>4,18611	0,22467<br>7	-<br>0,0955<br>2	-108,976	-73,1911	5,24939

BTzPhOMe4	404	Done	75,47	-5,9967	glide-grid_2YXJtest	-6,652	-2,65187	-0,16626	0	-0,57089	-57,9649	-3,39765	0,206365	-0,06146	-83,1631	-61,3625	15,1392
BTz6	96	Done	94,16	-5,99413	glide-grid_2YXJtest	-8,35183	-3,00094	-0,32	0	-1,17233	-58,4578	-8,35631	0,372355	-0,05457	-95,5984	-66,8141	9,64783
BTzPhDMN1	316	Done	34,83	-5,98922	glide-grid_2YXJtest	-7,24602	-2,82556	-0,29855	0	-0,64844	-58,0095	-4,52963	0,1132	-0,00675	-87,6437	-62,5391	8,78987
BTzPhOMe1	378	Done	42,85	-5,98534	glide-grid_2YXJtest	-6,67144	-3,46908	0	0	-0,44543	-55,8714	-0,36831	0,133987	-0,0421	-75,7948	-56,2397	12,8482
BTzPhCl2	267	Done	62,92	-5,98269	glide-grid_2YXJtest	-6,30139	-2,61795	-0,20756	0	-0,35376	-57,3407	-2,6789	0,154136	-0,00738	-81,4748	-60,0196	10,5842
BTzNaf4	147	Done	50	-5,94701	glide-grid_2YXJtest	-6,72801	-3,1268	-0,1918	0	-0,29358	-49,7318	-3,63942	0,174997	-0,25834	-79,4683	-53,3712	3,26409
BTz2	64	Done	65,72	-5,94338	glide-grid_2YXJtest	-6,88698	-2,34471	-0,46586	0	-1,1432	-48,966	-4,97135	0,26605	-0,00526	-74,858	-53,9374	8,08358
BTzPh2	206	Done	71,45	-5,93893	glide-grid_2YXJtest	-8,40963	-4,04938	-0,16	0	-0,56107	-58,0686	-5,30802	0,213536	-0,15308	-93,4096	-63,3766	11,5524
BTzPhDMN7	359	Done	53,35	-5,93245	glide-grid_2YXJtest	-6,97885	-2,18766	-0,58805	0	-0,04347	-48,8371	-10,8453	0,235707	-0,32675	-86,2197	-59,6823	10,2317
BTzPh5	225	Done	62,98	-5,92707	glide-grid_2YXJtest	-6,97547	-2,98202	-0,06265	0	-0,3615	-62,0944	-4,90988	0,278558	-0,00665	-94,9516	-67,0042	6,78575
BTzPh4	217	Done	74,77	-5,91938	glide-grid_2YXJtest	-6,90288	-2,66849	-0,31418	0	-0,24402	-57,8752	-6,65507	0,25966	-0,04384	-89,2982	-64,5302	8,91294
BTzPhDMN3	331	Done	61,71	-5,91497	glide-grid_2YXJtest	-8,27997	-3,48968	-0,28789	0	-0,42601	-60,9526	-5,83555	0,164133	-0,31756	-100,741	-66,7882	6,12271
BTz9	118	Done	69,11	-5,9024	glide-grid_2YXJtest	-6,8457	-2,59037	-0,48	0	-0,23946	-43,0105	-10,165	0,419624	-0,28021	-75,3409	-53,1756	7,95413
BTzPhCl8	305	Done	64,94	-5,89958	glide-grid_2YX	-8,44588	-3,6382	-0,32	0	-0,3715	-61,2997	-7,76676	0,262284	-0,1483	-108,456	-69,0665	6,55934

					Jtest		9							8			
BTz3	69	Done	76,89	-5,89234	glide-<br>grid_2YX<br>Jtest	-<br>6,82284	-<br>2,4360<br>5	-0,42989	0	-1,12531	-<br>51,1488	-<br>3,78912	0,29965<br>3	-<br>0,0054<br>3	-78,5588	-54,9379	4,61401
BTzPh<br>OMe3	395	Done	68,67	-5,88605	glide-<br>grid_2YX<br>Jtest	-<br>6,56165	-<br>2,4354<br>2	-0,30451	0	-0,45525	-<br>55,6139	-<br>5,06848	0,18507<br>4	-<br>0,0105<br>7	-86,6427	-60,6824	3,65661
BOxN<br>af7	18	Done	59,23	-5,88035	glide-<br>grid_2YX<br>Jtest	-<br>5,88035	-<br>2,7215<br>9	0	0	-0,10046	-<br>57,4112	-<br>2,32828	0,22326<br>4	-<br>0,0617<br>6	-74,1298	-59,7395	13,6474
BTzPh<br>DMN<br>6	351	Done	66,09	-5,86649	glide-<br>grid_2YX<br>Jtest	-<br>6,68879	-<br>3,0809<br>1	-0,16	0	-0,71171	-<br>59,8248	0,64669<br>5	0,22075<br>6	-<br>0,0626<br>9	-86,6654	-59,1781	4,59581
BTz8	110	Done	69,32	-5,8656	glide-<br>grid_2YX<br>Jtest	-6,8089	-<br>2,2698	-0,43183	0	-0,91283	-<br>49,4273	-<br>6,09716	0,40676<br>4	-<br>0,2152<br>7	-79,1707	-55,5244	9,64316
BTzPh<br>OMe3	396	Done	67,94	-5,86266	glide-<br>grid_2YX<br>Jtest	-<br>6,85896	-<br>2,4429<br>5	-0,2884	0	-0,43154	-59,071	-<br>6,15878	0,18426	-<br>0,0029<br>7	-89,6247	-65,2297	8,56881
BOxP<br>h9	29	Done	74,82	-5,84122	glide-<br>grid_2YX<br>Jtest	-<br>5,84122	-<br>2,1745<br>6	0	0	-0,21687	-<br>56,8843	-<br>4,98287	0,33067	-<br>0,1888<br>2	-82,1574	-61,8672	7,87101
BTzPh<br>OMe1	385	Done	38,49	-5,83694	glide-<br>grid_2YX<br>Jtest	-<br>7,81134	-<br>3,5029<br>3	-0,16	0	-0,43026	-<br>59,1087	-<br>5,28278	0,13242<br>3	-<br>0,1027<br>3	-90,0416	-64,3915	13,3942
BTzPh<br>3	211	Done	86,92	-5,83574	glide-<br>grid_2YX<br>Jtest	-<br>8,20074	-<br>3,7746<br>1	-0,16753	0	-0,78285	-55,198	-5,9232	0,23811<br>2	-<br>0,0654<br>9	-90,8844	-61,1212	8,25077
BTzN<br>af2	135	Done	62,49	-5,82221	glide-<br>grid_2YX<br>Jtest	-<br>7,04901	-<br>3,2221<br>1	-0,16	0	-0,19182	-<br>52,5929	-5,5098	0,1299	-<br>0,1488<br>7	-85,1378	-58,1027	2,65055
BTzPh<br>Cl2	269	Done	65,52	-5,8165	glide-<br>grid_2YX<br>Jtest	-6,9798	-<br>2,4420<br>5	-0,53524	0	-0,37261	-<br>57,0588	-<br>6,02714	0,15335<br>9	-<br>0,0262<br>5	-88,0598	-63,0859	9,66988
BTzN<br>af7	171	Done	57,36	-5,79542	glide-<br>grid_2YX<br>Jtest	-<br>6,78622	-<br>2,7130<br>3	-0,31185	0	-0,25389	-<br>58,3633	-<br>4,05652	0,22540<br>4	-<br>0,2062<br>1	-87,7104	-62,4198	7,13522
BTzPh<br>OMe7	437	Done	67,96	-5,78026	glide-<br>grid_2YX<br>Jtest	-<br>7,78216	-<br>1,7600<br>3	-0,66555	0	-0,37498	-<br>61,5996	-<br>13,2484	0,25528<br>6	-<br>0,1696<br>4	-102,088	-74,848	13,5657

BTzNaf4	149	Done	51,14	-5,77551	glide-grid_2YXJtest	-7,21251	-2,26751	-0,4199	0	-0,40995	-52,595	-10,3798	0,174279	-0,10272	-88,1123	-62,9748	8,76689
BTzPh6	233	Done	69,78	-5,77176	glide-grid_2YXJtest	-7,21996	-2,45818	-0,28861	0	-0,67627	-50,5777	-9,17478	0,294136	-0,18593	-85,3732	-59,7525	6,551
BTzPhOMe3	398	Done	69,33	-5,77026	glide-grid_2YXJtest	-7,10826	-2,52609	-0,5582	0	-0,41157	-59,3928	-5,43331	0,183449	-0,0112	-90,9344	-64,8261	7,8517
BOx1	3	Done	70,05	-5,76314	glide-grid_2YXJtest	-5,76314	-2,49131	0	0	-0,23131	-41,7001	-6,78729	0,222507	-0,15993	-60,9623	-48,4874	11,4505
BTzNaf8	184	Done	77,02	-5,76011	glide-grid_2YXJtest	-8,43271	-3,56484	-0,37936	0	-0,54684	-60,531	-6,87293	0,238059	-0,12223	-94,4587	-67,4039	20,0662
BTzPhCl1	264	Done	48,84	-5,75626	glide-grid_2YXJtest	-6,73786	-2,38437	-0,35271	0	-0,12376	-50,4071	-7,40041	0,126457	-0,37305	-82,2914	-57,8075	9,28896
BTzPhOMe2	392	Done	43,26	-5,74144	glide-grid_2YXJtest	-7,44794	-3,19773	-0,01758	0	-0,44599	-59,5895	-6,42701	0,160269	-0,00338	-89,8957	-66,0165	14,9559
BTzPhDMN2	323	Done	37,55	-5,73628	glide-grid_2YXJtest	-6,80858	-2,01735	-0,39086	0	-0,12692	-47,9213	-10,5318	0,14048	-0,43811	-83,7951	-58,453	9,7407
BTzPhCl7	297	Done	67,35	-5,73466	glide-grid_2YXJtest	-6,88036	-2,35649	-0,16	0	-0,74765	-60,5884	-5,06808	0,249015	-0,07561	-91,4845	-65,6565	8,49758
BTzPh7	241	Done	68,26	-5,72169	glide-grid_2YXJtest	-7,16989	-2,62255	-0,16	0	-0,36205	-67,8001	-5,75201	0,308669	-0,08115	-104,286	-73,5521	5,50219
BTz5	87	Done	101,73	-5,719	glide-grid_2YXJtest	-7,3027	-2,65688	-0,32	0	-1,2618	-57,4741	-3,61037	0,354295	-0,00305	-88,6205	-61,0845	9,47859
BTzPhOMe4	407	Done	56,17	-5,71411	glide-grid_2YXJtest	-7,08141	-2,59322	-0,608	0	-0,13674	-50,5452	-9,02233	0,204725	-0,06756	-83,5764	-59,5675	7,86858
BTzPhOMe6	426	Done	65,1	-5,70795	glide-grid_2YXJtest	-7,34385	-2,242	-0,72	0	-0,14748	-57,2997	-9,24244	0,241209	-0,22422	-97,6298	-66,5422	8,22274
BTzPhOMe3	400	Done	60,84	-5,70491	glide-grid_2YX	-7,35701	-2,6798	-0,39174	0	-0,1695	-60,2923	-8,45194	0,18426	-0,0178	-94,5415	-68,7442	12,2224

					Jtest		1							3			
BTzPhDMN8	365	Done	68	-5,69293	glide-grid_2YXJtest	-6,51523	-2,25367	-0,32	0	-0,10238	-58,7363	-6,26931	0,249046	-0,21101	-91,2289	-65,0056	8,55348
BTzPhCl6	292	Done	53,67	-5,6885	glide-grid_2YXJtest	-7,0888	-3,19764	-0,32	0	-0,42504	-58,0457	-2,52103	0,233296	-0,09898	-84,3114	-60,5667	4,12214
BTzPh3	212	Done	79,9	-5,67647	glide-grid_2YXJtest	-8,15727	-3,34111	-0,32	0	-0,9704	-62,4375	-4,18298	0,237111	-0,01354	-93,7587	-66,6205	7,58862
BTzPh5	224	Done	70,77	-5,67354	glide-grid_2YXJtest	-6,49724	-2,58975	-0,32	0	-0,51992	-57,3542	-2,97655	0,278558	-0,03194	-83,2773	-60,3308	6,62022
BTzPhCl6	290	Done	61,61	-5,66601	glide-grid_2YXJtest	-6,56561	-1,69645	-0,46844	0	0	-43,2028	-14,4619	0,234095	-0,30539	-85,193	-57,6647	7,16655
BTzPhOMe7	433	Done	64,5	-5,66284	glide-grid_2YXJtest	-6,67694	-2,03918	-0,53439	0	-0,09948	-56,9809	-7,56138	0,256104	-0,27674	-88,7766	-64,5423	8,23303
BTzPhDMN6	356	Done	54,21	-5,65991	glide-grid_2YXJtest	-8,24011	-3,17342	-0,4531	0	-0,45764	-58,7925	-8,41775	0,219998	-0,17366	-97,7008	-67,2102	8,33591
BTzPhOMe3	399	Done	68,81	-5,65941	glide-grid_2YXJtest	-7,28541	-3,35144	0	0	-0,95723	-61,2729	-0,60319	0,18426	-0,00688	-89,3983	-61,8761	9,46292
BTzPh2	202	Done	80,87	-5,64747	glide-grid_2YXJtest	-6,50537	-2,39065	-0,30243	0	-0,38813	-54,4108	-5,85721	0,213536	-0,03858	-78,1143	-60,268	16,8666
BTzPh8	250	Done	64,31	-5,63949	glide-grid_2YXJtest	-8,01999	-3,347	-0,23994	0	-0,37175	-54,656	-9,80262	0,322369	-0,18048	-98,8324	-64,4586	6,84302
BTzPhOMe1	379	Done	46,46	-5,63477	glide-grid_2YXJtest	-6,66297	-2,28924	-0,32	0	-0,241	-51,7872	-7,49316	0,133203	-0,2326	-83,5292	-59,2804	9,61972
BTzNaf2	137	Done	50,35	-5,62918	glide-grid_2YXJtest	-8,09988	-3,66377	-0,232	0	-0,5388	-56,386	-5,83122	0,130594	-0,10192	-90,0453	-62,2172	5,43805
BTzPhCl1	265	Done	50,53	-5,61931	glide-grid_2YXJtest	-6,77661	-2,40623	-0,42107	0	-0,13891	-50,4491	-7,77298	0,125702	-0,24771	-81,4849	-58,2221	6,22999

BTzPhDMN9	373	Done	65,06	-5,61709	glide-grid_2YXJtest	-6,66349	-2,68945	-0,16	0	-0,81456	-48,4624	-4,56802	0,260944	-0,1521	-77,6235	-53,0304	3,51385
BTz9	120	Done	61,04	-5,61297	glide-grid_2YXJtest	-7,96777	-2,36087	-0,57913	0	-1,05268	-63,7325	-7,97105	0,41828	-0,0111	-101,26	-71,7035	10,8863
BTzNaf9	191	Done	66,27	-5,59184	glide-grid_2YXJtest	-8,12394	-2,71244	-0,7596	0	-0,27557	-64,0392	-8,98325	0,250027	-0,07692	-105,75	-73,0224	4,54641
BTzNaf3	143	Done	60,59	-5,58783	glide-grid_2YXJtest	-8,06863	-2,89189	-0,32	0	-0,86387	-66,6282	-5,30322	0,153343	-0,01932	-97,7776	-71,9315	15,6356
BTzNaf8	178	Done	59,86	-5,58329	glide-grid_2YXJtest	-6,36339	-2,51859	-0,16	0	-0,20375	-55,1426	-5,78974	0,238783	-0,09424	-83,8103	-60,9324	7,74184
BTz7	102	Done	76,54	-5,57995	glide-grid_2YXJtest	-6,52325	-1,8533	-0,48	0	-0,87232	-41,8333	-8,77272	0,391599	-0,30166	-73,5379	-50,606	7,07397
BTz5	86	Done	93,86	-5,56635	glide-grid_2YXJtest	-6,50405	-2,62664	-0,26641	0	-0,74846	-48,0461	-4,86784	0,352856	-0,08292	-72,9482	-52,9139	7,16402
BTzNaf5	157	Done	55,35	-5,55282	glide-grid_2YXJtest	-6,61672	-2,6325	0	0	-0,70345	-61,5991	-1,83712	0,192988	-0,11823	-86,2948	-63,4362	9,40806
BTzPhCl6	289	Done	63,14	-5,55167	glide-grid_2YXJtest	-5,95057	-2,02503	-0,48	0	-0,0714	-38,8735	-8,6798	0,234897	-0,36339	-66,0839	-47,5533	12,8192
BTzPh7	244	Done	59,14	-5,54929	glide-grid_2YXJtest	-8,08139	-3,51577	-0,16	0	-0,57245	-65,8755	-4,99379	0,308669	-0,099	-101,18	-70,8693	11,2749
BTzPhDMN4	336	Done	59,46	-5,54238	glide-grid_2YXJtest	-6,32338	-2,51128	-0,16	0	-0,42621	-59,2331	-1,99824	0,185208	-0,14972	-84,0067	-61,2313	8,78645
BTzPhOMe5	414	Done	66,99	-5,52967	glide-grid_2YXJtest	-6,54457	-2,36761	-0,67549	0	-0,18811	-49,9535	-5,08455	0,224435	-0,27743	-80,3621	-55,038	4,47854
BTzPhDMN8	369	Done	61,93	-5,5203	glide-grid_2YXJtest	-8,0294	-2,54631	-0,69345	0	-0,13232	-57,2468	-11,4618	0,249046	-0,32475	-101,504	-68,7087	10,3259
BTzPh4	221	Done	58,43	-5,51338	glide-grid_2YX	-8,03678	-2,933	-0,34181	0	-0,51464	-59,0032	-9,30161	0,258658	-0,1605	-101,041	-68,3048	6,00205

					Jtest									8			
BTzPh<br>8	248	Done	70,85	-5,50771	glide-<br>grid_2YX<br>Jtest	-<br>6,49851	-<br>2,4345<br>2	-0,1507	0	-0,59812	-<br>60,6321	-<br>3,49616	0,32236<br>9	-<br>0,0815<br>1	-87,6489	-64,1283	9,72909
BTzPh<br>DMN<br>9	371	Done	66,74	-5,48703	glide-<br>grid_2YX<br>Jtest	-<br>6,10753	-<br>3,0145<br>5	0	0	-0,26576	-<br>55,6953	0,70027	0,26169<br>4	-<br>0,4091<br>9	-77,4856	-54,995	10,2158
BTzPh<br>OMe4	408	Done	63,19	-5,48556	glide-<br>grid_2YX<br>Jtest	-<br>7,11926	-<br>3,0375<br>8	0	0	-0,67576	-<br>68,1444	-<br>1,36161	0,20554<br>3	0	-94,2379	-69,506	16,2474
BTzPh<br>DMN<br>2	324	Done	47,67	-5,48002	glide-<br>grid_2YX<br>Jtest	-<br>6,70682	-<br>2,9348<br>1	-0,12967	0	-0,40851	-<br>58,3463	-<br>2,80734	0,13975<br>1	-<br>0,0351<br>6	-83,2829	-61,1537	6,97006
BTzN<br>af5	155	Done	57,47	-5,46398	glide-<br>grid_2YX<br>Jtest	-<br>6,28768	-<br>2,4514<br>2	-0,19957	0	-0,27278	-<br>60,4145	-<br>2,10073	0,19371<br>2	-<br>0,2217<br>9	-86,0256	-62,5153	6,36761
BTzPh<br>Cl7	296	Done	53,48	-5,45306	glide-<br>grid_2YX<br>Jtest	-<br>6,35266	-<br>2,4843<br>2	-0,32	0	-0,20683	-<br>54,4483	-<br>5,39086	0,24901<br>5	-<br>0,0594<br>8	-80,6975	-59,8392	10,4578
BTzPh<br>Cl5	284	Done	69,79	-5,43401	glide-<br>grid_2YX<br>Jtest	-<br>6,33771	-<br>2,5424<br>9	0	0	-0,52488	-<br>62,6036	-<br>2,31452	0,21732	-<br>0,0103<br>1	-87,8137	-64,9181	9,85631
BTzPh<br>OMe6	429	Done	75,42	-5,42985	glide-<br>grid_2YX<br>Jtest	-<br>7,43175	-<br>2,8833<br>6	-0,37843	0	-0,2183	-49,295	-9,5566	0,24038<br>8	-0,2938	-84,3748	-58,8516	9,72911
BTzN<br>af7	173	Done	62,23	-5,42036	glide-<br>grid_2YX<br>Jtest	-<br>7,80086	-<br>2,9911<br>3	-0,60233	0	-0,47759	-<br>51,9369	-<br>8,38053	0,22540<br>4	-<br>0,1012<br>9	-93,8327	-60,3175	4,77918
BTz5	85	Done	85,52	-5,41278	glide-<br>grid_2YX<br>Jtest	-<br>6,34838	-<br>2,2543<br>1	-0,456	0	-0,24423	-<br>42,4981	-<br>9,89532	0,35285<br>6	-0,1375	-73,3873	-52,3934	3,43222
BTzPh<br>DMN<br>7	357	Done	73,32	-5,41012	glide-<br>grid_2YX<br>Jtest	-<br>6,03062	-<br>1,7909<br>1	-0,66741	0	-0,14322	-<br>43,9744	-<br>8,03561	0,23646<br>7	-<br>0,2614<br>9	-73,9908	-52,0101	10,0089
BTzPh<br>7	240	Done	64,52	-5,4039	glide-<br>grid_2YX<br>Jtest	-6,3947	-<br>2,1426<br>8	-0,16102	0	-0,66747	-<br>55,1261	-<br>6,32541	0,30965<br>2	-<br>0,0280<br>8	-82,3191	-61,4515	13,776
BTz3	71	Done	81,85	-5,38453	glide-<br>grid_2YX<br>Jtest	-<br>7,01653	-<br>1,9995<br>1	-0,7055	0	-0,52714	-<br>43,3388	-<br>12,6936	0,30110<br>6	-<br>0,0145<br>1	-80,9351	-56,0324	7,67463

BTzPh9	260	Done	62,48	-5,37467	glide-grid_2YXJtest	-7,90677	-3,11759	-0,16	0	-0,75516	-69,054	-4,39038	0,332533	-0,09529	-104,794	-73,4444	8,1615
BTz7	103	Done	79,94	-5,37375	glide-grid_2YXJtest	-6,97415	-3,22731	-0,12609	0	-1,03492	-58,9197	-0,17464	0,393	-0,00664	-84,5536	-59,0943	11,3259
BTzNaf9	188	Done	70,06	-5,36712	glide-grid_2YXJtest	-6,81532	-3,29284	-0,27554	0	-0,19931	-55,6121	-2,35306	0,250027	-0,1641	-84,9849	-57,9652	5,81961
BOxPhOMe9	56	Done	89,1	-5,3669	glide-grid_2YXJtest	-5,3669	-1,60348	-0,19377	0	-0,33917	-56,8817	-3,44694	0,278691	-0,14804	-73,4505	-60,3287	16,6883
BTzPhDMN2	322	Done	54,25	-5,36083	glide-grid_2YXJtest	-6,21873	-1,97521	-0,30939	0	-0,30382	-53,7793	-6,80692	0,14048	-0,06079	-81,2352	-60,5862	8,22889
BTzPh8	253	Done	69,37	-5,35254	glide-grid_2YXJtest	-8,02514	-3,84388	-0,12019	0	-0,75603	-70,2529	-0,66982	0,321398	-0,01332	-99,5142	-70,9227	13,4863
BTzPhOMe1	380	Done	40,95	-5,35206	glide-grid_2YXJtest	-6,41926	-1,90002	-0,456	0	-0,10417	-51,308	-9,01942	0,133203	-0,17396	-82,6292	-60,3275	11,2389
BTzPh2	204	Done	64,27	-5,34826	glide-grid_2YXJtest	-6,57506	-2,15553	-0,456	0	-0,01994	-46,8942	-10,6166	0,212541	-0,21893	-73,9658	-57,5108	17,4987
BTzNaf1	124	Done	47,17	-5,34805	glide-grid_2YXJtest	-6,01495	-2,85358	0	0	-0,25261	-54,1275	-1,87649	0,104976	-0,02589	-77,5893	-56,004	6,67396
BTzPh4	220	Done	58,9	-5,3289	glide-grid_2YXJtest	-7,7873	-2,8012	-0,48	0	-0,19582	-57,1598	-11,2019	0,25966	-0,03166	-96,6352	-68,3618	13,1811
BTzPhOMe4	409	Done	59,79	-5,3083	glide-grid_2YXJtest	-6,9527	-2,42148	-0,48	0	-0,0585	-47,2301	-11,2739	0,205543	-0,14567	-78,9056	-58,504	16,8914
BTzNaf9	186	Done	64,86	-5,29919	glide-grid_2YXJtest	-6,07929	-1,78719	-0,16	0	-0,30465	-61,3397	-5,35405	0,250747	-0,20811	-85,9528	-66,6938	17,0766
BTz4	76	Done	92,75	-5,29878	glide-grid_2YXJtest	-5,75878	-1,85664	-0,60555	0	-0,02598	-31,9855	-11,7447	0,326906	-0,23653	-60,4267	-43,7303	4,42798
BT9	9	Done	65,31	-5,29481	glide-grid_2YX	-5,29481	-1,9520	-0,00304	0	-0,55209	-55,4935	-1,82068	0,337282	-0,0771	-73,5892	-57,3142	9,0026

					Jtest		1							8			
BTzPhDMN4	335	Done	63,2	-5,28988	glide-grid_2YXJtest	-5,85658	-1,92582	-0,40134	0	0	-34,7422	-11,0018	0,185962	-0,32801	-66,1779	-45,744	6,69019
BTzPhOMe7	435	Done	68,87	-5,28615	glide-grid_2YXJtest	-6,92205	-2,67007	-0,31623	0	-0,01949	-49,7218	-9,75591	0,256104	-0,22288	-81,3503	-59,4777	14,4384
BTzPhOMe8	445	Done	86,65	-5,27463	glide-grid_2YXJtest	-6,91053	-1,86947	-0,49116	0	-0,52648	-52,1289	-10,6491	0,269328	-0,08894	-85,7074	-62,7781	16,0234
BTzNaf5	156	Done	52,45	-5,27325	glide-grid_2YXJtest	-6,32165	-2,36779	-0,32	0	-0,16323	-47,378	-6,49447	0,193712	-0,32128	-77,1804	-53,8724	4,64231
BTz9	117	Done	64,94	-5,26827	glide-grid_2YXJtest	-6,21157	-2,35175	-0,36585	0	-0,68868	-56,7493	-2,52677	0,419624	-0,00843	-81,136	-59,2761	9,44445
BTzPh8	252	Done	57,36	-5,26518	glide-grid_2YXJtest	-7,79728	-3,54369	-0,10557	0	-0,58562	-62,6225	-4,37113	0,321398	-0,09701	-96,8019	-66,9936	6,85991
BTz6	97	Done	97,94	-5,25024	glide-grid_2YXJtest	-7,60794	-2,59276	-0,608	0	-0,87826	-52,8935	-7,8655	0,372355	-0,07677	-86,1777	-60,759	5,6526
BTzPhCl9	310	Done	77,06	-5,24855	glide-grid_2YXJtest	-6,64885	-1,28888	-0,57905	0	-0,13565	-54,7228	-12,2399	0,273295	-0,34646	-92,8014	-66,9627	8,36928
BTzPh7	239	Done	68,42	-5,24801	glide-grid_2YXJtest	-6,02811	-1,74748	-0,34144	0	-0,2306	-49,7908	-7,97509	0,309652	-0,33244	-76,1968	-57,7659	11,3099
BTz4	83	Done	103,74	-5,24255	glide-grid_2YXJtest	-7,78415	-2,94714	-0,47305	0	-1,11779	-56,8323	-4,64373	0,326906	-0,0349	-87,6978	-61,476	10,4012
BTzPh8	249	Done	59,52	-5,24065	glide-grid_2YXJtest	-6,68885	-3,2857	-0,01847	0	-0,116	-65,497	-1,14218	0,321398	-0,1439	-90,764	-66,6392	6,56674
BTzPhOMe2	394	Done	55,92	-5,23958	glide-grid_2YXJtest	-7,45548	-2,80853	-0,60418	0	-0,58822	-53,3934	-5,75035	0,15947	-0,0818	-87,0856	-59,1437	5,99252
BTzNaf7	169	Done	63,49	-5,23739	glide-grid_2YXJtest	-5,79809	-1,75838	-0,44856	0	-0,2077	-55,6166	-3,89897	0,226133	-0,2439	-82,7354	-59,5156	4,40303

BTzNaf9	189	Done	71,24	-5,23525	glide-grid_2YXJtest	-7,61575	-3,37663	-0,04062	0	-0,41182	-74,3927	-0,71762	0,250747	-0,21015	-107,625	-75,1104	15,8034
BTzPhOMe2	393	Done	58,2	-5,23288	glide-grid_2YXJtest	-7,44418	-2,17523	-0,88205	0	-0,20439	-52,5647	-10,2539	0,15947	-0,17566	-86,99	-62,8186	8,42318
BTzPhOMe2	388	Done	61,77	-5,22854	glide-grid_2YXJtest	-6,25944	-2,1644	-0,52241	0	-0,18893	-50,8452	-5,06095	0,160269	-0,24256	-77,8326	-55,9061	8,03012
BTzPhCl4	277	Done	65,43	-5,2149	glide-grid_2YXJtest	-5,6188	-1,95731	-0,32981	0	0	-30,2729	-11,6921	0,199256	-0,26347	-62,7187	-41,9651	5,6023
BTz9	122	Done	63,34	-5,21225	glide-grid_2YXJtest	-7,75725	-3,71757	-0,16	0	-0,46497	-62,6373	-4,16173	0,41828	-0,07686	-94,088	-66,7991	9,58165
BTzPhDMN3	333	Done	48	-5,2074	glide-grid_2YXJtest	-7,6952	-2,27574	-0,62436	0	-0,45975	-57,5454	-10,6498	0,164133	-0,02475	-97,7422	-68,1952	5,79387
BTzPhDMN9	375	Done	67,41	-5,19836	glide-grid_2YXJtest	-7,63276	-2,06546	-0,65223	0	-0,55274	-52,3678	-11,8463	0,260944	-0,22794	-93,828	-64,2141	7,53585
BTzPhDMN4	338	Done	49,87	-5,19662	glide-grid_2YXJtest	-6,63362	-2,49982	-0,20848	0	-0,15579	-57,3851	-5,62924	0,184456	-0,24033	-84,9143	-63,0144	7,37468
BTzNaf9	190	Done	62,24	-5,1965	glide-grid_2YXJtest	-7,6472	-2,66852	-0,5549	0	-0,13196	-53,1795	-11,641	0,250747	-0,13743	-95,059	-64,8205	9,13317
BTzPh7	245	Done	59,94	-5,18854	glide-grid_2YXJtest	-7,86114	-3,46551	-0,2968	0	-0,67158	-55,9527	-5,15591	0,308669	-0,1649	-93,4029	-61,1086	5,79775
BTzPhOMe9	452	Done	91,05	-5,17634	glide-grid_2YXJtest	-6,55654	-2,28837	-0,32	0	-0,06431	-62,0829	-6,29577	0,280258	-0,1156	-91,4487	-68,3787	11,0284
BTzPhOMe4	411	Done	59,98	-5,1672	glide-grid_2YXJtest	-7,1632	-3,07355	-0,16	0	-0,32195	-57,2915	-5,44639	0,204725	-0,13089	-83,841	-62,7379	12,6477
BTzPhCl3	274	Done	74,2	-5,16532	glide-grid_2YXJtest	-6,51642	-2,39512	-0,28219	0	-0,44048	-54,6898	-5,53751	0,176453	-0,00998	-84,1045	-60,2273	2,28636
BTzPh4	215	Done	66,05	-5,16452	glide-grid_2YX	-5,73122	-2,4957	-0,05103	0	-0,30127	-53,4595	-2,19443	0,260666	-0,1416	-75,3147	-55,654	10,2928

					Jtest		7							7			
BTz5	89	Done	89,52	-5,1634	glide-grid_2YXJtest	-7,5298	-2,28344	-0,456	0	-0,68196	-48,8959	-11,6509	0,351423	-0,2674	-86,2445	-60,5467	6,7746
BTz8	113	Done	89,03	-5,15704	glide-grid_2YXJtest	-7,51184	-2,28813	-0,59478	0	-0,50043	-51,7438	-11,339	0,405393	-0,24585	-89,5862	-63,0828	10,5599
BTzPhOMe5	420	Done	58,35	-5,14238	glide-grid_2YXJtest	-7,15888	-2,20183	-0,52458	0	-0,2236	-55,7105	-9,77736	0,223613	-0,18035	-89,5984	-65,4879	12,4722
BTz5	88	Done	92,29	-5,14053	glide-grid_2YXJtest	-7,50553	-2,76879	-0,33411	0	-0,81198	-54,113	-6,68156	0,351423	-0,23419	-86,9379	-60,7946	8,17309
BTzPhDMN4	339	Done	53,36	-5,13708	glide-grid_2YXJtest	-7,51438	-2,3991	-0,456	0	-0,33448	-51,3665	-10,694	0,185208	-0,33759	-90,5028	-62,0605	10,2643
BTzPhDMN8	370	Done	70,3	-5,1302	glide-grid_2YXJtest	-7,7104	-2,36063	-0,28051	0	-0,10297	-51,822	-15,3335	0,248293	-0,32347	-98,1228	-67,1555	10,2479
BTzNaf8	183	Done	58,96	-5,12804	glide-grid_2YXJtest	-7,66014	-3,28467	-0,2316	0	-0,42051	-56,1682	-6,42837	0,238059	-0,18875	-90,741	-62,5966	7,13401
BTzPhCl7	295	Done	63,84	-5,12394	glide-grid_2YXJtest	-5,52284	-2,34958	-0,13422	0	-0,10175	-54,544	-2,75209	0,249814	-0,0471	-76,064	-57,2961	9,88348
BTzNaf2	136	Done	46,69	-5,12377	glide-grid_2YXJtest	-7,58867	-2,35013	-0,58123	0	-0,25897	-54,811	-10,0689	0,130594	-0,27805	-94,2642	-64,8799	9,9413
BTzPh8	247	Done	71,89	-5,11811	glide-grid_2YXJtest	-5,89821	-1,41729	-0,456	0	-0,21838	-50,0425	-9,6141	0,322369	-0,18468	-79,1357	-59,6566	9,8682
BTzPhDMN6	353	Done	60,18	-5,11288	glide-grid_2YXJtest	-6,16508	-1,7963	-0,16	0	0	-55,2431	-9,20002	0,219998	-0,28662	-87,7255	-64,4431	5,57182
BTzPh4	218	Done	82,84	-5,10495	glide-grid_2YXJtest	-6,54195	-2,00902	-0,5106	0	-0,25889	-51,406	-8,66989	0,258658	-0,15131	-80,0156	-60,0759	9,38701
BTzNaf8	177	Done	72,55	-5,09948	glide-grid_2YXJtest	-5,66018	-1,86533	-0,16	0	-0,18234	-49,4714	-7,18694	0,23951	-0,14041	-78,1339	-56,6583	6,16301

BTzPhCl1	262	Done	52,69	-5,091	glide-grid_2YXJtest	-5,595	-2,237	-0,28487	0	-0,33357	-50,9456	-2,04045	0,127216	-0,01343	-70,0361	-52,9861	6,92811
BTzPhOMe8	442	Done	62,85	-5,08441	glide-grid_2YXJtest	-6,09851	-2,75826	-0,14232	0	-0,03802	-53,9698	-3,39078	0,269328	-0,22213	-79,488	-57,3605	12,6369
BTzNaf1	128	Done	40,69	-5,0822	glide-grid_2YXJtest	-7,3869	-3,36015	-0,16	0	-0,57814	-63,1401	-1,32795	0,1043	-0,03672	-91,7888	-64,468	8,29713
BTzPhOMe1	386	Done	43,5	-5,06699	glide-grid_2YXJtest	-7,62099	-3,44309	-0,16	0	-0,44661	-56,0015	-5,2575	0,132423	-0,115	-86,6841	-61,259	10,4849
BTzPhDMN1	320	Done	39,31	-5,06019	glide-grid_2YXJtest	-7,68729	-3,26948	-0,16	0	-0,69701	-61,1009	-3,45051	0,1132	-0,10139	-89,447	-64,5514	10,4447
BTz2	63	Done	71,16	-5,05739	glide-grid_2YXJtest	-5,85739	-2,52481	-0,16	0	-0,47802	-45,5644	-4,26699	0,26605	-0,04235	-63,9486	-49,8314	13,5691
BTzPhCl6	293	Done	65,87	-5,05216	glide-grid_2YXJtest	-7,59846	-3,78396	-0,16	0	-0,61108	-56,8386	-2,4959	0,234095	-0,0612	-87,7303	-59,3345	11,142
BTzPhOMe6	427	Done	69,22	-5,052	glide-grid_2YXJtest	-6,6879	-2,72158	-0,3923	0	-0,52798	-47,0332	-5,26408	0,241209	-0,14599	-77,1908	-52,2973	4,56492
BTz7	106	Done	80,03	-5,0428	glide-grid_2YXJtest	-7,5878	-2,08334	-0,6921	0	-0,62352	-50,9442	-12,186	0,390204	-0,20393	-91,6638	-63,1301	7,64888
BTzNaf3	142	Done	62,56	-5,03313	glide-grid_2YXJtest	-7,39813	-3,22857	0	0	-0,81254	-67,1944	-1,00102	0,154052	-0,00119	-98,2249	-68,1954	5,94153
BTz5	91	Done	84,49	-5,02951	glide-grid_2YXJtest	-7,58271	-3,08342	-0,02912	0	-1,34174	-58,398	-3,31777	0,351423	-0,06229	-87,4435	-61,7158	9,47782
BTzNaf5	159	Done	65,42	-5,0263	glide-grid_2YXJtest	-7,5405	-3,24542	-0,456	0	-0,09044	-56,1009	-6,58488	0,193712	-0,14958	-93,9949	-62,6858	8,28772
BTzPhOMe8	443	Done	68,03	-5,01809	glide-grid_2YXJtest	-6,39829	-1,55941	-0,456	0	-0,20343	-54,7093	-10,0786	0,268515	-0,20071	-83,6912	-64,7879	15,5442
BTzPhDMN	317	Done	30,47	-5,00733	glide-grid_2YX	-7,31203	-3,4737	-0,04807	0	-0,74836	-60,2729	-0,46047	0,113909	-0,0730	-85,7832	-60,7334	6,1191

1					Jtest		1							8			
BTzPhOMe5	416	Done	76,6	-5,00407	glide-grid_2YXJtest	-6,39507	-1,9751	-0,16	0	-0,4872	-57,8042	-6,79726	0,223613	-0,08658	-87,9013	-64,6014	7,88014
BTzPh1	198	Done	48,47	-5,00353	glide-grid_2YXJtest	-7,35033	-2,66763	-0,18416	0	-0,5537	-55,3051	-7,78182	0,184509	-0,19681	-87,1641	-63,0869	11,9362
BTz4	82	Done	106,2	-5,00198	glide-grid_2YXJtest	-7,53538	-2,64313	-0,32	0	-0,92664	-54,0494	-8,23576	0,326906	-0,03468	-83,1147	-62,2852	12,573
BTzPh9	257	Done	64,7	-5,00065	glide-grid_2YXJtest	-6,44885	-1,61086	-0,304	0	-0,11365	-59,9017	-9,78137	0,332533	-0,29058	-94,9236	-69,683	6,13369
BTzPhOMe9	449	Done	80,34	-4,99604	glide-grid_2YXJtest	-5,64414	-1,20759	-0,43376	0	-0,11327	-40,0144	-13,6482	0,28187	-0,12346	-71,3396	-53,6626	14,2335
BTz8	109	Done	76,68	-4,98353	glide-grid_2YXJtest	-5,92683	-2,33738	-0,38428	0	-0,23803	-43,8606	-5,62483	0,406764	-0,33715	-67,9166	-49,4855	9,28881
BTzPh6	237	Done	66,23	-4,97233	glide-grid_2YXJtest	-7,64493	-3,60062	-0,15575	0	-0,67524	-53,5655	-4,59898	0,294136	-0,13933	-86,3127	-58,1645	6,74526
BTzPhOMe6	422	Done	75,03	-4,96552	glide-grid_2YXJtest	-5,61362	-2,0862	-0,16	0	-0,11898	-50,173	-5,97377	0,242032	-0,08576	-73,7356	-56,1468	12,7204
BTz6	95	Done	90,18	-4,93977	glide-grid_2YXJtest	-6,53917	-2,20408	-0,53312	0	-0,41826	-49,6626	-8,15868	0,375193	-0,05197	-81,3634	-57,8213	10,2119
BTzPhOMe9	456	Done	71	-4,92588	glide-grid_2YXJtest	-6,92778	-2,92834	-0,00138	0	-0,425	-69,6446	-1,61008	0,280258	-0,12958	-89,2082	-71,2547	23,4541
BTzPhOMe6	424	Done	69,1	-4,92511	glide-grid_2YXJtest	-5,93921	-1,62515	-0,37938	0	-0,23794	-48,7674	-9,06883	0,241209	-0,13925	-77,4998	-57,8362	11,8668
BTzPhOMe7	439	Done	59,19	-4,92289	glide-grid_2YXJtest	-7,54659	-2,60053	-0,56889	0	-0,04727	-55,0853	-10,7652	0,255286	-0,21615	-94,301	-65,8505	5,53515
BTzPhDMN9	377	Done	64,92	-4,91651	glide-grid_2YXJtest	-7,49671	-2,91214	-0,37759	0	-0,65514	-62,6603	-4,30511	0,260195	-0,03325	-94,7007	-66,9654	7,03095

BTzPh5	227	Done	66,89	-4,91402	glide-grid_2YXJtest	-7,35332	-3,1363	-0,16568	0	-0,5755	-61,2799	-4,2223	0,278558	-0,05706	-95,3771	-65,5022	6,00734
BTz3	72	Done	83,73	-4,91363	glide-grid_2YXJtest	-7,30113	-2,47799	-0,73467	0	0	-42,2579	-13,4861	0,298207	-0,25088	-78,0538	-55,7439	7,69206
BTzNaf6	165	Done	55,84	-4,90525	glide-grid_2YXJtest	-7,28575	-3,10455	-0,32	0	-0,44121	-53,3023	-6,43525	0,210443	-2,28E-05	-87,4888	-59,7375	4,98602
BTzPhOMe6	428	Done	71,32	-4,89967	glide-grid_2YXJtest	-6,90157	-2,20405	-0,46086	0	-0,38534	-46,5799	-11,0877	0,240388	-0,09956	-84,3634	-57,6676	3,25451
BTzPh9	261	Done	65,91	-4,89198	glide-grid_2YXJtest	-7,56458	-2,89237	-0,4641	0	-0,89953	-57,4401	-5,04668	0,332533	-0,01211	-88,8403	-62,4868	10,528
BTzPhOMe3	401	Done	67,48	-4,88537	glide-grid_2YXJtest	-6,85307	-2,53162	-0,32	0	-0,75221	-58,8098	-3,15374	0,183449	-0,01913	-83,8716	-61,9636	6,035
BTzPhDMN9	376	Done	71,58	-4,88315	glide-grid_2YXJtest	-7,39225	-3,17716	-0,34457	0	-0,47094	-63,1553	-2,85221	0,260944	-0,07493	-93,8475	-66,0075	14,4086
BTz6	99	Done	92,95	-4,88148	glide-grid_2YXJtest	-7,42548	-2,14897	-0,65416	0	-0,86296	-52,9562	-9,46063	0,372355	-0,06485	-86,1516	-62,4168	8,15702
BTz4	77	Done	102,68	-4,87332	glide-grid_2YXJtest	-5,81192	-1,77891	-0,16827	0	-1,36532	-50,2444	-2,10364	0,328349	0	-71,2028	-52,348	5,33551
BTzPhOMe4	406	Done	69,35	-4,84406	glide-grid_2YXJtest	-5,85966	-2,40134	-0,16	0	-0,18785	-45,793	-5,43931	0,205543	-0,21047	-70,1838	-51,2323	11,5355
BTzPhOMe5	418	Done	70,12	-4,84169	glide-grid_2YXJtest	-6,48209	-2,68914	0	0	-0,45156	-61,4395	-3,19545	0,224435	-0,01453	-89,6826	-64,635	7,81456
BTz8	112	Done	67,84	-4,84119	glide-grid_2YXJtest	-7,19599	-2,56085	-0,33653	0	-0,18218	-51,4937	-11,6371	0,405393	-0,20157	-88,4305	-63,1308	11,3365
BTzPh5	229	Done	81,63	-4,82843	glide-grid_2YXJtest	-7,42373	-3,26092	-0,03282	0	-0,44017	-53,5271	-7,51147	0,277558	-0,16431	-87,8003	-61,0385	7,7016
BTzPh1	199	Done	52,85	-4,81719	glide-grid_2YX	-7,41439	-3,3823	-0,16	0	-0,6511	-53,4628	-4,01868	0,18549	-0,1304	-82,5512	-57,4814	6,94015

					Jtest		9							4			
BTz9	123	Done	81,94	-4,81101	glide-grid_2YXJtest	-7,35601	-2,62772	-0,42357	0	-0,28333	-47,4482	-12,4378	0,41828	-0,20159	-85,6074	-59,886	9,14255
BTzPhDMN4	342	Done	53,73	-4,80279	glide-grid_2YXJtest	-7,46899	-2,08042	-0,32	0	-0,39559	-52,0184	-13,523	0,184456	-0,22807	-93,383	-65,5414	7,65316
BTzPhCl1	266	Done	47,84	-4,8013	glide-grid_2YXJtest	-7,2874	-3,68318	0	0	-0,42955	-55,5379	-2,31474	0,126457	-0,17701	-84,553	-57,8526	5,33731
BTz6	98	Done	101,86	-4,79735	glide-grid_2YXJtest	-7,34135	-1,93053	-0,32	0	-1,6949	-59,4569	-5,30291	0,372355	0	-90,5627	-64,7598	6,90445
BTzPh1	197	Done	50,57	-4,77086	glide-grid_2YXJtest	-7,07556	-3,02717	-0,37982	0	-0,36603	-54,7375	-4,19337	0,18549	-0,12216	-79,2111	-58,9308	10,5474
BTzPhCl9	308	Done	56,37	-4,76848	glide-grid_2YXJtest	-5,66808	-1,4664	-0,456	0	-0,28469	-51,3998	-6,13388	0,274081	-0,245	-78,7462	-57,5337	5,98621
BTzPh6	231	Done	65,86	-4,76384	glide-grid_2YXJtest	-5,54394	-2,04226	-0,16	0	-0,03263	-50,8612	-5,45446	0,295128	-0,24295	-71,7427	-56,3157	14,8244
BTzPhOMe8	446	Done	65,74	-4,74508	glide-grid_2YXJtest	-6,74698	-2,27415	-0,16	0	-0,25314	-68,7172	-5,12922	0,268515	-0,12297	-92,2434	-73,8464	22,6835
BTzPhCl6	291	Done	51,75	-4,72985	glide-grid_2YXJtest	-5,87555	-2,22007	-0,42102	0	-0,43692	-54,0508	-2,13992	0,234095	-0,00812	-78,9749	-56,1907	6,00089
BTzNaf1	131	Done	46,42	-4,72964	glide-grid_2YXJtest	-7,35674	-2,91459	-0,16	0	-0,58234	-59,5727	-4,85182	0,103626	-0,09703	-93,3259	-64,4245	2,18276
BTzNaf8	181	Done	52,59	-4,71789	glide-grid_2YXJtest	-7,09839	-2,366	-0,51265	0	-0,20966	-63,3047	-6,20011	0,238783	-0,15361	-97,7266	-69,5048	10,8848
BTzNaf8	182	Done	74,68	-4,71274	glide-grid_2YXJtest	-7,16344	-2,9306	-0,15367	0	-0,0408	-65,4205	-6,42059	0,238783	-0,04305	-99,6333	-71,8411	11,9389
BTzPhDMN3	332	Done	48,57	-4,71271	glide-grid_2YXJtest	-7,19351	-2,53418	-0,20302	0	-0,34631	-59,9298	-8,16641	0,163391	-0,05194	-92,5585	-68,0962	8,26184

BTz3	73	Done	83,49	-4,70605	glide-grid_2YXJtest	-7,09495	-2,5553	-0,49973	0	-0,33662	-48,7218	-10,1634	0,298207	-0,04091	-80,4567	-58,8852	7,69383
BTzPhOMe8	440	Done	66,1	-4,69574	glide-grid_2YXJtest	-5,34384	-1,53553	-0,21131	0	-0,10111	-47,7557	-8,6161	0,270143	-0,08584	-72,1132	-56,3718	12,3261
BTz4	81	Done	98,49	-4,683	glide-grid_2YXJtest	-7,0559	-2,47094	-0,16	0	-0,66496	-45,715	-10,2238	0,326906	-0,26759	-79,2112	-55,9388	6,60749
BTzNaf6	168	Done	54,79	-4,67597	glide-grid_2YXJtest	-7,34857	-1,9384	-0,28438	0	-0,89876	-59,7359	-8,2283	0,209716	-0,21571	-94,2533	-67,9642	10,6312
BTzPhOMe9	451	Done	83,22	-4,659	glide-grid_2YXJtest	-5,6731	-1,69921	-0,16	0	-0,17854	-57,2244	-6,23439	0,281063	-0,12005	-82,8144	-63,4587	11,0044
BTzPhOMe4	410	Done	60,01	-4,65287	glide-grid_2YXJtest	-6,64687	-2,5958	-0,47114	0	-0,45389	-55,0993	-3,71193	0,204725	-0,01902	-79,4083	-58,8112	8,64065
BTz8	111	Done	70,64	-4,65003	glide-grid_2YXJtest	-6,25043	-2,51915	-0,43011	0	-0,17002	-42,0514	-7,89025	0,408139	-0,25318	-73,138	-49,9416	6,08415
BTzPhCl4	278	Done	68,51	-4,64644	glide-grid_2YXJtest	-5,54574	-1,52313	-0,52842	0	-0,04482	-43,2206	-8,71499	0,198458	-0,17954	-72,3263	-51,9356	6,78054
BTzNaf4	150	Done	48,19	-4,63824	glide-grid_2YXJtest	-7,01554	-2,5927	-0,29377	0	-0,18959	-52,4918	-7,97162	0,174997	-0,29414	-87,0003	-60,4634	8,65121
BTzPhCl5	288	Done	66,53	-4,63663	glide-grid_2YXJtest	-7,19043	-3,23911	0	0	-0,78581	-65,2829	-0,59524	0,21732	-0,02939	-92,9542	-65,8782	8,56731
BTzPhDMN1	319	Done	32,46	-4,63093	glide-grid_2YXJtest	-7,22813	-3,32128	-0,16	0	-0,54708	-56,0793	-2,68871	0,113909	-0,10641	-85,082	-58,768	6,0711
BTzPhCl8	303	Done	63,45	-4,62956	glide-grid_2YXJtest	-5,77526	-2,23632	-0,16	0	0	-52,417	-4,77524	0,262284	-0,30409	-80,3129	-57,1922	8,63472
BTzPh1	200	Done	51,42	-4,61961	glide-grid_2YXJtest	-7,24671	-3,63903	0	0	-0,88686	-56,1907	-0,6386	0,184509	0	-75,9601	-56,8293	9,70786
BTzPhDMN	366	Done	63,74	-4,6039	glide-grid_2YX	-5,6503	-1,5665	-0,22099	0	-0,15213	-56,5782	-7,2653	0,249046	-0,0409	-84,4772	-63,8435	7,40097

8					Jtest		8							4			
BTzNaf7	174	Done	53,5	-4,573	glide-grid_2YXJtest	-7,0237	-2,64434	-0,50714	0	-0,15524	-47,1684	-9,02377	0,225404	-0,23041	-80,9744	-56,1922	6,53957
BTzPhOMe7	436	Done	60,11	-4,56592	glide-grid_2YXJtest	-6,20182	-2,52541	-0,33116	0	-0,39658	-49,8035	-4,62407	0,256104	-0,02099	-74,8927	-54,4276	6,82881
BTzPhOMe9	454	Done	79,5	-4,54482	glide-grid_2YXJtest	-6,18072	-2,39358	-0,34936	0	-0,17898	-52,2218	-5,09761	0,281063	-0,16414	-78,7724	-57,3194	8,1587
BTzPh6	232	Done	60,24	-4,53701	glide-grid_2YXJtest	-5,52781	-2,01913	-0,15642	0	-0,02549	-52,3302	-5,05274	0,295128	-0,24749	-76,4356	-57,3829	11,7496
BTzPhDMN2	325	Done	41,07	-4,5325	glide-grid_2YXJtest	-6,9974	-2,54351	-0,26837	0	-0,48742	-57,8865	-6,09495	0,14048	-0,03001	-86,5553	-63,9815	12,4501
BTzPhOMe6	423	Done	71,91	-4,52261	glide-grid_2YXJtest	-5,53671	-1,94842	-0,16	0	-0,07044	-55,0858	-4,47071	0,241209	-0,17417	-79,6159	-59,5565	8,53221
BTzPh7	238	Done	67,19	-4,50808	glide-grid_2YXJtest	-5,06878	-2,28407	0	0	-0,34816	-54,2732	0,698223	0,310638	-0,13826	-75,1751	-53,575	1,99105
BTzPhCl3	276	Done	74,16	-4,50694	glide-grid_2YXJtest	-7,06094	-2,76082	-0,43608	0	-0,24943	-57,1574	-6,1056	0,17724	-0,01814	-90,338	-63,263	7,61349
BTzNaf6	166	Done	57,23	-4,50549	glide-grid_2YXJtest	-6,95619	-2,6854	-0,34006	0	-0,05321	-57,1289	-6,65399	0,210443	-0,23343	-91,2679	-63,7829	7,81049
BTz8	114	Done	76,41	-4,50171	glide-grid_2YXJtest	-7,04671	-2,37665	0	0	-1,27665	-61,1699	-4,61285	0,405393	-0,04838	-93,1722	-65,7828	6,21641
BTzPh1	196	Done	53,73	-4,49914	glide-grid_2YXJtest	-5,75594	-1,97441	-0,26156	0	-0,1835	-50,8184	-5,60668	0,184509	-0,13906	-74,3508	-56,4251	6,12165
BTz3	75	Done	65,35	-4,49518	glide-grid_2YXJtest	-7,01608	-2,33931	-0,5207	0	-0,88139	-45,9367	-7,47735	0,298207	-0,15445	-76,7817	-53,414	6,3644
BTzPhDMN7	362	Done	70,56	-4,49423	glide-grid_2YXJtest	-7,00333	-2,35289	-0,31169	0	-0,04871	-51,7165	-10,6537	0,235707	-0,34186	-92,2861	-62,3703	5,92577

BTzPh1	195	Done	49,27	-4,47337	glide-grid_2YXJtest	-5,44747	-2,21877	-0,16	0	-0,01857	-40,7221	-6,21811	0,18549	-0,2668	-61,3972	-46,9402	6,07623
BTzPhDMN4	337	Done	41,32	-4,43984	glide-grid_2YXJtest	-5,42334	-1,74822	-0,03987	0	-0,47916	-47,3935	-5,48034	0,185208	-0,14958	-68,6657	-52,8739	6,46906
BTz9	119	Done	64,79	-4,43927	glide-grid_2YXJtest	-6,03967	-2,53606	-0,29322	0	-0,24749	-45,8682	-5,42598	0,420972	-0,27656	-72,3357	-51,2942	9,55858
BTzPhDMN4	341	Done	47,76	-4,4348	glide-grid_2YXJtest	-6,9582	-2,72527	-0,36627	0	-0,06879	-50,7419	-8,38658	0,184456	-0,18725	-77,8538	-59,1285	13,6411
BTzPhOMe9	450	Done	89,82	-4,40879	glide-grid_2YXJtest	-5,42289	-0,97694	-0,7441	0	-0,00991	-47,4292	-9,83526	0,281063	-0,12624	-72,1173	-57,2645	16,611
BTzPhDMN7	361	Done	68,23	-4,39527	glide-grid_2YXJtest	-6,82967	-2,22014	-0,17057	0	-0,2579	-58,6705	-8,02173	0,235707	-0,27999	-96,1113	-66,6922	7,23164
BTzPhOMe3	403	Done	59,35	-4,39051	glide-grid_2YXJtest	-6,99301	-3,14357	-0,01622	0	-0,77728	-61,3076	-1,03559	0,183449	-0,01867	-86,4075	-62,3432	6,99356
BTzPhOMe5	417	Done	56,63	-4,38365	glide-grid_2YXJtest	-6,02405	-1,85897	-0,50097	0	-0,03236	-42,0752	-9,24938	0,224435	-0,36501	-72,8712	-51,3246	7,53698
BA	1	Done	2,36	-4,36826	glide-grid_2YXJtest	-4,37596	-1,37233	-0,38482	0	-0,3021	-26,6732	-6,70262	0,096752	-0,0744	-44,9269	-33,3759	1,21008
BTzPh2	205	Done	60,93	-4,34499	glide-grid_2YXJtest	-6,80989	-2,70768	-0,16	0	-0,41194	-58,1656	-5,47319	0,213536	-0,01455	-86,5263	-63,6387	10,6279
BTzPhOMe7	434	Done	68,51	-4,3379	glide-grid_2YXJtest	-5,7181	-1,73238	-0,02828	0	-0,30673	-64,6461	-4,17145	0,255286	-0,04797	-86,8277	-68,8176	8,59169
BTzPhOMe5	421	Done	62,47	-4,33757	glide-grid_2YXJtest	-6,97957	-3,05249	-0,14455	0	-0,23096	-49,5445	-7,64405	0,223613	-0,15135	-76,8501	-57,1886	12,5075
BTzNaf1	127	Done	38,27	-4,3327	glide-grid_2YXJtest	-5,5895	-1,49364	-0,28513	0	-0,00087	-48,2698	-8,69547	0,103626	-0,19567	-78,5965	-56,9653	2,12793
BTzPh3	213	Done	83,83	-4,31091	glide-grid_2YX	-6,79871	-2,4843	-0,3442	0	-0,20448	-55,5066	-8,08135	0,238112	-0,0163	-88,8558	-63,5879	6,2385

					Jtest		2										
BTzPh8	246	Done	54,53	-4,29357	glide-grid_2YXJtest	-4,85427	-1,27555	-0,60859	0	0	-42,8565	-6,17685	0,323344	-0,22412	-64,6777	-49,0334	8,09601
BTzPhCl4	281	Done	68,7	-4,29054	glide-grid_2YXJtest	-6,83244	-3,19916	-0,12824	0	-0,75277	-62,2322	1,10047	0,198458	-0,00419	-87,0693	-61,1318	8,43776
BTz2	65	Done	62,18	-4,28036	glide-grid_2YXJtest	-5,53826	-2,51192	-0,1126	0	-0,53361	-44,4019	-2,5746	0,267495	-0,04134	-65,1742	-46,9765	7,64752
BTzPhDMN2	326	Done	48,89	-4,26605	glide-grid_2YXJtest	-6,73675	-2,82074	-0,16966	0	-0,38948	-49,1973	-5,79236	0,14048	-0,16863	-76,5537	-54,9897	10,2813
BTzPhDMN2	321	Done	40,62	-4,26567	glide-grid_2YXJtest	-4,75337	-2,44235	0	0	-0,17967	-36,2968	-1,63524	0,141211	-0,21245	-50,8819	-37,9321	4,87197
BTzPh4	219	Done	58,17	-4,24909	glide-grid_2YXJtest	-6,62639	-2,49992	-0,58907	0	-0,09392	-50,4742	-6,01101	0,25966	-0,27777	-79,7721	-56,4852	8,0725
BTzPhOMe1	382	Done	39,89	-4,23696	glide-grid_2YXJtest	-5,78006	-2,13795	-0,32	0	-0,06726	-51,3174	-4,68327	0,133203	-0,1197	-73,1491	-56,0007	8,87972
BTzPhCl3	275	Done	77,99	-4,22431	glide-grid_2YXJtest	-6,75221	-2,71038	-0,30247	0	-0,35398	-57,8743	-4,44529	0,17724	-0,00211	-87,675	-62,3196	7,07438
BTzPh9	258	Done	73,9	-4,22133	glide-grid_2YXJtest	-6,60183	-2,33208	-0,43554	0	-0,27789	-52,3883	-6,89897	0,333491	-0,23556	-82,0555	-59,2873	10,9058
BTzPhOMe7	438	Done	66,46	-4,20901	glide-grid_2YXJtest	-6,21091	-2,21731	-0,21276	0	-0,29227	-52,3571	-6,59117	0,255286	-0,13733	-81,0376	-58,9483	4,10567
BTzNaf4	153	Done	64,78	-4,20368	glide-grid_2YXJtest	-6,86988	-2,63853	-0,456	0	-0,00182	-54,4335	-7,16571	0,174279	-0,15128	-84,4195	-61,5992	8,23444
BTzPhOMe8	447	Done	60,37	-4,18501	glide-grid_2YXJtest	-6,18691	-2,26103	-0,29272	0	-0,01968	-38,9596	-11,0761	0,268515	-0,27259	-68,1612	-50,0358	6,34325
BTz4	79	Done	112,41	-4,18328	glide-grid_2YXJtest	-5,79028	-2,4786	0	0	-0,42803	-46,6479	-5,84955	0,329799	-0,00362	-66,1239	-52,4974	20,6884

BTzPhDMN6	354	Done	60,8	-4,17753	glide-grid_2YXJtest	-6,61193	-2,17092	-0,32	0	-0,18417	-52,7177	-8,87002	0,220756	-0,19121	-80,8475	-61,5877	15,343
BTzPh3	214	Done	70,73	-4,16129	glide-grid_2YXJtest	-6,84049	-2,42316	-0,4645	0	-0,35075	-57,1645	-6,45768	0,237111	-0,01231	-87,7631	-63,6221	4,96355
BTzPhOMe9	457	Done	79,24	-4,15123	glide-grid_2YXJtest	-6,77493	-2,0506	-0,59399	0	-0,40122	-49,1608	-9,06978	0,280258	-0,19088	-76,0454	-58,2306	15,3466
BTzPhOMe9	455	Done	86,64	-4,1497	glide-grid_2YXJtest	-6,1516	-2,07337	-0,16	0	-0,86816	-61,3805	-1,74204	0,280258	0	-84,923	-63,1225	8,38333
BTzPh7	242	Done	67,59	-4,14727	glide-grid_2YXJtest	-6,52777	-2,32217	-0,42154	0	-0,10166	-50,9653	-7,62654	0,309652	-0,2998	-83,4125	-58,5919	6,21545
BTzPhOMe4	412	Done	67,87	-4,13494	glide-grid_2YXJtest	-6,75774	-2,48646	-0,16	0	-0,61804	-51,7876	-6,88091	0,204725	-0,07645	-79,723	-58,6685	10,2719
BTzNaf9	185	Done	79,17	-4,11328	glide-grid_2YXJtest	-4,67398	-1,34626	-0,29324	0	-0,14513	-51,0396	-2,60073	0,251469	-0,19873	-72,0182	-53,6403	3,82688
BTzPh9	259	Done	68,14	-4,10775	glide-grid_2YXJtest	-6,55845	-1,84379	-0,37356	0	-0,36545	-44,5924	-12,2425	0,333491	-0,24314	-78,3227	-56,8349	13,8184
BTz1	57	Done	49,04	-4,08857	glide-grid_2YXJtest	-5,79837	-2,4557	0	0	-0,73737	-43,9419	-4,00569	0,228099	-0,03546	-64,4261	-47,9476	8,5079
BTzPh6	234	Done	66,91	-4,075	glide-grid_2YXJtest	-6,4555	-2,14392	-0,68978	0	-0,04351	-43,6808	-9,06237	0,295128	-0,33002	-75,288	-52,7432	9,49256
BTzPhOMe7	431	Done	68,82	-4,06966	glide-grid_2YXJtest	-4,71776	-1,70095	-0,08827	0	-0,09952	-53,6615	-1,78485	0,256924	-0,13514	-71,5081	-55,4463	12,1579
BTzPhDMN7	363	Done	62,42	-4,04091	glide-grid_2YXJtest	-6,62111	-2,4603	-0,32	0	-0,58828	-65,998	-0,77859	0,23495	-0,07079	-90,7219	-66,7766	4,7147
BTzPhDMN3	334	Done	59,21	-4,03424	glide-grid_2YXJtest	-6,71344	-2,17181	-0,26176	0	-0,06071	-52,3649	-10,3447	0,163391	-0,2126	-87,0458	-62,7096	4,60091
BTzNaf5	158	Done	55,8	-4,02721	glide-grid_2YX	-6,46651	-2,3418	-0,443	0	0	-50,0251	-7,64953	0,193712	-0,2267	-80,4556	-57,6746	7,26029

					Jtest		3							1			
BTzPhCl6	294	Done	65,59	-3,97176	glide-grid_2YXJtest	-6,51806	-1,52968	-0,608	0	-0,29025	-52,0802	-9,18488	0,234095	-0,34248	-85,4599	-61,2651	9,66164
BTz4	80	Done	105,32	-3,9389	glide-grid_2YXJtest	-6,3077	-2,61931	0	0	-0,8858	-53,1443	-3,14461	0,326906	-0,00059	-74,7733	-56,2889	6,20038
BTzPhOMe6	430	Done	61,17	-3,91476	glide-grid_2YXJtest	-6,53846	-1,78987	-0,50833	0	-0,16062	-55,7668	-9,12354	0,240388	-0,16315	-86,0686	-64,8904	13,7782
BTz2	66	Done	57,01	-3,90714	glide-grid_2YXJtest	-6,41494	-1,68027	-0,50787	0	-0,89937	-48,3458	-7,44479	0,264611	-0,05804	-73,0734	-55,7906	9,70954
BTz8	115	Done	65,79	-3,89697	glide-grid_2YXJtest	-6,44197	-2,40898	-0,304	0	-0,19354	-47,6699	-8,9001	0,405393	-0,22233	-78,2586	-56,57	8,66525
BTzPhCl5	287	Done	66,38	-3,8725	glide-grid_2YXJtest	-6,4263	-2,74309	0	0	-0,20589	-62,4137	-2,29045	0,21732	-0,23038	-86,1968	-64,7042	16,3954
BTzPhDMN5	347	Done	60,26	-3,82107	glide-grid_2YXJtest	-6,26037	-2,25503	-0,27003	0	-0,26567	-48,4494	-6,34393	0,203997	-0,29958	-80,286	-54,7933	4,65008
BTzPhDMN5	348	Done	53,39	-3,81789	glide-grid_2YXJtest	-6,33209	-2,03059	-0,16	0	-0,35197	-50,0023	-7,93698	0,203997	-0,30287	-83,0689	-57,9392	4,02368
BTz5	90	Done	89,72	-3,81463	glide-grid_2YXJtest	-6,36563	-1,9561	-0,39681	0	-0,49307	-50,0373	-7,70728	0,351423	-0,21313	-78,1741	-57,7446	5,90461
BTzPhOMe1	381	Done	41,91	-3,81054	glide-grid_2YXJtest	-5,06434	-1,30512	-0,36878	0	0	-44,3796	-7,41867	0,132423	-0,19109	-66,5959	-51,7982	5,77671
BTzPh8	251	Done	59,97	-3,80692	glide-grid_2YXJtest	-6,25762	-2,1264	-0,32	0	-0,83591	-57,8048	-2,47383	0,322369	-0,03638	-85,1186	-60,2787	3,55164
BTzPhDMN1	318	Done	34,51	-3,80024	glide-grid_2YXJtest	-6,14704	-2,52964	-0,2272	0	-0,56895	-46,8828	-3,72614	0,1132	-0,0314	-69,0338	-50,6089	6,44086
BTzPhOMe5	419	Done	67,02	-3,79033	glide-grid_2YXJtest	-5,80683	-1,17246	-0,70944	0	-0,0175	-47,4661	-10,4119	0,223613	-0,19595	-76,9502	-57,878	7,30622

BTzPhDMN6	355	Done	67,09	-3,78966	glide-grid_2YXJtest	-6,29876	-1,68988	-0,32	0	-0,01799	-46,7083	-12,1493	0,220756	-0,33384	-82,5097	-58,8575	8,03236
BTz2	67	Done	65,65	-3,75255	glide-grid_2YXJtest	-6,28655	-2,43527	-0,32	0	-1,15227	-46,5128	-2,04039	0,264611	-0,01193	-59,097	-48,5532	19,7755
BTz7	104	Done	75,58	-3,74771	glide-grid_2YXJtest	-6,10261	-1,56747	-0,456	0	-0,29204	-50,343	-9,19085	0,390204	-0,28153	-79,857	-59,5338	8,03539
BTzPh5	228	Done	89,84	-3,73401	glide-grid_2YXJtest	-6,24821	-2,47418	-0,56212	0	-0,04193	-48,9554	-5,25666	0,278558	-0,21227	-75,7014	-54,212	8,58504
BTzPhOMe9	453	Done	70,05	-3,66803	glide-grid_2YXJtest	-5,30393	-1,65857	-0,16	0	-0,09069	-57,5484	-3,5128	0,281063	-0,27139	-78,9554	-61,0612	11,9959
BTz3	74	Done	82,83	-3,66696	glide-grid_2YXJtest	-6,18066	-1,79245	-0,36	0	-1,19421	-50,9471	-3,5275	0,298207	-0,05572	-73,9763	-54,4746	4,27812
BTzPhCl4	282	Done	61,42	-3,65168	glide-grid_2YXJtest	-6,20428	-2,09649	-0,32	0	-0,32361	-48,1987	-6,94763	0,198458	-0,21056	-76,7491	-55,1463	7,52786
BTzPh6	236	Done	70,03	-3,63521	glide-grid_2YXJtest	-6,16731	-1,89665	-0,20336	0	-0,12081	-51,819	-9,83967	0,294136	-0,17372	-82,1533	-61,6587	10,6432
BTzPh6	235	Done	69,71	-3,60996	glide-grid_2YXJtest	-6,06066	-2,31227	-0,28646	0	-0,10865	-48,6696	-6,03028	0,295128	-0,3104	-72,9102	-54,6998	13,0865
BTzNaf6	167	Done	65,99	-3,54168	glide-grid_2YXJtest	-6,07378	-2,19209	-0,23497	0	-0,28588	-56,8819	-4,52693	0,209716	-0,04743	-83,1011	-61,4088	6,46802
BTzPh4	222	Done	57,23	-3,53521	glide-grid_2YXJtest	-6,20141	-1,74542	-0,3812	0	-0,10675	-46,4159	-11,2852	0,258658	-0,21312	-77,9673	-57,7011	6,92338
BTzNaf9	192	Done	63,14	-3,53196	glide-grid_2YXJtest	-6,20456	-1,64976	-0,40366	0	-0,06749	-57,4973	-8,47621	0,250027	-0,18739	-93,3571	-65,9735	4,52823
BTzPhDMN4	340	Done	55,34	-3,43236	glide-grid_2YXJtest	-5,89076	-1,57061	-0,70141	0	-0,04739	-36,9367	-10,7626	0,185208	-0,29534	-67,6162	-47,6993	6,65471
BTzPhDMN	349	Done	59,41	-3,43139	glide-grid_2YX	-6,02669	-1,8510	-0,6337	0	-0,11824	-51,6375	-6,11044	0,203241	-0,1285	-79,1104	-57,7479	3,80265

5					Jtest		3							2			
BTz1	58	Done	49,98	-3,42784	glide-<br>grid_2YX<br>Jtest	-<br>5,78444	-<br>1,3354<br>5	-0,80672	0	-0,10465	-<br>34,4359	-<br>12,5041	0,22525<br>8	-<br>0,1654<br>6	-62,599	-46,94	3,1039
BTzPh<br>OMe8	448	Done	68,17	-3,41991	glide-<br>grid_2YX<br>Jtest	-<br>6,04361	-<br>1,3072<br>2	-0,56048	0	-0,26243	-<br>57,8081	-<br>8,07564	0,26851<br>5	-<br>0,0802<br>6	-83,8332	-65,8837	14,1484
BTzPh<br>Cl9	311	Done	64,63	-3,41754	glide-<br>grid_2YX<br>Jtest	-<br>5,96384	-<br>2,9454<br>1	-0,34411	0	-0,22792	-<br>54,3213	0,04314<br>7	0,27408<br>1	-<br>0,0108<br>8	-77,4789	-54,2782	2,85827
BTzPh<br>DMN<br>8	368	Done	69,07	-3,38535	glide-<br>grid_2YX<br>Jtest	-<br>5,81975	-<br>1,5934<br>2	-0,6963	0	-0,27623	-<br>50,3526	-<br>6,01908	0,24904<br>6	-<br>0,0823<br>6	-76,7458	-56,3717	4,80652
BTzPh<br>7	243	Done	58,82	-3,28385	glide-<br>grid_2YX<br>Jtest	-<br>5,73455	-<br>1,9405<br>6	-0,16	0	-0,25982	-<br>59,7948	-<br>4,17875	0,30965<br>2	-<br>0,0672<br>6	-84,3341	-63,9736	9,31088
BTzPh<br>Cl8	306	Done	53,54	-3,20659	glide-<br>grid_2YX<br>Jtest	-<br>5,75289	-<br>0,9754<br>8	-0,39957	0	-0,07361	-<br>49,3968	-<br>12,3244	0,26228<br>4	-<br>0,2480<br>2	-80,7762	-61,7212	12,2017
BTzPh<br>Cl7	299	Done	59,11	-3,18312	glide-<br>grid_2YX<br>Jtest	-<br>5,72942	-<br>1,7020<br>6	-0,32	0	0	-<br>54,7625	-<br>6,75356	0,24901<br>5	-<br>0,2052<br>1	-83,1628	-61,516	4,72132
BTz1	59	Done	43,92	-3,04144	glide-<br>grid_2YX<br>Jtest	-<br>5,51434	-<br>2,0976<br>7	-0,17406	0	-0,58899	-<br>39,0049	-<br>5,36046	0,22525<br>8	-<br>0,1245<br>6	-59,2766	-44,3653	4,10005
BTzPh<br>Cl7	300	Done	58,78	-3,03105	glide-<br>grid_2YX<br>Jtest	-<br>5,57735	-<br>2,2059<br>6	0	0	-0,32293	-<br>57,9685	-<br>1,68638	0,24901<br>5	-<br>0,1460<br>9	-79,5767	-59,6548	6,48072
BTzPh<br>Cl9	312	Done	62,13	-2,95358	glide-<br>grid_2YX<br>Jtest	-<br>5,49988	-<br>1,7593<br>1	-0,16	0	0	-<br>54,9551	-<br>4,59343	0,27408<br>1	-<br>0,4178<br>8	-79,6378	-59,5486	8,42747
BTz1	60	Done	51,65	-2,50028	glide-<br>grid_2YX<br>Jtest	-<br>5,08608	-<br>2,0314<br>2	-0,11236	0	-0,30778	-<br>40,3806	-4,6614	0,22525<br>8	-<br>0,1415<br>4	-57,3357	-45,042	5,35427
BTz1	61	Done	48,27	-2,32915	glide-<br>grid_2YX<br>Jtest	-<br>4,96415	-<br>1,5884<br>2	-0,17804	0	-0,04787	-<br>34,4806	-9,7689	0,22525<br>8	-<br>0,1857<br>1	-53,4725	-44,2495	6,62767
BA	2	Done	2,31	-0,47492	glide-<br>grid_2YX<br>Jtest	-<br>3,05491	-<br>1,1041<br>4	0	0	-0,46364	-<br>30,5265	-<br>1,02352	0,19272<br>7	0	-37,4048	-31,5501	1,63684

Table S6. Glide docking scores of docked ligands against Bcl-2 (4LVT)

title	i_i_glide_lignum	docking_status	r_glide_cpu_time	r_i_docking_score	s_i_glide_gridfile	r_i_glide_gscore	r_i_glide_lipo	r_i_glide_hbond	r_i_glide_metal	r_i_glide_rewards	r_i_glide_evdw	r_i_glide_ecoul	r_i_glide_erotb	r_i_glide_esite	r_i_glide_emodel	r_i_glide_energy	r_i_glide_einternal
4LVT - with-deletions_ligand	1	Done	141,59	-12,4952	glide-grid_4LVT	-12,6678	-5,29677	-0,0016	0	-1,64876	-72,2525	-14,1772	0,128115	-0,10956	-152,324	-86,4297	18,71
4LVT - with-deletions_ligand	2	Done	168,95	-10,4207	glide-grid_4LVT	-11,2979	-4,83315	0	0	-1,77302	-74,0657	-7,32743	0,12884	-0,01818	-132,465	-81,3931	10,9176
4LVT - with-deletions_ligand	3	Done	156,4	-8,86599	glide-grid_4LVT	-11,0475	-4,2965	0	0	-1,82465	-72,2743	-9,31569	0,12884	-0,04411	-131,43	-81,59	15,9264
BOxPhCl5	34	Done	98,09	-8,22408	glide-grid_4LVT	-8,22408	-4,29729	0	0	-0,38463	-67,4253	-2,45241	0,214968	-0,018	-100,283	-69,8777	7,07315
BOxPhDMN3	41	Done	61,24	-7,97028	glide-grid_4LVT	-7,97028	-3,44368	-0,27368	0	-0,72132	-62,6537	-3,73904	0,161947	0	-90,2352	-66,3927	18,5435
BOxPhCl3	32	Done	83,99	-7,9104	glide-grid_4LVT	-7,9104	-4,64327	0	0	-0,46537	-60,1203	0,195538	0,174922	0	-81,0015	-59,9248	13,5532
BOxPh6	26	Done	71,77	-7,89462	glide-grid_4LVT	-7,89462	-4,22995	-0,22907	0	-0,48721	-64,5875	-0,07486	0,292205	0	-90,1124	-64,6624	5,70392
BOx9	11	Done	89,7	-7,79131	glide-grid_4LVT	-7,79131	-3,39577	-0,59844	0	-0,74132	-59,1681	-3,42026	0,415668	0	-92,0525	-62,5883	6,61516
BTzPhCl5	296	Done	91,1	-7,76225	glide-grid_4LVT	-8,66595	-3,88823	-0,487	0	-0,38812	-57,1929	-7,06742	0,21732	-0,20017	-101,972	-64,2603	12,52
BTzPhCl8	315	Done	65,18	-7,63317	glide-grid_4LVT	-8,77887	-4,23298	-0,24488	0	-0,77696	-61,8493	-3,78153	0,262284	-0,12664	-106,115	-65,6309	6,41312
BTz5	96	Done	109,55	-7,60776	glide-grid_4LVT	-8,07366	-2,65258	-0,30817	0	-1,27893	-54,7463	-8,49053	0,351423	-0,17451	-90,1654	-63,2368	12,9402
BOxPh3	23	Done	88,55	-7,5787	glide-grid_4LVT	-7,5787	-3,87142	-0,16	0	-0,52173	-49,1655	-4,17349	0,235165	-0,17641	-78,9049	-53,339	4,64736
BTz7	114	Done	107,66	-7,56906	glide-grid_4L	-8,51236	-3,0780	-0,35589	0	-1,11023	-56,454	-8,697	0,391599	-0,2325	-97,6366	-65,1512	13,363

					VT		4				2			3			
BOxPh2	22	Done	89,95	-7,51446	glide-grid_4LVT	-7,51446	-3,78598	-0,08813	0	-0,25924	-63,0526	-2,55256	0,210609	-0,05621	-91,685	-65,6052	8,08061
BOxNaf3	14	Done	71,9	-7,32898	glide-grid_4LVT	-7,32898	-3,48245	-0,16	0	-0,30055	-53,5509	-4,7004	0,151965	-0,15534	-81,2346	-58,2513	9,01547
BOxPhCl7	36	Done	69,06	-7,31064	glide-grid_4LVT	-7,31064	-3,06451	-0,16	0	-0,26603	-65,9697	-4,64511	0,246669	-0,07151	-95,8746	-70,6148	13,1985
BT6	62	Done	74,09	-7,30574	glide-grid_4LVT	-7,30574	-2,88627	0	0	-1,10697	-65,7049	-1,87711	0,287971	-0,03366	-82,8326	-67,582	27,4836
BT5	61	Done	74,46	-7,23536	glide-grid_4LVT	-7,23536	-3,5302	0	0	-0,65248	-62,2073	-1,00296	0,266438	-0,05832	-89,0583	-63,2103	7,79729
BTzPhCl8	316	Done	61,65	-7,13308	glide-grid_4LVT	-8,53338	-4,05724	-0,32	0	-0,50444	-54,2941	-6,96255	0,261492	-0,1541	-89,8167	-61,2566	15,2019
BTz4	88	Done	113,94	-6,9664	glide-grid_4LVT	-7,4264	-2,49861	-0,16	0	-1,09449	-56,699	-6,59986	0,326906	-0,17528	-87,016	-63,2989	12,1715
BTz3	80	Done	67,72	-6,95592	glide-grid_4LVT	-7,41222	-2,02024	-0,68668	0	-0,7619	-49,6901	-10,7335	0,298207	-0,14709	-82,6102	-60,4236	14,151
BTzPh9	267	Done	70,39	-6,93773	glide-grid_4LVT	-7,71783	-3,01441	-0,52661	0	-0,50538	-53,8654	-7,48395	0,333491	-0,18906	-85,4673	-61,3493	16,7563
BOxPhOMe2	49	Done	87,2	-6,89584	glide-grid_4LVT	-6,89584	-3,32337	0	0	-0,11667	-60,9343	-3,63135	0,157917	-0,0223	-86,0515	-64,5657	14,2508
BTzPhOMe6	435	Done	92,49	-6,83396	glide-grid_4LVT	-7,84806	-3,50205	-0,35544	0	-0,23459	-57,0717	-6,51097	0,241209	-0,16695	-97,7781	-63,5827	11,301
BTzPhCl6	302	Done	67,89	-6,81451	glide-grid_4LVT	-7,71411	-3,14269	-0,37885	0	-0,4601	-52,1209	-7,81615	0,234095	-0,1881	-93,2326	-59,9371	9,17943
BT3	59	Done	69,17	-6,74279	glide-grid_4LVT	-6,74279	-3,50841	0	0	-0,55105	-57,0632	0,098814	0,213345	-0,05834	-77,5279	-56,9644	8,25022
BOxPhOMe3	50	Done	89,52	-6,70819	glide-grid_4LVT	-6,70819	-3,59223	0	0	-0,13119	-56,1427	-2,21572	0,181871	-0,02715	-77,6022	-58,3585	12,0741
BOxPhCl1	30	Done	74,78	-6,69877	glide-	-	-	-0,255	0	-0,49453	-	-	0,1242	-	-76,2967	-	7,98751

					grid_4L VT	6,69877	2,93695				55,2722	1,98206	34	0,07561		57,2542	
BTzPh8	261	Done	56,38	-6,64305	glide-grid_4L VT	-8,09125	-3,40907	-0,32	0	-0,82929	-62,8608	-4,60605	0,321398	-0,02034	-96,7134	-67,4668	8,41063
BTzPhCl3	286	Done	92,93	-6,61481	glide-grid_4L VT	-7,96591	-3,75396	-0,01485	0	-0,65921	-62,729	-3,75674	0,176453	-0,01438	-88,8964	-66,4858	17,4922
BOx5	7	Done	146,43	-6,59293	glide-grid_4L VT	-6,59293	-2,85248	0	0	-0,24497	-54,6126	-6,26051	0,348638	-0,17441	-85,2451	-60,8731	6,54916
BTzNaf9	203	Done	62,95	-6,56186	glide-grid_4L VT	-9,09396	-3,84952	-0,48151	0	-0,52	-56,7849	-9,87224	0,250027	-0,17288	-101,254	-66,6571	10,8785
BTzPh9	269	Done	59,77	-6,5364	glide-grid_4L VT	-7,9846	-3,27562	-0,28283	0	-1,25176	-56,9353	-4,28311	0,332533	-0,0177	-83,7262	-61,2184	15,0957
BOx4	6	Done	145,9	-6,47714	glide-grid_4L VT	-6,47714	-2,71509	0	0	-0,28958	-53,4611	-6,39676	0,324101	-0,16399	-78,9109	-59,8579	11,529
BTzPhCl2	281	Done	85,89	-6,45914	glide-grid_4L VT	-7,62244	-2,8763	-0,32	0	-0,80244	-52,5213	-7,05979	0,153359	-0,09202	-88,9661	-59,5811	4,08086
BTz8	120	Done	79,29	-6,39423	glide-grid_4L VT	-6,85293	-2,17862	-0,78184	0	-0,47675	-50,6218	-8,47467	0,405393	-0,01883	-77,9452	-59,0965	14,4769
BOxPhOMe6	53	Done	90,13	-6,36198	glide-grid_4L VT	-6,36198	-3,17945	0	0	-0,17865	-56,0519	-2,68612	0,23879	-0,03716	-79,0293	-58,738	9,61093
BTzNaf5	167	Done	66,78	-6,35251	glide-grid_4L VT	-7,17621	-3,5488	-0,13244	0	-0,27451	-53,7761	-4,18148	0,193712	-0,09814	-86,4655	-57,9576	3,67568
BTzNaf7	183	Done	57,97	-6,33341	glide-grid_4L VT	-7,32421	-2,60179	-0,31527	0	-0,21291	-58,4946	-9,16611	0,225404	-0,12	-96,2629	-67,6607	8,54937
BTzPh4	229	Done	96,52	-6,30273	glide-grid_4L VT	-7,28623	-3,27421	-0,49231	0	-0,38604	-48,0482	-5,27846	0,25966	-0,19916	-84,6606	-53,3267	5,12764
BTzPhCl8	313	Done	55,9	-6,29269	glide-grid_4L VT	-6,69159	-3,24638	-0,2949	0	-0,33512	-52,1614	-2,42751	0,263079	-0,10608	-77,4229	-54,589	11,9617
BTzNaf4	163	Done	57,37	-6,25993	glide-grid_4L VT	-8,71833	-4,81905	0	0	-0,64514	-63,1845	-1,62568	0,174997	-0,02607	-96,5322	-64,8102	10,2479

BOxPhOMe1	48	Done	56,8	-6,24462	glide-grid_4LVT	-6,24462	-2,7299	-0,41193	0	-0,19239	-55,4858	-1,38895	0,130906	-0,05866	-76,7454	-56,8748	5,5235
BOxPhDMN8	46	Done	70,84	-6,21079	glide-grid_4LVT	-6,21079	-1,88141	-0,56668	0	-0,06695	-56,6491	-6,09388	0,246826	-0,19604	-82,7941	-62,743	13,1613
BTz4	90	Done	123,69	-6,20023	glide-grid_4LVT	-7,14133	-2,00041	-0,52781	0	-0,08333	-34,2976	-18,5266	0,328349	-0,36426	-78,9885	-52,8242	7,30593
BTz9	131	Done	70,34	-6,19902	glide-grid_4LVT	-7,79942	-2,62441	-0,608	0	-0,26208	-48,1921	-13,7917	0,420972	-0,24754	-95,6993	-61,9838	15,8975
BTzPh8	262	Done	58,85	-6,18503	glide-grid_4LVT	-8,56553	-3,90127	-0,20634	0	-0,91458	-56,0149	-6,14771	0,322369	-0,14281	-99,3775	-62,1626	7,91115
BTzNaf9	200	Done	65,28	-6,18354	glide-grid_4LVT	-7,63174	-1,66638	-0,69044	0	-0,13044	-57,2581	-15,6299	0,250027	-0,1871	-102,433	-72,888	14,5925
BTzNaf4	159	Done	54,62	-6,18179	glide-grid_4LVT	-6,96279	-1,93822	-0,40252	0	-0,15421	-53,6816	-11,7028	0,174997	-0,20335	-92,9429	-65,3844	8,86191
BOxNaf4	15	Done	55,1	-6,14624	glide-grid_4LVT	-6,14624	-2,65886	-0,32	0	0	-53,1169	-2,88494	0,172882	-0,25167	-72,8482	-56,0018	8,66045
BOx3	5	Done	112,67	-6,13946	glide-grid_4LVT	-6,13946	-2,95514	0	0	-0,24789	-53,8554	-3,4243	0,295398	-0,02542	-75,0476	-57,2797	7,10016
BOx6	8	Done	144,4	-6,13911	glide-grid_4LVT	-6,13911	-2,7441	-0,00298	0	-0,2644	-50,7227	-4,82982	0,369602	-0,23662	-73,6122	-55,5525	12,4015
BTzPh5	237	Done	76,07	-6,12824	glide-grid_4LVT	-7,17664	-2,94236	-0,51579	0	-0,13749	-47,1982	-8,2793	0,278558	-0,25775	-84,4078	-55,4775	8,26945
BT2	58	Done	52,13	-6,12301	glide-grid_4LVT	-6,12301	-2,9265	0	0	-0,41895	-57,4669	-0,42571	0,180737	-0,02109	-74,9643	-57,8926	9,94777
BTzPhDMN9	386	Done	69,94	-6,11327	glide-grid_4LVT	-7,16547	-1,77936	-0,73901	0	-0,70495	-58,2892	-8,18237	0,260195	-0,06054	-89,7366	-66,4715	9,79799
BOxPhOMe9	56	Done	78,86	-6,10067	glide-grid_4LVT	-6,10067	-2,11164	-0,32	0	-0,08027	-56,1727	-6,03856	0,278691	-0,15303	-81,5928	-62,2113	11,7824
BTzPhOMe3	411	Done	88,76	-6,10065	glide-grid_4LVT	-7,72665	-3,98214	0	0	-0,35432	-59,4728	-3,23138	0,18426	-0,11611	-94,7622	-62,7042	7,96744

BTz7	116	Done	100,99	-6,07125	glide-grid_4LVT	-8,42615	-3,08	-0,16	0	-1,12634	-59,5331	-6,99005	0,390204	-0,42486	-96,5577	-66,5231	17,1855
BTzPh6	245	Done	82,29	-6,03139	glide-grid_4LVT	-7,47959	-3,43554	-0,21165	0	-0,79049	-53,1349	-4,05306	0,294136	-0,07134	-82,0602	-57,188	7,64148
BOxNaf7	18	Done	60,84	-6,0148	glide-grid_4LVT	-6,0148	-2,85028	0	0	-0,13033	-57,5867	-2,14358	0,223264	-0,05659	-78,3313	-59,7303	6,92377
BOxPh8	28	Done	75,68	-5,97989	glide-grid_4LVT	-5,97989	-2,13644	-0,22529	0	-0,13497	-56,2419	-5,1873	0,319509	-0,21251	-80,4823	-61,4292	9,23573
BOxPhOMe8	55	Done	78,33	-5,97943	glide-grid_4LVT	-5,97943	-2,15149	0	0	-0,01364	-63,2391	-5,12639	0,266935	-0,15032	-84,7941	-68,3655	20,015
BT1	57	Done	30,38	-5,96726	glide-grid_4LVT	-5,96726	-2,96424	0	0	-0,203	-56,4715	-0,32827	0,143227	-0,07044	-73,2432	-56,7997	8,50963
BOx7	9	Done	122,73	-5,96381	glide-grid_4LVT	-5,96381	-2,97241	-0,0201	0	-0,36574	-55,8466	-1,0196	0,387492	-0,04778	-73,7525	-56,8662	9,31528
BOx8	10	Done	91,91	-5,94998	glide-grid_4LVT	-5,94998	-2,40401	0	0	-0,17036	-54,3879	-5,84836	0,402729	-0,18169	-77,4631	-60,2363	13,4257
BTzPhCl4	292	Done	80,17	-5,94823	glide-grid_4LVT	-7,33433	-3,56071	-0,16602	0	-0,40448	-55,2699	-3,29248	0,197663	-0,14342	-81,24	-58,5624	9,64227
BTzNaf5	170	Done	66,2	-5,94779	glide-grid_4LVT	-8,38709	-4,4103	0	0	-0,61299	-62,4642	-2,30216	0,193712	-0,08899	-98,122	-64,7664	8,12673
BOxPhDMN4	42	Done	63,5	-5,93354	glide-grid_4LVT	-5,93354	-2,01346	-0,25086	0	-0,21913	-53,8183	-5,80396	0,182995	-0,07158	-76,0276	-59,6222	9,89042
BTzNaf3	150	Done	68,9	-5,92755	glide-grid_4LVT	-6,52175	-1,87997	-0,61872	0	-0,21583	-46,7127	-9,16593	0,154763	-0,25146	-85,7507	-55,8787	7,19618
BTzPh6	247	Done	84,77	-5,89503	glide-grid_4LVT	-8,34573	-3,87222	-0,19216	0	-0,88377	-53,7451	-5,85215	0,295128	-0,12763	-95,5251	-59,5972	5,41491
BOxNaf2	13	Done	64,86	-5,88629	glide-grid_4LVT	-5,88629	-2,03338	-0,30763	0	-0,10741	-53,2016	-4,63325	0,12855	-0,21136	-78,4311	-57,8348	4,22525
BOxNaf1	12	Done	53,54	-5,87527	glide-grid_4LVT	-5,87527	-2,35176	0	0	-0,22509	-58,8881	-2,78605	0,102315	-0,03843	-81,1959	-61,6742	10,5505

BTz2	74	Done	69,37	-5,86502	glide-grid_4LVT	-6,44302	-2,03253	-0,32	0	-1,06971	-51,6268	-4,42992	0,264611	-0,03955	-75,7226	-56,0568	5,90078
BTz6	106	Done	113,16	-5,8309	glide-grid_4LVT	-6,7731	-2,10504	-0,61368	0	-0,08333	-40,3225	-12,7513	0,373771	-0,41599	-77,44	-53,0738	10,5601
BTzPhOMe6	439	Done	76,63	-5,82952	glide-grid_4LVT	-7,46542	-3,47493	-0,2596	0	-0,38881	-54,9049	-4,77344	0,241209	-0,12203	-89,0634	-59,6784	10,2698
BOxPhDMN7	45	Done	70,12	-5,82501	glide-grid_4LVT	-5,82501	-1,57986	-0,16	0	-0,19338	-60,0325	-6,33788	0,233478	-0,17294	-84,7484	-66,3704	11,2221
BTzPh6	243	Done	82,98	-5,8138	glide-grid_4LVT	-6,5939	-2,12532	-0,3992	0	-0,1392	-46,6318	-10,7997	0,295128	-0,27376	-80,61	-57,4315	16,157
BTzNaf4	158	Done	70,75	-5,79771	glide-grid_4LVT	-6,36441	-1,49117	-0,52255	0	-0,35676	-53,7761	-9,59709	0,175718	-0,04129	-83,6229	-63,3732	20,5119
BTzPhOMe1	393	Done	46,03	-5,78358	glide-grid_4LVT	-7,03738	-2,72035	-0,22234	0	-0,57918	-47,4925	-7,61214	0,132423	-0,13149	-77,5199	-55,1047	5,2489
BOx2	4	Done	87,03	-5,77237	glide-grid_4LVT	-5,77237	-2,811	0	0	-0,14664	-53,4401	-2,32364	0,261818	-0,056	-73,4628	-55,7637	5,38512
BTzPhDMN7	370	Done	54,25	-5,76153	glide-grid_4LVT	-6,58383	-2,15949	-0,32	0	-0,06889	-47,7424	-10,5134	0,235707	-0,30704	-85,0504	-58,2558	7,34836
BTzPhOMe5	427	Done	78,96	-5,75757	glide-grid_4LVT	-6,77247	-1,81039	-0,29514	0	-0,52741	-48,9635	-11,3973	0,224435	-0,2062	-85,0373	-60,3607	10,8633
BTz8	122	Done	75,56	-5,74726	glide-grid_4LVT	-6,69056	-2,20182	-0,32	0	-1,35365	-59,6425	-0,9777	0,406764	-0,09307	-83,6574	-60,6202	9,39528
BTzPh3	221	Done	92,4	-5,74109	glide-grid_4LVT	-6,76479	-2,39107	-0,60039	0	-0,25939	-41,3259	-10,125	0,238112	-0,16701	-74,2872	-51,4509	7,0638
BTz9	130	Done	74,78	-5,72133	glide-grid_4LVT	-6,66463	-1,90384	-0,68991	0	-0,38344	-43,4496	-11,7489	0,419624	-0,17225	-78,174	-55,1984	9,4102
BOxPh1	21	Done	73,78	-5,71762	glide-grid_4LVT	-5,71762	-2,61325	-0,4573	0	-0,22341	-48,194	-1,00813	0,182605	-0,04536	-63,5448	-49,2022	5,07295
BOxPh4	24	Done	74,81	-5,71635	glide-grid_4LVT	-5,71635	-1,97028	-0,33117	0	-0,07471	-48,7992	-6,61805	0,256709	-0,16423	-73,9905	-55,4172	5,55073

BTzNaf2	147	Done	71,92	-5,71471	glide-grid_4LVT	-6,94151	-2,2664	-0,02014	0	-0,55564	-63,5808	-6,60965	0,1299	-0,05874	-97,2808	-70,1904	6,86643
BTzPhOMe7	444	Done	85,01	-5,71045	glide-grid_4LVT	-6,72455	-2,10694	-0,43006	0	0	-46,792	-12,0726	0,256104	-0,29317	-87,8197	-58,8645	8,0343
BT9	65	Done	67,07	-5,70559	glide-grid_4LVT	-5,70559	-2,12488	-0,27815	0	-0,42023	-55,8233	-2,57427	0,337282	-0,04231	-76,7912	-58,3976	9,72216
BTzPh6	244	Done	67,16	-5,70258	glide-grid_4LVT	-6,69338	-2,67539	-0,16	0	-0,82199	-51,4495	-3,6441	0,295128	-0,21204	-81,5638	-55,0936	5,76384
BTzPhDMN9	383	Done	76,53	-5,69742	glide-grid_4LVT	-6,31792	-1,57795	-0,71225	0	-0,0356	-47,03	-11,747	0,261694	-0,14026	-83,9459	-58,777	9,51878
BTzNaf2	146	Done	51,81	-5,68887	glide-grid_4LVT	-6,76117	-2,6466	-0,29096	0	-0,12766	-48,0124	-8,17603	0,130594	-0,19952	-82,9906	-56,1884	4,11099
BTzPh6	248	Done	84,01	-5,66941	glide-grid_4LVT	-8,20151	-3,21586	-0,5421	0	-0,24638	-54,8676	-10,2274	0,294136	-0,2138	-93,2429	-65,095	16,7594
BT7	63	Done	65,86	-5,66038	glide-grid_4LVT	-5,66038	-1,80595	0	0	-0,69549	-61,0767	-2,68917	0,306732	-0,00846	-78,8457	-63,7659	13,5975
BOxNaf9	20	Done	69,02	-5,65956	glide-grid_4LVT	-5,65956	-2,64495	0	0	-0,19104	-57,5349	-1,30301	0,248626	0	-76,2301	-58,8379	7,52223
BOxNaf8	19	Done	63,45	-5,65883	glide-grid_4LVT	-5,65883	-2,18341	-0,138	0	-0,0412	-64,4685	-1,91847	0,23665	-0,02167	-85,5978	-66,387	7,0095
BTzNaf1	139	Done	48,89	-5,65397	glide-grid_4LVT	-6,91077	-2,35457	-0,48858	0	-0,2828	-48,3282	-9,19585	0,103626	-0,09266	-83,8322	-57,5241	4,40556
BTzNaf1	137	Done	51,9	-5,64724	glide-grid_4LVT	-6,39084	-1,75211	-0,16	0	-0,64956	-52,7098	-7,67105	0,1043	-0,14732	-81,5277	-60,3808	13,432
BTz2	75	Done	72,14	-5,63745	glide-grid_4LVT	-6,43745	-1,98964	-0,45022	0	-0,15006	-40,1172	-12,3997	0,26605	-0,24777	-70,2822	-52,5169	16,2769
BTzNaf6	175	Done	61,44	-5,6309	glide-grid_4LVT	-6,6217	-2,37702	-0,28988	0	-0,17113	-57,3634	-6,45996	0,210443	-0,15694	-90,2174	-63,8233	12,7437
BTz3	82	Done	90,19	-5,63057	glide-grid_4LVT	-6,57997	-2,04522	-0,46943	0	-0,19666	-42,154	-12,0307	0,299653	-0,25601	-74,3492	-54,1847	14,9526

BOxPhOMe4	51	Done	59,34	-5,62295	glide-grid_4LVT	-5,62295	-2,27522	0	0	-0,01195	-57,1525	-3,27706	0,203133	-0,18973	-74,9949	-60,4296	19,3668
BTzNaf2	145	Done	75,8	-5,61522	glide-grid_4LVT	-6,47312	-2,11217	-0,43123	0	-0,29203	-55,7257	-5,40137	0,130594	-0,1718	-80,3833	-61,127	16,364
BTzPhCl9	320	Done	57,77	-5,60978	glide-grid_4LVT	-6,50938	-2,03154	-0,48092	0	-0,28042	-48,4617	-8,42453	0,274081	-0,30382	-81,676	-56,8862	8,20918
BTzNaf3	153	Done	69,1	-5,60747	glide-grid_4LVT	-6,94057	-1,9634	-0,456	0	-0,44955	-53,3426	-9,4566	0,153343	-0,13935	-87,9209	-62,7992	9,13804
BTzPhCl3	287	Done	95,73	-5,60279	glide-grid_4LVT	-8,13069	-4,25391	0	0	-0,64039	-60,3959	-2,13946	0,17724	-0,07292	-91,1998	-62,5354	13,3177
BOxPh5	25	Done	102,74	-5,59074	glide-grid_4LVT	-5,59074	-1,79311	-0,24941	0	-0,09167	-47,2521	-7,66304	0,275615	-0,2201	-74,4567	-54,9152	4,38763
BOxPhDMN5	43	Done	78,2	-5,57564	glide-grid_4LVT	-5,57564	-1,95523	-0,16	0	-0,20769	-51,3384	-4,82767	0,201769	-0,16343	-73,5576	-56,166	5,88557
BTzPhOMe1	397	Done	45,05	-5,5678	glide-grid_4LVT	-7,5422	-4,17736	0	0	-0,25369	-57,851	-2,10717	0,132423	-0,03495	-83,7407	-59,9582	8,264
BTzPhDMN6	363	Done	66,43	-5,54506	glide-grid_4LVT	-6,36736	-1,87243	-0,63956	0	-0,09194	-48,905	-8,64406	0,220756	-0,24233	-86,368	-57,5491	6,96618
BTzNaf6	174	Done	61,88	-5,51931	glide-grid_4LVT	-6,29941	-2,15214	-0,43283	0	-0,31089	-52,9102	-5,75497	0,210443	-0,10524	-82,1664	-58,6651	8,56389
BTzPhDMN3	342	Done	53,14	-5,50964	glide-grid_4LVT	-6,84274	-1,66091	-0,48954	0	-0,40021	-51,1517	-11,2687	0,163391	-0,20757	-83,2524	-62,4204	16,3044
BTzPhOMe7	443	Done	71,24	-5,50101	glide-grid_4LVT	-6,14911	-1,88363	-0,83547	0	-0,175	-41,1248	-8,1323	0,256924	-0,23585	-73,4126	-49,2571	11,6161
BTzPhCl6	305	Done	75,47	-5,4836	glide-grid_4LVT	-8,0299	-3,63076	-0,28468	0	-0,58875	-55,3407	-5,53975	0,234095	-0,16181	-94,158	-60,8805	9,47716
BTzNaf7	182	Done	58,28	-5,47611	glide-grid_4LVT	-6,25621	-2,02284	-0,60187	0	-0,03266	-46,8762	-8,4962	0,225404	-0,20599	-78,6244	-55,3724	7,53871
BTzPhOMe6	441	Done	81,08	-5,47249	glide-grid_4LVT	-7,47439	-3,01388	-0,32	0	-0,61226	-52,8619	-6,83231	0,240388	-0,10069	-85,0002	-59,6942	8,68156

BTzNaf7	187	Done	53,16	-5,47202	glide-grid_4LVT	-8,00412	-2,84933	-0,28447	0	-0,46697	-54,455	-11,2658	0,224677	-0,21542	-93,6508	-65,7208	14,0708
BTz9	129	Done	70,64	-5,46181	glide-grid_4LVT	-6,40511	-2,25921	-0,16	0	-1,09932	-52,0197	-4,04445	0,419624	-0,09856	-72,2793	-56,0641	23,2584
BTzNaf7	185	Done	61,78	-5,46042	glide-grid_4LVT	-7,84092	-3,58849	-0,33258	0	-0,47327	-55,3631	-5,09874	0,225404	-0,13902	-94,6916	-60,4618	9,68721
BTzPhCl1	275	Done	69,19	-5,43644	glide-grid_4LVT	-6,33394	-1,9572	-0,32	0	-0,31613	-50,722	-8,06918	0,126457	-0,12059	-78,9657	-58,7912	11,5198
BOxPhOMe5	52	Done	76,29	-5,43436	glide-grid_4LVT	-5,43436	-2,23059	-0,10597	0	-0,00127	-58,0045	-2,55561	0,222015	-0,03499	-75,499	-60,5602	14,074
BTzPhOMe1	392	Done	49,92	-5,4219	glide-grid_4LVT	-6,4891	-3,04066	-0,28301	0	-0,17768	-51,0344	-3,36533	0,133203	-0,06444	-80,367	-54,3997	4,28792
BT4	60	Done	80,95	-5,41595	glide-grid_4LVT	-5,41595	-2,03236	0	0	-0,46275	-54,5351	-2,75626	0,241723	-0,02236	-71,3268	-57,2914	10,8284
BTz6	111	Done	116,53	-5,4079	glide-grid_4LVT	-7,9519	-2,61689	-0,39312	0	-0,95736	-53,3742	-9,53645	0,372355	-0,25771	-91,7979	-62,9107	12,4609
BTzNaf5	168	Done	66,34	-5,40757	glide-grid_4LVT	-6,45597	-2,01004	-0,57332	0	0	-47,7018	-9,45446	0,193712	-0,26307	-83,3357	-57,1563	10,1322
BTzPhOMe5	430	Done	82,42	-5,40719	glide-grid_4LVT	-7,04759	-3,18299	-0,32	0	-0,12924	-54,8541	-5,0821	0,224435	-0,13477	-84,3148	-59,9362	18,171
BTzPhDMN3	341	Done	48,72	-5,40551	glide-grid_4LVT	-6,42921	-2,10131	-0,4824	0	-0,13806	-46,3314	-8,97422	0,164133	-0,20887	-82,1677	-55,3056	7,14116
BTzPh5	238	Done	85,49	-5,39881	glide-grid_4LVT	-6,46271	-2,02935	-0,456	0	-0,00011	-49,9713	-9,82573	0,277558	-0,28239	-82,3017	-59,797	8,11283
BOxPhDMN1	39	Done	42,69	-5,39867	glide-grid_4LVT	-5,39867	-2,34928	0	0	-0,05759	-48,4089	-4,1133	0,111822	-0,06619	-67,6109	-52,5222	4,18927
BTzPhOMe4	421	Done	74,02	-5,39816	glide-grid_4LVT	-7,04256	-2,12464	-0,52032	0	-0,3247	-47,3833	-11,8411	0,205543	-0,13311	-86,1081	-59,2244	8,22132
BTzPhCl1	278	Done	59,85	-5,39582	glide-grid_4LVT	-7,88192	-4,07973	0	0	-0,66604	-58,4921	-2,13691	0,126457	-0,01747	-88,6442	-60,629	4,8933

BTzPh9	268	Done	67,61	-5,39428	glide-grid_4LVT	-6,38508	-2,01905	-0,456	0	-0,15728	-52,9151	-8,99414	0,333491	-0,09138	-83,9616	-61,9092	14,0513
BTzPhDMN2	336	Done	54,78	-5,39144	glide-grid_4LVT	-6,61824	-2,0502	-0,33464	0	-0,31391	-50,998	-8,55737	0,139751	-0,22573	-80,3919	-59,5554	8,29027
BTzPh1	207	Done	65,35	-5,39135	glide-grid_4LVT	-6,36545	-2,17376	-0,26437	0	-0,67978	-41,9048	-7,96719	0,18549	-0,14272	-70,4974	-49,872	4,75782
BTzPh9	272	Done	56,06	-5,39031	glide-grid_4LVT	-7,92241	-3,24726	-0,32	0	-0,67958	-52,514	-8,05196	0,332533	-0,17461	-90,1412	-60,566	8,18645
BTzPhOMe5	431	Done	86,37	-5,3872	glide-grid_4LVT	-7,4037	-1,9966	-0,49018	0	-0,22462	-53,5119	-13,4584	0,223613	-0,22155	-91,3074	-66,9703	14,2513
BTzPhDMN7	372	Done	63,8	-5,38699	glide-grid_4LVT	-6,43919	-1,75195	-0,16	0	-0,16885	-61,9406	-8,2913	0,23495	-0,25262	-94,61	-70,2319	7,37217
BOxPhCl8	37	Done	79,39	-5,38445	glide-grid_4LVT	-5,38445	-2,02095	-0,15258	0	0	-62,2799	-2,04156	0,259952	-0,05064	-79,1354	-64,3215	14,9583
BTzPhCl1	277	Done	64,55	-5,38041	glide-grid_4LVT	-6,53771	-2,1692	-0,16	0	-0,51192	-52,7792	-7,16859	0,125702	-0,10805	-78,595	-59,9478	13,7102
BTz7	119	Done	109,35	-5,37624	glide-grid_4LVT	-7,92124	-2,82408	-0,35791	0	-0,95312	-55,8135	-7,8682	0,390204	-0,20542	-92,9465	-63,6817	9,11473
BTzNaf4	162	Done	56,9	-5,36633	glide-grid_4LVT	-7,74363	-3,52323	-0,16607	0	-0,60698	-49,5044	-6,58177	0,174997	-0,15987	-85,0256	-56,0862	4,69615
BTz5	101	Done	109,91	-5,36301	glide-grid_4LVT	-7,72941	-2,46655	-0,11114	0	-1,08563	-53,6568	-10,0202	0,351423	-0,23165	-88,7033	-63,677	14,0253
BTzPhCl1	276	Done	64,65	-5,34335	glide-grid_4LVT	-6,32495	-1,99526	-0,29812	0	-0,5444	-40,1306	-9,66063	0,126457	-0,15799	-69,6464	-49,7913	4,44709
BTzPhCl9	321	Done	56,6	-5,34311	glide-grid_4LVT	-6,48881	-1,55968	-0,5346	0	-0,12363	-54,6387	-10,556	0,274081	-0,22964	-85,4755	-65,1947	26,4237
BTzPhDMN7	369	Done	83,43	-5,33751	glide-grid_4LVT	-5,95801	-1,54868	-0,65104	0	-0,00996	-48,0337	-9,06451	0,236467	-0,22344	-81,9675	-57,0982	8,20437
BTzPhOMe3	414	Done	72,63	-5,30967	glide-grid_4LVT	-7,28247	-2,49881	-0,47486	0	-0,47978	-46,6819	-10,2927	0,183449	-0,13447	-80,1105	-56,9746	10,5409

BTzNaf8	191	Done	68,97	-5,30412	glide-grid_4LVT	-6,29492	-1,85549	-0,54528	0	-0,06035	-56,4815	-7,26792	0,238783	-0,15832	-87,743	-63,7495	9,09821
BOxPhOMe7	54	Done	64,12	-5,30298	glide-grid_4LVT	-5,30298	-1,6085	-0,48283	0	0	-43,4415	-7,89068	0,253695	-0,10967	-63,8646	-51,3322	11,9275
BTzPhDMN4	354	Done	56,08	-5,28577	glide-grid_4LVT	-7,95197	-2,4247	-0,38458	0	-0,62157	-60,9987	-10,0902	0,184456	-0,14211	-99,4533	-71,0889	14,3683
BTzNaf7	188	Done	68,47	-5,28459	glide-grid_4LVT	-7,95719	-2,43234	-0,54028	0	-0,00439	-60,0323	-13,2582	0,224677	-0,21453	-105,034	-73,2904	11,6078
BTzNaf9	198	Done	46,14	-5,27422	glide-grid_4LVT	-6,05432	-2,31008	-0,32	0	0	-44,655	-8,49523	0,250747	-0,16795	-74,6236	-53,1502	5,34201
BTzPh8	258	Done	46,27	-5,26994	glide-grid_4LVT	-5,83064	-2,5431	-0,48242	0	0	-41,7702	-5,35095	0,323344	-0,23732	-67,6466	-47,1212	10,7771
BTzPhOMe2	402	Done	71,44	-5,2673	glide-grid_4LVT	-6,8089	-2,253	-0,32	0	-0,27513	-52,1976	-9,00153	0,15947	-0,16013	-84,1135	-61,1992	7,53648
BTzNaf8	190	Done	53,75	-5,26406	glide-grid_4LVT	-6,04416	-1,6441	-0,69334	0	-0,34539	-46,5555	-7,56671	0,238783	-0,13733	-78,0824	-54,1222	6,28427
BTzPhDMN4	349	Done	54,08	-5,26048	glide-grid_4LVT	-6,24398	-1,89767	-0,52591	0	-0,08556	-47,6361	-8,79099	0,185208	-0,21961	-82,3863	-56,4271	5,04353
BTzNaf8	196	Done	75,22	-5,25432	glide-grid_4LVT	-7,92692	-3,40424	-0,269	0	-0,65824	-53,3496	-6,78247	0,238059	-0,14865	-89,2329	-60,1321	2,96927
BTzPh9	271	Done	60,35	-5,25262	glide-grid_4LVT	-7,70332	-2,74327	-0,50693	0	-0,69907	-54,8475	-7,92558	0,333491	-0,15633	-84,9316	-62,7731	23,9991
BTzPh1	206	Done	70,91	-5,25259	glide-grid_4LVT	-5,99619	-2,07472	-0,23598	0	-0,49346	-51,2968	-5,2327	0,18549	-0,02778	-75,2141	-56,5295	9,82625
BOxPhDMN6	44	Done	68,19	-5,25188	glide-grid_4LVT	-5,25188	-1,94366	-0,1944	0	-0,18337	-54,3327	-2,83578	0,218523	-0,00697	-74,2461	-57,1685	4,13632
BTz3	81	Done	83,61	-5,25048	glide-grid_4LVT	-6,18098	-2,2157	-0,31955	0	-0,88402	-51,774	-1,74605	0,299653	-0,21076	-70,0396	-53,52	12,1245
BTzPhOMe4	419	Done	66,34	-5,24768	glide-grid_4LVT	-6,61498	-1,95543	-0,456	0	-0,57054	-49,9269	-7,92588	0,204725	-0,15251	-81,619	-57,8527	5,07003

BOxNaf5	16	Done	72,7	-5,23648	glide-grid_4LVT	-5,23648	-2,07119	0	0	-0,03319	-61,0728	-1,15221	0,19158	-0,09721	-76,3706	-62,225	13,1035
BTzNaf8	189	Done	61,58	-5,22868	glide-grid_4LVT	-5,78938	-1,58726	-0,32	0	-0,24018	-49,3187	-7,87778	0,23951	-0,23384	-80,1606	-57,1965	7,12933
BTzNaf6	176	Done	62,91	-5,22624	glide-grid_4LVT	-6,67444	-1,68738	-0,31137	0	-0,36028	-55,3604	-10,444	0,209716	-0,19051	-93,6194	-65,8044	3,64865
BTzPhDMN2	335	Done	37,98	-5,22317	glide-grid_4LVT	-6,29547	-1,96378	-0,46058	0	-0,27356	-48,3372	-7,56946	0,14048	-0,18575	-75,3216	-55,9067	17,8508
BTzPh2	214	Done	100,55	-5,22232	glide-grid_4LVT	-6,08022	-1,89142	-0,32849	0	-0,09258	-44,1564	-10,2281	0,213536	-0,23924	-77,3121	-54,3844	6,35052
BTzPhDMN5	358	Done	57,81	-5,21708	glide-grid_4LVT	-6,28098	-2,43982	-0,18103	0	-0,10874	-54,708	-5,87003	0,203241	-0,13874	-84,3108	-60,578	5,95605
BTzPhOMe5	426	Done	69,9	-5,21541	glide-grid_4LVT	-6,23031	-1,65989	-0,48	0	-0,33668	-51,3642	-7,31655	0,224435	-0,31248	-75,1803	-58,6807	19,5729
BTz1	66	Done	52,19	-5,21512	glide-grid_4LVT	-5,69892	-1,98671	0	0	-1,01387	-51,3358	-2,14609	0,225258	-0,0349	-68,0339	-53,4819	7,59521
BTzNaf8	195	Done	53,62	-5,20989	glide-grid_4LVT	-7,74199	-3,61661	-0,21712	0	-0,62924	-58,2521	-3,75678	0,238059	-0,04095	-88,102	-62,0089	8,54722
BTzPh5	236	Done	89,26	-5,20352	glide-grid_4LVT	-6,02722	-1,55947	-0,44543	0	-0,16849	-41,6198	-12,0938	0,278558	-0,23735	-72,8778	-53,7136	8,53461
BTzNaf4	165	Done	74,16	-5,20054	glide-grid_4LVT	-7,86674	-3,91555	-0,16	0	-0,58571	-43,5429	-6,99669	0,174279	-0,15311	-73,9307	-50,5396	2,34298
BTzPhOMe6	440	Done	70,88	-5,191	glide-grid_4LVT	-7,1929	-2,91932	-0,32	0	-0,20962	-59,4586	-5,98871	0,240388	-0,11311	-91,0067	-65,4473	8,53986
BTzPhCl2	280	Done	82,63	-5,18433	glide-grid_4LVT	-6,13313	-1,90634	-0,30028	0	-0,10833	-42,778	-10,4493	0,153359	-0,26523	-76,9443	-53,2273	4,92641
BTzPhDMN5	356	Done	61,96	-5,18342	glide-grid_4LVT	-6,00712	-2,18341	-0,28984	0	-0,15729	-52,6628	-5,43076	0,203997	-0,13283	-82,233	-58,0936	5,90092
BTzNaf7	181	Done	73,32	-5,1733	glide-grid_4LVT	-5,734	-1,76189	-0,69325	0	0	-41,8052	-7,38476	0,226133	-0,30701	-69,2634	-49,19	12,9294

BTzPh4	228	Done	79,47	-5,14769	glide-grid_4LVT	-5,92869	-1,79066	-0,50654	0	-0,05251	-37,0179	-11,5554	0,25966	-0,25443	-66,7151	-48,5733	9,4084
BTzPhOMe5	428	Done	81,72	-5,13197	glide-grid_4LVT	-6,52297	-1,94261	-0,54947	0	-0,00331	-49,8305	-10,1099	0,223613	-0,24317	-77,2181	-59,9404	20,8133
BTzPhOMe2	399	Done	67,04	-5,13093	glide-grid_4LVT	-5,65243	-2,16106	-0,54956	0	0	-32,9789	-8,44989	0,161071	-0,18646	-59,4149	-41,4288	8,12739
BTz9	135	Done	96,3	-5,12878	glide-grid_4LVT	-7,67378	-2,10755	-0,42317	0	-1,12694	-57,1613	-9,82059	0,41828	-0,10325	-90,2574	-66,9819	16,2157
BTz7	112	Done	101,79	-5,12297	glide-grid_4LVT	-5,58177	-1,51946	-0,16	0	-0,44903	-53,786	-6,17522	0,390204	-0,2279	-76,6444	-59,9612	11,3692
BTzPhOMe5	432	Done	68,21	-5,11997	glide-grid_4LVT	-7,13647	-3,29952	-0,32	0	-0,14657	-53,9947	-5,30273	0,223613	-0,09884	-87,0626	-59,2975	7,30945
BTzPhCl3	284	Done	94,05	-5,11909	glide-grid_4LVT	-6,01409	-1,94344	-0,28218	0	-0,30236	-55,1469	-4,68131	0,17724	-0,20381	-79,9227	-59,8283	7,36949
BOxNaf6	17	Done	43,49	-5,1128	glide-grid_4LVT	-5,1128	-1,97828	0	0	-0,1	-47,4871	-5,32423	0,208302	-0,06983	-65,9593	-52,8113	8,36173
BTz9	128	Done	58,78	-5,10842	glide-grid_4LVT	-5,56712	-1,73852	-0,41305	0	-0,28966	-46,1294	-7,52552	0,41828	-0,10888	-70,8617	-53,6549	6,48228
BTzPhDMN4	348	Done	63,62	-5,10699	glide-grid_4LVT	-5,88799	-1,45077	-0,44449	0	-0,09049	-42,5889	-11,8372	0,185208	-0,18242	-73,6356	-54,4261	10,0187
BTzPhOMe3	412	Done	75,3	-5,106	glide-grid_4LVT	-6,7581	-2,17083	-0,45707	0	-0,36925	-48,3741	-8,48066	0,18426	-0,25439	-80,0525	-56,8548	12,2073
BTzNaf9	204	Done	56,18	-5,09915	glide-grid_4LVT	-7,77175	-3,52619	-0,16	0	-0,67779	-55,5507	-4,69271	0,250027	-0,17636	-89,6923	-60,2434	3,21353
BTzPh7	256	Done	71,1	-5,09486	glide-grid_4LVT	-7,62696	-2,28564	-0,59991	0	-0,7204	-59,2881	-8,71324	0,308669	-0,05829	-91,5121	-68,0013	14,4462
BTzPh3	220	Done	102,79	-5,08658	glide-grid_4LVT	-5,84138	-1,54802	-0,32	0	-0,77649	-57,0892	-3,68786	0,238112	-0,02734	-74,4954	-60,7771	17,1208
BTzPh2	215	Done	86,14	-5,08362	glide-grid_4LVT	-6,15592	-1,99943	-0,44216	0	-0,23892	-43,5584	-8,52442	0,213536	-0,23236	-73,0485	-52,0829	12,5436

BTzPhDMN5	355	Done	72,62	-5,07187	glide-grid_4LVT	-5,68347	-1,39458	-0,47831	0	-0,05623	-46,4494	-9,30252	0,204756	-0,24126	-79,1335	-55,752	9,51749
BTzPhOMe4	418	Done	81,02	-5,06424	glide-grid_4LVT	-6,07984	-2,39901	-0,31277	0	-0,30838	-47,8315	-4,91255	0,205543	-0,13677	-70,2248	-52,7441	15,5929
BTzPhCl3	283	Done	97,22	-5,05648	glide-grid_4LVT	-5,47448	-1,76493	-0,39964	0	-0,01307	-46,0906	-5,46939	0,17803	-0,34993	-74,9792	-51,56	5,96023
BOxPhCl9	38	Done	54,39	-5,05156	glide-grid_4LVT	-5,05156	-2,20799	0	0	0	-53,0361	-2,01438	0,271767	-0,16138	-69,9441	-55,0504	6,17879
BTz4	93	Done	121,56	-5,0459	glide-grid_4LVT	-7,4188	-1,62944	-0,42584	0	-1,94912	-52,3878	-6,57972	0,326906	-0,13496	-83,9759	-58,9675	6,28784
BTzPhCl5	297	Done	85,44	-5,04431	glide-grid_4LVT	-6,19491	-2,59333	0	0	-0,84991	-58,3837	0,113813	0,21732	-0,06688	-78,2071	-58,2699	10,5447
BOx1	3	Done	71,9	-5,03828	glide-grid_4LVT	-5,03828	-2,28238	0	0	-0,12291	-49,7854	-2,19509	0,222507	-0,03695	-65,6855	-51,9805	6,98453
BTzPhDMN1	331	Done	33,78	-5,03533	glide-grid_4LVT	-7,63253	-3,18603	-0,21409	0	-1,32273	-53,9542	-2,05337	0,113909	-0,01787	-82,9993	-56,0075	4,33903
BTzPhDMN1	326	Done	41,49	-5,03251	glide-grid_4LVT	-5,77611	-1,91692	-0,32	0	-0,1285	-42,4262	-7,54024	0,113909	-0,27225	-68,4687	-49,9665	8,34359
BTzPh6	242	Done	68,44	-5,0253	glide-grid_4LVT	-5,586	-1,76894	-0,66695	0	0	-37,2414	-9,08622	0,296124	-0,22123	-66,1332	-46,3276	9,45862
BTzPh3	226	Done	90,76	-5,02366	glide-grid_4LVT	-7,70286	-3,48997	-0,17708	0	-0,77916	-47,4646	-6,54688	0,237111	-0,13849	-79,8284	-54,0115	5,79326
BTz4	89	Done	118,99	-5,01636	glide-grid_4LVT	-5,95496	-1,85263	-0,72574	0	0	-32,5323	-12,4731	0,328349	-0,20736	-63,5949	-45,0054	12,3102
BTzPhCl3	285	Done	96,46	-5,01235	glide-grid_4LVT	-6,13395	-2,39211	-0,16	0	-0,00251	-45,9937	-8,08104	0,17724	-0,24474	-80,0592	-54,0747	3,59717
BTzPhOMe3	408	Done	79,53	-5,01034	glide-grid_4LVT	-6,00664	-1,97974	-0,38553	0	-0,20787	-48,0731	-7,04436	0,18426	-0,15746	-77,9662	-55,1174	10,4202
BTzPh7	250	Done	74,72	-4,99547	glide-grid_4LVT	-5,55617	-1,64404	-0,32412	0	-0,1561	-51,0671	-7,10127	0,310638	-0,124	-79,5003	-58,1684	11,2454

BOxPh7	27	Done	65,04	-4,98116	glide-grid_4LVT	-4,98116	-1,75436	0	0	0	-56,2995	-4,4993	0,306757	-0,04368	-77,5705	-60,7988	5,21361
BTzPhOMe7	446	Done	73,26	-4,97606	glide-grid_4LVT	-6,35626	-2,08842	-0,28165	0	-0,07974	-58,1673	-7,20437	0,255286	-0,1727	-85,7517	-65,3717	13,5578
BTzNaf4	160	Done	58,17	-4,96841	glide-grid_4LVT	-5,95191	-1,80125	-0,48803	0	-0,07441	-47,6254	-7,70449	0,174997	-0,22627	-81,5892	-55,3299	7,23608
BTz4	92	Done	124,6	-4,94507	glide-grid_4LVT	-7,31387	-2,6166	0	0	-1,05641	-54,7669	-6,02863	0,326906	-0,32513	-84,4067	-60,7956	12,3982
BTzPhCl6	303	Done	60,36	-4,9448	glide-grid_4LVT	-6,0905	-2,42573	-0,18351	0	0	-45,7726	-7,57885	0,234095	-0,2899	-76,3007	-53,3514	7,40193
BTzPhOMe7	447	Done	68,87	-4,93556	glide-grid_4LVT	-6,57146	-2,01727	-0,51783	0	-0,06581	-42,7801	-12,5758	0,256104	-0,20129	-78,6294	-55,3559	9,31879
BOxPhCl2	31	Done	84,25	-4,93132	glide-grid_4LVT	-4,93132	-1,76644	-0,26243	0	-0,14167	-51,4899	-1,61367	0,151079	-0,09531	-67,9753	-53,1036	2,95711
BOxPh9	29	Done	72,8	-4,9269	glide-grid_4LVT	-4,9269	-1,65398	-0,32	0	0	-46,829	-4,86906	0,33067	-0,21178	-63,4625	-51,6981	11,657
BTz7	113	Done	107,73	-4,9211	glide-grid_4LVT	-5,8644	-1,75532	-0,48438	0	-0,27735	-49,5217	-6,5463	0,391599	-0,28093	-76,2122	-56,068	10,7893
BTzPhCl7	310	Done	65,5	-4,91942	glide-grid_4LVT	-6,31972	-1,27467	-0,29247	0	-0,64042	-59,6809	-8,37466	0,248219	-0,12014	-90,7943	-68,0555	10,6825
BTzPhCl2	282	Done	71,99	-4,91674	glide-grid_4LVT	-6,49434	-2,52179	-0,32	0	-0,43128	-49,1665	-5,67242	0,152585	-0,06467	-74,7644	-54,8389	8,10759
BTz8	124	Done	79,16	-4,91623	glide-grid_4LVT	-7,27103	-2,67315	-0,23828	0	-1,08573	-52,3593	-6,31319	0,405393	-0,11432	-80,6743	-58,6725	12,7922
BTzNaf9	199	Done	63,61	-4,91519	glide-grid_4LVT	-5,90599	-1,15063	-0,53945	0	-0,09594	-41,5546	-13,9275	0,250747	-0,20386	-79,2603	-55,4821	5,94644
BTzPhCl7	308	Done	57,67	-4,90995	glide-grid_4LVT	-5,80955	-1,76867	-0,33737	0	-0,21429	-54,0142	-5,99039	0,249015	-0,13898	-81,6209	-60,0046	10,0453
BTzNaf3	152	Done	74,21	-4,9084	glide-grid_4LVT	-5,9321	-1,5676	-0,32	0	-0,26176	-53,6437	-6,92477	0,154052	-0,2159	-83,045	-60,5684	15,0504

BTzPhDMN7	375	Done	65,81	-4,9076	glide-grid_4LVT	-7,4878	-2,05645	-0,58333	0	-0,18876	-62,6679	-10,885	0,23495	-0,12807	-98,3025	-73,5529	16,3932
BTz6	104	Done	115,03	-4,90744	glide-grid_4LVT	-5,36714	-1,34122	-0,58069	0	-0,54468	-39,8071	-8,04461	0,372355	-0,07586	-65,0933	-47,8517	5,621
BTzPh7	251	Done	74,51	-4,89643	glide-grid_4LVT	-5,67653	-1,11189	-0,44529	0	-0,08842	-48,6658	-11,2854	0,309652	-0,21448	-80,3229	-59,9512	10,391
BTz5	102	Done	122,43	-4,89565	glide-grid_4LVT	-7,44665	-2,58482	-0,32511	0	-0,75518	-54,3058	-8,19635	0,351423	-0,18821	-85,2137	-62,5021	15,0379
BTzPh4	230	Done	99,71	-4,89473	glide-grid_4LVT	-6,33173	-2,27348	-0,47341	0	-0,05689	-51,7047	-6,90767	0,258658	-0,16522	-80,1725	-58,6124	7,96015
BOxPhDMN2	40	Done	66,49	-4,89332	glide-grid_4LVT	-4,89332	-1,5387	-0,16	0	-0,11817	-45,6763	-5,47438	0,138334	-0,10981	-64,1128	-51,1507	8,35183
BTzPhCl4	291	Done	83,57	-4,86428	glide-grid_4LVT	-6,00208	-1,80961	-0,4491	0	-0,01889	-45,6813	-9,43957	0,198458	-0,22293	-79,5331	-55,1209	8,05338
BTzPhOMe2	405	Done	73,69	-4,83778	glide-grid_4LVT	-7,04908	-2,36389	-0,65381	0	-0,53096	-41,0291	-9,6818	0,15947	-0,15617	-72,3774	-50,7109	6,49633
BTzNaf7	186	Done	52,55	-4,83439	glide-grid_4LVT	-7,28509	-2,8719	-0,16	0	-0,47741	-57,5404	-6,52818	0,225404	-0,14493	-91,7264	-64,0686	9,97739
BTzPhOMe4	417	Done	85,23	-4,8331	glide-grid_4LVT	-5,84	-1,86149	-0,19278	0	-0,08229	-46,6402	-9,35108	0,205543	-0,17432	-78,5924	-55,9913	9,15449
BTzPhDMN8	379	Done	78,04	-4,8242	glide-grid_4LVT	-5,8764	-1,75691	-0,22249	0	-0,11119	-54,7419	-7,30886	0,248293	-0,20068	-81,6802	-62,0507	9,63006
BTzPhCl8	318	Done	59,1	-4,82039	glide-grid_4LVT	-7,36669	-3,0382	-0,11997	0	-0,7214	-55,4104	-5,40434	0,262284	-0,16824	-87,6641	-60,8148	13,7155
BTzPh1	208	Done	65,7	-4,82015	glide-grid_4LVT	-6,07695	-1,48811	-0,42101	0	-0,25796	-42,4679	-11,1931	0,184509	-0,29202	-71,8096	-53,661	7,18551
BTzPhOMe5	425	Done	103,32	-4,81863	glide-grid_4LVT	-5,45743	-1,95292	-0,4658	0	0	-38,6275	-7,76615	0,225259	-0,16767	-68,3172	-46,3937	3,28833
BTzPhDMN4	350	Done	54,32	-4,81372	glide-grid_4LVT	-6,25072	-1,69513	-0,46679	0	-0,00859	-44,9117	-12,2832	0,184456	-0,17661	-78,1753	-57,1949	4,77125

BTzPhCl5	295	Done	94,25	-4,81234	glide-grid_4LVT	-5,20494	-1,77998	-0,4701	0	-0,08652	-41,3165	-5,79843	0,218122	-0,15088	-63,1849	-47,1149	12,8105
BTzNaf6	173	Done	48,49	-4,80986	glide-grid_4LVT	-5,37056	-2,3451	-0,17228	0	0	-43,6857	-3,22339	0,211172	-0,39656	-68,7953	-46,9091	9,38291
BTzNaf8	193	Done	53,82	-4,80124	glide-grid_4LVT	-7,18174	-2,40574	-0,48619	0	-0,20374	-55,7492	-9,69743	0,238783	-0,08278	-95,0191	-65,4466	9,12623
BTz8	121	Done	81,45	-4,78293	glide-grid_4LVT	-5,72623	-1,73477	-0,59527	0	-0,06114	-39,1837	-9,58633	0,406764	-0,34468	-67,4981	-48,7701	8,88957
BTzPhDMN3	339	Done	63,26	-4,77414	glide-grid_4LVT	-5,36834	-1,78026	-0,32	0	-0,10147	-38,0272	-7,52902	0,164879	-0,30078	-69,7126	-45,5562	4,30473
BOxPhDMN9	47	Done	57,15	-4,76968	glide-grid_4LVT	-4,76968	-1,76133	-0,15964	0	-0,01492	-48,6851	-3,22584	0,258739	-0,1744	-64,6448	-51,9109	6,58679
BTz5	97	Done	110,77	-4,76503	glide-grid_4LVT	-5,70063	-1,78428	-0,10848	0	-1,25253	-49,3606	-2,68377	0,352856	-0,0376	-70,0646	-52,0444	7,461
BTzPhCl9	319	Done	61,97	-4,76274	glide-grid_4LVT	-5,16164	-0,42951	-0,64	0	0	-42,307	-12,0372	0,274868	-0,44607	-69,8883	-54,3442	18,3066
BTzPh4	231	Done	65,62	-4,75577	glide-grid_4LVT	-7,13307	-2,99721	-0,42691	0	-0,39261	-46,42	-7,17686	0,25966	-0,17848	-81,4537	-53,5969	7,64963
BTzPhDMN6	362	Done	66,51	-4,75256	glide-grid_4LVT	-5,37306	-1,36329	-0,54935	0	-0,00814	-44,2575	-7,71887	0,221516	-0,30309	-71,9976	-51,9764	11,8247
BTzPhOMe2	400	Done	77,13	-4,75012	glide-grid_4LVT	-5,78102	-2,13955	-0,46338	0	-0,08529	-41,999	-6,17301	0,160269	-0,22716	-65,8601	-48,172	13,5394
BTzPhOMe9	462	Done	71,29	-4,74537	glide-grid_4LVT	-5,75947	-1,44934	-0,42769	0	0	-47,8865	-10,0075	0,281063	-0,26805	-81,6364	-57,8939	8,9565
BTzPhOMe9	463	Done	75,47	-4,7426	glide-grid_4LVT	-5,7567	-1,64693	-0,16105	0	-0,32977	-50,6957	-8,07186	0,281063	-0,15446	-76,1609	-58,7676	18,5921
BTzPh8	260	Done	70,54	-4,74226	glide-grid_4LVT	-5,73306	-1,78857	-0,52863	0	-0,03369	-47,2247	-7,81317	0,322369	-0,17134	-74,7278	-55,0379	8,96395
BTzNaf8	192	Done	72,24	-4,74114	glide-grid_4LVT	-6,18934	-1,74784	-0,41604	0	-0,16039	-46,9496	-10,6351	0,238059	-0,16039	-83,0388	-57,5847	4,52729

BTzPhOMe9	464	Done	91,61	-4,73641	glide-grid_4LVT	-6,11661	-0,54402	-0,67748	0	-0,37106	-39,1622	-17,5874	0,280258	-0,2081	-73,3854	-56,7496	15,779
BTzPhCl6	304	Done	66,77	-4,72942	glide-grid_4LVT	-6,12972	-1,1344	-0,62038	0	-0,13985	-48,2941	-12,2555	0,233296	-0,21536	-81,8117	-60,5496	8,37334
BTzPhOMe6	434	Done	87,13	-4,71354	glide-grid_4LVT	-5,36164	-1,93847	-0,20694	0	-0,28276	-45,0909	-3,55161	0,242032	-0,38822	-68,8152	-48,6425	9,15031
BTzPhOMe2	401	Done	73,94	-4,70972	glide-grid_4LVT	-5,74192	-1,73423	-0,27018	0	-0,29333	-46,2018	-5,99604	0,160269	-0,39495	-67,2058	-52,1978	18,831
BTzPhOMe1	391	Done	53,75	-4,70844	glide-grid_4LVT	-5,73664	-2,10842	-0,16903	0	-0,44386	-45,7223	-5,3615	0,133203	-0,05819	-71,3861	-51,0838	6,11602
BTzPhDMN5	357	Done	58,15	-4,70408	glide-grid_4LVT	-5,75248	-1,94823	-0,24147	0	-0,35408	-51,0656	-4,57226	0,203997	-0,17358	-77,9934	-55,6379	6,60526
BTzPhDMN4	347	Done	71,34	-4,69241	glide-grid_4LVT	-5,25911	-1,80489	-0,38935	0	-0,00198	-42,545	-6,09142	0,185962	-0,20789	-70,3422	-48,6365	6,94685
BTz5	98	Done	117,55	-4,68875	glide-grid_4LVT	-5,62645	-2,10545	-0,48	0	-0,07243	-37,198	-6,84513	0,352856	-0,43475	-65,1633	-44,0431	8,25085
BTzPhDMN7	371	Done	52,67	-4,68713	glide-grid_4LVT	-5,73353	-1,50178	-0,41014	0	-0,14136	-47,5177	-9,29387	0,235707	-0,14599	-77,6851	-56,8116	6,20172
BTzNaf5	171	Done	78,8	-4,68457	glide-grid_4LVT	-7,19877	-3,44296	-0,23085	0	-0,39861	-49,5401	-4,68572	0,193712	-0,1402	-82,8147	-54,2258	2,85706
BTzPh9	273	Done	62,22	-4,67556	glide-grid_4LVT	-7,34816	-2,14434	-0,7411	0	-0,19198	-55,0027	-10,8692	0,332533	-0,22275	-92,2397	-65,8719	8,36328
BTzPh2	213	Done	94,96	-4,67478	glide-grid_4LVT	-5,16248	-1,93124	-0,32	0	-0,10773	-45,1534	-3,62211	0,214534	-0,21705	-62,5619	-48,7755	12,4072
BTzPhOMe5	429	Done	63,26	-4,67476	glide-grid_4LVT	-6,31516	-1,35864	-0,78817	0	-0,02426	-42,1897	-13,3816	0,224435	-0,25179	-78,342	-55,5714	8,23233
BTzPhDMN1	328	Done	39,38	-4,67459	glide-grid_4LVT	-5,93139	-1,34755	-0,34813	0	-0,68137	-43,5703	-8,64011	0,1132	-0,19301	-69,8429	-52,2104	8,25896
BTzPhOMe8	455	Done	61,92	-4,62793	glide-grid_4LVT	-6,00813	-1,62287	-0,48612	0	-0,12946	-47,9093	-9,67472	0,268515	-0,19153	-76,5386	-57,584	11,4532

BTzPh5	241	Done	103,78	-4,62749	glide-grid_4LVT	-7,22279	-3,21563	-0,16	0	-0,22584	-57,0656	-5,71454	0,277558	-0,18842	-87,2838	-62,7802	8,09523
BTzNaf5	172	Done	80,58	-4,62122	glide-grid_4LVT	-7,21652	-2,0416	-0,52571	0	-0,13891	-57,2142	-11,1262	0,192988	-0,17365	-93,4881	-68,3404	15,2299
BOxPhCl6	35	Done	87,7	-4,61983	glide-grid_4LVT	-4,61983	-1,63321	0	0	-0,13764	-48,8162	-3,92604	0,231742	-0,051	-66,0754	-52,7423	6,26407
BTzPhDMN3	345	Done	50,79	-4,61922	glide-grid_4LVT	-7,10702	-2,18748	-0,59699	0	-0,30192	-50,4569	-9,7933	0,164133	-0,19292	-85,3871	-60,2502	8,62709
BTzPhOMe8	453	Done	74,41	-4,59809	glide-grid_4LVT	-5,61219	-1,62118	-0,5572	0	0	-39,9399	-9,89664	0,269328	-0,22164	-68,8535	-49,8365	6,19395
BTzPh2	217	Done	72,08	-4,58997	glide-grid_4LVT	-7,05487	-3,67269	0	0	-0,50156	-59,9908	-0,24269	0,213536	-0,05821	-83,9388	-60,2335	6,99353
BT8	64	Done	65,31	-4,58022	glide-grid_4LVT	-4,58022	-1,72481	-0,16009	0	-0,03363	-44,9199	-3,85615	0,323069	-0,16034	-58,4719	-48,7761	9,75631
BTzPhOMe6	437	Done	88,15	-4,57768	glide-grid_4LVT	-5,95788	-1,59466	-0,44175	0	-0,15734	-56,9	-7,41693	0,240388	-0,04698	-83,7931	-64,3169	11,1027
BTzPhDMN1	325	Done	39,06	-4,57332	glide-grid_4LVT	-5,24022	-2,04858	-0,16	0	-0,41418	-45,9929	-0,99472	0,114621	-0,28323	-62,1409	-46,9876	14,4927
BTzPh7	253	Done	77,98	-4,56575	glide-grid_4LVT	-6,01395	-1,75275	-0,53336	0	-0,29616	-50,9683	-7,37957	0,308669	-0,085	-77,7478	-58,3479	7,85265
BTzNaf7	184	Done	55,73	-4,56255	glide-grid_4LVT	-6,01075	-0,39452	-0,57353	0	-0,00553	-41,9672	-18,7915	0,224677	-0,34476	-83,7957	-60,7587	10,4701
BTz9	132	Done	66,14	-4,55447	glide-grid_4LVT	-6,90927	-1,84968	-0,48	0	-0,9133	-53,5484	-8,96919	0,41828	-0,06177	-87,2945	-62,5176	9,38216
BTzPhDMN8	377	Done	50,57	-4,5468	glide-grid_4LVT	-5,3691	-1,57461	-0,16	0	-0,12847	-51,3996	-6,90219	0,249046	-0,14975	-73,7929	-58,3018	7,11577
BTz2	79	Done	65,52	-4,5305	glide-grid_4LVT	-7,0645	-2,1404	-0,58987	0	-0,74762	-49,7863	-8,5364	0,264611	-0,08146	-80,6559	-58,3227	7,36214
BTzPhOMe8	456	Done	71,32	-4,52322	glide-grid_4LVT	-6,15912	-1,6142	-0,1593	0	-0,78188	-50,7833	-7,63151	0,269328	-0,18918	-78,6778	-58,4149	13,2612

BTzNaf5	166	Done	67,06	-4,5212	glide-grid_4LVT	-5,1328	-2,06222	-0,16	0	0	-38,9843	-5,91918	0,194439	-0,26792	-66,6485	-44,9035	2,83704
BTzPhCl8	317	Done	59,31	-4,51709	glide-grid_4LVT	-7,06339	-3,36268	-0,12302	0	-0,62259	-57,3936	-1,20944	0,262284	-0,16629	-86,1283	-58,603	8,28864
BTz6	109	Done	133,67	-4,51605	glide-grid_4LVT	-6,87375	-2,16954	-0,60998	0	-0,16293	-49,7991	-10,0807	0,372355	-0,3016	-82,3201	-59,8799	8,41069
BTz3	87	Done	78,26	-4,51522	glide-grid_4LVT	-7,03612	-2,16791	-0,38033	0	-1,16411	-47,0646	-7,54129	0,298207	-0,13755	-76,5385	-54,6059	6,70175
BTzPhDMN6	367	Done	68,76	-4,50922	glide-grid_4LVT	-7,01832	-2,04987	-0,45444	0	-0,01609	-50,5734	-12,1758	0,220756	-0,36364	-86,0689	-62,7492	19,5881
BTzPhDMN3	340	Done	51,91	-4,50195	glide-grid_4LVT	-5,25675	-0,5548	-0,64787	0	-0,01913	-38,4943	-13,4918	0,164133	-0,25059	-71,3852	-51,9861	5,63068
BTzPhOMe9	469	Done	71,1	-4,49809	glide-grid_4LVT	-7,12179	-2,70596	-0,2159	0	-0,60709	-56,7799	-6,07833	0,280258	-0,12235	-82,5637	-62,8582	17,2088
BTzNaf6	180	Done	53,54	-4,49786	glide-grid_4LVT	-7,17046	-2,48776	-0,39471	0	-0,34661	-53,3652	-8,76388	0,209716	-0,16826	-88,4454	-62,129	6,39307
BTzPhOMe3	409	Done	76,65	-4,47962	glide-grid_4LVT	-5,49692	-1,89279	-0,14242	0	-0,09167	-46,975	-6,19415	0,18426	-0,27644	-67,7514	-53,1691	13,4937
BTzPh3	222	Done	98,87	-4,46572	glide-grid_4LVT	-5,79882	-1,69988	-0,30693	0	-0,44229	-49,0984	-6,71229	0,237111	-0,12507	-71,4957	-55,8107	14,4368
BTz6	105	Done	119,36	-4,46535	glide-grid_4LVT	-5,40755	-1,93105	-0,55952	0	0	-37,2887	-7,78937	0,373771	-0,25792	-64,599	-45,0781	8,11042
BTzPhCl4	289	Done	79,44	-4,44571	glide-grid_4LVT	-4,84961	-2,13082	-0,19223	0	0	-35,602	-3,64422	0,199256	-0,39909	-58,3867	-39,2462	7,01031
BTzPhDMN5	361	Done	66,07	-4,44399	glide-grid_4LVT	-7,03929	-1,9265	-0,456	0	-0,42757	-54,3417	-10,4832	0,203241	-0,14289	-86,2075	-64,825	15,0998
BTzPhCl2	279	Done	79,09	-4,44111	glide-grid_4LVT	-4,75981	-1,88221	-0,10056	0	-0,20621	-48,8384	-1,12671	0,154136	-0,11404	-66,947	-49,9651	5,40095
BTzPh5	239	Done	76,97	-4,44105	glide-grid_4LVT	-6,88035	-2,97946	-0,26304	0	-0,12944	-46,8659	-7,79969	0,278558	-0,27372	-80,0866	-54,6656	11,9036

BTzPh4	232	Done	63,96	-4,43375	glide-grid_4LVT	-6,89215	-2,81847	-0,28889	0	-0,75734	-49,595	-4,89778	0,25966	-0,0727	-79,8885	-54,4928	8,44894
BTzPhDMN6	364	Done	56,24	-4,42709	glide-grid_4LVT	-5,47349	-1,96176	-0,46066	0	-0,08182	-45,5402	-4,7356	0,220756	-0,20266	-71,5653	-50,2758	6,1062
BTzPhOMe8	454	Done	60,72	-4,42162	glide-grid_4LVT	-5,43572	-2,27732	-0,2804	0	-0,00355	-46,0381	-4,72126	0,269328	-0,13369	-71,0809	-50,7594	7,10398
BTzPh7	257	Done	71,72	-4,41539	glide-grid_4LVT	-7,08799	-2,04388	-0,42891	0	-0,16411	-52,7738	-12,515	0,308669	-0,24382	-91,0961	-65,2887	10,4758
BTzPhOMe8	460	Done	64,96	-4,41223	glide-grid_4LVT	-7,03593	-2,66543	-0,01834	0	-0,76537	-62,9814	-4,34605	0,268515	-0,05433	-90,2387	-67,3275	11,5461
BTz5	100	Done	123,85	-4,40968	glide-grid_4LVT	-6,77468	-2,33404	0	0	-1,04885	-55,5434	-5,03334	0,351423	-0,21104	-82,2933	-60,5767	10,6345
BTzPh3	219	Done	111,19	-4,38509	glide-grid_4LVT	-4,97929	-1,7789	-0,16	0	-0,66725	-41,7327	-1,89112	0,239117	-0,24195	-64,5393	-43,6239	6,26267
BTzPhOMe1	396	Done	45,98	-4,36836	glide-grid_4LVT	-6,20176	-1,75124	-0,16	0	-0,66983	-51,473	-7,01409	0,132423	-0,12736	-74,8711	-58,4871	14,7434
BTzPhOMe3	410	Done	79,39	-4,36417	glide-grid_4LVT	-5,70217	-1,76027	-0,2511	0	-0,00295	-43,6602	-10,1419	0,183449	-0,167	-68,1714	-53,8021	11,0715
BTzPhCl9	322	Done	71,06	-4,36013	glide-grid_4LVT	-5,76043	-0,78651	-0,43889	0	-0,2	-52,6228	-11,835	0,273295	-0,20194	-81,2241	-64,4578	16,2976
BTzPhDMN9	384	Done	77,21	-4,34989	glide-grid_4LVT	-5,17219	-1,4789	-0,25568	0	-0,17649	-54,7135	-3,59728	0,260944	-0,2468	-76,1897	-58,3108	10,0977
BTzNaf6	177	Done	58,02	-4,33154	glide-grid_4LVT	-6,71204	-2,55742	-0,16	0	-0,67111	-47,1482	-5,83142	0,210443	-0,30183	-82,2892	-52,9796	4,71888
BTzPh2	216	Done	79,86	-4,33134	glide-grid_4LVT	-5,55814	-1,1488	-0,16	0	-0,71855	-52,9616	-6,58618	0,212541	-0,10732	-77,3616	-59,5478	8,70964
BTzPhOMe9	467	Done	80,82	-4,32408	glide-grid_4LVT	-6,32598	-1,98759	0	0	-1,00843	-66,7188	-1,67343	0,280258	-0,02327	-89,0848	-68,3922	11,8344
BTzNaf5	169	Done	68,06	-4,3154	glide-grid_4LVT	-5,3793	-0,8856	-0,38641	0	-0,19673	-50,1459	-9,00217	0,192988	-0,24593	-78,9196	-59,148	2,98858

BTzNaf8	194	Done	70,62	-4,31367	glide-grid_4LVT	-6,76437	-2,72689	-0,13238	0	-0,33736	-54,5476	-6,3878	0,238783	-0,12097	-87,3381	-60,9354	9,05445
BTz3	85	Done	97,99	-4,31077	glide-grid_4LVT	-6,69967	-2,11695	-0,16	0	-0,85616	-50,8898	-7,87493	0,298207	-0,13904	-78,0143	-58,7647	13,8455
BTz3	84	Done	98,09	-4,30927	glide-grid_4LVT	-6,69677	-2,10005	-0,456	0	-0,61353	-45,2677	-9,6901	0,298207	-0,10849	-74,42	-54,9578	9,40085
BTz2	76	Done	70,52	-4,30353	glide-grid_4LVT	-5,24713	-1,84745	-0,32	0	-0,71414	-44,7468	-1,79201	0,26605	-0,12546	-62,486	-46,5388	6,71952
BTzNaf1	136	Done	54,35	-4,30294	glide-grid_4LVT	-4,96984	-1,89298	-0,16	0	-0,52975	-43,667	-1,34756	0,104976	-0,10661	-60,7382	-45,0145	4,65791
BTzPhDMN3	346	Done	70,26	-4,29786	glide-grid_4LVT	-6,97706	-2,20782	-0,30421	0	-0,29569	-48,3922	-11,407	0,163391	-0,20207	-85,7525	-59,7992	2,45149
BTzPhCl5	298	Done	84,15	-4,28065	glide-grid_4LVT	-5,69555	-0,921	-0,456	0	-0,13675	-50,5298	-10,9633	0,216522	-0,22734	-80,6484	-61,4931	7,25084
BTzPhOMe4	416	Done	88,29	-4,27929	glide-grid_4LVT	-4,93459	-0,98463	-0,31199	0	-0,20897	-31,8733	-11,7896	0,206365	-0,27326	-58,6809	-43,6629	6,77343
BTzPh8	265	Done	68,19	-4,27094	glide-grid_4LVT	-6,94354	-2,63263	-0,32	0	-0,75642	-46,767	-7,37768	0,321398	-0,11088	-75,3288	-54,1447	11,2659
BTzPh5	235	Done	101,55	-4,26889	glide-grid_4LVT	-4,88049	-1,36275	-0,58011	0	0	-34,1666	-9,0035	0,27956	-0,15833	-59,2537	-43,1701	10,2732
BTzNaf3	156	Done	69,3	-4,25882	glide-grid_4LVT	-6,74662	-2,21778	-0,29837	0	-0,35485	-55,429	-6,71967	0,154052	-0,25027	-89,0655	-62,1487	4,86514
BTzPhOMe7	445	Done	74,09	-4,25667	glide-grid_4LVT	-5,27077	-1,55879	-0,16	0	-0,29775	-48,7036	-6,07724	0,256104	-0,16357	-73,0979	-54,7808	12,0844
BTzPhOMe4	420	Done	79,14	-4,25584	glide-grid_4LVT	-5,88954	-1,99488	-0,27125	0	-0,53758	-43,2144	-6,61238	0,205543	-0,13879	-72,494	-49,8268	8,9916
BOxPhCl4	33	Done	97,21	-4,25559	glide-grid_4LVT	-4,25559	-1,17986	-0,36558	0	0	-43,2533	-3,95554	0,196117	-0,15027	-57,8725	-47,2089	5,21397
BTz6	107	Done	116,65	-4,24919	glide-grid_4LVT	-5,84859	-1,80865	-0,76347	0	0	-31,3561	-11,973	0,375193	-0,28791	-61,8821	-43,3291	14,4784

BTzPh4	233	Done	66,96	-4,24769	glide-grid_4LVT	-6,77109	-2,63243	-0,24653	0	-0,54327	-47,114	-7,04398	0,258658	-0,19522	-77,1174	-54,158	4,05717
BTzPh4	227	Done	83,31	-4,24753	glide-grid_4LVT	-4,81423	-1,60546	-0,3848	0	0	-45,1852	-3,83392	0,260666	-0,25029	-68,0398	-49,0191	5,58962
BTzPhCl8	314	Done	55,28	-4,23868	glide-grid_4LVT	-5,13828	-1,62361	-0,17252	0	-0,22922	-48,3667	-5,59029	0,262284	-0,11834	-72,6541	-53,9569	7,16743
BTzPh8	264	Done	60,78	-4,23003	glide-grid_4LVT	-6,76213	-1,9838	-0,304	0	-0,04344	-51,0471	-12,826	0,321398	-0,27604	-86,6261	-63,8731	8,16563
BTzPhCl1	274	Done	65,08	-4,191	glide-grid_4LVT	-4,695	-1,99449	0	0	-0,56122	-44,4011	0,486713	0,127216	-0,11946	-56,5694	-43,9144	4,87849
BTzPhOMe9	461	Done	72,77	-4,19055	glide-grid_4LVT	-4,83865	-1,80136	-0,30256	0	-0,02708	-52,8773	-1,4132	0,28187	-0,13367	-72,1821	-54,2905	9,17753
BTz8	127	Done	71,14	-4,18948	glide-grid_4LVT	-6,73448	-2,11816	-0,16	0	-1,13817	-58,9185	-4,726	0,405393	-0,06872	-84,13	-63,6445	11,6755
BTzPhOMe1	395	Done	42,04	-4,18265	glide-grid_4LVT	-5,93145	-1,85484	-0,16	0	-0,38854	-48,4336	-6,84982	0,133203	-0,21211	-72,9223	-55,2834	13,2074
BTz8	126	Done	77,36	-4,17794	glide-grid_4LVT	-6,72294	-2,27116	-0,4836	0	-0,62893	-56,6866	-5,94774	0,405393	-0,01815	-83,5958	-62,6343	13,0612
BTzPhDMN1	327	Done	42,99	-4,1518	glide-grid_4LVT	-5,1259	-1,24751	-0,34836	0	-0,09219	-41,4348	-9,22023	0,113909	-0,09698	-64,9508	-50,655	6,16291
BTzPhCl4	294	Done	79,65	-4,1491	glide-grid_4LVT	-6,7017	-2,01042	-0,4801	0	-0,11667	-44,1875	-11,613	0,198458	-0,34165	-80,6008	-55,8005	7,86351
BTzNaf9	197	Done	67,1	-4,14755	glide-grid_4LVT	-4,70825	-1,52803	-0,32907	0	-0,05714	-47,0203	-3,88913	0,251469	-0,11109	-71,1665	-50,9095	4,73949
BTzPhDMN4	352	Done	58,05	-4,13956	glide-grid_4LVT	-6,59796	-2,14236	-0,23834	0	-0,88851	-45,7506	-6,6118	0,185208	-0,23465	-74,2027	-52,3624	3,54046
BTzNaf2	149	Done	62,05	-4,12274	glide-grid_4LVT	-6,59344	-1,87861	-0,456	0	-0,1	-47,0794	-10,938	0,130594	-0,29476	-83,0704	-58,0173	4,91294
BTz4	95	Done	119,04	-4,12083	glide-grid_4LVT	-6,66243	-1,94759	-0,16	0	-1,24846	-53,6853	-5,30276	0,326906	-0,15361	-81,118	-58,9881	6,05497

BTzNaf1	141	Done	50,1	-4,11785	glide-grid_4LVT	-6,46465	-1,72986	-0,51566	0	-0,27661	-49,5691	-9,06646	0,103626	-0,20771	-79,6593	-58,6356	10,9218
BTzPhOMe7	449	Done	68,89	-4,10947	glide-grid_4LVT	-6,11137	-2,44252	-0,32	0	-0,26868	-62,8822	-1,07245	0,255286	-0,03047	-81,7345	-63,9546	15,5783
BTzNaf4	161	Done	60,83	-4,09558	glide-grid_4LVT	-5,53258	-1,81536	-0,08911	0	-0,0688	-59,5093	-4,46291	0,174279	-0,08868	-82,5454	-63,9722	11,3139
BTzNaf6	179	Done	72,79	-4,08588	glide-grid_4LVT	-6,61798	-1,5981	-0,49687	0	-0,39381	-56,1999	-9,33882	0,209716	-0,1281	-89,7446	-65,5387	7,41803
BTz1	67	Done	47,02	-4,06146	glide-grid_4LVT	-4,92686	-1,61834	-0,48	0	0	-27,7415	-8,40174	0,226675	-0,40785	-41,7429	-36,1433	16,9058
BTzNaf3	151	Done	64,84	-4,06094	glide-grid_4LVT	-4,81574	-1,55758	-0,16839	0	0	-51,6115	-2,39177	0,154052	-0,30447	-71,7822	-54,0033	5,88707
BTzPhCl4	290	Done	85,68	-4,05878	glide-grid_4LVT	-4,95808	-1,1005	-0,35192	0	-0,02478	-37,1937	-10,2596	0,198458	-0,28071	-63,5905	-47,4533	5,51428
BTzPhOMe8	452	Done	64,76	-4,03823	glide-grid_4LVT	-4,68633	-1,17453	-0,31709	0	-0,25253	-54,8237	-2,75569	0,270143	-0,05779	-73,9139	-57,5794	8,79302
BTzPhDMN2	334	Done	60,54	-4,02663	glide-grid_4LVT	-4,88453	-0,81906	-0,57372	0	-0,02522	-37,5427	-9,70312	0,14048	-0,2744	-65,8349	-47,2458	2,77474
BTzPh7	252	Done	74,56	-4,02105	glide-grid_4LVT	-5,01185	-1,09216	-0,21472	0	-0,34344	-48,9047	-7,38455	0,309652	-0,11827	-74,4424	-56,2892	5,4639
BTzPhOMe3	415	Done	74,12	-4,01765	glide-grid_4LVT	-6,62015	-2,26588	-0,32	0	-0,2222	-52,8458	-8,26564	0,183449	-0,11338	-82,9607	-61,1115	11,083
BTzPhDMN6	366	Done	69,97	-4,01315	glide-grid_4LVT	-6,44755	-1,61847	-0,59679	0	-0,01993	-52,5998	-10,2203	0,220756	-0,27008	-88,5226	-62,8202	7,10634
BTz1	68	Done	53,96	-3,99337	glide-grid_4LVT	-4,91357	-1,66684	0	0	-0,80339	-49,5486	-0,95201	0,226675	-0,04978	-64,2458	-50,5006	6,32698
BTzPhOMe7	450	Done	63,18	-3,98036	glide-grid_4LVT	-5,98226	-1,71655	-0,24724	0	-0,05039	-45,1365	-11,8935	0,255286	-0,18252	-76,4666	-57,03	7,29059
BTz3	86	Done	100,33	-3,97627	glide-grid_4LVT	-6,48997	-2,10318	-0,16	0	-0,84332	-50,3274	-6,87026	0,298207	-0,13477	-77,4983	-57,1977	7,4003

BTzPhDMN7	373	Done	61,46	-3,97458	glide-grid_4LVT	-6,40898	-1,32843	-0,68852	0	-0,12416	-51,4829	-11,0481	0,235707	-0,27222	-87,0718	-62,531	8,536
BTzNaf3	154	Done	68,54	-3,96691	glide-grid_4LVT	-6,33191	-2,74465	-0,21922	0	-0,01272	-53,3722	-4,55359	0,154052	-0,15773	-84,6547	-57,9258	7,94595
BTzPhOMe4	424	Done	80,66	-3,96264	glide-grid_4LVT	-6,58544	-2,36	0	0	-0,51861	-61,7105	-5,33688	0,204725	-0,02549	-85,5569	-67,0474	17,3737
BTz4	94	Done	126,5	-3,96155	glide-grid_4LVT	-6,49495	-1,76311	-0,3622	0	-1,0785	-45,3606	-7,66855	0,326906	-0,19974	-74,8905	-53,0291	2,78582
BTzPhDMN8	378	Done	63,98	-3,94888	glide-grid_4LVT	-4,99528	-1,39313	-0,16	0	-0,54086	-42,6181	-4,76018	0,249046	-0,30541	-65,8719	-47,3782	4,71985
BTz6	108	Done	121,04	-3,93119	glide-grid_4LVT	-6,28889	-1,77455	-0,20526	0	-0,82796	-51,56	-7,04	0,372355	-0,21948	-77,1515	-58,6	15,9044
BTzPhOMe1	390	Done	48,22	-3,91274	glide-grid_4LVT	-4,59884	-1,45625	-0,16	0	-0,21777	-39,2538	-3,10916	0,133987	-0,46974	-57,3474	-42,363	11,9934
BTz2	77	Done	62,01	-3,91261	glide-grid_4LVT	-5,17051	-1,91774	-0,32	0	-0,52753	-41,9659	-2,76695	0,267495	-0,1594	-60,7377	-44,7328	9,89406
BTzPh6	249	Done	76,24	-3,89349	glide-grid_4LVT	-6,56609	-2,21686	-0,27602	0	-0,60027	-54,652	-6,236	0,294136	-0,09908	-82,375	-60,888	10,1746
BTz6	110	Done	141,44	-3,87854	glide-grid_4LVT	-6,42254	-2,13006	-0,16	0	-1,09517	-50,9289	-4,88768	0,372355	-0,13007	-75,0999	-55,8166	9,52189
BTz4	91	Done	122,75	-3,8775	glide-grid_4LVT	-5,4845	-1,9845	-0,41414	0	-0,09167	-39,585	-6,23241	0,329799	-0,40987	-65,1906	-45,8174	6,44331
BTzPhOMe3	413	Done	81,91	-3,87191	glide-grid_4LVT	-5,83961	-2,21022	-0,24577	0	-0,1885	-50,0558	-5,26369	0,183449	-0,08622	-73,3327	-55,3195	5,52126
BTz9	133	Done	69,65	-3,87028	glide-grid_4LVT	-6,22508	-2,04978	-0,16318	0	-0,88479	-54,8611	-4,27064	0,41828	-0,16195	-79,71	-59,1317	9,57124
BTzPhOMe4	423	Done	71,82	-3,86871	glide-grid_4LVT	-5,86471	-2,3799	-0,14454	0	-0,131	-51,7024	-4,93369	0,204725	-0,08882	-73,988	-56,6361	14,4822
BTzPhOMe2	406	Done	72,81	-3,86784	glide-grid_4LVT	-6,08374	-1,24656	-0,27024	0	-0,72149	-51,6108	-8,31648	0,15947	-0,1769	-76,904	-59,9273	13,5253

BTzPh3	225	Done	105,18	-3,86443	glide-grid_4LVT	-6,35223	-2,08881	-0,50196	0	-0,11365	-44,724	-9,8592	0,238112	-0,17083	-76,8539	-54,5832	7,52677
BTzPh8	259	Done	66,03	-3,86428	glide-grid_4LVT	-4,64438	-0,59005	-0,56774	0	-0,21614	-44,3174	-8,36415	0,322369	-0,12232	-68,6051	-52,6816	6,18143
BTzPhDMN6	368	Done	49,82	-3,83944	glide-grid_4LVT	-6,41964	-1,77213	-0,456	0	-0,1976	-50,1939	-10,1966	0,219998	-0,17472	-80,8661	-60,3905	11,2994
BTzPhCl7	307	Done	63,52	-3,83541	glide-grid_4LVT	-4,23431	-1,71671	-0,32	0	0	-48,5347	0,544865	0,249814	-0,1024	-65,3183	-47,9899	4,39184
BTzPhOMe2	403	Done	75,56	-3,83234	glide-grid_4LVT	-5,53304	-1,98625	-0,19863	0	-0,32577	-51,5474	-3,37021	0,160269	-0,09975	-66,7986	-54,9176	11,3001
BTz3	83	Done	94,32	-3,82904	glide-grid_4LVT	-5,46104	-1,88437	-0,69942	0	-0,19749	-32,3618	-7,8909	0,301106	-0,17914	-61,5291	-40,2527	4,44097
BTz1	71	Done	47,13	-3,81913	glide-grid_4LVT	-6,29203	-1,66608	-0,32	0	-1,11777	-49,7241	-5,90953	0,225258	-0,0408	-72,6547	-55,6336	9,38614
BTzPhOMe6	436	Done	70,8	-3,81505	glide-grid_4LVT	-4,82915	-1,71082	-0,16	0	-0,27877	-52,5631	-0,99506	0,241209	-0,14336	-67,7142	-53,5581	7,68498
BTzPhDMN8	376	Done	60,35	-3,79401	glide-grid_4LVT	-4,41451	-0,34031	-0,71077	0	0	-37,1897	-8,78602	0,249802	-0,43583	-60,1416	-45,9757	9,03975
BA	1	Done	2,83	-3,74334	glide-grid_4LVT	-3,75104	-0,73012	-0,69071	0	-0,3021	-20,642	-6,84178	0,096752	-0,0665	-36,5487	-27,4837	1,04218
BTzPhOMe3	407	Done	84,64	-3,72921	glide-grid_4LVT	-4,40481	-1,22962	-0,16	0	-0,08982	-48,3864	-2,95376	0,185074	-0,24807	-66,2129	-51,3401	14,0005
BTzPhDMN7	374	Done	69,83	-3,72153	glide-grid_4LVT	-6,23063	-1,76957	-0,60805	0	-0,14885	-47,3892	-8,79152	0,235707	-0,25168	-81,8387	-56,1808	5,59905
BTzPh6	246	Done	75,45	-3,68932	glide-grid_4LVT	-6,06982	-2,00937	-0,48	0	-0,17771	-42,9922	-7,8065	0,295128	-0,37728	-72,2566	-50,7987	7,91706
BTz8	123	Done	94,13	-3,65054	glide-grid_4LVT	-5,25094	-1,81308	-0,32363	0	-0,08333	-32,9229	-9,42915	0,408139	-0,37852	-63,4094	-42,352	7,4631
BTzPhDMN1	330	Done	39,51	-3,64559	glide-grid_4LVT	-5,99239	-2,20644	-0,12598	0	-0,12904	-43,7286	-8,21171	0,1132	-0,22595	-68,83	-51,9403	6,29335

BTzPhOMe8	458	Done	49,97	-3,63203	glide-grid_4LVT	-5,63393	-2,18225	-0,16	0	-0,18324	-49,9473	-5,0555	0,268515	-0,12126	-69,1418	-55,0028	8,94724
BTzNaf3	157	Done	67,24	-3,59976	glide-grid_4LVT	-6,27896	-1,52521	-0,16	0	-0,70109	-55,8894	-7,1504	0,153343	-0,17897	-84,7711	-63,0398	6,19012
BTzPhOMe2	404	Done	52,86	-3,597	glide-grid_4LVT	-5,3035	-0,9906	-0,52137	0	0	-37,7377	-11,1495	0,160269	-0,39249	-62,0459	-48,8872	17,3049
BTzPhCl6	301	Done	70,52	-3,5946	glide-grid_4LVT	-3,9935	-1,7716	-0,16	0	0	-39,3176	-0,45904	0,234897	-0,26206	-53,1231	-39,7766	4,70267
BTzNaf1	142	Done	52,59	-3,5933	glide-grid_4LVT	-6,1905	-1,98993	-0,16	0	-0,33902	-50,9172	-7,07942	0,1043	-0,19808	-78,6686	-57,9966	12,2739
BTzNaf2	148	Done	56,88	-3,58563	glide-grid_4LVT	-6,05053	-1,9997	-0,44691	0	0	-43,0864	-9,05631	0,130594	-0,22175	-76,5974	-52,1427	3,18476
BTzPhOMe7	448	Done	67,85	-3,58369	glide-grid_4LVT	-5,21959	-1,46692	-0,3142	0	-0,14523	-40,3085	-9,23907	0,256104	-0,14806	-64,1374	-49,5476	8,47725
BTzPhCl9	323	Done	63,4	-3,58198	glide-grid_4LVT	-6,12828	-1,7054	-0,43128	0	-0,01829	-48,7013	-10,189	0,274081	-0,28398	-82,9977	-58,8903	7,77132
BTzPhDMN9	385	Done	61,65	-3,5787	glide-grid_4LVT	-4,6251	-0,91391	-0,20619	0	-0,50022	-41,8565	-6,67275	0,260944	-0,17199	-65,396	-48,5292	3,54941
BTzPhCl6	306	Done	66,33	-3,56473	glide-grid_4LVT	-6,11103	-2,20259	-0,47332	0	-0,29144	-48,5457	-4,98391	0,234095	-0,2029	-72,9924	-53,5296	12,0378
BTzPhDMN5	359	Done	65,75	-3,56321	glide-grid_4LVT	-6,00251	-1,53604	-0,3904	0	-0,43425	-53,0354	-5,731	0,203997	-0,33439	-80,0348	-58,7664	9,06996
BTzPhDMN2	338	Done	58,04	-3,55913	glide-grid_4LVT	-6,02983	-1,93109	-0,11243	0	-0,30199	-53,2284	-5,87912	0,14048	-0,28152	-84,5355	-59,1075	2,90291
BTzPh1	211	Done	63,97	-3,55631	glide-grid_4LVT	-6,15351	-1,8692	-0,44931	0	-0,05833	-40,0719	-11,0134	0,18549	-0,30657	-65,6088	-51,0852	19,0092
BTzPhCl5	299	Done	84,81	-3,5338	glide-grid_4LVT	-6,0876	-2,05722	-0,40925	0	-0,22526	-49,1376	-6,48102	0,21732	-0,18415	-77,9813	-55,6186	9,71466
BTzPh3	224	Done	98,74	-3,49814	glide-grid_4LVT	-5,97894	-2,32009	-0,13335	0	-0,31495	-53,6066	-3,86794	0,237111	-0,18715	-74,3553	-57,4745	8,60917

BTzPhCl4	293	Done	90,41	-3,49738	glide-grid_4LVT	-6,03928	-2,0407	-0,16	0	-0,75404	-44,3188	-6,58804	0,198458	-0,07885	-73,1603	-50,9069	6,17838
BTzPhDMN9	389	Done	63,51	-3,49689	glide-grid_4LVT	-6,07709	-1,78442	-0,32	0	-0,53375	-51,7598	-6,59678	0,260195	-0,1216	-78,86	-58,3566	9,53143
BTz7	118	Done	101,89	-3,49244	glide-grid_4LVT	-6,03744	-1,98933	-0,3759	0	-0,04167	-47,7491	-9,16658	0,390204	-0,25831	-75,9818	-56,9157	8,69444
BTz9	134	Done	68,41	-3,47968	glide-grid_4LVT	-6,02468	-1,3572	-0,304	0	-0,28997	-49,9294	-11,4272	0,41828	-0,28124	-82,5285	-61,3566	4,8249
BTzPhOMe7	451	Done	59,69	-3,47601	glide-grid_4LVT	-6,09971	-1,653	-0,72477	0	-0,1375	-53,3363	-7,37286	0,255286	-0,06697	-78,1068	-60,7092	12,6307
BTzNaf4	164	Done	64,87	-3,46497	glide-grid_4LVT	-5,98837	-1,91326	-0,48939	0	0	-51,9518	-6,54657	0,174279	-0,18043	-77,1274	-58,4984	4,40219
BTzPh3	223	Done	107,05	-3,44836	glide-grid_4LVT	-5,81336	-2,29049	-0,29546	0	-0,24448	-49,4392	-4,2459	0,238112	-0,1122	-77,3832	-53,6851	4,68025
BTzPhOMe9	466	Done	76,28	-3,44166	glide-grid_4LVT	-5,07756	-1,58232	-0,32	0	-0,25077	-44,6212	-4,56009	0,281063	-0,29046	-67,1202	-49,1813	9,54524
BTzNaf1	143	Done	52	-3,43431	glide-grid_4LVT	-6,06141	-1,30924	-0,32	0	-0,73786	-46,3503	-8,4905	0,103626	-0,20685	-73,1101	-54,8408	8,11609
BTzPhDMN8	381	Done	63,66	-3,4259	glide-grid_4LVT	-5,935	-1,82843	-0,28815	0	-0,07652	-49,5716	-7,74374	0,249046	-0,35081	-78,0517	-57,3153	9,11334
BTzPhDMN3	343	Done	65	-3,41955	glide-grid_4LVT	-5,78455	-1,75594	-0,36326	0	-0,16391	-55,9609	-4,92906	0,164133	-0,12818	-84,0681	-60,8899	4,93835
BTzPhDMN9	387	Done	69,29	-3,40532	glide-grid_4LVT	-5,83972	-2,18892	0	0	-0,75735	-59,0898	-1,08135	0,260944	-0,0377	-79,5243	-60,1712	11,2787
BTzPhDMN1	332	Done	42,43	-3,3942	glide-grid_4LVT	-6,0213	-2,13848	-0,16	0	-0,0788	-39,8122	-10,5592	0,1132	-0,18273	-68,3836	-50,3715	5,29884
BTzPhOMe8	457	Done	71,13	-3,38457	glide-grid_4LVT	-5,02047	-1,49903	-0,20852	0	-0,31214	-50,0949	-4,32128	0,269328	-0,11716	-69,9205	-54,4162	11,5116
BTzNaf1	138	Done	56,98	-3,35058	glide-grid_4LVT	-4,32468	-1,29022	-0,01368	0	-0,29746	-45,6937	-2,84654	0,1043	-0,11595	-61,5547	-48,5403	7,59706

BTzPhDMN6	365	Done	67,88	-3,34672	glide-grid_4LVT	-4,39892	-0,86096	-0,26851	0	-0,27213	-42,7284	-6,33253	0,219998	-0,13102	-62,7054	-49,0609	5,72881
BTzPh7	255	Done	66,39	-3,32892	glide-grid_4LVT	-5,77962	-2,07413	-0,31898	0	-0,17485	-50,2745	-5,49112	0,309652	-0,18393	-73,8212	-55,7656	15,6262
BTzNaf3	155	Done	73,49	-3,32883	glide-grid_4LVT	-5,80963	-1,94063	-0,17567	0	-0,20958	-55,968	-4,35433	0,153343	-0,18555	-77,9788	-60,3223	7,0648
BTzPh4	234	Done	70,33	-3,32761	glide-grid_4LVT	-5,99381	-1,92156	-0,40429	0	-0,10633	-47,2386	-8,54279	0,258658	-0,17693	-78,093	-55,7814	3,01855
BTzPhDMN2	333	Done	52,51	-3,32349	glide-grid_4LVT	-3,81119	-1,42732	-0,16	0	-0,06292	-33,1756	-0,97269	0,141211	-0,49748	-44,8275	-34,1483	5,95595
BTzPhOMe6	438	Done	67,67	-3,32206	glide-grid_4LVT	-4,95796	-1,40214	-0,48351	0	-0,04454	-38,1836	-7,34668	0,241209	-0,25779	-58,7616	-45,5303	13,3736
BTzPhOMe5	433	Done	73,26	-3,30883	glide-grid_4LVT	-5,95083	-1,54809	-0,43681	0	-0,35934	-51,2607	-7,65675	0,223613	-0,11867	-79,2217	-58,9175	7,81036
BTzPh1	205	Done	76,94	-3,30793	glide-grid_4LVT	-3,97483	-1,56182	-0,0693	0	-0,23961	-39,5674	-0,66566	0,186474	-0,21235	-49,2618	-40,2331	9,41251
BTzNaf9	202	Done	53,04	-3,29689	glide-grid_4LVT	-5,74759	-1,31804	-0,52972	0	0	-47,1566	-10,2919	0,250747	-0,24896	-78,4756	-57,4485	8,94975
BTzPhDMN8	382	Done	62,97	-3,28352	glide-grid_4LVT	-5,86372	-1,50028	-0,44616	0	-0,40111	-55,0917	-5,85039	0,248293	-0,13232	-77,7626	-60,9421	13,864
BTz5	103	Done	109,28	-3,27054	glide-grid_4LVT	-5,82374	-1,70627	-0,456	0	-0,15901	-44,8857	-9,20126	0,351423	-0,22941	-72,4271	-54,0869	8,50461
BTzPh9	266	Done	55,38	-3,26822	glide-grid_4LVT	-3,82892	-1,19553	-0,02987	0	-0,2767	-41,1876	-1,93716	0,334451	-0,31133	-56,9485	-43,1247	4,67267
BTzNaf1	140	Done	49,59	-3,26295	glide-grid_4LVT	-5,56765	-1,03946	-0,40318	0	-0,09081	-49,5942	-9,23306	0,1043	-0,27383	-79,0121	-58,8273	8,47842
BTzPhCl9	324	Done	54,1	-3,23395	glide-grid_4LVT	-5,78025	-1,30509	-0,44677	0	-0,38189	-53,0842	-7,91545	0,274081	-0,07905	-82,4689	-60,9996	5,88252
BTzPh2	218	Done	89,23	-3,2211	glide-grid_4LVT	-5,6918	-2,16866	-0,26311	0	-0,17029	-44,632	-5,79571	0,213536	-0,20233	-70,1687	-50,4277	6,66018

BTzNaf2	144	Done	62,14	-3,20315	glide-grid_4LVT	-3,69085	-0,89439	-0,24268	0	-0,02568	-33,5531	-5,48228	0,131291	-0,15939	-49,2451	-39,0354	2,51261
BTzPhDMN9	388	Done	60,74	-3,18813	glide-grid_4LVT	-5,69723	-1,30645	-0,35857	0	-0,19648	-50,7624	-8,98617	0,260944	-0,21063	-79,9457	-59,7486	11,366
BTzPhOMe9	465	Done	70,27	-3,18047	glide-grid_4LVT	-4,81637	-1,92991	-0,05859	0	-0,11579	-47,4069	-3,45551	0,281063	-0,10447	-64,1065	-50,8624	9,33174
BTz1	70	Done	52,52	-3,17853	glide-grid_4LVT	-5,53513	-2,03569	-0,2811	0	-0,65677	-42,3027	-4,23269	0,225258	-0,03679	-61,1085	-46,5354	6,92503
BTzPhOMe6	442	Done	69,62	-3,16543	glide-grid_4LVT	-5,78913	-1,77784	-0,16	0	-0,28355	-58,498	-5,15432	0,240388	-0,11008	-80,1114	-63,6523	17,1031
BTz5	99	Done	125,5	-3,16278	glide-grid_4LVT	-4,74648	-2,01021	-0,4178	0	0	-31,0972	-5,70004	0,354295	-0,2629	-54,5135	-36,7973	2,81236
BTz1	72	Done	54,56	-3,12476	glide-grid_4LVT	-5,71056	-1,62502	-0,16	0	-0,76557	-43,958	-6,85308	0,225258	-0,15936	-66,5028	-50,8111	6,66556
BTz8	125	Done	103,47	-3,11531	glide-grid_4LVT	-5,47011	-1,43453	-0,608	0	-0,34969	-42,532	-8,80309	0,405393	-0,03622	-67,9476	-51,3351	5,79365
BTz7	115	Done	110,32	-3,11494	glide-grid_4LVT	-4,71534	-1,85726	-0,30538	0	-0,1	-34,8778	-5,84973	0,393	-0,22435	-57,0941	-40,7275	7,74515
BTzPhDMN3	344	Done	51,58	-3,07666	glide-grid_4LVT	-5,55746	-1,47257	-0,30548	0	-0,50167	-50,0174	-5,1413	0,163391	-0,16907	-71,0134	-55,1587	5,99662
BTzPhOMe8	459	Done	55,63	-3,05055	glide-grid_4LVT	-5,05245	-1,27546	-0,16	0	-0,40722	-52,2201	-5,01044	0,268515	-0,1157	-73,4447	-57,2306	6,61343
BTzPh7	254	Done	76,44	-3,00585	glide-grid_4LVT	-5,38635	-1,35531	-0,32	0	-0,41828	-52,9401	-5,54375	0,309652	-0,12385	-76,8916	-58,4838	8,93919
BTz7	117	Done	98,91	-2,9903	glide-grid_4LVT	-5,3452	-1,63982	-0,16	0	-0,25635	-47,5179	-6,12114	0,390204	-0,38516	-68,5765	-53,6391	11,9917
BTzPh5	240	Done	109,21	-2,97152	glide-grid_4LVT	-5,48572	-1,52034	-0,3308	0	-0,12393	-49,9568	-7,41936	0,278558	-0,17846	-75,5672	-57,3761	8,38653
BTzPhDMN4	351	Done	50,06	-2,95327	glide-grid_4LVT	-5,33057	-1,4172	-0,38223	0	-0,19004	-46,3466	-6,44757	0,185208	-0,24184	-72,7042	-52,7941	6,4182

BTzNaf9	201	Done	65,64	-2,93428	glide-grid_4LVT	-5,31478	-1,29469	-0,21725	0	-0,19266	-51,6876	-7,63668	0,250747	-0,13105	-72,8229	-59,3243	21,3356
BTzPhDMN5	360	Done	58,83	-2,93413	glide-grid_4LVT	-5,44833	-1,74658	-0,29912	0	-0,4919	-56,0439	-1,41758	0,203997	-0,09988	-79,0711	-57,4615	5,96678
BTz2	78	Done	58,74	-2,90719	glide-grid_4LVT	-5,41499	-1,89664	0	0	-0,73232	-51,5904	-2,88905	0,264611	-0,03776	-69,2712	-54,4794	5,78815
BTzPhCl7	312	Done	64,15	-2,85586	glide-grid_4LVT	-5,40216	-1,19774	-0,32	0	-0,31129	-52,8196	-7,41944	0,249015	-0,06825	-81,0922	-60,2391	6,86065
BTzPh1	209	Done	66,42	-2,85232	glide-grid_4LVT	-5,15702	-1,89773	-0,16	0	-0,51183	-42,5741	-2,53516	0,18549	-0,26397	-63,2154	-45,1093	5,29144
BTzPhDMN2	337	Done	46,38	-2,82816	glide-grid_4LVT	-5,29306	-2,09354	0	0	-0,30514	-56,381	-0,76027	0,14048	-0,10177	-77,3875	-57,1413	8,45706
BTzPhOMe1	394	Done	43,22	-2,82551	glide-grid_4LVT	-4,36861	-1,75035	0	0	-0,08419	-42,8366	-3,26304	0,133203	-0,03599	-55,2021	-46,0996	9,82249
BTzPhOMe9	468	Done	61,22	-2,77396	glide-grid_4LVT	-4,77586	-1,17487	-0,44187	0	0	-42,049	-7,59749	0,280258	-0,19731	-62,6273	-49,6465	9,52482
BTzPhCl3	288	Done	90,84	-2,77341	glide-grid_4LVT	-5,32741	-2,22471	-0,0316	0	-0,19554	-53,4762	-1,40947	0,17724	-0,16757	-73,5325	-54,8857	6,53019
BTzPhDMN4	353	Done	57,61	-2,77261	glide-grid_4LVT	-5,29601	-1,16287	-0,16	0	-0,46827	-47,0071	-8,10507	0,184456	-0,12321	-71,3192	-55,1122	7,2606
BTzPh9	270	Done	76,64	-2,75598	glide-grid_4LVT	-5,13648	-1,71487	-0,15317	0	-0,35753	-54,2209	-2,96441	0,333491	-0,08869	-77,1527	-57,1854	7,32979
BTzPhCl5	300	Done	87,36	-2,75499	glide-grid_4LVT	-5,30879	-1,66931	-0,16	0	-0,1189	-44,352	-7,30922	0,21732	-0,26391	-72,8603	-51,6612	2,79164
BTzPhOMe4	422	Done	69,56	-2,75293	glide-grid_4LVT	-4,74693	-1,34838	-0,16976	0	-0,17154	-47,6158	-5,09667	0,204725	-0,11668	-66,248	-52,7125	9,11211
BTzPhCl7	309	Done	74,18	-2,74848	glide-grid_4LVT	-3,89418	-0,7586	-0,29404	0	0	-48,0848	-3,27421	0,249015	-0,19519	-61,9189	-51,359	7,71722
BTzPhCl7	311	Done	65,58	-2,52801	glide-grid_4LVT	-5,07431	-1,31443	-0,44436	0	-0,38088	-45,9426	-5,14045	0,249015	-0,11545	-68,8546	-51,0831	7,09954

BTzPh1	210	Done	63,8	-2,50415	glide-grid_4LVT	-4,85095	-1,31606	-0,15167	0	-0,55254	-45,0806	-4,19053	0,184509	-0,13259	-62,5832	-49,2711	6,73067
BTz1	69	Done	49,78	-2,44425	glide-grid_4LVT	-4,15405	-1,08369	-0,32	0	-0,21754	-25,5992	-8,69781	0,228099	-0,17628	-48,5667	-34,2971	6,06571
BTzPhOMe1	398	Done	45,46	-2,33947	glide-grid_4LVT	-4,89347	-1,44729	-0,18208	0	-0,07436	-45,9487	-6,49327	0,132423	-0,05074	-62,518	-52,4419	10,5887
BTzPh8	263	Done	58,22	-2,28117	glide-grid_4LVT	-4,73187	-0,87201	-0,58023	0	-0,25599	-47,0728	-5,63822	0,322369	-0,14664	-70,549	-52,7111	5,29916
BTz1	73	Done	49,65	-2,22616	glide-grid_4LVT	-4,86116	-1,76773	-0,05585	0	-0,69186	-45,8903	-1,60201	0,225258	-0,03616	-54,1109	-47,4923	15,4223
BTzPhDMN1	329	Done	34,58	-2,18015	glide-grid_4LVT	-4,48485	-0,73637	-0,56401	0	0	-32,0162	-10,1388	0,113909	-0,17675	-48,9398	-42,155	13,7797
BTzPhDMN8	380	Done	67,8	-2,16865	glide-grid_4LVT	-4,60305	-1,2267	-0,27227	0	-0,09772	-35,2235	-7,82184	0,249046	-0,32095	-56,8024	-43,0453	8,25907
BTzNaf6	178	Done	52,03	-1,93403	glide-grid_4LVT	-4,38473	-0,55348	-0,36829	0	-0,07515	-44,2375	-8,32837	0,210443	-0,13713	-67,9362	-52,5658	7,29309
BTzPh1	212	Done	66,91	-1,86887	glide-grid_4LVT	-4,49597	-1,31011	-0,31092	0	-0,0838	-45,4489	-4,1566	0,184509	-0,07972	-63,7719	-49,6055	3,29794
BA	2	Done	2,37	-1,56454	glide-grid_4LVT	-4,14454	-1,61047	0	0	-0,37856	-29,448	-5,57063	0,192727	-0,04024	-39,5431	-35,0187	10,2

Table S7. Glide docking scores of docked ligands against Mcl-1 (5LOF)

title	i_i_glide_lignum	docking_status	r_glide_cpu_time	r_i_docking_score	s_i_glide_gridfile	r_i_glide_gscore	r_i_glide_lipo	r_i_glide_hbond	r_i_glide_metal	r_i_glide_rewards	r_i_glide_evdw	r_i_glide_ecoul	r_i_glide_erotb	r_i_glide_esite	r_i_glide_emodel	r_i_glide_energy	r_i_glide_einternal
5LOF - prepared_ligand	1	Done	44,15	-11,4003	glide-grid_5LOF_2	-11,5365	-4,95902	-0,4	0	-2,06214	-62,4717	-7,77255	0,235742	-0,06163	-121,315	-70,2442	10,1576
5LOF - prepare	2	Done	47,19	-9,6516	glide-grid_5	-10,77	-4,57	-0,4	0	-1,728	-59,89	-8,339	0,235742	-0,06	-119,9	-68,23	6,40846

d_ligand					LOF_2	58	554			9	4	91		141	09	4	
5LOF - prepared_ligand	3	Done	48,9	-9,30408	glide-grid_5LOF_2	-11,0178	-4,53763	-0,4	0	-2,13111	-60,3853	-7,35584	0,236928	-0,06332	-122,106	-67,7412	6,77478
BTzPhCl4	290	Done	33,53	-7,88369	glide-grid_5LOF_2	-8,78299	-3,27967	-0,78496	0	-0,33833	-36,9745	-15,4641	0,198458	-0,41014	-83,1935	-52,4386	11,7005
BTzPhDMN1	325	Done	15,34	-7,56464	glide-grid_5LOF_2	-8,23154	-3,40576	-0,47542	0	-0,5729	-36,9908	-12,3803	0,114621	-0,18549	-81,7	-49,3711	13,7547
BTzNaf8	191	Done	54,07	-7,35698	glide-grid_5LOF_2	-8,34778	-3,41162	-0,16	0	-0,30312	-58,7683	-9,52377	0,238783	-0,34484	-106,503	-68,2921	12,2518
BTzNaf7	183	Done	42,93	-7,35267	glide-grid_5LOF_2	-8,34347	-3,36819	-0,39929	0	-0,11667	-56,5212	-10,9151	0,225404	-0,22141	-102,232	-67,4363	12,1841
BTzNaf9	198	Done	51,83	-7,15851	glide-grid_5LOF_2	-7,93861	-3,17033	-0,35508	0	-0,24425	-57,3667	-8,79922	0,250747	-0,23148	-101,378	-66,1659	12,1668
BOxPhCl7	36	Done	46,9	-7,13098	glide-grid_5LOF_2	-7,13098	-3,31617	-0,24694	0	-0,28546	-55,8138	-4,14132	0,246669	-0,11719	-83,0657	-59,9551	10,7758
BOxPhCl3	32	Done	28,47	-7,12921	glide-grid_5LOF_2	-7,12921	-3,18641	-0,61838	0	-0,30428	-50,9287	-3,77374	0,174922	-0,08257	-76,69	-54,7025	5,91613
BTzNaf7	182	Done	38,55	-7,11829	glide-grid_5LOF_2	-7,89839	-3,06616	-0,70544	0	-0,57577	-52,031	-7,08112	0,225404	-0,1127	-90,2667	-59,1121	8,50309
BTzNaf2	144	Done	22,62	-7,05461	glide-grid_5LOF_2	-7,54231	-3,57984	-0,16486	0	-0,16845	-44,8625	-8,12232	0,131291	-0,29898	-87,0705	-52,9849	3,86829
BOxNaf6	17	Done	32,69	-7,0372	glide-grid_5	-7,037	-3,34	-0,064	0	-0,157	-60,39	-3,896	0,208302	-0,07	-86,63	-64,28	9,52607

				1	LOF_2	21	302	22		26		03		71	75	61	
BTzPhCl5	296	Done	39,16	-7,02395	glide-grid_5LOF_2	-7,92765	-2,74464	-0,29448	0	-1,07216	-52,9036	-7,65869	0,21732	-0,2397	-94,5309	-60,5623	3,72256
BTzNaf5	166	Done	38,25	-7,02205	glide-grid_5LOF_2	-7,63365	-2,8128	-0,56064	0	-0,05483	-49,1153	-11,5694	0,194439	-0,20865	-94,8373	-60,6847	11,0646
BOxPhOMe9	56	Done	69,13	-7,00463	glide-grid_5LOF_2	-7,00463	-3,3047	-0,3415	0	-0,15352	-58,4413	-3,33087	0,278691	-0,0619	-81,4133	-61,7722	18,2217
BOxPh4	24	Done	24,59	-6,96391	glide-grid_5LOF_2	-6,96391	-3,75043	0	0	-0,54143	-50,5104	-1,59859	0,256709	-0,16345	-73,5656	-52,109	5,07685
BOxPh5	25	Done	34,03	-6,944	glide-grid_5LOF_2	-6,944	-3,169	-0,32	0	-0,19167	-55,0019	-4,45387	0,275615	-0,12077	-82,8998	-59,4558	6,62314
BTzPhCl3	284	Done	33,13	-6,90205	glide-grid_5LOF_2	-7,79705	-3,17971	-0,19686	0	-1,29893	-58,261	-1,74601	0,17724	-0,12384	-91,132	-60,007	3,444
BOxNaf7	18	Done	33,37	-6,79123	glide-grid_5LOF_2	-6,79123	-2,96309	-0,16	0	0	-54,6359	-7,16752	0,223264	-0,08448	-85,4925	-61,8034	3,82826
BOxPhCl6	35	Done	41,33	-6,76459	glide-grid_5LOF_2	-6,76459	-3,04924	-0,46452	0	-0,30478	-49,9543	-3,86954	0,231742	-0,09965	-77,0316	-53,8239	3,73723
BTzPhOMe4	416	Done	40,33	-6,76381	glide-grid_5LOF_2	-7,41911	-2,76255	-0,4296	0	-0,66787	-45,5643	-8,80944	0,206365	-0,16583	-83,0908	-54,3737	13,2799
BTzNaf3	150	Done	26,37	-6,7489	glide-grid_5LOF_2	-7,3431	-2,6298	-0,47665	0	-0,24743	-32,9553	-13,6356	0,154763	-0,45088	-77,4915	-46,5909	7,9277
BTzPhCl4	289	Done	32,8	-6,7218	glide-grid_5	-7,125	-3,43	-0,080	0	-0,029	-45,15	-6,948	0,199256	-0,48	-82,94	-52,10	7,94253

				9	LOF_2	79	418	14		89	96	57		058	91	81	
BOxNaf5	16	Done	36,62	-6,71564	glide-grid_5 LOF_2	-6,71564	-3,27736	0	0	-0,31911	-54,7318	-2,48307	0,19158	-0,20169	-80,3514	-57,2149	4,94089
BOxPhCl8	37	Done	51,33	-6,7124	glide-grid_5 LOF_2	-6,7124	-3,2993	-0,30953	0	-0,31219	-52,3871	-2,49163	0,259952	-0,05823	-72,3083	-54,8787	13,2445
BOxNaf1	12	Done	18,8	-6,67971	glide-grid_5 LOF_2	-6,67971	-2,94004	0	0	-0,29858	-53,3716	-4,92814	0,102315	-0,1356	-75,5436	-58,2997	16,1723
BOxPhDMN2	40	Done	27,22	-6,67499	glide-grid_5 LOF_2	-6,67499	-2,64792	-0,23801	0	-0,37432	-52,3823	-4,94896	0,138334	-0,19162	-76,1363	-57,3313	11,759
BTzPhOMe5	425	Done	48,68	-6,65999	glide-grid_5 LOF_2	-7,29879	-3,06869	-0,16	0	-0,13285	-55,6583	-7,92199	0,225259	-0,1913	-93,8078	-63,5802	15,171
BTzPhCl8	315	Done	48,43	-6,65473	glide-grid_5 LOF_2	-7,80043	-4,03118	-0,02201	0	-0,168	-61,5343	-3,54709	0,262284	-0,23274	-93,7394	-65,0814	17,0425
BTzNaf6	173	Done	33,38	-6,63425	glide-grid_5 LOF_2	-7,19495	-3,77613	-0,16	0	-0,24874	-47,7528	-4,04285	0,211172	-0,22718	-82,4751	-51,7956	4,26776
BTzPhCl3	285	Done	31,22	-6,62533	glide-grid_5 LOF_2	-7,74693	-2,85387	-0,23895	0	-1,08663	-61,3502	-3,69483	0,17724	-0,12298	-95,1259	-65,045	7,67179
BTzPhOMe6	439	Done	53,99	-6,62259	glide-grid_5 LOF_2	-8,25849	-3,32821	-0,16	0	-0,9232	-61,1444	-4,97318	0,241209	-0,28509	-98,7765	-66,1175	13,7371
BOxNaf2	13	Done	21,75	-6,59152	glide-grid_5 LOF_2	-6,59152	-3,18906	0	0	-0,45	-50,5062	-2,89717	0,12855	-0,12112	-68,5557	-53,4033	15,4384
BOxPhCl4	33	Done	30,26	-6,56	glide-grid_5	-6,56	-3,17	-0,220	0	-0,251	-50,95	-3,146	0,196117	-0,09	-73,51	-54,10	6,40753

					LOF_2		285	48		86	74	13		114	34	35	
BTzPh5	236	Done	30,58	-6,55737	glide-grid_5 LOF_2	-7,38107	-2,94429	-0,70802	0	-0,09	-43,7644	-9,97594	0,278558	-0,23271	-80,9996	-53,7404	6,33939
BTzNaf8	189	Done	55,01	-6,54919	glide-grid_5 LOF_2	-7,10989	-2,52626	-0,53693	0	-0,12571	-34,8064	-14,3503	0,23951	-0,26762	-80,1705	-49,1567	4,32282
BTzNaf4	159	Done	26,89	-6,54238	glide-grid_5 LOF_2	-7,32338	-3,32782	-0,16	0	-0,08742	-52,6694	-5,66102	0,174997	-0,44052	-87,8167	-58,3304	5,83605
BT8	64	Done	45,81	-6,52099	glide-grid_5 LOF_2	-6,52099	-2,57644	0	0	-0,95684	-59,9812	-1,00798	0,323069	-0,16052	-77,2347	-60,9892	17,6874
BOxPh9	29	Done	56,91	-6,51439	glide-grid_5 LOF_2	-6,51439	-3,08493	-0,16	0	-0,15817	-58,5257	-2,90579	0,33067	-0,07981	-78,7494	-61,4315	18,615
BOxPhDMN4	42	Done	38,69	-6,49373	glide-grid_5 LOF_2	-6,49373	-3,05216	0	0	-0,60804	-55,9234	-1,34753	0,182995	-0,01823	-71,3736	-57,271	18,1288
BTzPhDMN4	348	Done	28,79	-6,47737	glide-grid_5 LOF_2	-7,25837	-2,70173	-0,48	0	-0,5706	-45,3682	-7,45335	0,185208	-0,30483	-81,8847	-52,8215	3,65693
BOxPhOMe5	52	Done	44,51	-6,47411	glide-grid_5 LOF_2	-6,47411	-3,12761	-0,16	0	-0,13217	-53,8062	-3,66873	0,222015	-0,03573	-77,48	-57,475	10,6386
BTzPhCl5	297	Done	30,43	-6,47168	glide-grid_5 LOF_2	-7,62228	-2,96051	-0,43885	0	-0,83319	-57,1789	-4,65564	0,21732	-0,04975	-85,7978	-61,8346	13,5495
BOxPh1	21	Done	18,73	-6,46428	glide-grid_5 LOF_2	-6,46428	-3,24541	-0,19469	0	-0,17367	-46,2984	-4,5091	0,182605	-0,04182	-68,6762	-50,8075	5,95476
BTzPh3	220	Done	34,75	-6,462	glide-grid_5	-7,216	-2,42	-0,775	0	-0,222	-32,02	-13,89	0,238112	-0,34	-70,50	-45,91	9,89656

					LOF_2	8	331	07		32	16	5		889	34	66	
BOxNaf4	15	Done	29,83	-6,419	glide-grid_5 LOF_2	-6,419	-3,2617	-0,07528	0	-0,325	-47,5846	-3,31976	0,172882	-0,05271	-70,7908	-50,9044	4,01705
BTzPhDMN5	355	Done	37,61	-6,4113	glide-grid_5 LOF_2	-7,02229	-2,43523	-0,33293	0	-0,45	-42,6739	-11,1021	0,204756	-0,2105	-80,7603	-53,776	8,41974
BOxNaf8	19	Done	51,48	-6,40552	glide-grid_5 LOF_2	-6,40552	-2,88087	-0,13573	0	-0,05	-60,8871	-1,90847	0,23665	-0,24495	-86,3727	-62,7956	6,00158
BTzNaf9	197	Done	56,37	-6,39537	glide-grid_5 LOF_2	-6,95607	-2,60892	-0,52567	0	-0,1108	-45,6641	-8,79618	0,251469	-0,35951	-83,5066	-54,4603	8,65924
BTzPhDMN3	342	Done	27,95	-6,38718	glide-grid_5 LOF_2	-7,72028	-2,51403	-0,91012	0	-0,30911	-39,5965	-13,6082	0,163391	-0,12936	-72,5867	-53,2047	12,5589
BTz9	129	Done	45,69	-6,38692	glide-grid_5 LOF_2	-7,33022	-3,27541	-0,27546	0	-1,02724	-49,8896	-2,98574	0,419624	-0,22939	-78,3392	-52,8753	10,3385
BTz6	106	Done	38,35	-6,3867	glide-grid_5 LOF_2	-7,3289	-2,79547	-0,9103	0	-0,07485	-43,5774	-10,0708	0,373771	-0,23257	-75,3527	-53,6482	16,1571
BOxPh6	26	Done	34,6	-6,38256	glide-grid_5 LOF_2	-6,38256	-2,77443	-0,40193	0	-0,23806	-53,0275	-3,2996	0,292205	-0,11403	-76,5112	-56,3271	10,7112
BOxNaf3	14	Done	29,93	-6,37934	glide-grid_5 LOF_2	-6,37934	-2,86808	-0,17696	0	-0,23732	-48,6717	-4,45048	0,151965	-0,14778	-73,2761	-53,1222	4,29262
BOxPhCl1	30	Done	19,72	-6,3693	glide-grid_5 LOF_2	-6,3693	-3,03377	-0,17426	0	-0,12469	-46,7225	-5,17234	0,124234	-0,04884	-67,8116	-51,8949	10,6118
BTzPhCl5	298	Done	32,02	-6,3670	glide-grid_5	-7,781	-3,05	-0,32	0	-0,949	-57,72	-4,383	0,216522	-0,12	-88,47	-62,10	9,97951

				1	LOF_2	91	518			72	61	81		966	26	99	
BOxPhDMN8	46	Done	49,48	-6,3288	glide-grid_5LOF_2	-6,3288	-1,94583	0	0	-0,54398	-58,3328	-6,05727	0,246826	-0,26059	-84,1915	-64,39	11,1168
BTzPhDMN3	340	Done	27,67	-6,32713	glide-grid_5LOF_2	-7,08193	-1,92138	-0,92395	0	-0,10067	-34,0499	-15,5951	0,164133	-0,25832	-74,0119	-49,645	7,52385
BTzPhOMe7	443	Done	57,91	-6,3186	glide-grid_5LOF_2	-6,9667	-2,96722	0	0	-1,03744	-50,7386	-2,86783	0,256924	-0,25185	-79,195	-53,6064	16,9352
BTzPh6	243	Done	35,44	-6,29298	glide-grid_5LOF_2	-7,07308	-2,54582	-0,16	0	-1,07339	-52,8606	-5,57365	0,295128	-0,10992	-86,3095	-58,4342	5,13432
BTz4	88	Done	36,79	-6,28986	glide-grid_5LOF_2	-6,74986	-2,74002	-0,05203	0	-1,51613	-48,8597	-1,95481	0,326906	-0,03238	-65,6868	-50,8145	9,25881
BTz5	96	Done	32,1	-6,28559	glide-grid_5LOF_2	-6,75149	-3,48318	-0,14977	0	-0,54703	-46,4549	-3,20008	0,351423	-0,12018	-66,1942	-49,6549	10,3063
BTzPhCl7	310	Done	47,5	-6,27008	glide-grid_5LOF_2	-7,67038	-3,35084	-0,27761	0	-0,72835	-62,3286	-2,47338	0,248219	-0,07437	-93,5291	-64,802	4,0587
BTzNaf3	151	Done	31,37	-6,26075	glide-grid_5LOF_2	-7,01555	-2,77047	-0,29094	0	-0,36441	-57,2032	-5,16144	0,154052	-0,10942	-89,6487	-62,3647	10,3239
BOxPhCl9	38	Done	50,08	-6,25428	glide-grid_5LOF_2	-6,25428	-2,75461	0	0	-0,04374	-52,4656	-6,15044	0,271767	-0,18185	-74,2558	-58,6161	15,5242
BTzPh8	260	Done	50,91	-6,24972	glide-grid_5LOF_2	-7,24052	-3,33532	0	0	-0,89368	-59,6596	-0,82965	0,322369	-0,22647	-87,8233	-60,4892	7,67462
BTzPhCl9	322	Done	58,75	-6,2478	glide-grid_5	-7,648	-3,10	-0,298	0	-1,240	-62,27	-0,701	0,273295	-0,06	-88,15	-62,98	9,37391

				5	LOF_2	15	008	28		83	85	78		306	98	03	
BOxPh3	23	Done	30,04	-6,24581	glide-grid_5LOF_2	-6,24581	-2,77047	-0,54657	0	-0,22677	-47,6077	-3,26539	0,235165	-0,06698	-70,0414	-50,8731	5,67078
BOxPhOMe6	53	Done	51,69	-6,24398	glide-grid_5LOF_2	-6,24398	-2,96113	-0,2249	0	-0,14579	-53,3695	-2,66329	0,23879	-0,08299	-75,2905	-56,0328	9,44697
BOx4	6	Done	38,46	-6,22311	glide-grid_5LOF_2	-6,22311	-2,69739	-0,48	0	-0,09654	-41,9559	-7,52847	0,324101	-0,04622	-64,9592	-49,4844	7,74052
BTzPhCl1	276	Done	15,37	-6,21014	glide-grid_5LOF_2	-7,19174	-2,46291	-0,39124	0	-1,24121	-42,4419	-6,88722	0,126457	-0,06765	-73,1347	-49,3291	6,48884
BOxPh8	28	Done	43,83	-6,20224	glide-grid_5LOF_2	-6,20224	-2,68888	-0,35799	0	-0,225	-55,0568	-2,57324	0,319509	-0,11105	-77,2933	-57,6301	9,25571
BTzNaf9	200	Done	58,4	-6,20085	glide-grid_5LOF_2	-7,64905	-3,5107	-0,16	0	-0,98593	-56,6231	-2,5722	0,250027	-0,02546	-83,6414	-59,1953	7,68114
BTzPhCl6	301	Done	40,36	-6,18505	glide-grid_5LOF_2	-6,58395	-2,13286	-0,41203	0	-0,10273	-43,8829	-11,9992	0,234897	-0,1772	-80,7414	-55,8821	11,1362
BOx9	11	Done	45,95	-6,18147	glide-grid_5LOF_2	-6,18147	-3,05889	0	0	-0,43217	-53,2629	-2,68245	0,415668	-0,04056	-73,3431	-55,9454	11,0907
BT1	57	Done	14,26	-6,16867	glide-grid_5LOF_2	-6,16867	-3,1316	-0,16	0	-0,18244	-42,4764	-4,49667	0,143227	-0,03954	-60,1168	-46,973	9,54812
BTzPh5	235	Done	31,5	-6,16744	glide-grid_5LOF_2	-6,77904	-2,47289	-0,37591	0	-0,68587	-40,2575	-8,86201	0,27956	-0,18176	-75,4212	-49,1195	9,62407
BTzNaf1	137	Done	16,73	-6,1649	glide-grid_5	-6,908	-2,84	-0,339	0	-0,106	-48,55	-6,139	0,1043	-0,37	-81,39	-54,69	7,77415

				4	LOF_2	54	242	15		93	42	16		575	9	34	
BTzPhCl3	286	Done	27,27	-6,13932	glide-grid_5 LOF_2	-7,49042	-2,35899	-0,46068	0	-1,19585	-48,1943	-7,54397	0,176453	-0,11005	-78,477	-55,7382	8,13836
BOxPhCl5	34	Done	37,11	-6,13189	glide-grid_5 LOF_2	-6,13189	-2,92956	-0,15783	0	-0,29911	-53,313	-1,51558	0,214968	-0,06738	-77,0795	-54,8285	0,988714
BTzPhCl6	303	Done	32,25	-6,13102	glide-grid_5 LOF_2	-7,27672	-3,11288	-0,25475	0	-0,66246	-45,756	-6,68539	0,234095	-0,19011	-77,4233	-52,4414	8,95314
BTz4	89	Done	42,24	-6,12168	glide-grid_5 LOF_2	-7,06028	-3,3153	-0,38541	0	-0,64099	-38,8357	-6,7649	0,328349	-0,09041	-65,5309	-45,6006	7,3592
BTzPh3	219	Done	40	-6,09606	glide-grid_5 LOF_2	-6,69026	-2,27762	-0,48336	0	-0,70597	-36,4026	-9,6516	0,239117	-0,19455	-73,3402	-46,0542	4,17756
BTz7	112	Done	32,69	-6,0912	glide-grid_5 LOF_2	-6,55	-1,9725	-0,32	0	-1,48776	-50,1017	-4,22358	0,390204	-0,02132	-72,4854	-54,3253	5,02671
BTzNaf5	167	Done	39,5	-6,0852	glide-grid_5 LOF_2	-6,9089	-3,50333	-0,26963	0	-0,50165	-46,8033	-2,67522	0,193712	-0,08656	-74,4373	-49,4785	7,15681
BTzPh9	269	Done	47,39	-6,07728	glide-grid_5 LOF_2	-7,52548	-2,96523	-0,16	0	-0,92744	-55,4029	-6,01753	0,332533	-0,13256	-86,8564	-61,4205	10,1735
BTz7	113	Done	32,57	-6,05103	glide-grid_5 LOF_2	-6,99433	-2,28489	-0,34466	0	-1,3448	-48,6548	-6,25519	0,391599	-0,04056	-77,8192	-54,91	9,95788
BT6	62	Done	32,14	-6,02068	glide-grid_5 LOF_2	-6,02068	-2,19341	-0,09268	0	-0,96923	-57,2769	-0,81287	0,287971	-0,06756	-74,7272	-58,0898	14,5012
BTzPhOMe4	418	Done	40,02	-5,9947	glide-grid_5	-7,010	-2,61	-0,48	0	-0,472	-41,79	-8,347	0,205543	-0,30	-77,62	-50,13	3,75915

				7	LOF_2	37	969			71	13	6		18	69	89	
BOx8	10	Done	33,45	-5,98056	glide-grid_5LOF_2	-5,98056	-2,79883	-0,16	0	-0,37444	-52,9226	-2,69256	0,402729	0	-72,4771	-55,6152	8,09918
BTzPhDMN6	362	Done	49,06	-5,97977	glide-grid_5LOF_2	-6,60027	-1,69808	-0,32	0	-0,49732	-42,2955	-13,3785	0,221516	-0,18484	-79,2325	-55,6741	17,1221
BOxPh7	27	Done	38,04	-5,97388	glide-grid_5LOF_2	-5,97388	-2,65961	-0,16	0	-0,26835	-59,2213	-1,15689	0,306757	-0,05808	-76,9508	-60,3782	13,0585
BTzNaf6	175	Done	39,16	-5,97338	glide-grid_5LOF_2	-6,96418	-3,35735	-0,16	0	-0,53296	-48,1098	-3,66456	0,210443	-0,16915	-76,8627	-51,7744	4,41149
BT2	58	Done	18,81	-5,93351	glide-grid_5LOF_2	-5,93351	-2,84289	-0,16	0	-0,1615	-38,1239	-6,12649	0,180737	-0,1247	-53,9045	-44,2504	8,82887
BTzPhOMe1	393	Done	20,26	-5,93036	glide-grid_5LOF_2	-7,18416	-3,30084	-0,12028	0	-0,42889	-60,4654	-2,51573	0,132423	-0,06594	-85,5819	-62,9812	12,7542
BOxPhOMe1	48	Done	21,29	-5,9288	glide-grid_5LOF_2	-5,9288	-3,17727	0	0	-0,24167	-52,2309	-0,09106	0,130906	-0,01556	-68,2723	-52,322	6,61575
BTzPhCl4	292	Done	34,68	-5,92699	glide-grid_5LOF_2	-7,31309	-3,1285	-0,29746	0	-0,87083	-57,3518	-1,62336	0,197663	-0,10287	-84,0745	-58,9752	4,6226
BTzPh9	268	Done	52,65	-5,92357	glide-grid_5LOF_2	-6,91437	-2,07289	-0,4875	0	-0,74893	-48,5991	-8,85597	0,333491	-0,18019	-82,8029	-57,4551	10,5016
BOxPhDMN5	43	Done	41,26	-5,90557	glide-grid_5LOF_2	-5,90557	-2,46713	-0,09563	0	-0,57709	-43,2131	-3,96592	0,201769	-0,21194	-65,2932	-47,1791	2,69491
BTzPhCl2	279	Done	21,64	-5,9028	glide-grid_5	-6,221	-2,40	-0,404	0	-0,071	-38,33	-8,817	0,154136	-0,25	-71,18	-47,15	3,72861

				2	LOF_2	52	991	91		16	8	22		019	3	53	
BTzPh9	267	Done	54,79	-5,88546	glide-grid_5LOF_2	-6,66556	-2,6958	-0,16	0	-0,96126	-59,7099	-0,38277	0,333491	-0,13908	-81,4178	-60,0926	16,5191
BTzPh8	259	Done	45,48	-5,87964	glide-grid_5LOF_2	-6,65974	-2,60035	-0,1288	0	-0,45714	-42,5239	-8,41246	0,322369	-0,40775	-75,8867	-50,9364	8,29295
BTzPh2	213	Done	29,06	-5,87922	glide-grid_5LOF_2	-6,36692	-2,06524	-0,50105	0	-0,71704	-30,8741	-10,284	0,214534	-0,21182	-65,9645	-41,1581	4,21728
BTzNaf4	160	Done	29,74	-5,86315	glide-grid_5LOF_2	-6,84665	-2,64739	-0,39061	0	-0,18859	-45,7431	-9,23174	0,174997	-0,12314	-81,6926	-54,9749	4,67904
BTzPh5	238	Done	32,52	-5,85973	glide-grid_5LOF_2	-6,92363	-2,41959	-0,4736	0	-0,6288	-56,6529	-5,33407	0,277558	-0,04645	-86,1685	-61,987	4,38331
BT4	60	Done	30,91	-5,85713	glide-grid_5LOF_2	-5,85713	-2,70076	0	0	-0,73864	-47,3302	-1,88517	0,241723	-0,01017	-64,8137	-49,2154	4,62917
BTzPhCl1	274	Done	21,26	-5,84852	glide-grid_5LOF_2	-6,35252	-2,19146	-0,19434	0	-1,20788	-42,443	-4,66517	0,127216	-0,06414	-72,5957	-47,1081	5,04312
BTzPhDMN6	364	Done	39,88	-5,84778	glide-grid_5LOF_2	-6,89418	-2,1022	-0,19951	0	-0,7971	-50,6187	-9,18835	0,220756	-0,10694	-80,6567	-59,807	16,9694
BOxPh2	22	Done	24,92	-5,8418	glide-grid_5LOF_2	-5,8418	-3,01367	-0,11308	0	-0,15727	-46,265	-2,28547	0,210609	-0,11232	-63,7075	-48,5505	9,02695
BTzNaf7	181	Done	47,17	-5,82592	glide-grid_5LOF_2	-6,38662	-2,72869	-0,08563	0	-0,28778	-49,2728	-5,40528	0,226133	-0,23622	-80,135	-54,6781	10,4307
BOxPhOMe3	50	Done	38,56	-5,8232	glide-grid_5	-5,823	-2,79	-0,292	0	-0,154	-51,65	-0,808	0,181871	-0,05	-69,43	-52,46	6,30127

				3	LOF_2	23	816	94		68	92	75		505	01	8	
BTzPh6	245	Done	38,84	-5,81044	glide-grid_5LOF_2	-7,25864	-2,8871	-0,16	0	-0,95177	-57,4415	-3,97708	0,294136	-0,08527	-84,9339	-61,4186	7,18472
BTzPhOMe6	436	Done	50,02	-5,80825	glide-grid_5LOF_2	-6,82235	-2,97124	-0,22063	0	-0,29547	-46,8909	-6,97693	0,241209	-0,18513	-76,9864	-53,8679	8,97397
BT7	63	Done	48,28	-5,80678	glide-grid_5LOF_2	-5,80678	-2,5207	-0,13119	0	-0,53894	-52,8113	-1,41638	0,306732	-0,06966	-73,1252	-54,2277	5,3888
BTzNaf6	177	Done	33,02	-5,805	glide-grid_5LOF_2	-8,1855	-4,08448	-0,14235	0	-0,80014	-62,3302	-0,23299	0,210443	-0,28741	-96,4993	-62,0972	11,8365
BTzPh7	252	Done	37,22	-5,78608	glide-grid_5LOF_2	-6,77688	-3,14389	-0,24166	0	-0,58364	-55,5956	-1,99453	0,309652	-0,03839	-81,6259	-57,5901	9,32989
BTzPhOMe5	430	Done	47,58	-5,78503	glide-grid_5LOF_2	-7,42543	-2,70738	-0,41737	0	-0,71692	-45,782	-8,76554	0,224435	-0,20427	-82,6065	-54,5476	5,74271
BTzNaf6	174	Done	39,11	-5,78015	glide-grid_5LOF_2	-6,56025	-2,87441	-0,09175	0	-0,3684	-54,4388	-4,37453	0,210443	-0,05801	-81,4426	-58,8133	7,26386
BTzPh2	216	Done	28,66	-5,77369	glide-grid_5LOF_2	-7,00049	-2,75075	-0,21748	0	-0,6381	-52,9346	-5,7541	0,212541	-0,09686	-80,6448	-58,6887	9,85281
BTzPh1	207	Done	15,8	-5,76541	glide-grid_5LOF_2	-6,73951	-1,89398	-0,34078	0	-1,179	-38,8619	-9,34413	0,18549	-0,16652	-69,1028	-48,2061	8,01215
BTz8	121	Done	42,47	-5,76532	glide-grid_5LOF_2	-6,70862	-2,0394	-0,54712	0	-0,42134	-45,5546	-9,29498	0,406764	-0,43554	-78,4472	-54,8496	16,5636
BTzPh2	215	Done	32,54	-5,7604	glide-grid_5	-6,832	-2,26	-0,337	0	-0,745	-51,78	-6,353	0,213536	-0,15	-86,01	-58,13	5,39458

				5	LOF_2	75	412	69		54	07	53		688	41	42	
BTzPh4	229	Done	27,11	-5,74905	glide-grid_5 LOF_2	-6,73255	-2,74273	-0,16	0	-0,81481	-51,2286	-2,5228	0,25966	-0,33482	-79,0342	-53,7514	6,5642
BTzPhOMe9	463	Done	69,02	-5,7483	glide-grid_5 LOF_2	-6,7624	-2,72875	-0,11806	0	-0,89919	-55,8709	-3,21232	0,281063	-0,02207	-79,2594	-59,0832	19,037
BTzPhCl4	291	Done	28,31	-5,74513	glide-grid_5 LOF_2	-6,88293	-2,42166	-0,47072	0	-0,92559	-50,093	-4,31962	0,198458	-0,11083	-78,5723	-54,4126	7,76813
BTzNaf4	158	Done	31,29	-5,74224	glide-grid_5 LOF_2	-6,30894	-3,00997	-0,08462	0	-0,15358	-52,844	-2,1973	0,175718	-0,26469	-80,6653	-55,0413	7,95156
BTzNaf2	145	Done	29,48	-5,74222	glide-grid_5 LOF_2	-6,60012	-2,91327	-0,2211	0	-0,12043	-50,8057	-4,47327	0,130594	-0,26464	-79,8649	-55,2789	7,40993
BTzPhOMe7	448	Done	49,14	-5,73659	glide-grid_5 LOF_2	-7,37249	-2,31518	-0,53669	0	0	-46,3321	-14,4542	0,256104	-0,29199	-83,567	-60,7863	22,0947
BTzPhCl9	320	Done	44,56	-5,72977	glide-grid_5 LOF_2	-6,62937	-2,34411	-0,16	0	-1,05238	-51,3696	-3,47269	0,274081	-0,25758	-80,0196	-54,8423	11,493
BTzPhOMe6	434	Done	50,95	-5,72723	glide-grid_5 LOF_2	-6,37533	-2,15782	-0,68949	0	0	-38,2243	-9,56791	0,242032	-0,42365	-75,1579	-47,7922	7,84994
BOx1	3	Done	28,38	-5,72265	glide-grid_5 LOF_2	-5,72265	-2,68297	-0,20912	0	-0,12664	-38,2084	-6,16605	0,222507	-0,09109	-55,6235	-44,3745	10,2393
BTzNaf3	153	Done	26,63	-5,70964	glide-grid_5 LOF_2	-7,04274	-2,85779	-0,48506	0	-0,24477	-41,9959	-8,99391	0,153343	-0,15959	-70,2094	-50,9898	5,54205
BTzNaf3	152	Done	33,05	-5,6988	glide-grid_5	-6,722	-3,09	-0,231	0	-0,212	-46,21	-5,278	0,154052	-0,23	-78,16	-51,49	2,11809

				3	LOF_2	53	664	89		05	43	45		351	23	28	
BTz3	80	Done	26,15	-5,69391	glide-grid_5LOF_2	-6,15021	-2,92149	-0,16	0	-0,38232	-43,0043	-4,9529	0,298207	-0,09145	-61,9357	-47,9572	10,8999
BTzPhDMN4	347	Done	37,5	-5,68192	glide-grid_5LOF_2	-6,24862	-1,93644	-0,39736	0	-0,31432	-41,7804	-10,0207	0,185962	-0,19434	-77,9568	-51,8011	7,1678
BOxPhOMe2	49	Done	34,95	-5,67904	glide-grid_5LOF_2	-5,67904	-2,66055	-0,05076	0	-0,1349	-48,0103	-3,05609	0,157917	-0,13182	-66,203	-51,0663	7,82687
BOx2	4	Done	39,61	-5,67734	glide-grid_5LOF_2	-5,67734	-2,55181	-0,16	0	-0,03399	-32,4594	-9,39549	0,261818	-0,16106	-56,0636	-41,8549	4,62415
BTzPhCl8	314	Done	46,94	-5,6718	glide-grid_5LOF_2	-6,5714	-2,509	-0,52554	0	0	-43,8121	-8,97945	0,262284	-0,26162	-77,3111	-52,7915	6,68516
BTzPhOMe2	399	Done	30,31	-5,6713	glide-grid_5LOF_2	-6,1928	-2,51871	-0,13068	0	-0,00621	-38,6953	-10,0035	0,161071	-0,26299	-73,545	-48,6988	10,3265
BTz4	90	Done	44,48	-5,66261	glide-grid_5LOF_2	-6,60371	-2,3754	-0,78614	0	-0,12084	-31,3301	-13,2716	0,328349	-0,09245	-67,5807	-44,6017	2,27861
BTzPhOMe2	401	Done	29,1	-5,65853	glide-grid_5LOF_2	-6,69073	-2,92359	-0,45952	0	-0,0874	-47,2576	-5,90101	0,160269	-0,13247	-73,316	-53,1586	13,0217
BOxPhDMN3	41	Done	32,07	-5,64317	glide-grid_5LOF_2	-5,64317	-2,25888	-0,04963	0	-0,42911	-54,4146	-1,17008	0,161947	-0,17125	-71,5739	-55,5847	6,66869
BTzPhCl9	319	Done	52,26	-5,62156	glide-grid_5LOF_2	-6,02046	-2,08558	-0,24547	0	-0,83789	-50,1002	-1,80295	0,274868	-0,35093	-73,3425	-51,9032	14,2898
BTzNaf8	190	Done	45,83	-5,6079	glide-grid_5	-6,388	-3,18	-0,162	0	-0,224	-53,92	-1,626	0,238783	-0,11	-74,36	-55,55	13,3323

				6	LOF_2	06	848	79		3	38	35		113	31	02	
BOxPhDMN6	44	Done	50,01	-5,60309	glide-grid_5LOF_2	-5,60309	-2,0333	0	0	-0,20506	-52,4965	-5,36613	0,218523	-0,15351	-74,446	-57,8626	7,79447
BTzPhCl3	283	Done	33,09	-5,60148	glide-grid_5LOF_2	-6,01948	-2,3505	-0,22382	0	-0,0602	-38,6932	-8,57262	0,17803	-0,34243	-70,4944	-47,2658	9,2419
BTzNaf2	146	Done	22,28	-5,58568	glide-grid_5LOF_2	-6,65798	-2,53887	-0,16966	0	-1,12747	-48,7198	-3,02348	0,130594	-0,06306	-75,869	-51,7433	5,23184
BTzPhCl5	295	Done	37,26	-5,5854	glide-grid_5LOF_2	-5,978	-2,29848	-0,23152	0	0	-42,6523	-7,30799	0,218122	-0,43732	-74,6767	-49,9603	5,34174
BTzPhOMe2	402	Done	31,55	-5,56537	glide-grid_5LOF_2	-7,10697	-3,23174	-0,40842	0	-0,19331	-50,3139	-5,81957	0,15947	-0,04434	-74,8791	-56,1335	10,2774
BOxPhOMe7	54	Done	51,75	-5,55433	glide-grid_5LOF_2	-5,55433	-2,43778	-0,09325	0	-0,13771	-50,7643	-3,35059	0,253695	-0,09848	-68,6878	-54,1149	9,35537
BTzNaf9	199	Done	63,27	-5,5512	glide-grid_5LOF_2	-6,542	-3,07534	-0,16	0	-0,21047	-49,8571	-4,86912	0,250747	-0,12371	-77,3369	-54,7262	6,71053
BTzPhCl2	281	Done	23,55	-5,54864	glide-grid_5LOF_2	-6,71194	-2,50719	-0,60648	0	-0,21382	-44,5504	-7,54476	0,153359	-0,17858	-76,8989	-52,0952	5,96073
BTzPhOMe2	403	Done	34,06	-5,54739	glide-grid_5LOF_2	-7,24809	-2,6014	-0,35222	0	-0,85366	-45,571	-6,83775	0,160269	-0,29687	-81,3777	-52,4088	4,12013
BTzPh5	237	Done	28,17	-5,52347	glide-grid_5LOF_2	-6,57187	-2,3129	-0,34772	0	-0,68379	-45,2592	-6,90033	0,278558	-0,208	-77,5578	-52,1596	5,96944
BT5	61	Done	29,6	-5,5223	glide-grid_5	-5,522	-2,31	-0,246	0	-0,371	-46,18	-2,687	0,266438	-0,14	-60,69	-48,87	12,1046

				7	LOF_2	37	438	8		68	26	93		363	33	06	
BTzNaf7	184	Done	48,09	-5,5214	glide-grid_5LOF_2	-6,9696	-2,38602	-0,60047	0	-0,06498	-52,8488	-9,46021	0,224677	-0,08134	-85,0668	-62,309	8,10059
BTz9	130	Done	50,29	-5,50425	glide-grid_5LOF_2	-6,44755	-2,22512	-0,16	0	-1,13699	-47,3636	-6,13585	0,419624	-0,05651	-75,3397	-53,4994	8,90291
BTzPhCl7	312	Done	38,52	-5,5026	glide-grid_5LOF_2	-8,0489	-2,5635	-0,62976	0	-1,11999	-56,0382	-6,98699	0,249015	-0,13471	-93,7945	-63,0252	11,7751
BTzPhOMe6	435	Done	57,49	-5,50187	glide-grid_5LOF_2	-6,51597	-2,289	-0,32	0	0	-45,2975	-9,46379	0,241209	-0,46373	-80,8728	-54,7613	9,99683
BTzPhOMe5	426	Done	43,94	-5,46667	glide-grid_5LOF_2	-6,48157	-2,01705	-0,16	0	-0,82848	-46,8739	-7,04667	0,224435	-0,29978	-71,0864	-53,9206	19,7577
BTzPhOMe1	391	Done	19,31	-5,46206	glide-grid_5LOF_2	-6,49026	-2,86193	-0,30696	0	-0,24915	-47,2803	-5,19621	0,133203	-0,06198	-75,9729	-52,4765	4,08574
BTzPh7	253	Done	44,29	-5,45988	glide-grid_5LOF_2	-6,90808	-2,3469	-0,37655	0	-0,6611	-53,2079	-6,28567	0,308669	-0,22895	-84,0412	-59,4936	6,26508
BTzPhCl2	280	Done	24,67	-5,44145	glide-grid_5LOF_2	-6,39025	-2,32145	-0,162	0	-0,71828	-45,6324	-5,379	0,153359	-0,25341	-73,4744	-51,0114	4,35487
BTzPhDMN2	333	Done	24,56	-5,43436	glide-grid_5LOF_2	-5,92206	-1,40968	-0,41126	0	-0,12371	-28,2221	-15,045	0,141211	-0,45076	-64,6032	-43,2671	7,16767
BTzPh6	242	Done	34,09	-5,4281	glide-grid_5LOF_2	-5,9888	-2,59273	0	0	-1,0854	-52,5725	-0,405813	0,296124	-0,03904	-79,3212	-52,1667	1,77669
BTzPhCl7	309	Done	39,08	-5,4228	glide-grid_5	-6,568	-2,55	-0,177	0	-0,737	-53,56	-3,867	0,249015	-0,09	-79,52	-57,43	11,2642

				8	LOF_2	58	212	89		96	7	75		112	44	48	
BTz1	67	Done	22,77	-<br>5,4160<br>8	glide-<br>grid_5<br>LOF_2	-<br>6,281<br>48	-<br>2,72<br>703	-<br>0,518<br>59	0	-<br>0,336<br>18	-<br>35,02<br>41	-<br>7,398<br>49	0,226<br>675	-<br>0,06<br>537	-<br>61,35<br>82	-<br>42,42<br>26	4,1788<br>7
BTzPhCl<br>8	313	Done	51,28	-<br>5,3861<br>6	glide-<br>grid_5<br>LOF_2	-<br>5,785<br>06	-<br>1,54<br>171	-<br>0,571<br>61	0	0	-<br>31,06<br>99	-<br>13,88<br>71	0,263<br>079	-<br>0,29<br>826	-<br>68,40<br>14	-<br>44,95<br>7	3,7938
BTzPh1	208	Done	18,48	-5,384	glide-<br>grid_5<br>LOF_2	-<br>6,640<br>8	-<br>3,05<br>987	-0,16	0	-<br>0,333<br>13	-<br>45,76<br>82	-<br>6,295<br>93	0,184<br>509	-<br>0,03<br>951	-<br>69,67<br>84	-<br>52,06<br>41	7,9882<br>2
BTzPhCl<br>8	316	Done	49,83	-<br>5,3787<br>5	glide-<br>grid_5<br>LOF_2	-<br>6,779<br>05	-<br>2,73<br>313	-<br>0,120<br>06	0	-<br>1,157<br>06	-<br>59,36<br>67	0,146<br>842	0,261<br>492	-<br>0,08<br>399	-<br>79,46<br>2	-<br>59,21<br>98	8,3056<br>7
BTzNaf1	139	Done	16,41	-<br>5,3733<br>8	glide-<br>grid_5<br>LOF_2	-<br>6,630<br>18	-<br>3,28<br>435	-<br>0,004<br>77	0	-<br>0,321<br>25	-<br>57,57<br>96	-<br>1,316<br>12	0,103<br>626	-<br>0,04<br>704	-<br>79,49<br>69	-<br>58,89<br>57	7,1147<br>3
BTzPh1	205	Done	22,77	-<br>5,3540<br>6	glide-<br>grid_5<br>LOF_2	-<br>6,020<br>96	-<br>3,14<br>116	-<br>0,046<br>14	0	-<br>0,426<br>68	-<br>37,14<br>52	-<br>2,827<br>37	0,186<br>474	-<br>0,31<br>209	-62,16	-<br>39,97<br>25	8,9197<br>3
BTzPhO<br>Me4	421	Done	29,85	-<br>5,3522<br>2	glide-<br>grid_5<br>LOF_2	-<br>6,996<br>62	-<br>2,61<br>456	-<br>0,462<br>46	0	-<br>0,719<br>21	-<br>40,76<br>61	-<br>8,185<br>23	0,205<br>543	-<br>0,13<br>984	-<br>77,55<br>67	-<br>48,95<br>13	5,7407<br>2
BTzPhCl<br>1	277	Done	16,51	-<br>5,3514<br>2	glide-<br>grid_5<br>LOF_2	-<br>6,508<br>72	-<br>3,06<br>142	-0,16	0	-<br>0,249<br>9	-<br>47,52<br>59	-<br>4,975<br>81	0,125<br>702	-<br>0,04<br>044	-<br>72,15<br>78	-<br>52,50<br>17	4,2053<br>2
BTzNaf2	147	Done	28,29	-<br>5,3445<br>5	glide-<br>grid_5<br>LOF_2	-<br>6,571<br>35	-<br>2,54<br>21	-<br>0,239<br>95	0	-<br>0,321<br>45	-<br>51,00<br>56	-<br>6,523<br>92	0,129<br>9	-<br>0,06<br>888	-<br>78,78<br>06	-<br>57,52<br>95	6,3997<br>6
BTzPh8	262	Done	34,38	-<br>5,3399<br>1	glide-<br>grid_5<br>LOF_2	-<br>7,720<br>41	-<br>4,04<br>656	-0,16	0	-<br>0,979<br>36	-<br>55,09<br>82	-<br>0,166<br>27	0,322<br>369	-<br>0,07<br>701	-<br>78,31<br>34	-<br>55,26<br>45	16,106<br>8
BTzPhCl<br>6	304	Done	32,99	-<br>5,3310	glide-<br>grid_5	-<br>6,731	-<br>2,63	-<br>0,250	0	-<br>0,892	-<br>53,78	-<br>2,712	0,233<br>296	-<br>0,09	-<br>78,35	-<br>56,49	6,9253<br>5

				6	LOF_2	36	422	63		63	62	05		106	62	83	
BTzPh3	222	Done	29,46	-5,32882	glide-grid_5LOF_2	-6,66192	-2,51568	-0,44945	0	-0,728	-47,3263	-5,04611	0,237111	-0,08267	-72,8182	-52,3725	2,98257
BTzPhCl7	308	Done	37,25	-5,32047	glide-grid_5LOF_2	-6,22007	-0,94031	-0,80052	0	0	-36,7005	-17,9867	0,249015	-0,19523	-75,292	-54,6872	10,7253
BTzNaf6	180	Done	31,21	-5,30651	glide-grid_5LOF_2	-7,97911	-3,62547	-0,11293	0	-0,79705	-65,0569	-1,97939	0,209716	-0,10362	-94,224	-67,0363	9,60002
BTzPh4	230	Done	36,87	-5,27291	glide-grid_5LOF_2	-6,70991	-2,58052	-0,32	0	-0,50401	-52,0609	-5,91293	0,258658	-0,07405	-78,4972	-57,9738	7,95603
BTzPhOMe5	428	Done	47,06	-5,25508	glide-grid_5LOF_2	-6,64608	-2,75513	-0,16116	0	-0,7642	-57,9469	-1,55254	0,223613	-0,05896	-80,3003	-59,4995	8,07229
BTzPhOMe7	444	Done	55,37	-5,24228	glide-grid_5LOF_2	-6,25638	-2,66136	-0,45893	0	-0,04473	-45,0109	-5,04815	0,256104	-0,3397	-70,4053	-50,0591	9,97977
BTzPhOMe7	446	Done	50,8	-5,2391	glide-grid_5LOF_2	-6,6193	-2,57274	-0,16	0	-0,40727	-53,244	-5,98442	0,255286	-0,17471	-76,5058	-59,2284	17,4753
BTzPhDMN5	357	Done	33,94	-5,21905	glide-grid_5LOF_2	-6,26745	-1,8181	-0,61606	0	-0,47784	-45,472	-7,70612	0,203997	-0,12993	-73,6403	-53,1781	8,25322
BTzPh2	214	Done	29,38	-5,21593	glide-grid_5LOF_2	-6,07383	-2,64009	-0,15136	0	-0,13551	-38,8817	-8,1967	0,213536	-0,18681	-69,9112	-47,0784	3,20509
BOxPhDMN7	45	Done	41,54	-5,21537	glide-grid_5LOF_2	-5,21537	-2,11379	0	0	-0,48166	-52,9243	-0,91165	0,233478	-0,07044	-67,8936	-53,836	7,45054
BTzPhDMN5	359	Done	40,09	-5,2120	glide-grid_5	-7,651	-2,45	-0,180	0	-0,913	-59,06	-8,340	0,203997	-0,10	-98,60	-67,40	9,21216

				8	LOF_2	38	547	78		07	02	92		191	01	11	
BTzNaf1	138	Done	19,58	-5,20304	glide-grid_5LOF_2	-6,17714	-2,25415	-0,22588	0	-0,13199	-43,4478	-9,07992	0,1043	-0,13504	-76,0921	-52,5277	-3,29943
BTzNaf8	192	Done	58,96	-5,19424	glide-grid_5LOF_2	-6,64244	-2,77063	-0,28872	0	-0,31319	-56,4964	-4,33994	0,238059	-0,03223	-81,1913	-60,8358	-9,71894
BTzPhDMN9	386	Done	61,43	-5,18975	glide-grid_5LOF_2	-6,24195	-2,08075	-0,34034	0	-0,83761	-47,3873	-5,41561	0,260195	-0,06174	-71,506	-52,803	-8,51658
BTzPh5	241	Done	34,09	-5,18651	glide-grid_5LOF_2	-7,78181	-3,28227	-0,5489	0	-0,30508	-54,3973	-7,86115	0,277558	-0,02409	-86,2834	-62,2585	-10,5787
BOx5	7	Done	37,28	-5,18188	glide-grid_5LOF_2	-5,18188	-2,44163	-0,16	0	0	-42,3649	-5,34722	0,348638	-0,00856	-61,4144	-47,7122	-5,91096
BTzPh9	273	Done	47,79	-5,17826	glide-grid_5LOF_2	-7,85086	-2,91318	-0,32093	0	-1,48486	-59,5987	-3,13607	0,332533	-0,01408	-89,2402	-62,7348	-7,78499
BTzPhCl7	307	Done	43,59	-5,1753	glide-grid_5LOF_2	-5,5742	-2,56797	0	0	-0,09068	-46,2507	-3,31738	0,249814	-0,35522	-70,6804	-49,5681	9,7769
BOx7	9	Done	33,98	-5,16568	glide-grid_5LOF_2	-5,16568	-2,17981	0	0	-0,27915	-41,8588	-5,46697	0,387492	-0,18122	-58,9867	-47,3258	-7,44937
BTzNaf2	148	Done	21,92	-5,14918	glide-grid_5LOF_2	-7,61408	-3,66223	-0,2293	0	-0,1335	-44,7874	-8,9542	0,130594	-0,13715	-82,6512	-53,7416	-2,90274
BOxPhOMe4	51	Done	24,91	-5,14482	glide-grid_5LOF_2	-5,14482	-2,43881	-0,00041	0	-0,0988	-45,1874	-3,20576	0,203133	-0,06971	-60,9204	-48,3932	-6,00213
BTzPhOMe5	427	Done	38,49	-5,1379	glide-grid_5	-6,152	-1,87	-0,652	0	-0,102	-44,73	-7,393	0,224435	-0,40	-74,57	-52,12	9,59223

				8	LOF_2	88	161	11		66	25	62		527	11	61	
BTz2	74	Done	28,88	-5,1327	glide-grid_5LOF_2	-5,7107	-2,24977	-0,46102	0	-0,58962	-41,0568	-3,97916	0,264611	-0,02519	-54,7371	-45,0359	13,3021
BOxNaf9	20	Done	59,85	-5,13191	glide-grid_5LOF_2	-5,13191	-2,19118	0	0	-0,00323	-59,9625	-0,80722	0,248626	-0,06692	-75,821	-60,7698	8,38399
BTzPh4	227	Done	30,43	-5,1291	glide-grid_5LOF_2	-5,6958	-2,38739	-0,06191	0	-0,6374	-40,0543	-3,93023	0,260666	-0,27751	-65,681	-43,9845	4,28558
BTzPhOMe3	410	Done	35,27	-5,12425	glide-grid_5LOF_2	-6,46225	-2,26138	-0,41445	0	-0,72551	-52,0273	-3,76018	0,183449	-0,07896	-75,5529	-55,7875	5,76904
BTzPh6	244	Done	34,16	-5,11087	glide-grid_5LOF_2	-6,10167	-2,3575	-0,26278	0	-0,6543	-50,2222	-3,51551	0,295128	-0,08379	-73,6898	-53,7377	8,95269
BTzPhOMe3	409	Done	30,96	-5,10889	glide-grid_5LOF_2	-6,12619	-2,34646	-0,29098	0	0	-50,7233	-5,98975	0,18426	-0,23838	-74,1128	-56,7131	19,1215
BT9	65	Done	46,63	-5,10707	glide-grid_5LOF_2	-5,10707	-2,2912	-0,02771	0	-0,46532	-51,1952	-0,3956	0,337282	-0,04102	-63,0282	-51,5908	7,18063
BOx3	5	Done	35,63	-5,10683	glide-grid_5LOF_2	-5,10683	-2,34086	-0,03291	0	-0,25008	-42,4923	-4,18945	0,295398	-0,02534	-52,9416	-46,6817	14,8256
BTzNaf5	169	Done	30,53	-5,09326	glide-grid_5LOF_2	-6,15716	-2,7799	-0,08997	0	-0,65733	-51,5666	-1,48258	0,192988	-0,02223	-70,2182	-53,0492	6,60147
BTzPhCl1	275	Done	16,1	-5,08786	glide-grid_5LOF_2	-5,98536	-2,6604	-0,00555	0	-0,14937	-47,4155	-4,50065	0,126457	-0,25062	-74,0106	-51,9161	4,87201
BTzPhDMN9	383	Done	65,1	-5,0818	glide-grid_5	-5,702	-1,88	-0,403	0	-0,275	-46,48	-5,441	0,261694	-0,26	-72,74	-51,93	9,50257

				9	LOF_2	39	057	54		24	91	53		405	46	06	
BTzPhCl2	282	Done	24,49	-5,07845	glide-grid_5LOF_2	-6,65605	-2,53424	-0,24728	0	-0,73145	-55,7631	-2,72866	0,152585	-0,09823	-83,1876	-58,4917	2,94636
BTzPhCl5	300	Done	32,77	-5,07561	glide-grid_5LOF_2	-7,62941	-3,5222	-0,16	0	-0,29176	-53,3096	-7,29263	0,21732	-0,11339	-88,8514	-60,6023	8,75978
BTzPhOMe1	395	Done	19,3	-5,06954	glide-grid_5LOF_2	-6,81834	-2,38748	-0,16	0	-0,96773	-45,9048	-5,90934	0,133203	-0,25469	-72,3771	-51,8142	13,8007
BOxPhCl2	31	Done	27,18	-5,06819	glide-grid_5LOF_2	-5,06819	-1,98541	-0,17873	0	-0,05	-46,1257	-3,88096	0,151079	-0,11671	-63,9687	-50,0066	6,41584
BTzPhOMe3	412	Done	29,77	-5,0678	glide-grid_5LOF_2	-6,7199	-2,60036	-0,31033	0	-0,06193	-45,1775	-9,81253	0,18426	-0,20079	-79,5652	-54,99	5,65344
BTzPhDMN4	349	Done	25,34	-5,05825	glide-grid_5LOF_2	-6,04175	-2,27536	-0,16	0	-0,41224	-47,0093	-4,46388	0,185208	-0,35931	-72,8501	-51,4732	6,85369
BTzPhDMN1	327	Done	19,96	-5,05341	glide-grid_5LOF_2	-6,02751	-1,45499	-0,46674	0	-0,37181	-32,6871	-13,8142	0,113909	-0,14138	-65,2332	-46,5014	7,33313
BTzNaf1	136	Done	22,49	-5,04372	glide-grid_5LOF_2	-5,71062	-2,52576	-0,16	0	-0,24256	-47,7178	-2,89611	0,104976	-0,06697	-72,7663	-50,6139	7,16438
BTzPhDMN2	336	Done	28,41	-5,04231	glide-grid_5LOF_2	-6,26911	-2,67962	-0,14811	0	-0,21444	-45,7038	-7,12923	0,139751	-0,01211	-71,41	-52,8331	4,3158
BTzNaf4	163	Done	28,31	-5,03971	glide-grid_5LOF_2	-7,49811	-3,21422	-0,30047	0	-0,46063	-50,1363	-7,2023	0,174997	-0,11063	-87,0671	-57,3386	4,33719
BTzPhOMe9	462	Done	74,34	-5,0389	glide-grid_5	-6,053	-1,69	-0,408	0	-0,014	-44,70	-11,86	0,281063	-0,19	-78,36	-56,57	11,1861

				7	LOF_2	07	78	35		04	31	91		843	95	22	
BTzNaf9	201	Done	56,12	-5,03877	glide-grid_5LOF_2	-7,41927	-3,319	-0,20949	0	-0,3051	-54,5226	-5,64465	0,250747	-0,2636	-89,9106	-60,1673	6,99553
BTzPhOMe5	429	Done	37,54	-5,03645	glide-grid_5LOF_2	-6,67685	-2,67305	-0,31187	0	-0,19248	-49,3798	-7,12881	0,224435	-0,18558	-80,8729	-56,5086	10,5786
BTzPh1	206	Done	17,51	-5,03109	glide-grid_5LOF_2	-5,77469	-2,29086	-0,29761	0	-0,87455	-44,5705	-1,34179	0,18549	-0,06735	-65,2274	-45,9123	4,1886
BTzPhDMN6	363	Done	48,32	-5,03012	glide-grid_5LOF_2	-5,85242	-1,7342	-0,22311	0	-0,48592	-38,1444	-10,3036	0,220756	-0,1772	-66,1821	-48,448	8,27887
BTz9	128	Done	39,18	-5,02363	glide-grid_5LOF_2	-5,48233	-1,18853	-0,29009	0	-0,04356	-31,3034	-17,4779	0,41828	-0,19158	-62,4866	-48,7813	9,72084
BTzPhDMN3	341	Done	26,32	-5,02336	glide-grid_5LOF_2	-6,04706	-1,70028	-0,58406	0	-0,49899	-41,524	-7,32282	0,164133	-0,25324	-70,0512	-48,8468	6,0367
BTzNaf6	176	Done	42,09	-4,99761	glide-grid_5LOF_2	-6,44581	-2,71587	0	0	-0,63385	-58,7591	-2,3365	0,209716	-0,01737	-83,0914	-61,0956	3,97011
BTz6	104	Done	41,09	-4,99615	glide-grid_5LOF_2	-5,45585	-1,3368	-0,14472	0	-1,20403	-50,1671	-3,39871	0,372355	-0,12449	-68,1723	-53,5658	9,93797
BTz1	66	Done	23,09	-4,99051	glide-grid_5LOF_2	-5,47431	-2,70344	-0,15766	0	-0,17894	-36,1737	-5,40084	0,225258	-0,04071	-52,3268	-41,5745	8,88965
BTzPhCl6	302	Done	38,34	-4,98385	glide-grid_5LOF_2	-5,88345	-2,59234	-0,27758	0	-0,05235	-43,6016	-4,96176	0,234095	-0,27093	-68,0103	-48,5633	6,99227
BTzPhOMe8	452	Done	55,09	-4,9796	glide-grid_5	-5,627	-1,72	-0,287	0	0	-40,48	-9,864	0,270143	-0,38	-70,37	-50,34	17,6968

				9	LOF_2	79	304	91			24	38		321	27	67	
BTzPh4	228	Done	22,52	-4,97023	glide-grid_5LOF_2	-5,75123	-1,90703	-0,37386	0	-0,0446	-42,8692	-8,95528	0,25966	-0,19865	-73,6664	-51,8245	4,77748
BTzPh6	247	Done	35,74	-4,96642	glide-grid_5LOF_2	-7,41712	-2,55891	-0,32	0	-0,88566	-56,6529	-6,57954	0,295128	-0,1281	-93,4991	-63,2325	8,25298
BTzPhOMe5	432	Done	39,06	-4,95723	glide-grid_5LOF_2	-6,97373	-2,5458	-0,35974	0	-0,59484	-55,616	-5,39384	0,223613	-0,10709	-86,0991	-61,0098	4,37852
BTz2	76	Done	30,27	-4,94909	glide-grid_5LOF_2	-5,89269	-2,27872	-0,32644	0	-0,45784	-36,3727	-7,4728	0,26605	-0,15617	-59,1096	-43,8455	11,5344
BTzPhOMe7	449	Done	52,66	-4,93878	glide-grid_5LOF_2	-6,94068	-1,93252	-0,60971	0	-0,6801	-45,6413	-9,68232	0,255286	-0,23922	-78,2213	-55,3236	6,36123
BTz2	75	Done	32,75	-4,92679	glide-grid_5LOF_2	-5,72679	-2,4009	-0,32	0	0	-33,3448	-8,45393	0,26605	-0,33661	-58,0426	-41,7987	8,23729
BTzPhDMN9	384	Done	66,27	-4,92609	glide-grid_5LOF_2	-5,74839	-1,24144	-0,3975	0	-0,31454	-47,5509	-9,87005	0,260944	-0,1978	-75,8509	-57,421	11,5557
BTzPhDMN8	377	Done	60,49	-4,91583	glide-grid_5LOF_2	-5,73813	-1,62077	-0,51321	0	-0,35602	-50,0817	-3,97715	0,249046	-0,39652	-75,387	-54,0588	7,65637
BTzPhDMN1	331	Done	16,3	-4,91144	glide-grid_5LOF_2	-7,50864	-3,11032	-0,37891	0	-0,48418	-36,2456	-10,8501	0,113909	-0,20935	-69,906	-47,0957	6,2888
BTzPhOMe4	423	Done	34,97	-4,89403	glide-grid_5LOF_2	-6,89003	-2,58759	-0,19535	0	-0,82437	-50,6603	-5,66259	0,204725	-0,10504	-78,0356	-56,3229	5,4538
BTzNaf7	186	Done	41,31	-4,8816	glide-grid_5	-7,332	-2,69	-0,588	0	-0,162	-43,26	-11,91	0,225404	-0,15	-80,21	-55,18	9,71852

				7	LOF_2	37	938	7		33	37	79		649	01	16	
BTzPh5	239	Done	26,36	-4,88097	glide-grid_5LOF_2	-7,32027	-2,89371	-0,53512	0	-0,18143	-45,5978	-10,8724	0,278558	-0,07781	-83,9021	-56,4702	8,23882
BTzPh8	261	Done	46,24	-4,87274	glide-grid_5LOF_2	-6,32094	-2,3004	-0,22829	0	-0,76333	-59,2449	-2,03274	0,321398	-0,08317	-79,2998	-61,2776	12,8164
BTzPhDMN7	370	Done	39,91	-4,86605	glide-grid_5LOF_2	-5,68835	-1,76022	-0,37065	0	-0,39411	-47,2066	-6,19366	0,235707	-0,10969	-73,1379	-53,4003	6,67412
BT3	59	Done	22,32	-4,844	glide-grid_5LOF_2	-4,844	-1,87678	-0,02453	0	-0,58397	-50,336	-0,04835	0,213345	-0,04802	-59,7146	-50,3844	10,902
BTzPh4	233	Done	24,81	-4,84206	glide-grid_5LOF_2	-7,36546	-2,51393	-0,32	0	-1,00198	-51,5007	-6,63004	0,258658	-0,21866	-82,0146	-58,1307	6,92745
BA	1	Done	3,7	-4,83616	glide-grid_5LOF_2	-4,84386	-2,13536	-0,41032	0	-0,35052	-24,5071	-4,93824	0,096752	-0,07832	-42,1615	-29,4453	0,257526
BTzNaf4	161	Done	26,09	-4,83295	glide-grid_5LOF_2	-6,26995	-2,9774	-0,18964	0	-0,37391	-51,2303	-1,8244	0,174279	-0,06811	-71,393	-53,0547	6,18265
BTzPh8	264	Done	37,51	-4,82746	glide-grid_5LOF_2	-7,35956	-2,94811	-0,32	0	-0,67523	-53,4592	-6,19787	0,321398	-0,13498	-83,8419	-59,6571	9,14165
BOxPhOMe8	55	Done	65,7	-4,82552	glide-grid_5LOF_2	-4,82552	-1,65264	0	0	-0,2541	-58,7793	-1,47884	0,266935	-0,02492	-71,6366	-60,2581	11,8204
BOxPhDMN1	39	Done	18,69	-4,81545	glide-grid_5LOF_2	-4,81545	-1,25713	-0,16	0	-0,33301	-43,7115	-5,70715	0,111822	-0,13548	-61,7168	-49,4187	6,63615
BTzNaf1	142	Done	15,83	-4,8097	glide-grid_5	-7,406	-3,40	-0,16	0	-0,254	-57,95	-3,433	0,1043	-0,27	-89,46	-61,38	8,13247

				2	LOF_2	92	978			37	08	5		45	77	43	
BTz5	97	Done	33,01	-4,80749	glide-grid_5LOF_2	-5,74309	-1,99146	-0,32	0	-0,37628	-32,5805	-10,9596	0,352856	-0,13524	-61,4685	-43,5402	5,64981
BTzNaf1	143	Done	19,09	-4,78801	glide-grid_5LOF_2	-7,41511	-2,98139	-0,48	0	-0,31622	-50,1382	-7,63536	0,103626	-0,08891	-80,6761	-57,7736	5,18018
BTz8	122	Done	38,6	-4,78414	glide-grid_5LOF_2	-5,72744	-1,6049	-0,15471	0	-1,15949	-40,9788	-5,67357	0,406764	-0,31513	-64,1045	-46,6524	9,75363
BTzPhDMN7	372	Done	37,46	-4,77714	glide-grid_5LOF_2	-5,82934	-1,24227	-0,64	0	-0,15132	-41,3521	-12,2984	0,23495	-0,11834	-69,6553	-53,6505	8,07829
BTzPhOMe3	407	Done	33,61	-4,76616	glide-grid_5LOF_2	-5,44176	-2,06452	-0,25587	0	-0,77553	-44,3535	-1,40590	0,185074	-0,10235	-68,7639	-45,7594	7,58355
BTzPhOMe3	408	Done	36,48	-4,75859	glide-grid_5LOF_2	-5,75489	-1,81828	-0,29486	0	-0,11953	-45,3233	-8,79991	0,18426	-0,12033	-76,1022	-54,1232	4,84644
BTzPhDMN6	365	Done	47,44	-4,75715	glide-grid_5LOF_2	-5,80935	-1,73323	-0,41351	0	-0,6048	-43,0084	-6,9716	0,219998	-0,08165	-66,7196	-49,9799	5,4418
BTzPhDMN2	335	Done	18,76	-4,75248	glide-grid_5LOF_2	-5,82478	-2,41738	0	0	-0,43756	-50,4273	-1,78643	0,14048	-0,32099	-71,9899	-52,2138	9,01282
BTzNaf4	165	Done	33,39	-4,74876	glide-grid_5LOF_2	-7,41496	-3,20963	-0,32843	0	-0,15146	-56,9536	-5,86359	0,174279	-0,17251	-90,3357	-62,8172	5,95777
BTzPhDMN5	356	Done	36,49	-4,74228	glide-grid_5LOF_2	-5,56598	-1,70265	-0,25365	0	-0,19226	-44,5363	-8,44262	0,203997	-0,12821	-70,9585	-52,9789	9,66761
BTzPhOMe3	411	Done	37,39	-4,7350	glide-grid_5	-6,361	-2,31	-0,275	0	-0,161	-54,30	-6,644	0,18426	-0,08	-83,72	-60,95	11,8754

				1	LOF_2	01	019	02		72	69	57		631	77	15	
BTzNaf5	171	Done	38,37	-4,73348	glide-grid_5 LOF_2	-7,24768	-3,29578	-0,32	0	-0,62811	-55,0099	-2,29721	0,193712	-0,10243	-88,0097	-57,3071	4,35992
BTzPhOMe2	404	Done	23,36	-4,73266	glide-grid_5 LOF_2	-6,43916	-2,07786	-0,24787	0	-0,82309	-44,0495	-6,1866	0,160269	-0,32014	-71,9688	-50,2361	10,0733
BTzNaf7	187	Done	40,89	-4,72352	glide-grid_5 LOF_2	-7,25562	-2,2974	-0,79865	0	-0,15964	-45,7454	-11,7648	0,224677	-0,17262	-80,7914	-57,5102	11,6017
BTzNaf8	195	Done	45,97	-4,7009	glide-grid_5 LOF_2	-7,233	-3,31797	-0,27423	0	-0,24127	-56,5216	-4,36534	0,238059	-0,15671	-85,485	-60,887	8,33215
BTzPh7	257	Done	36,09	-4,69232	glide-grid_5 LOF_2	-7,36492	-2,63455	-0,47654	0	-0,56517	-54,2495	-6,95783	0,308669	-0,24119	-88,1664	-61,2074	6,29056
BTzNaf2	149	Done	24,41	-4,67738	glide-grid_5 LOF_2	-7,14808	-3,53747	-0,15024	0	-0,86324	-46,0768	-1,71415	0,130594	-0,16677	-73,0723	-47,7909	4,2459
BTzPhOMe7	447	Done	53,91	-4,67067	glide-grid_5 LOF_2	-6,30657	-1,75463	-0,47002	0	-0,14916	-44,5692	-11,515	0,256104	-0,23315	-80,5483	-56,0842	6,28434
BTzPh3	223	Done	38,62	-4,66936	glide-grid_5 LOF_2	-7,03436	-2,78267	-0,29234	0	-1,18691	-52,3356	-2,00683	0,238112	-0,09274	-78,992	-54,3424	5,76837
BTzPhDMN3	339	Done	33	-4,66901	glide-grid_5 LOF_2	-5,26321	-1,67832	-0,42625	0	-0,08352	-35,8395	-8,04977	0,164879	-0,24056	-64,0062	-43,8893	4,38412
BTzPhCl5	299	Done	31,2	-4,66535	glide-grid_5 LOF_2	-7,21915	-3,27223	-0,32	0	-0,01572	-44,6988	-9,27824	0,21732	-0,20184	-81,2428	-53,9771	6,43475
BTzPhOMe1	390	Done	19,53	-4,6610	glide-grid_5	-5,347	-1,89	-0,283	0	-0,115	-32,82	-8,932	0,133987	-0,20	-64,69	-41,75	4,23696

				6	LOF_2	16	207	1		32	66	6		944	32	92	
BTzPhCl9	324	Done	47,38	-4,62938	glide-grid_5LOF_2	-7,17568	-2,60346	-0,32	0	-1,05224	-50,071	-4,86274	0,274081	-0,2411	-85,9401	-54,9338	6,30273
BTz2	77	Done	29,55	-4,62728	glide-grid_5LOF_2	-5,88518	-2,11501	-0,43802	0	-0,08981	-33,8682	-10,7396	0,267495	-0,20547	-61,7076	-44,6078	13,7053
BTzPhOMe2	406	Done	27,35	-4,62682	glide-grid_5LOF_2	-6,84272	-2,56763	-0,25799	0	-0,3993	-48,4255	-8,55986	0,15947	-0,07202	-72,3995	-56,9853	17,4155
BTzPhCl8	317	Done	49,38	-4,58747	glide-grid_5LOF_2	-7,13377	-2,44408	-0,636	0	-0,78567	-47,3829	-6,57063	0,262284	-0,17556	-76,4239	-53,9536	11,3354
BTz4	92	Done	38,54	-4,57093	glide-grid_5LOF_2	-6,93973	-2,94784	-0,09475	0	-1,32554	-49,7085	-2,58098	0,326906	-0,02593	-71,8363	-52,2894	5,902
BTzPhOMe3	414	Done	30,41	-4,55658	glide-grid_5LOF_2	-6,52938	-2,11991	-0,39245	0	-0,74429	-54,5809	-4,37877	0,183449	-0,07031	-79,2293	-58,9597	5,97109
BTzPhOMe2	400	Done	28,3	-4,53976	glide-grid_5LOF_2	-5,57066	-1,02634	-0,61538	0	0	-28,6143	-14,9642	0,160269	-0,41386	-64,1453	-43,5786	3,79568
BTzPhCl3	287	Done	31,01	-4,52907	glide-grid_5LOF_2	-7,05697	-2,53183	-0,4404	0	-0,849	-43,4769	-5,95344	0,17724	-0,34612	-74,8163	-49,4303	5,63117
BTzPh9	266	Done	46,13	-4,52844	glide-grid_5LOF_2	-5,08914	-1,22436	-0,34089	0	0	-39,9942	-9,45454	0,334451	-0,44046	-67,6792	-49,4487	11,747
BTzNaf7	188	Done	48,46	-4,51707	glide-grid_5LOF_2	-7,18967	-3,33078	-0,18193	0	-0,22558	-62,8182	-2,4469	0,224677	-0,16811	-88,205	-65,2651	12,4648
BTzPhDMN4	350	Done	32,37	-4,5159	glide-grid_5	-5,952	-1,18	-0,753	0	-0,025	-38,78	-13,22	0,184456	-0,24	-68,66	-52,01	10,8146

				5	LOF_2	95	567	39		63	37	63		958	64		
BTzNaf3	154	Done	27,7	-4,51107	glide-grid_5 LOF_2	-6,87607	-3,07144	-0,30123	0	-0,15223	-44,2389	-7,22375	0,154052	-0,20972	-75,9429	-51,4626	5,64093
BTzPhOMe4	420	Done	33,39	-4,50684	glide-grid_5 LOF_2	-6,14054	-2,23031	-0,56145	0	-0,53867	-41,233	-5,35785	0,205543	-0,15033	-66,2115	-46,5908	7,94592
BTz9	133	Done	47,9	-4,50441	glide-grid_5 LOF_2	-6,85921	-2,15782	-0,16	0	-1,14601	-54,6834	-5,84688	0,41828	-0,20245	-81,9239	-60,5303	13,4368
BTzPh6	249	Done	31,2	-4,49095	glide-grid_5 LOF_2	-7,16355	-2,81186	-0,42727	0	-1,11925	-53,9237	-2,61719	0,294136	-0,01055	-79,8556	-56,5409	8,96278
BTz1	68	Done	22,96	-4,48565	glide-grid_5 LOF_2	-5,40585	-2,50891	-0,1428	0	-0,04226	-31,7375	-7,18202	0,226675	-0,27437	-52,2544	-38,9195	10,914
BTzPhOMe8	454	Done	51,39	-4,4821	glide-grid_5 LOF_2	-5,4962	-2,20063	-0,2067	0	-0,1844	-53,2543	-2,11269	0,269328	-0,19418	-68,6332	-55,367	21,4615
BTzPh7	251	Done	43,98	-4,47653	glide-grid_5 LOF_2	-5,25663	-2,03149	-0,16	0	-0,51692	-48,5485	-2,24491	0,309652	-0,0937	-69,2801	-50,7934	6,72059
BOxPhDMN9	47	Done	61,26	-4,45964	glide-grid_5 LOF_2	-4,45964	-1,39718	0	0	-0,24811	-57,2562	-0,92861	0,258739	-0,07099	-70,4283	-58,1848	8,41092
BTzPhDMN7	374	Done	49,4	-4,45908	glide-grid_5 LOF_2	-6,96818	-2,81185	-0,40849	0	-0,31225	-55,3344	-3,65278	0,235707	-0,35668	-82,5523	-58,9872	14,7141
BTz7	118	Done	39,21	-4,45856	glide-grid_5 LOF_2	-7,00356	-2,79482	-0,64	0	-0,26847	-44,2838	-9,09915	0,390204	-0,11141	-73,0619	-53,383	9,82825
BTz8	120	Done	38,98	-4,4474	glide-grid_5	-4,906	-1,38	-0,157	0	-0,970	-48,57	-2,086	0,405393	-0,05	-61,57	-50,66	8,46372

				5	LOF_2	15	267	46		26	75	75		928	87	42	
BTzNaf5	168	Done	32,16	-<br>4,4461<br>2	glide-<br>grid_5<br>LOF_2	-<br>5,494<br>52	-<br>1,83<br>345	0	0	-<br>0,399<br>84	-<br>57,01<br>29	-<br>3,943<br>91	0,193<br>712	-<br>0,01<br>272	-<br>81,36<br>25	-<br>60,95<br>68	6,8874<br>3
BTzPh1	212	Done	19,28	-<br>4,4447<br>2	glide-<br>grid_5<br>LOF_2	-<br>7,071<br>82	-<br>2,86<br>238	-<br>0,474<br>04	0	-<br>0,286<br>84	-<br>44,40<br>44	-<br>8,798<br>42	0,184<br>509	-<br>0,09<br>308	-<br>76,58<br>1	-<br>53,20<br>29	4,4070<br>4
BTz6	107	Done	36,87	-<br>4,4420<br>8	glide-<br>grid_5<br>LOF_2	-<br>6,041<br>48	-<br>2,66<br>688	-0,32	0	-<br>0,095<br>69	-<br>27,00<br>95	-<br>11,00<br>39	0,375<br>193	-<br>0,33<br>305	-<br>57,81<br>19	-<br>38,01<br>34	9,3011<br>1
BTzNaf3	157	Done	26,85	-<br>4,4336<br>7	glide-<br>grid_5<br>LOF_2	-<br>7,112<br>87	-<br>3,42<br>8	-<br>0,170<br>19	0	-<br>0,461<br>03	-<br>55,33<br>85	-<br>2,360<br>92	0,153<br>343	-<br>0,08<br>593	-<br>80,18<br>86	-<br>57,69<br>94	7,2901
BTzPh7	255	Done	40,94	-<br>4,4048<br>8	glide-<br>grid_5<br>LOF_2	-<br>6,855<br>58	-<br>2,67<br>494	-0,64	0	-<br>0,147<br>15	-<br>37,17<br>63	-<br>11,24<br>8	0,309<br>652	-<br>0,15<br>713	-<br>71,14<br>12	-<br>48,42<br>44	8,1199<br>9
BTzPhO<br>Me6	440	Done	54,05	-<br>4,4025<br>4	glide-<br>grid_5<br>LOF_2	-<br>6,404<br>44	-<br>2,61<br>352	-<br>0,088<br>86	0	-<br>0,840<br>35	-<br>56,34<br>38	-<br>1,311<br>01	0,240<br>388	-<br>0,08<br>825	-<br>71,06<br>32	-<br>57,65<br>48	15,195<br>7
BTzPh3	221	Done	29,98	-<br>4,3942<br>7	glide-<br>grid_5<br>LOF_2	-<br>5,417<br>97	-<br>2,12<br>938	-<br>0,155<br>06	0	-<br>0,133<br>84	-<br>42,18<br>91	-<br>6,860<br>52	0,238<br>112	-<br>0,09<br>927	-<br>67,36<br>19	-<br>49,04<br>96	7,9329<br>9
BTz8	127	Done	35,7	-<br>4,3934<br>7	glide-<br>grid_5<br>LOF_2	-<br>6,938<br>47	-<br>2,50<br>507	-<br>0,758<br>24	0	-<br>0,227<br>27	-<br>45,30<br>02	-<br>9,994<br>39	0,405<br>393	-<br>0,08<br>913	-<br>77,85<br>5	-<br>55,29<br>46	7,2148<br>7
BTzPhO<br>Me4	424	Done	40,29	-<br>4,3701<br>6	glide-<br>grid_5<br>LOF_2	-<br>6,992<br>96	-<br>2,97<br>134	-<br>0,266<br>34	0	-<br>0,675<br>19	-<br>54,29<br>94	-<br>3,192<br>66	0,204<br>725	-<br>0,09<br>096	-<br>75,50<br>92	-<br>57,49<br>21	12,878<br>2
BTzNaf3	156	Done	26,93	-<br>4,3573	glide-<br>grid_5<br>LOF_2	-<br>6,845<br>1	-<br>2,72<br>942	-<br>0,407<br>9	0	-<br>0,117<br>49	-<br>41,91<br>87	-<br>9,861<br>03	0,154<br>052	-<br>0,16<br>925	-<br>74,31<br>44	-<br>51,77<br>97	8,3031<br>7
BTzPh7	250	Done	37,52	-<br>4,3550	glide-<br>grid_5	-<br>4,915	-<br>1,50	-<br>0,133	0	-<br>0,154	-<br>43,86	-<br>7,114	0,310<br>638	-<br>0,17	-<br>67,15	-<br>50,98	12,009<br>7

				3	LOF_2	73	672	59		12	9			139	88	3	
BTzPhOMe9	468	Done	57,85	-4,35322	glide-grid_5LOF_2	-6,35512	-2,08534	-0,4716	0	-0,75336	-47,12	-5,93626	0,280258	-0,07864	-68,6284	-53,0563	13,4234
BTzPhCl4	293	Done	36,22	-4,34752	glide-grid_5LOF_2	-6,88942	-2,52664	-0,16	0	-1,04954	-57,2227	-1,63258	0,198458	-0,24568	-85,2598	-58,8553	8,98498
BTzPhOMe4	417	Done	37,11	-4,34375	glide-grid_5LOF_2	-5,35065	-2,41369	0	0	-0,43725	-42,9861	-2,24043	0,205543	-0,21988	-64,4779	-45,2265	6,07493
BTzNaf1	140	Done	17,84	-4,3404	glide-grid_5LOF_2	-6,6451	-2,07496	-0,19141	0	-0,80459	-41,0751	-10,1558	0,1043	-0,10133	-72,1195	-51,2308	14,037
BTzPhOMe2	405	Done	33,78	-4,32754	glide-grid_5LOF_2	-6,53884	-2,69353	-0,2893	0	-0,67627	-51,6329	-2,39326	0,15947	-0,09858	-70,3288	-54,0262	14,5336
BTzPhDMN9	387	Done	65	-4,31488	glide-grid_5LOF_2	-6,74928	-2,34679	-0,29775	0	-0,56114	-48,1793	-8,66371	0,260944	-0,09603	-81,5499	-56,843	6,59848
BTzPhDMN6	367	Done	45,73	-4,31229	glide-grid_5LOF_2	-6,82139	-1,29947	-0,85936	0	-0,31038	-37,642	-16,7918	0,220756	-0,17208	-77,3972	-54,4338	10,8706
BTzPhDMN3	346	Done	34,65	-4,31188	glide-grid_5LOF_2	-6,99108	-2,60867	-0,32	0	-0,35758	-50,7499	-8,09488	0,163391	-0,1165	-80,2768	-58,8448	8,01203
BOx6	8	Done	35,98	-4,309	glide-grid_5LOF_2	-4,309	-2,07512	0	0	-0,18623	-46,8446	-0,48908	0,369602	-0,00166	-58,0148	-47,3337	6,13715
BTzNaf5	172	Done	34,72	-4,30693	glide-grid_5LOF_2	-6,90223	-2,89099	-0,39084	0	-0,43099	-50,8694	-4,97456	0,192988	-0,09274	-78,9011	-55,844	5,14513
BTzPhDMN1	326	Done	15,71	-4,2848	glide-grid_5	-5,028	-2,29	-0,111	0	-0,101	-41,97	-2,518	0,113909	-0,16	-60,73	-44,48	2,91762

				4	LOF_2	44	137	66		3	02	05		18	49	83	
BTzPhOMe6	438	Done	51,59	-4,28302	glide-grid_5LOF_2	-5,91892	-1,50866	-0,35582	0	0	-46,5916	-11,3545	0,241209	-0,26289	-77,8679	-57,9462	14,0046
BTzPhOMe6	441	Done	54,68	-4,25664	glide-grid_5LOF_2	-6,25854	-2,69359	-0,12436	0	-0,49734	-57,8607	-1,70411	0,240388	-0,03498	-77,9726	-59,5648	11,9987
BTzPhDMN9	388	Done	56,9	-4,23551	glide-grid_5LOF_2	-6,74461	-2,98075	-0,25254	0	-0,70769	-49,3422	-2,69538	0,260944	-0,19317	-75,7301	-52,0375	7,64121
BTzPhDMN7	371	Done	38,63	-4,21763	glide-grid_5LOF_2	-5,26403	-1,1907	-0,37256	0	-0,13968	-44,5747	-9,32915	0,235707	-0,16869	-72,4049	-53,9039	5,59486
BTzNaf4	162	Done	25,18	-4,2079	glide-grid_5LOF_2	-6,5852	-3,12928	0	0	-0,95486	-59,1734	-1,88669	0,174997	-0,00039	-82,4792	-57,2867	6,54881
BTzPhDMN3	343	Done	36,68	-4,19193	glide-grid_5LOF_2	-6,55693	-2,07064	-0,46789	0	-0,68217	-45,1396	-7,40655	0,164133	-0,13241	-73,5787	-52,5462	8,68329
BTzPh3	225	Done	35,63	-4,18596	glide-grid_5LOF_2	-6,67376	-2,58356	-0,3024	0	-0,63176	-52,7436	-4,08753	0,238112	-0,14384	-81,266	-56,8312	9,74648
BTzPhOMe1	397	Done	19,44	-4,18402	glide-grid_5LOF_2	-6,15842	-2,4534	-0,14176	0	-0,7865	-51,0707	-1,88386	0,132423	-0,07307	-64,2364	-52,9545	17,1481
BTz5	100	Done	33,77	-4,17668	glide-grid_5LOF_2	-6,54168	-2,76427	-0,4727	0	-0,14353	-42,291	-8,51501	0,351423	-0,12082	-67,2863	-50,806	12,8097
BTz3	85	Done	36,42	-4,17013	glide-grid_5LOF_2	-6,55903	-2,24956	-0,55033	0	-0,10949	-33,1298	-13,7076	0,298207	-0,23522	-64,3011	-46,8374	5,573
BTzPhDMN4	354	Done	32,01	-4,1616	glide-grid_5	-6,827	-2,38	-0,398	0	-0,519	-50,65	-6,847	0,184456	-0,14	-76,83	-57,50	8,62843

				6	LOF_2	86	458	78		53	98	03		939	91	68	
BTzPh8	258	Done	38,9	-<br>4,1612<br>4	glide-<br>grid_5<br>LOF_2	-<br>4,721<br>94	-<br>0,55<br>898	-<br>0,318<br>28	0	-<br>0,069<br>76	-<br>41,32<br>54	-<br>12,38<br>33	0,323<br>344	-<br>0,17<br>45	-<br>69,46<br>01	-<br>53,70<br>87	12,208<br>1
BTzNaf5	170	Done	34,48	-<br>4,1515<br>6	glide-<br>grid_5<br>LOF_2	-<br>6,590<br>86	-<br>3,05<br>862	-<br>0,126<br>74	0	-<br>0,136<br>31	-<br>57,67<br>34	-<br>1,879<br>19	0,193<br>712	-<br>0,29<br>736	-<br>84,81<br>1	-<br>59,55<br>26	7,6899<br>4
BTzPhO<br>Me8	456	Done	52,92	-<br>4,1509<br>5	glide-<br>grid_5<br>LOF_2	-<br>5,786<br>85	-<br>1,19<br>305	-<br>0,500<br>77	0	-<br>0,290<br>29	-<br>47,19<br>01	-<br>10,74<br>87	0,269<br>328	-<br>0,10<br>027	-<br>75,79<br>04	-<br>57,93<br>87	13,372<br>1
BTzPhO<br>Me3	415	Done	30,51	-<br>4,1499<br>4	glide-<br>grid_5<br>LOF_2	-<br>6,752<br>44	-<br>2,62<br>528	-<br>0,458<br>38	0	-<br>0,604<br>68	-<br>44,47<br>42	-<br>5,820<br>15	0,183<br>449	-<br>0,15<br>081	-<br>71,99<br>47	-<br>50,29<br>44	6,0056<br>7
BTzPhD<br>MN2	337	Done	22,23	-<br>4,1450<br>9	glide-<br>grid_5<br>LOF_2	-<br>6,609<br>99	-<br>2,96<br>545	-0,16	0	-<br>0,377<br>51	-<br>49,47<br>24	-<br>4,783<br>86	0,140<br>48	-<br>0,05<br>631	-<br>76,65<br>53	-<br>54,25<br>63	5,7742<br>5
BTzPhCl<br>8	318	Done	39,42	-<br>4,1429<br>3	glide-<br>grid_5<br>LOF_2	-<br>6,689<br>23	-<br>2,52<br>97	-<br>0,243<br>32	0	-<br>0,825<br>2	-<br>48,35<br>29	-<br>5,697<br>05	0,262<br>284	-<br>0,08<br>11	-<br>68,41<br>88	-54,05	24,853<br>8
BTzPh6	248	Done	34,18	-<br>4,1413<br>6	glide-<br>grid_5<br>LOF_2	-<br>6,673<br>46	-<br>2,19<br>81	-<br>0,524<br>04	0	-<br>0,765<br>89	-<br>53,14<br>05	-<br>5,212<br>28	0,294<br>136	-<br>0,04<br>07	-81,39	-<br>58,35<br>28	4,7781<br>5
BTzNaf6	179	Done	46,04	-<br>4,1328<br>1	glide-<br>grid_5<br>LOF_2	-<br>6,664<br>91	-<br>3,00<br>226	-<br>0,325<br>57	0	-<br>0,260<br>73	-<br>53,49<br>71	-<br>3,364<br>16	0,209<br>716	-<br>0,10<br>659	-<br>78,50<br>72	-<br>56,86<br>13	7,3694<br>7
BTzPhD<br>MN1	328	Done	15,11	-<br>4,1308<br>9	glide-<br>grid_5<br>LOF_2	-<br>5,387<br>69	-<br>1,97<br>748	0	0	-<br>0,621<br>35	-<br>44,24<br>2	-<br>4,224<br>57	0,113<br>2	-<br>0,05<br>627	-<br>59,37<br>34	-<br>48,46<br>65	9,9461<br>9
BTzPhO<br>Me8	457	Done	66,66	-<br>4,1166<br>9	glide-<br>grid_5<br>LOF_2	-<br>5,752<br>59	-<br>2,52<br>885	-<br>0,204<br>5	0	0	-<br>44,47<br>19	-<br>5,264<br>25	0,269<br>328	-<br>0,27<br>534	-<br>65,88<br>91	-<br>49,73<br>61	11,081<br>8
BTz3	87	Done	29,1	-<br>4,1037	glide-<br>grid_5	-<br>6,624	-<br>2,61	-<br>0,531	0	-<br>0,313	-<br>40,81	-<br>9,271	0,298<br>207	-<br>0,03	-<br>65,47	-<br>50,08	10,131<br>9

				1	LOF_2	61	086	95		5	28	36		516	33	42	
BTz4	91	Done	44,04	-4,10346	glide-grid_5LOF_2	-5,71046	-2,95977	0	0	-0,72532	-39,3472	-1,20689	0,329799	-0,20677	-62,8587	-40,5541	4,7954
BTzNaf1	141	Done	15,54	-4,09375	glide-grid_5LOF_2	-6,44055	-2,76811	-0,29505	0	-0,28907	-51,0628	-3,93236	0,103626	-0,04895	-73,7244	-54,9952	7,44244
BTzPhOMe7	445	Done	52,94	-4,08437	glide-grid_5LOF_2	-5,09847	-0,74163	-0,78944	0	-0,04633	-34,8264	-12,5857	0,256104	-0,14801	-63,3974	-47,4121	6,76346
BTzPhOMe9	469	Done	73,72	-4,07393	glide-grid_5LOF_2	-6,69763	-3,43479	-0,02219	0	-0,40501	-56,6968	-1,78729	0,280258	-0,01297	-76,1404	-58,4841	17,5699
BTzPh4	232	Done	25,9	-4,07177	glide-grid_5LOF_2	-6,53017	-3,10003	-0,16	0	-0,66548	-45,0576	-2,13494	0,25966	-0,29121	-71,1566	-47,1925	5,10256
BTzPh3	224	Done	32,93	-4,06111	glide-grid_5LOF_2	-6,54191	-2,14526	-0,07585	0	-1,36759	-57,267	-1,74229	0,237111	-0,06563	-78,713	-59,0092	8,75961
BTzPhDMN8	379	Done	60,33	-4,05607	glide-grid_5LOF_2	-5,10827	-1,38605	-0,34361	0	-0,61889	-41,5452	-5,61147	0,248293	-0,08904	-61,7198	-47,1567	4,43935
BTzPhOMe1	392	Done	17,15	-4,05101	glide-grid_5LOF_2	-5,11821	-2,15341	-0,14799	0	-0,32403	-41,1123	-2,78583	0,133203	-0,1525	-59,1469	-43,8981	4,08975
BTzPhOMe3	413	Done	39,58	-4,03782	glide-grid_5LOF_2	-6,00552	-2,25292	-0,30173	0	-0,717	-47,3769	-2,98247	0,183449	-0,10111	-67,171	-50,3594	6,38941
BTz3	82	Done	34,91	-4,03326	glide-grid_5LOF_2	-4,98266	-1,6543	-0,35415	0	-0,62376	-38,6404	-4,68747	0,299653	-0,01496	-54,038	-43,3279	9,49122
BTz2	79	Done	31,68	-4,033	glide-grid_5	-6,567	-2,83	-0,32	0	-0,489	-38,49	-7,458	0,264611	-0,14	-60,51	-45,95	9,36386

					LOF_2		075			46	51	83		782	27	39	
BTz1	71	Done	20,06	-4,02613	glide-grid_5LOF_2	-6,49903	-2,75498	-0,47708	0	-0,30428	-33,3911	-9,62564	0,225258	-0,07455	-55,9141	-43,0167	7,08502
BTzPhCl1	278	Done	16,92	-4,02082	glide-grid_5LOF_2	-6,50692	-3,47419	-0,14195	0	-0,12406	-39,197	-4,63868	0,126457	-0,23753	-64,8303	-43,8357	7,54165
BTzPhDMN1	329	Done	16,94	-4,015	glide-grid_5LOF_2	-6,3197	-2,987	-0,09037	0	-0,63776	-49,6263	-1,08267	0,113909	-0,07477	-68,7789	-50,709	10,7173
BTzPhDMN4	351	Done	30,77	-3,99819	glide-grid_5LOF_2	-6,37549	-2,2221	-0,5882	0	-0,36084	-41,2434	-7,53991	0,185208	-0,19641	-70,0926	-48,7833	4,97966
BTzNaf8	193	Done	47,07	-3,99704	glide-grid_5LOF_2	-6,37754	-3,07873	0	0	-0,32815	-53,9362	-1,92771	0,238783	-0,22348	-81,3741	-55,8639	8,57696
BTz2	78	Done	27,41	-3,98285	glide-grid_5LOF_2	-6,49065	-2,658	-0,608	0	-0,13237	-35,5018	-9,78592	0,264611	-0,11391	-61,3282	-45,2878	6,3366
BTz3	84	Done	37,63	-3,98144	glide-grid_5LOF_2	-6,36894	-2,55722	-0,3196	0	-0,323	-40,3654	-8,75715	0,298207	-0,13549	-66,988	-49,1225	8,48487
BTz3	81	Done	37,02	-3,97753	glide-grid_5LOF_2	-4,90803	-1,78468	-0,27595	0	-0,45654	-34,5734	-5,06048	0,299653	-0,20277	-54,9007	-39,6339	4,40636
BTzPhDMN8	376	Done	50,72	-3,97683	glide-grid_5LOF_2	-4,59733	-0,86131	-0,29585	0	-0,5174	-43,4061	-5,12831	0,249802	-0,23303	-64,2912	-48,5344	6,87838
BTzPhOMe1	394	Done	20,37	-3,95119	glide-grid_5LOF_2	-5,49429	-2,02643	-0,18809	0	-0,01436	-41,9168	-7,83455	0,133203	-0,12759	-64,3653	-49,7514	12,9317
BTz8	123	Done	38,42	-3,9310	glide-grid_5	-5,531	-1,48	-0,474	0	0	-27,55	-14,18	0,408139	-0,47	-62,46	-41,74	7,80565

				3	LOF_2	43	355	91			74	92		487	14	65	
BTzPhOMe8	453	Done	63,4	-3,89861	glide-grid_5LOF_2	-4,91271	-1,60124	-0,19066	0	0	-46,2131	-5,23334	0,269328	-0,29449	-66,6606	-51,4464	9,71943
BTz6	110	Done	40,19	-3,8903	glide-grid_5LOF_2	-6,4343	-1,9927	-0,28905	0	-1,38144	-52,3074	-3,47701	0,372355	-0,00653	-72,4488	-55,7844	11,3815
BTzPh4	234	Done	22,58	-3,86698	glide-grid_5LOF_2	-6,53318	-2,64545	-0,25955	0	-1,11845	-54,8205	-0,18243	0,258658	0	-74,3304	-55,003	4,31893
BTzPhOMe4	419	Done	33,16	-3,86226	glide-grid_5LOF_2	-5,22956	-1,0688	-0,63785	0	-0,09735	-43,1859	-9,28723	0,204725	-0,0779	-67,346	-52,4731	6,48897
BTzPh9	272	Done	49,57	-3,85924	glide-grid_5LOF_2	-6,39134	-2,52186	-0,15574	0	-0,77956	-56,0205	-2,71213	0,332533	-0,05887	-76,9308	-58,7326	8,99719
BTz6	108	Done	38,71	-3,8587	glide-grid_5LOF_2	-6,2164	-1,78282	-0,39315	0	-1,48691	-48,0463	-3,41232	0,372355	-0,0117	-69,7932	-51,4586	4,02488
BTzPhDMN3	345	Done	27,9	-3,85852	glide-grid_5LOF_2	-6,34632	-2,30652	-0,38109	0	-0,39134	-45,2188	-7,26927	0,164133	-0,08017	-72,8477	-52,488	7,76495
BTzPh1	210	Done	17,09	-3,84325	glide-grid_5LOF_2	-6,19005	-2,53497	-0,05987	0	-0,82268	-51,1907	-2,32527	0,184509	-0,04872	-71,6542	-53,516	3,76941
BTzPh2	217	Done	22,49	-3,83294	glide-grid_5LOF_2	-6,29784	-2,33	-0,22581	0	-0,79719	-51,5996	-3,04804	0,213536	-0,12119	-79,7896	-54,6477	2,83611
BTz1	70	Done	21,45	-3,83135	glide-grid_5LOF_2	-6,18795	-2,50622	-0,66632	0	-0,26695	-36,7779	-7,1888	0,225258	-0,0565	-56,6792	-43,9667	9,62247
BTzPhDMN8	378	Done	52,82	-3,8293	glide-grid_5	-4,875	-1,19	-0,028	0	-0,291	-49,15	-5,781	0,249046	-0,28	-71,45	-54,93	9,07315

				9	LOF_2	79	811	17		91	6	65		16	24	77	
BTzPhO<br>Me4	422	Done	40,07	-<br>3,8120<br>5	glide-<br>grid_5<br>LOF_2	-<br>5,806<br>05	-<br>2,47<br>448	-<br>0,183<br>11	0	-<br>0,017<br>56	-<br>53,01<br>3	-<br>4,093<br>84	0,204<br>725	-<br>0,07<br>09	-<br>75,10<br>83	-<br>57,10<br>68	7,5831<br>8
BTzNaf9	204	Done	50,94	-<br>3,8076<br>9	glide-<br>grid_5<br>LOF_2	-<br>6,480<br>29	-<br>3,34<br>845	0	0	-<br>0,601<br>61	-<br>55,07<br>81	-<br>0,107<br>2	0,250<br>027	-<br>0,01<br>026	-<br>76,51<br>86	-<br>55,18<br>53	5,3593<br>1
BTzPhD<br>MN5	360	Done	36,89	-<br>3,7989<br>6	glide-<br>grid_5<br>LOF_2	-<br>6,313<br>16	-<br>1,93<br>646	-<br>0,587<br>52	0	-<br>0,622<br>18	-<br>45,65<br>83	-<br>5,004<br>49	0,203<br>997	-<br>0,33<br>741	-<br>73,91<br>17	-<br>50,66<br>28	5,4219
BTzPhD<br>MN3	344	Done	27,87	-<br>3,7876<br>7	glide-<br>grid_5<br>LOF_2	-<br>6,268<br>47	-<br>2,06<br>544	-<br>0,462<br>96	0	-<br>1,143<br>63	-<br>41,85<br>8	-<br>3,760<br>3	0,163<br>391	-<br>0,10<br>29	-<br>65,61<br>38	-<br>45,61<br>83	1,5456<br>6
BTz7	119	Done	38,61	-<br>3,7809<br>3	glide-<br>grid_5<br>LOF_2	-<br>6,325<br>93	-<br>2,22<br>314	-<br>0,472<br>06	0	-<br>0,998<br>73	-<br>43,01<br>32	-<br>5,508<br>36	0,390<br>204	-<br>0,04<br>529	-<br>66,11<br>49	-<br>48,52<br>16	7,0464<br>2
BTz4	93	Done	40,29	-<br>3,7781<br>7	glide-<br>grid_5<br>LOF_2	-<br>6,151<br>07	-<br>2,70<br>333	0	0	-<br>1,293<br>8	-<br>47,04<br>06	-<br>0,709<br>45	0,326<br>906	-<br>0,02<br>24	-<br>62,62<br>56	-<br>47,75<br>01	8,8589<br>2
BTzPh5	240	Done	40,48	-<br>3,7751<br>2	glide-<br>grid_5<br>LOF_2	-<br>6,289<br>32	-<br>2,23<br>216	-<br>0,189<br>85	0	-<br>0,854<br>73	-<br>42,08<br>42	-<br>6,177<br>43	0,278<br>558	-<br>0,26<br>032	-<br>64,15<br>8	-<br>48,26<br>17	17,296<br>3
BTzPhCl<br>9	323	Done	46,37	-<br>3,7698<br>4	glide-<br>grid_5<br>LOF_2	-<br>6,316<br>14	-<br>1,82<br>192	-<br>0,503<br>76	0	-<br>0,458<br>69	-<br>49,79<br>84	-<br>7,038<br>96	0,274<br>081	-<br>0,26<br>009	-<br>78,05<br>38	-<br>56,83<br>74	8,5901<br>5
BTzPhCl<br>4	294	Done	35,89	-<br>3,7676	glide-<br>grid_5<br>LOF_2	-<br>6,320<br>2	-<br>2,67<br>645	-0,16	0	-<br>0,621<br>54	-<br>44,18<br>49	-<br>3,781<br>81	0,198<br>458	-<br>0,28<br>415	-<br>71,11<br>64	-<br>47,96<br>67	4,3457
BTzPh7	254	Done	38,6	-<br>3,7638<br>1	glide-<br>grid_5<br>LOF_2	-<br>6,144<br>31	-<br>2,33<br>69	-<br>0,118<br>48	0	-<br>1,177<br>26	-<br>49,93<br>44	-<br>1,464<br>69	0,309<br>652	-<br>0,10<br>489	-<br>74,79<br>32	-<br>51,39<br>91	3,7458<br>6
BTzNaf6	178	Done	43,07	-<br>3,7581	glide-<br>grid_5	-<br>6,208	-<br>2,15	-<br>0,419	0	-<br>0,134	-<br>44,43	-<br>9,017	0,210<br>443	-<br>0,13	-<br>76,00	-<br>53,45	4,4285<br>1

				5	LOF_2	85	37	88		6	55	55		671	86	3	
BTzNaf7	185	Done	40,47	-3,75347	glide-grid_5LOF_2	-6,13397	-3,03992	0	0	-0,66143	-50,2711	-0,74437	0,225404	-0,03281	-75,0591	-51,0155	3,16586
BTzPhDMN7	369	Done	53,48	-3,73601	glide-grid_5LOF_2	-4,35651	-0,89089	-0,03966	0	-0,08447	-42,5807	-7,74579	0,236467	-0,28705	-65,3821	-50,3264	9,6948
BTzPhOMe8	455	Done	51,5	-3,73311	glide-grid_5LOF_2	-5,11331	-1,03238	-0,32	0	-0,18078	-47,6196	-8,70916	0,268515	-0,16131	-67,1464	-56,3287	16,2873
BTzPhOMe8	459	Done	52,2	-3,72524	glide-grid_5LOF_2	-5,72714	-2,13527	-0,46348	0	-0,44534	-48,2603	-3,59031	0,268515	0	-63,285	-51,8506	14,6817
BTzPhOMe1	398	Done	19,51	-3,722	glide-grid_5LOF_2	-6,276	-2,63957	-0,377	0	0	-48,2595	-5,52522	0,132423	-0,15011	-65,0914	-53,7847	17,6181
BTzPhDMN2	334	Done	28,04	-3,71244	glide-grid_5LOF_2	-4,57034	-1,35964	0	0	-0,47615	-41,9355	-3,5854	0,14048	-0,24045	-61,5029	-45,5209	1,96833
BTz3	86	Done	37,59	-3,69826	glide-grid_5LOF_2	-6,21196	-2,58017	-0,60527	0	-0,19761	-40,7696	-6,6157	0,298207	-0,09628	-63,1264	-47,3853	8,22243
BTzPh1	209	Done	21,24	-3,691	glide-grid_5LOF_2	-5,9957	-2,52453	0	0	-1,40379	-46,0293	-0,323972	0,18549	0	-63,0174	-45,7054	5,66368
BTzPh3	226	Done	28,54	-3,68902	glide-grid_5LOF_2	-6,36822	-2,35949	-0,44379	0	-0,65773	-45,4748	-4,44245	0,237111	-0,20422	-65,1318	-49,9173	10,4612
BTz5	98	Done	38,86	-3,68247	glide-grid_5LOF_2	-4,62017	-1,08612	-0,32	0	-0,06283	-34,3866	-10,465	0,352856	-0,215	-57,7469	-44,8516	8,39262
BTzPhCl6	305	Done	39,16	-3,6746	glide-grid_5	-6,220	-2,46	-0,271	0	-0,048	-46,69	-8,252	0,234095	-0,10	-75,63	-54,95	8,7447

				7	LOF_2	97	04	99		22	85	4		167	1	09	
BTzPhOMe9	464	Done	80,97	-3,67396	glide-grid_5LOF_2	-5,05416	-1,42972	-0,16	0	-0,1157	-50,1322	-6,46901	0,280258	-0,15204	-72,3511	-56,6012	6,42473
BTz5	103	Done	31,95	-3,66158	glide-grid_5LOF_2	-6,21478	-2,69108	-0,32	0	-0,42347	-36,3883	-7,94242	0,351423	-0,12087	-59,4783	-44,3307	7,80013
BTzPhDMN9	385	Done	53,2	-3,64789	glide-grid_5LOF_2	-4,69429	-1,24641	-0,43719	0	-0,22596	-37,9058	-5,52406	0,260944	-0,32178	-57,6133	-43,4299	6,09337
BTz8	126	Done	43,64	-3,63579	glide-grid_5LOF_2	-6,18079	-2,1046	-0,17304	0	-1,35876	-54,4229	-1,46676	0,405393	-0,00862	-70,6567	-55,8896	13,5746
BTz7	115	Done	37,13	-3,6344	glide-grid_5LOF_2	-5,2348	-1,73647	-0,30162	0	-0,1113	-28,919	-11,7311	0,393	-0,27279	-59,7617	-40,6501	6,84017
BTz5	99	Done	40,13	-3,62028	glide-grid_5LOF_2	-5,20398	-2,07571	-0,15615	0	-0,17485	-38,4761	-5,57191	0,354295	-0,39198	-62,0131	-44,048	8,95319
BTz1	72	Done	24,14	-3,61873	glide-grid_5LOF_2	-6,20453	-2,3118	-0,43964	0	-0,47105	-36,5649	-8,70856	0,225258	-0,07275	-58,8336	-45,2735	9,12176
BTzPhDMN5	358	Done	40,85	-3,61725	glide-grid_5LOF_2	-4,68115	-0,66956	-0,85259	0	-0,136	-42,6391	-6,5593	0,203241	-0,11039	-62,2971	-49,1984	3,59966
BTzPhDMN1	330	Done	16,24	-3,61689	glide-grid_5LOF_2	-5,96369	-2,14084	-0,24995	0	-0,62106	-41,6852	-5,60008	0,1132	-0,14077	-59,884	-47,2853	9,1071
BTzNaf8	194	Done	58,43	-3,60694	glide-grid_5LOF_2	-6,05764	-1,27237	-0,19069	0	-0,99615	-55,8035	-6,28774	0,238783	-0,10387	-83,4324	-62,0913	14,0963
BTzNaf9	202	Done	51,17	-3,6024	glide-grid_5	-6,053	-2,49	-0,006	0	-0,696	-56,56	-0,419	0,250747	-0,21	-78,13	-56,98	9,21115

				3	LOF_2	13	298	88		52	38	39		64		32	
BTzPh6	246	Done	31,45	-3,59734	glide-grid_5LOF_2	-5,97784	-2,58782	-0,16	0	-0,441	-42,0453	-5,76576	0,295128	-0,11703	-67,6542	-47,811	3,79964
BTzPh8	263	Done	37,41	-3,55462	glide-grid_5LOF_2	-6,00532	-2,51005	-0,16	0	-0,45168	-47,6165	-4,64337	0,322369	-0,12863	-73,156	-52,2599	6,82596
BTzPhDMN9	389	Done	57,74	-3,54497	glide-grid_5LOF_2	-6,12517	-1,84606	-0,11887	0	-1,16074	-63,9995	-0,34969	0,260195	-0,00727	-81,9906	-64,3492	12,1273
BTz1	73	Done	21,95	-3,5416	glide-grid_5LOF_2	-6,1766	-2,6319	-0,38901	0	-0,16756	-36,818	-7,68553	0,225258	-0,21966	-55,6381	-44,5036	14,4368
BTzPh1	211	Done	19,03	-3,5363	glide-grid_5LOF_2	-6,1335	-2,81249	-0,22191	0	-0,31629	-45,8528	-3,50527	0,18549	-0,14987	-69,9782	-49,3581	6,92527
BTzPhCl9	321	Done	44,35	-3,52044	glide-grid_5LOF_2	-4,66614	-1,59274	-0,00916	0	-0,1588	-49,7106	-2,77561	0,274081	-0,27766	-69,4749	-52,4862	5,24439
BTzPhOMe8	458	Done	53,8	-3,52009	glide-grid_5LOF_2	-5,52199	-1,9319	-0,16	0	-0,33692	-46,4996	-5,7167	0,268515	-0,17921	-66,3737	-52,2163	8,89303
BTz7	116	Done	30,57	-3,51374	glide-grid_5LOF_2	-5,86864	-1,35318	-0,13559	0	-1,10613	-43,2094	-8,84851	0,390204	-0,1762	-65,376	-52,0579	13,484
BTz7	114	Done	35	-3,50685	glide-grid_5LOF_2	-4,45015	-0,6542	-0,47411	0	-0,5973	-35,3522	-7,76025	0,391599	-0,18451	-57,8091	-43,1124	3,82444
BTzPh8	265	Done	42,79	-3,50288	glide-grid_5LOF_2	-6,17548	-2,57108	-0,24968	0	-0,73971	-53,6639	-1,21861	0,321398	-0,07043	-72,87	-54,8825	6,93158
BTz8	125	Done	41,81	-3,4989	glide-grid_5	-5,853	-1,98	-0,060	0	-1,397	-52,39	-1,182	0,405393	-0,01	-70,52	-53,57	7,55192

				1	LOF_2	71	814	41		88	05	24		58	76	28	
BTzPhOMe1	396	Done	17,57	-3,45737	glide-grid_5LOF_2	-5,29077	-2,38212	0	0	-0,26078	-46,9508	-2,56896	0,132423	-0,0474	-59,3806	-49,5197	12,5055
BTz9	131	Done	42,38	-3,4508	glide-grid_5LOF_2	-5,0512	-1,69193	-0,33194	0	-0,17416	-39,2199	-6,90554	0,420972	-0,27732	-64,6922	-46,1255	7,34373
BTzPhDMN5	361	Done	36,13	-3,44842	glide-grid_5LOF_2	-6,04372	-2,39588	-0,23336	0	-1,02993	-41,6706	-2,7799	0,203241	-0,08727	-62,6236	-44,4505	2,0998
BTzPhCl7	311	Done	37,74	-3,43565	glide-grid_5LOF_2	-5,98195	-2,17224	-0,1093	0	-0,68543	-43,605	-5,33833	0,249015	-0,28299	-70,8505	-48,9434	4,11421
BTzPhOMe9	461	Done	70,68	-3,43196	glide-grid_5LOF_2	-4,08006	-0,82669	-0,13645	0	-0,03461	-42,956	-5,72857	0,28187	-0,35711	-63,5933	-48,6845	9,66141
BTzPhOMe9	466	Done	70,39	-3,41188	glide-grid_5LOF_2	-5,04778	-1,78526	-0,00798	0	-0,27629	-51,1786	-3,71708	0,281063	-0,14282	-67,096	-54,8956	16,2898
BTz4	95	Done	40,84	-3,34703	glide-grid_5LOF_2	-5,88863	-1,67763	-0,32453	0	-1,32675	-45,2143	-4,14029	0,326906	-0,00486	-60,7141	-49,3546	14,1513
BTzNaf3	155	Done	30,5	-3,33406	glide-grid_5LOF_2	-5,81486	-2,54505	-0,16709	0	-0,10665	-51,4725	-3,76947	0,153343	-0,01037	-73,4773	-55,242	4,90999
BTzNaf9	203	Done	43,42	-3,33213	glide-grid_5LOF_2	-5,86423	-2,15858	-0,32	0	-0,30416	-52,3694	-4,43831	0,250027	-0,0473	-72,2868	-56,8078	12,3029
BTzPhDMN6	368	Done	40,92	-3,32895	glide-grid_5LOF_2	-5,90915	-1,3358	-0,59157	0	-0,35288	-53,2913	-7,33309	0,219998	-0,08438	-77,1403	-60,6244	10,024
BTzNaf4	164	Done	27,73	-3,3285	glide-grid_5	-5,851	-2,14	0	0	-0,632	-58,14	-2,272	0,174279	0	-75,92	-60,42	12,1013

				4	LOF_2	94	587			04	77	84			81	05	
BTz6	109	Done	42,25	-3,31946	glide-grid_5LOF_2	-5,67716	-1,40709	-0,23169	0	-1,29173	-50,2623	-3,91281	0,372355	-0,01897	-68,8369	-54,1751	8,38155
BTzPhOMe6	437	Done	62,31	-3,28954	glide-grid_5LOF_2	-4,66974	-0,99829	-0,41979	0	0	-44,3683	-6,93463	0,240388	-0,23345	-64,2221	-51,3029	8,82065
BTz5	101	Done	32,43	-3,27982	glide-grid_5LOF_2	-5,64622	-1,7153	-0,34217	0	-0,66104	-39,4842	-7,91332	0,351423	-0,11792	-58,3658	-47,3975	10,5657
BTzPhDMN8	381	Done	49,34	-3,23357	glide-grid_5LOF_2	-5,74267	-2,13645	-0,005	0	-0,26745	-48,5327	-6,43564	0,249046	-0,19083	-73,8884	-54,9683	10,5503
BTz5	102	Done	39,13	-3,22301	glide-grid_5LOF_2	-5,77401	-1,15947	-0,58807	0	-1,22432	-45,7681	-5,21262	0,351423	-0,08328	-66,3957	-50,9807	9,48227
BTzPh2	218	Done	28,64	-3,18884	glide-grid_5LOF_2	-5,65954	-2,43187	-0,16	0	-0,03405	-36,2703	-7,93261	0,213536	-0,24375	-64,4068	-44,2029	2,19153
BTzPh4	231	Done	21,36	-3,17936	glide-grid_5LOF_2	-5,55666	-2,58478	0	0	-0,80319	-47,4939	-0,481017	0,25966	-0,12581	-65,6016	-47,0129	4,16434
BTzPhCl6	306	Done	40,76	-3,1273	glide-grid_5LOF_2	-5,6736	-2,24956	-0,21225	0	-0,86866	-52,3898	-0,307995	0,234095	-0,00394	-74,2364	-52,0818	2,20075
BTzPhOMe5	433	Done	35,53	-3,11516	glide-grid_5LOF_2	-5,75716	-2,51955	-0,13497	0	-0,24307	-50,1901	-3,40754	0,223613	-0,06254	-71,0154	-53,5977	7,38384
BTzPhOMe7	451	Done	46,24	-3,09359	glide-grid_5LOF_2	-5,71729	-2,16306	-0,45177	0	-0,03267	-49,9971	-4,90276	0,255286	-0,08981	-68,7671	-54,8999	13,2151
BTzPhOMe7	450	Done	51,79	-3,0773	glide-grid_5	-5,079	-1,55	-0,444	0	-0,035	-42,95	-7,085	0,255286	-0,08	-60,96	-50,04	12,6011

				1	LOF_2	21	861	47		48	88	55		516	12	44	
BTzPhOMe5	431	Done	37,5	-3,07098	glide-grid_5LOF_2	-5,08748	-1,65624	-0,25815	0	-0,0669	-49,1889	-5,36082	0,223613	-0,06623	-67,7686	-54,5497	11,1838
BTz8	124	Done	31,07	-3,07031	glide-grid_5LOF_2	-5,42511	-2,16174	-0,32	0	-0,11687	-38,14	-8,15359	0,405393	-0,10185	-57,6997	-46,2936	9,46704
BTzPhDMN7	375	Done	47,64	-3,03866	glide-grid_5LOF_2	-5,61886	-1,71771	-0,26153	0	-0,74134	-51,7358	-3,17679	0,23495	-0,06993	-70,5788	-54,9126	6,04598
BTz6	105	Done	35,66	-3,03543	glide-grid_5LOF_2	-3,97763	-0,76922	0	0	-1,21407	-44,109	-0,13368	0,373771	-0,14261	-55,4826	-44,2427	7,78317
BTzPh7	256	Done	34,64	-3,01915	glide-grid_5LOF_2	-5,55125	-2,43265	0	0	-0,6513	-51,9798	-0,94487	0,308669	-0,03525	-72,4291	-52,9247	4,76427
BTz7	117	Done	35,04	-3,00133	glide-grid_5LOF_2	-5,35623	-1,31406	-0,05128	0	-1,1884	-46,5988	-4,14782	0,390204	-0,24058	-65,6865	-50,7466	8,60082
BTzPhDMN2	338	Done	21,78	-2,98201	glide-grid_5LOF_2	-5,45271	-1,64427	-0,31823	0	-0,48468	-37,7081	-6,03104	0,14048	-0,35596	-59,7872	-43,7392	5,68685
BTzPhDMN8	380	Done	52,4	-2,94906	glide-grid_5LOF_2	-5,38346	-1,07778	-0,49934	0	-0,10514	-35,4569	-13,4305	0,249046	-0,16282	-66,7483	-48,8874	4,31132
BTz9	135	Done	48,72	-2,90988	glide-grid_5LOF_2	-5,45488	-1,50575	-0,56373	0	-0,02213	-39,9506	-11,1009	0,41828	-0,11888	-66,6542	-51,0516	7,57736
BTzPhDMN4	353	Done	23,78	-2,88513	glide-grid_5LOF_2	-5,40853	-1,82659	-0,34974	0	-0,44343	-44,1177	-4,30687	0,184456	-0,12132	-62,7204	-48,4246	5,87131
BTzPhDMN4	352	Done	31,04	-2,8508	glide-grid_5	-5,309	-1,78	-0,282	0	-0,326	-43,71	-5,192	0,185208	-0,14	-65,62	-48,90	6,24173

				9	LOF_2	29	031	38		61	08	24		083	73	3	
BTzPhOMe8	460	Done	63,01	-2,84618	glide-grid_5LOF_2	-5,46988	-2,21227	-0,05478	0	-0,3965	-55,2416	-1,11959	0,268515	-0,14482	-67,8292	-56,3612	15,7737
BTzPhDMN6	366	Done	41,96	-2,8438	glide-grid_5LOF_2	-5,2782	-1,91774	-0,06322	0	-0,31497	-51,0979	-3,71223	0,220756	-0,0913	-70,4997	-54,8101	8,25618
BTz1	69	Done	22,81	-2,83952	glide-grid_5LOF_2	-4,54932	-1,7544	-0,24853	0	-0,17959	-29,9116	-5,77066	0,228099	-0,23371	-46,6474	-35,6823	11,9507
BTzPhOMe9	467	Done	68,18	-2,78472	glide-grid_5LOF_2	-4,78662	-0,96186	-0,48	0	-0,3772	-43,8143	-6,6749	0,280258	-0,05587	-62,1499	-50,4892	9,10689
BTz9	132	Done	41,82	-2,77895	glide-grid_5LOF_2	-5,13375	-1,55896	-0,38829	0	-0,31495	-37,6552	-8,16427	0,41828	-0,18243	-56,2479	-45,8195	11,0789
BTz3	83	Done	39,65	-2,71753	glide-grid_5LOF_2	-4,34953	-0,73439	-0,32	0	-0,594	-33,64	-8,01243	0,301106	-0,11838	-56,4682	-41,6524	7,71698
BTz9	134	Done	41,37	-2,64655	glide-grid_5LOF_2	-5,19155	-1,24592	-0,26982	0	-0,05002	-32,9466	-14,5485	0,41828	-0,21447	-60,2527	-47,4951	9,86174
BTzPhDMN7	373	Done	50,85	-2,62165	glide-grid_5LOF_2	-5,05605	-0,78682	-0,59169	0	-0,01293	-37,6397	-11,923	0,235707	-0,22988	-68,0414	-49,5627	3,92024
BTzPhCl3	288	Done	30,05	-2,56777	glide-grid_5LOF_2	-5,12177	-1,86466	-0,17077	0	-0,04631	-41,7869	-6,04289	0,17724	-0,2215	-62,1597	-47,8298	9,34952
BTzPhOMe9	465	Done	62,37	-2,56439	glide-grid_5LOF_2	-4,20029	-1,18118	-0,16	0	-0,27831	-41,4751	-4,38447	0,281063	-0,13045	-61,4018	-45,8596	4,22876
BTz6	111	Done	39,76	-2,5467	glide-grid_5	-5,090	-0,91	-0,64	0	-1,008	-46,54	-3,408	0,372355	-0,06	-61,13	-49,95	9,3314

				7	LOF_2	77	322			5	14	6		304	52		
BTzPhD<br>MN1	332	Done	24,67	-<br>2,5404<br>2	glide-<br>grid_5<br>LOF_2	-<br>5,167<br>52	-<br>1,63<br>391	-<br>0,159<br>26	0	-<br>0,061<br>54	-<br>52,19<br>39	-<br>4,713<br>67	0,113<br>2	-<br>0,10<br>926	-<br>73,28<br>86	-<br>56,90<br>76	5,9199
BTzPh9	271	Done	45,49	-<br>2,4864	glide-<br>grid_5<br>LOF_2	-<br>4,937<br>1	-<br>1,00<br>643	-<br>0,300<br>75	0	-<br>0,188<br>81	-<br>45,63<br>88	-<br>7,839<br>67	0,333<br>491	-<br>0,31<br>671	-<br>69,31<br>18	-<br>53,47<br>85	11,683<br>6
BTzPhO<br>Me6	442	Done	42,89	-<br>2,4377<br>8	glide-<br>grid_5<br>LOF_2	-<br>5,061<br>48	-<br>1,23<br>921	-<br>0,298<br>92	0	-<br>0,259<br>11	-<br>52,99<br>15	-<br>5,502<br>15	0,240<br>388	-<br>0,02<br>973	-<br>71,64<br>82	-<br>58,49<br>37	11,236<br>9
BTz4	94	Done	40,43	-<br>2,3838<br>8	glide-<br>grid_5<br>LOF_2	-<br>4,917<br>28	-<br>0,58<br>304	-<br>0,885<br>34	0	0	-<br>31,08<br>78	-<br>13,55<br>96	0,326<br>906	-<br>0,18<br>748	-<br>57,16<br>9	-<br>44,64<br>74	5,9085<br>5
BTzPhD<br>MN8	382	Done	51,1	-<br>2,2772<br>2	glide-<br>grid_5<br>LOF_2	-<br>4,857<br>42	-<br>1,08<br>861	-<br>0,326<br>2	0	-<br>0,208<br>73	-<br>41,13<br>46	-<br>8,825<br>35	0,248<br>293	-<br>0,10<br>164	-<br>62,75<br>61	-<br>49,95<br>99	4,6345<br>4
BTzPh9	270	Done	48,8	-<br>1,9596<br>1	glide-<br>grid_5<br>LOF_2	-<br>4,340<br>11	-<br>1,76<br>171	0	0	-<br>0,288<br>28	-<br>49,91<br>38	-<br>0,353<br>87	0,333<br>491	-<br>0,07<br>484	-<br>65,38<br>46	-<br>50,26<br>77	7,937
BTzNaf8	196	Done	55,43	-<br>1,7164<br>3	glide-<br>grid_5<br>LOF_2	-<br>4,389<br>03	-<br>1,08<br>317	0	0	-<br>0,391<br>68	-<br>57,38<br>56	-<br>1,863<br>2	0,238<br>059	-<br>0,00<br>348	-<br>71,60<br>07	-<br>59,24<br>88	6,3962<br>9
BA	2	Done	1,74	-<br>1,4257<br>8	glide-<br>grid_5<br>LOF_2	-<br>4,005<br>78	-<br>2,19<br>723	-<br>0,131<br>28	0	-<br>0,295<br>23	-<br>27,53<br>14	-<br>0,953<br>71	0,192<br>727	-<br>0,05<br>514	-<br>34,26<br>82	-<br>28,48<br>51	1,6778<br>1

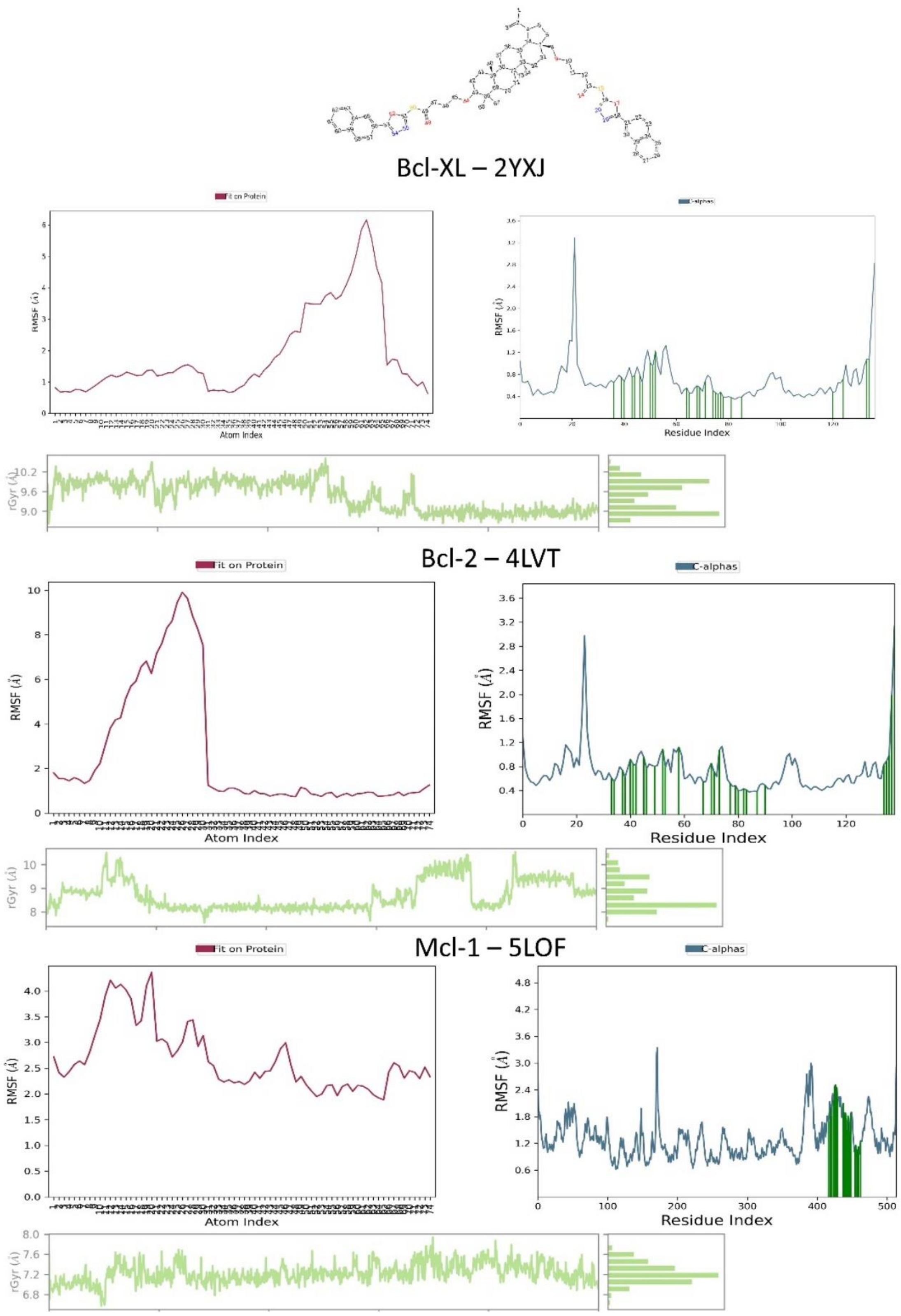

Figure S1. BOxNaf3 flexibility and compactness across Bcl-xL (2YXJ), Bcl-2 (4LVT), and Mcl-1 (5LOF). Top: 2D structure of BOxNaf1 with atom indices. For each target (rows): left, ligand heavy-atom RMSF (frames aligned on protein backbone); right, protein Cα RMSF per residue, (green bars mark contact residues); bottom of each row, ligand radius of gyration (rGyr) time series with distribution inset.

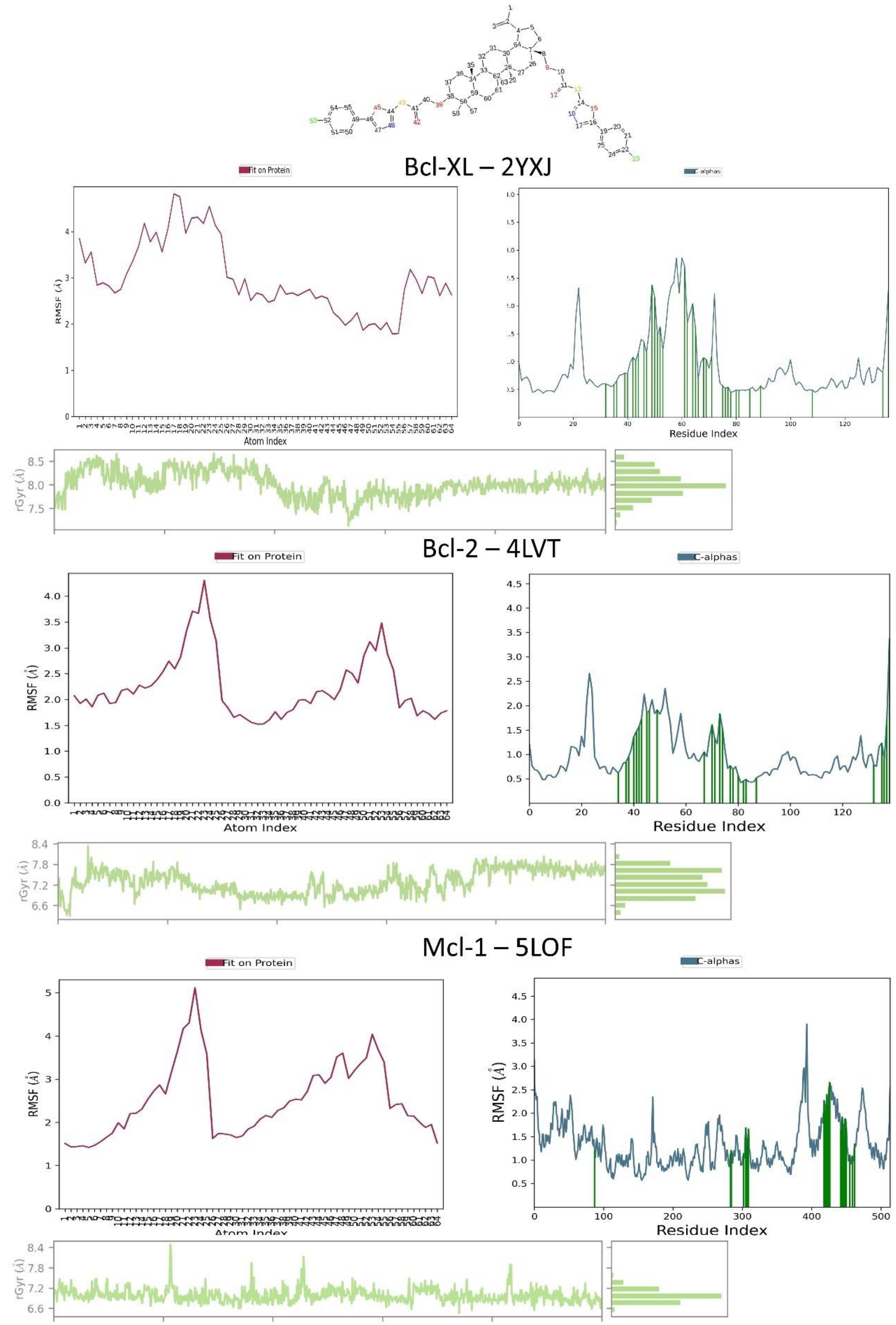

Figure S2. BOxPhCl1 flexibility and compactness across Bcl-xL (2YXJ), Bcl-2 (4LVT), and Mcl-1 (5LOF). Top: 2D structure of BOxNaf1 with atom indices. For each target (rows): left, ligand heavy-atom RMSF (frames aligned on protein backbone); right, protein Cα RMSF per residue, (green bars mark contact residues); bottom of each row, ligand radius of gyration (rGyr) time series with distribution inset.

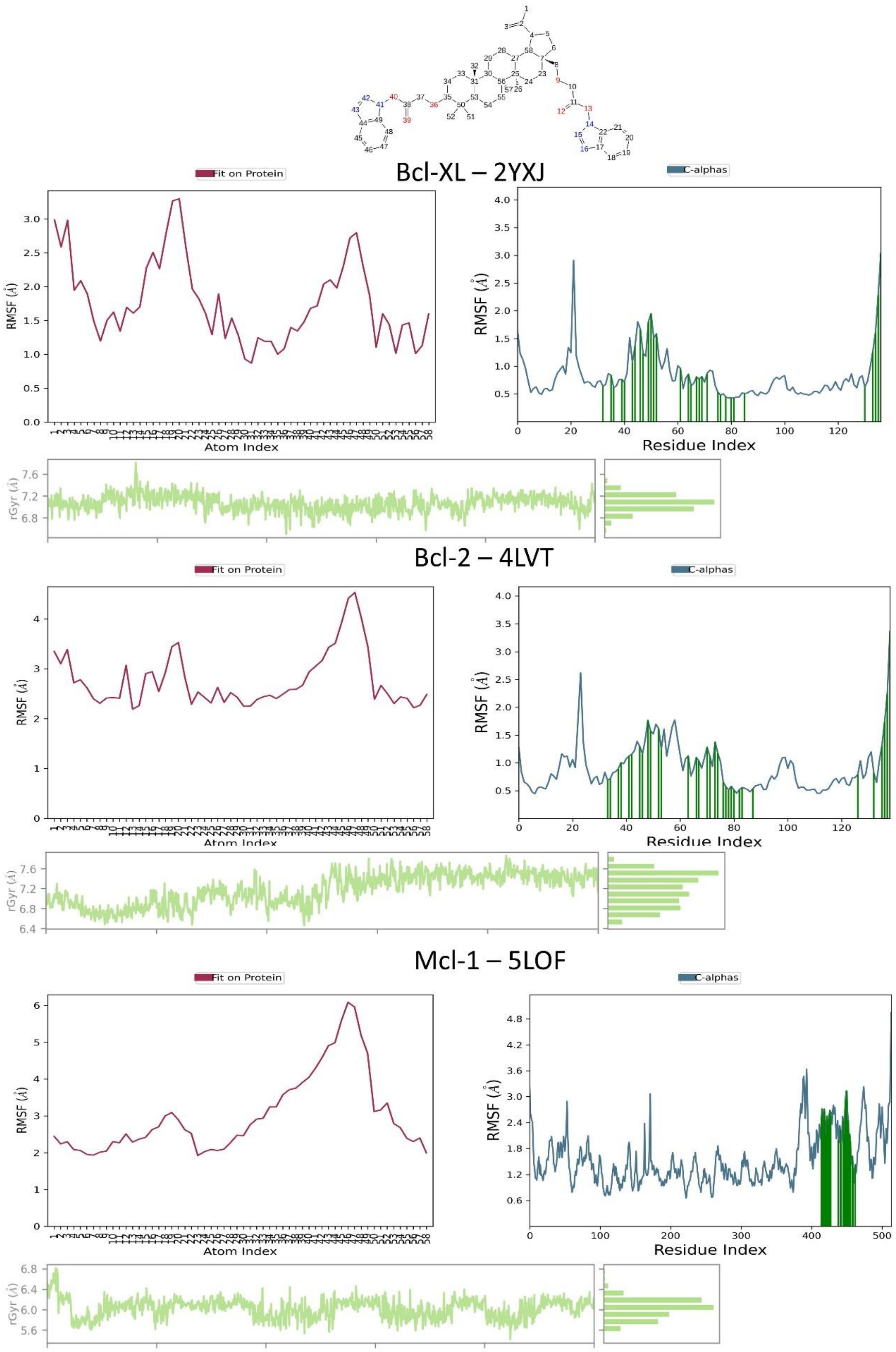

Figure S3. BT1 flexibility and compactness across Bcl-xL (2YXJ), Bcl-2 (4LVT), and Mcl-1 (5LOF). Top: 2D structure of BOxNaf1 with atom indices. For each target (rows): left, ligand heavy-atom RMSF (frames aligned on protein backbone); right, protein Cα RMSF per residue, (green bars mark contact residues); bottom of each row, ligand radius of gyration (rGyr) time series with distribution inset.

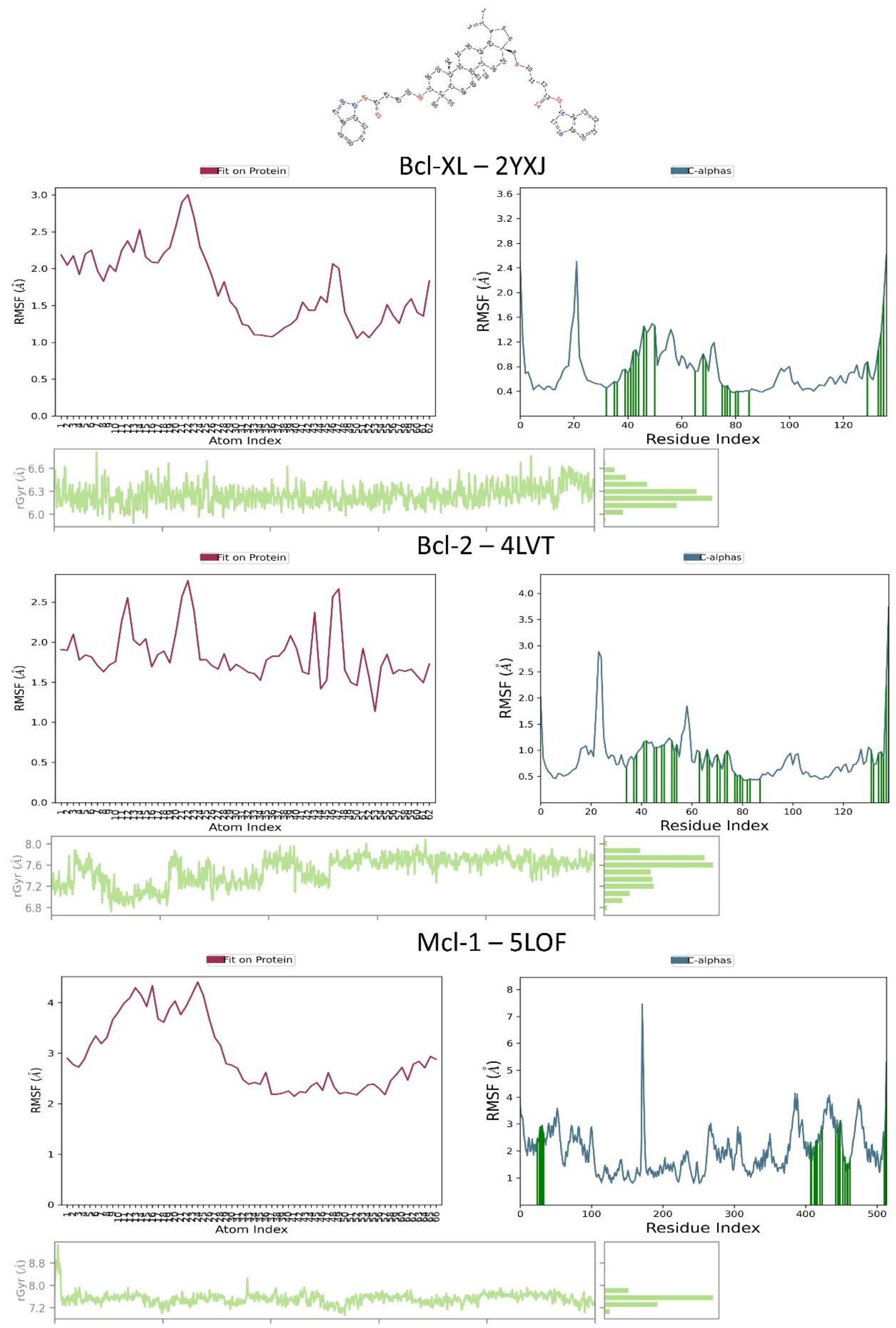

Figure S4. BT3 flexibility and compactness across Bcl-xL (2YXJ), Bcl-2 (4LVT), and Mcl-1 (5LOF). Top: 2D structure of BOxNaf1 with atom indices. For each target (rows): left, ligand heavy-atom RMSF (frames aligned on protein backbone); right, protein Cα RMSF per residue, (green bars mark contact residues); bottom of each row, ligand radius of gyration (rGyr) time series with distribution inset.
